# Coordination electrochemistry taming reversible hypervalent bromine redox for energetic six-electron-transfer lithium-bromine battery

**DOI:** 10.1038/s41467-026-75860-6

**Published:** 2026-07-18

**Authors:** Rumeng Feng, Wenyu Xu, Hongwei Wang, Binfen Wang, Jialin Li, Zelin Chang, Zhaodong Huang, Ze Chen, Xinliang Li

**Affiliations:** 1 School of Physics and Laboratory of Zhongyuan Light, Zhengzhou University, Zhengzhou, China; 2 Department of Materials Science and Engineering, City University of Hong Kong, Hong Kong SAR, P. R. China; 3 Department of Chemical and Biological Engineering, Hong Kong University of Science and Technology, Hong Kong, China; 4 School of Interdisciplinary Studies, Lingnan University, Hong Kong, China

**Keywords:** Batteries, Batteries, Energy, Batteries

## Abstract

Conversion-type static bromine batteries demonstrate promise in high output voltage and large capacity for rechargeable energy storage due to the inherent polyvalent reaction potential. Nevertheless, the combination of the limited two-electron 2Br^-^/Br_2_ redox couple and the redox-inactive organic ligands presents a fundamental bottleneck for the overall specific energy. Herein, ethyl viologen dibromide is developed as an energetically active positive electrode for organic lithium-bromine batteries by efficient coordination chemistry, featuring an advanced six-electron redox mechanism triggered by both intercalation and conversion reactions. The activated redox couple of 2Br^-^/2Br^+^ incubates a collaborative increase in capacity (632.8 mAh g^−1^_Br_) and discharge voltage (3.7 V). Besides, ethyl viologen undergoes reversible two-step intercalation and extraction of Li^+^ ions, contributing to additional energy storage. Reciprocal spectroscopic characterizations and computational electrochemistry indicate the chemisorption effect and interhalogen confinement and detail the dynamic mass-charge transfer pathway. This work sets a paradigm worth emulating for designing high-performance halogen batteries.

## Abstract (web summary)

Conversion-type bromine batteries offer energy-storage potential but face limited electron transfer. Here, authors design an ethyl viologen dibromide positive electrode to unlock a six-electron redox process, boosting capacity and voltage via synergistic bromine conversion and lithium-ion storage.

## Introduction

The development of rechargeable batteries with elevated operating voltages and enhanced capacities remains crucial for portable electronic devices and the integration of renewable energy sources^1–4^. Among various energy storage technologies, lithium batteries emerge as a leading choice due to their prominent electrochemical potential and practical performance^5–10^. Current positive electrode materials in lithium batteries can be classified into two primary categories. Intercalation compounds, exemplified by LiFePO_4_, boast high-voltage plateaus but suffer from modest specific capacities. Conversion-type positive electrodes, represented by S and Se, deliver substantial capacity yet suffer from low output voltages^11–15^.

Achieving a balance between these two electrochemical metrics relies on the expansive valence change window inherent to multielectron redox chemistry^16^. Halogens, distinguished by their inherent multivalent redox capability, stand out as prime candidates for multi-electron transfer systems^17,18^. Iodine manifests high electrochemical versatility across organic, aqueous, and ionic liquid electrolytes. Comprehensive characterization of redox couples in iodine systems, particularly the two-electron process (2I^−^/I_2_, 2.9 V vs. Li^+^/Li, 211 mAh g^−1^) and four-electron pathway (2I^−^/I_2_/2I^+^, 3.4 V vs. Li^+^/Li, 422 mAh g^−1^), establishes a robust theoretical foundation for advancing halogen-based battery research^19–22^. Although Cl-based systems (3.85 V vs. Li^+^/Li) offer a very high theoretical specific capacity, their practical deployment is hampered by the formation of gaseous intermediates, yielding compromised cycling durability and detrimental corrosion. In this regard, Br-based electrochemistry embodies an optimally balanced performance profile wherein two-electron redox chemistry (2Br^−^/Br_2_, 3.5 V vs. Li^+^/Li, 335 mAh g^−1^) surpasses iodine analogs in both operating voltage and capacity, effectively circumventing gaseous intermediates to attain fortified cycling durability. Theoretical analyses suggest that realizing the four-electron redox process (2Br^−^/2Br^+^) would enable a substantial leap in the specific energy and voltage of Br-based batteries^23–25^. However, critical challenges persist, particularly electrolyte decomposition at prohibitive potentials and reversibility limitations during hypervalent state conversion. Addressing both issues proves pivotal to positioning Br-based batteries as competitive next-generation high-energy storage solutions^26,27^.

**Fig. 1:**
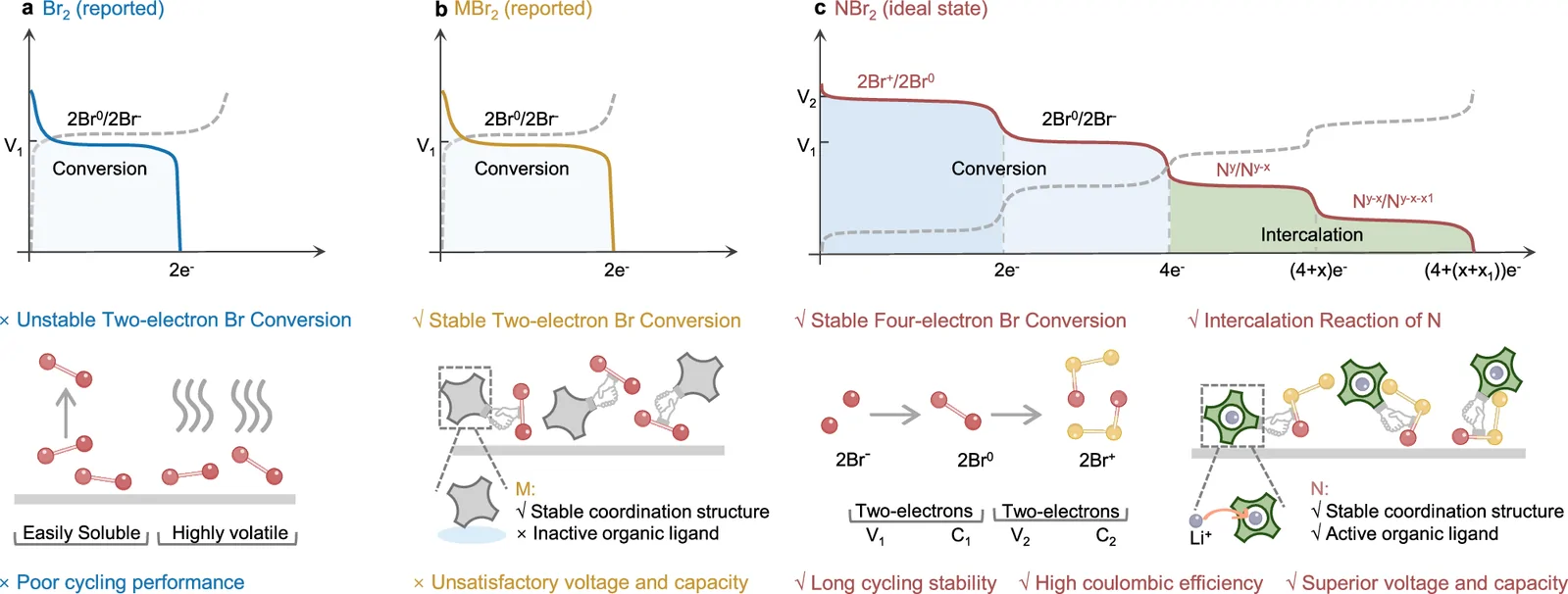
Design principle of the Li–Br battery. Schematic illustration of the challenges of reported bromine batteries and the ideal optimization strategies for advanced bromine batteries. **a** Conventional Br_2_ cathodes undergo a two-electron Br^−^/Br^0^ conversion at a single voltage plateau. However, the high solubility and volatility of bromine species severely limit the conversion extent and reversibility, leading to incomplete redox conversion and poor cycling performance. **b** In reported MBr_2_-type compound cathodes, where M denotes a stable but electrochemically inactive organic ligand, the two-electron Br^−^/Br^0^ conversion is stabilized through coordination, but the resulting voltage output and capacity remain limited. **c** In the proposed ideal NBr_2_ cathodes, N represents a redox-active organic ligand that forms a stable coordination structure with bromine species. This cathode enables a stable four-electron bromine conversion involving sequential Br^−^/Br^0^ and Br^0^/Br^+^ redox couples, while the organic ligand simultaneously provides additional Li^+^ intercalation sites for extra capacity. This design is therefore expected to deliver long cycling stability, high coulombic efficiency, and enhanced voltage output and capacity.

Bromine exhibits inherent volatility and thermodynamic instability under ambient conditions. Such corrosion propensity, fluidity, and the polybromide shuttle effect synergistically drive substantial degradation in capacity utilization and cycling stability when bromine is deployed as a battery active material (Fig. 1a)^28,29^. To overcome the aforementioned operational constraints, Br compound substitution furnishes a practical strategy that suppresses Br_2_ release while reinforcing thermal resilience, collectively improving cycling performance. Representative systems such as TPABr_3_, TBABr_3_, and ODABr_2_ elucidate that ligands can immobilize Br species through strong host–guest chemisorption during redox processes, thereby amplifying reaction kinetics and cycling durability (Fig. 1b)^30^. Nevertheless, electrochemically inert ligands impose inherent mass-volume burdens within electrode materials, fundamentally curbing attainable specific capacity and specific energy ceilings^31,32^. Viologens are classic organic electroactive materials with rigid frameworks^33^. Their structural characteristics enable highly reversible two-electron redox processes (EV^2+^/EV^+•^/EV^0^), fast kinetics, and good stability^34^. These properties underpin their applications in electrochromic devices, redox-flow batteries, and organic electrodes^35,36^. Critically, their dicationic character confers strong electrostatic affinity for polybromide intermediates, making them ideal scaffolds for suppressing shuttle effects in Br-based electrochemistry^37^. Current research on Br redox chemistry is largely confined to two-electron transfer reactions. In contrast, four-electron processes, which provide a pathway to simultaneously higher voltage and greater theoretical capacity, remain critically underexplored. The parasitic disproportionation of hypervalent Br^+^ species imposes a fundamental limitation on electrochemical reversibility^38,39^. The instability of Br^+^ species stems from their strong Lewis acidity and electron-deficient nature, making them prone to reduction. Stabilization via coordination with electron donors is therefore essential, yet halide anions differ drastically in effectiveness. Fluoride (F^−^) binds too strongly, causing overstabilization or unselective oxidation, while I^−^ acts as a reductant and induces decomposition. Cl^−^, by contrast, provides a favorable electronic and steric balance. Its lone-pair electrons delocalize the positive charge and improve thermodynamic stability, while polyhalide assembly affords crucial steric protection^25,40^. In pursuit of maximal Br redox efficiency, advanced electrode architectures necessitate revolutionary conversion mechanisms to unlock multi-electron transfer, with ligands simultaneously functioning as redox-active charge reservoirs via a rocking-chair-type mechanism (Fig. 1c)^18,41,42^.

In this work, we pioneer a six-electron-transfer lithium–bromine (Li–Br) battery anchored on ethyl viologen dibromide (EVBr_2_) as the electrochemically active nucleus. Precise coordination chemistry and electrochemistry actuate reversible four-electron-transfer 2Br^−^/2Br^+^ conversion, affording a definitive 632.8 mAh g^−1^_Br_ capacity paired with a 3.7 V discharge plateau. Concurrently, the EV ligand executes stepwise redox reactions via reciprocal Li^+^ intercalation/extraction, propelling specific energy by 134% to 1810 Wh kg^−1^_Br_. Integrated in situ/ex situ photoelectrochemical characterization and density functional theory (DFT) calculations suggest that ligand-derived chemisorption markedly fortifies Br redox reversibility, while chloride ions from the electrolyte forge stable conversion channels through Br–Cl coordination locking, steering the Br^0^ progressing to Br^+^ transformation trajectory. Holistically, this work demystifies the comprehensive six-electron transfer mechanism, interweaving conversion and intercalation/extraction pathways.

## Results

### Physicochemical properties and electrochemical performance of EVBr_2_

**Fig. 2:**
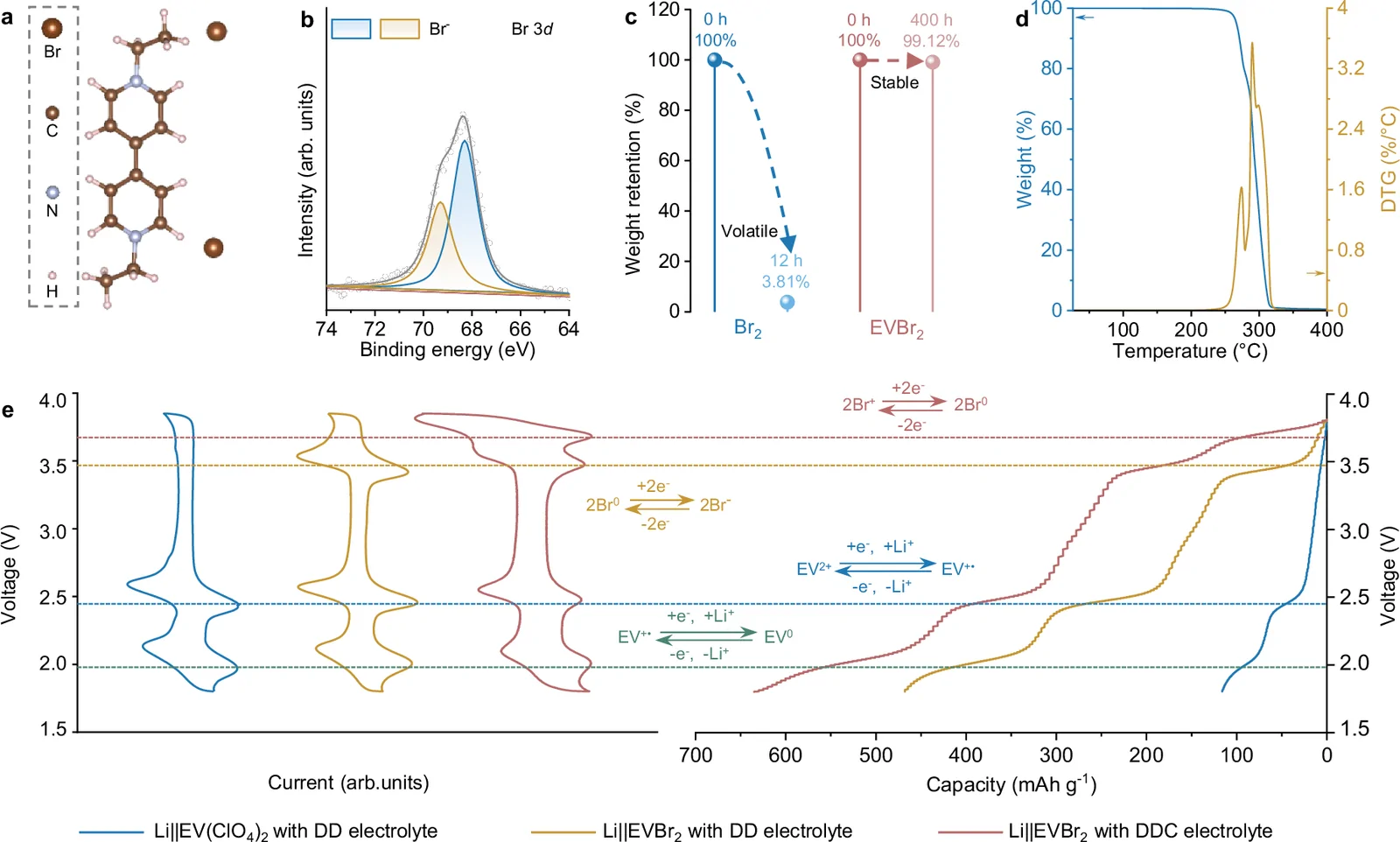
Physicochemical properties and electrochemical performance of ethyl viologen dibromide (EVBr_2_). **a** Molecular model of EVBr_2_. **b** High-resolution Br 3 *d* X-ray photoelectron spectroscopy (XPS) spectra of EVBr_2_. **c** Stability of EVBr_2_ and Br_2_ in an argon-filled glovebox. **d** Thermogravimetric analysis (TGA) curve of EVBr_2_ in N_2_ atmosphere. **e** Cyclic voltammetry (CV) and discharge curves of ethyl viologen diperchlorate (EV(ClO_4_)_2_) in DD (1.0 M LiTFSI in DME: DOL = 1:1 vol% with 1% LiNO_3_) electrolyte, EVBr_2_ in DD electrolyte, and EVBr_2_ in DDC (0.1 M LiCl in DD) electrolyte. The specific capacity of EVBr_2_ is calculated based on the mass of Br, while that of EV(ClO_4_)_2_ is calculated based on the mass of EV(ClO_4_)_2_. All electrochemical and characterization tests were conducted at 25 ± 1 °C, except for the TGA measurement. Source data are provided as a Source Data file.

Ethyl viologen dibromide, an organic ionic compound, comprises a divalent ethyl viologen dication (EV^2+^) and two bromide counterions (Br^−^) held by ionic bonds (Fig. 2a). Scanning electron microscopy (SEM) revealed uniform-sized particles of ~20 μm. Energy-dispersive X-ray spectroscopy (EDS) confirmed homogeneous distribution of C, N, and Br (Supplementary Fig. 1). Valence states of Br were probed via X-ray photoelectron spectroscopy (XPS), with the high-resolution Br 3 *d* spectra resolving two distinct peaks at 68.3 eV and 69.3 eV attributed to Br^−^ species (Fig. 2b and Supplementary Fig. 2). Comparative mass evolution analysis under Ar atmosphere at ambient temperature documents that EVBr_2_ manifests minimal mass loss (<0.9%) after 400 h, dramatically outperforming the conventional Br_2_ positive electrode, which loses nearly all mass within 12 h due to volatilization (Fig. 2c). This contrast underscores the favorable shelf stability of EVBr_2_. Thermogravimetric analysis (TGA) further confirms its thermal stability up to 250 °C, supporting its potential as an advanced electrode material (Fig. 2d). To evaluate its dissolution behavior, EVBr_2_ was immersed in the commercial electrolyte (1.0 M LiTFSI in DME: DOL = 1:1 vol% with 1% LiNO_3_, denoted as DD) for 6 h. The persistence of yellow particles indicated incomplete dissolution, with a solubility of approximately 0.02 M (Supplementary Fig. 3). Under identical conditions, the as-prepared electrode exhibited a lower apparent solubility of ~0.01 M (Supplementary Fig. 4). These results indicate that, despite its intrinsic solubility, EVBr_2_ dissolution is effectively suppressed by encapsulation within conductive carbon and polymeric binders during electrode fabrication.

To delineate the electrochemical redox activity, EVBr_2_ was adopted as the positive electrode material with lithium metal as the negative electrode. The electrochemical behavior of the system was systematically evaluated using a DD electrolyte. Cyclic voltammetry (CV) curves feature signature redox peaks at 3.53/3.42 V, corresponding to the Br^−^/Br^0^ redox couple, demonstrating pronounced electrochemical activity of Br moieties in EVBr_2_ (Supplementary Fig. 5). As anticipated, galvanostatic charge/discharge (GCD) curves exhibited distinct plateaus at the same potentials, indicating Br^−^/Br^0^ redox conversion. The observed prolonged potential plateau attests to the high conversion efficiency, which is comparable to the elemental Br benchmarks. Specifically, the Br-mass-normalized specific capacity reached 468.0 mAh g^−1^_Br_ within the specified electrochemical region.

Extending beyond the characterized Br transformation, two well-defined redox couples materialize at 2.14/1.97 V and 2.60/2.43 V, presenting a marked differentiation from the documented Br redox behavior. The resultant electrochemical fingerprints show a compelling correspondence to the EV^2+^ redox pathways. Experimental validation leveraged halogen-free ethyl viologen diperchlorate (EV(ClO_4_)_2_) as a model compound to decipher EV^2+^ electrochemistry in the same DD electrolyte. At 3 mV s^−1^, the Li | |EV(ClO_4_)_2_ battery displayed accurately aligned redox peaks at identical potentials, while synchronized charge/discharge plateaus affirmed electrochemical consistency in the GCD curves (Supplementary Fig. 6)^43^. Thus, the peak at 2.60/2.43 V corresponds to the single-electron reduction of EV^2+^ coupled with concerted Li^+^ ion intercalation, yielding monovalent radical species (EV^+•^). Subsequent electron transfer at 2.14/1.97 V drives the progressive reduction of EV^+•^, accompanied by secondary Li^+^ ion intercalation, which culminates in the formation of neutral EV^0^. Oxidation pathways precisely retrace the original reaction coordinate in reverse sequence (Fig. 2e)^44,45^. Variable-scan-rate CV analysis establishes unambiguous redox peak resolution even at 20 mV s^−1^, with voltammetric profiles maintaining essential morphological invariance across scan rates, attesting to fast redox kinetics and electrochemical reversibility throughout EV^2+^ bivalent transfer (Supplementary Fig. 7). Multiscale specific current evaluation revealed sustained two-electron plateau signatures persisting under 10 A g^−1^ high-rate operation (Supplementary Fig. 8). Cycling stability assessment, synergized with the acquired electrochemical evidence, collectively substantiates the functional viability of EV^2+^ as a positive electrode material (Supplementary Fig. 9).

Following the feasibility validation of the EV^2+^ positive electrode, a comprehensive electrochemical interrogation of EVBr_2_ in DD electrolyte identified three characteristic redox processes. Dynamic electrochemical diagnostics elucidate dual energy storage mechanisms that encompass stepwise redox transitions of EV^2+^/EV^+•^/EV^0^ coupled with concerted Li^+^ intercalation/extraction, operating alongside Br^−^/Br^0^ conversion chemistry^46^. CV curves across 0.5–50 mV s^−1^ scan rates maintain well-defined profile morphology, though accelerated scanning induces waveform distortion with significant polarization, while Br redox signatures persist unaffected (Supplementary Fig. 10). GCD curves revealed three distinct voltage plateaus that persisted with increasing specific currents (Supplementary Fig. 11). Notably, long-term cycling measurements further confirm the high reversibility and robust stability of this multistep electrochemical reaction (Supplementary Fig. 12). The energy storage mechanism of EVBr_2_ effectively integrates intercalation and conversion reactions, fundamentally circumventing the formation of electrochemically inert components common in bromides while maximizing the electrode material charge storage potential.

Advancing beyond current energy storage limitations, the development of a Br-based electrochemical mechanism via coordination confinement unlocks multi-electron transfer halogen redox chemistry for enhanced specific capacity and specific energy^38^. The incorporation of lithium chloride (LiCl) into DD electrolytes activates multi-electron Br redox chemistry via coordination. Systematic CV measurements with varying LiCl contents reveal that the newly activated redox peaks are most well-defined at a Cl^−^ concentration of 0.1 M (denoted as DDC), indicating the most pronounced electrochemical activity under this condition (Supplementary Fig. 13). Figure 2e displays the CV and GCD curves of the Li | |EVBr_2_ battery in DDC electrolyte, respectively. Distinct from the DD system, a newly emerged redox couple at 3.82/3.68 V presents a substantially narrowed half-peak width and intensified current response compared with the Br^−^/Br^0^ couple^27^. To unambiguously assign this redox feature, an inert positive electrode consisting of Ketjen black (KB) and polyvinylidene fluoride (PVDF) without EVBr_2_ was assembled in the same DDC electrolyte, and the voltage window was extended to 4.0 V (Supplementary Fig. 14). No discernible redox activity was observed around 3.82/3.86 V, confirming that the electrochemical response in this region originates from the Br^0^/Br^+^ conversion in EVBr_2_. Notably, a weak yet distinct redox feature appeared at ~4.0/3.85 V. Given the higher theoretical oxidation potential of the Cl^-^/Cl^0^ redox couple relative to Br^0^/Br^+^, this feature can be reasonably assigned to the Cl^-^/Cl^0^ process. Consistently, extending the upper cut-off voltage of the EVBr_2_-positive electrode to 4.0 V reproduced the corresponding Cl^-^/Cl^0^ redox peaks (Supplementary Fig. 15). Based on these observations, the electrochemical window was confined to 1.8–3.85 V to enable complete and reversible Br conversion while avoiding Cl evolution and its associated issues, such as gas leakage and corrosion. As evidenced by the CV and GCD curves in Fig. 2e, activation of the Br^0^/Br^+^ redox chemistry yields nearly a twofold increase in Br-derived capacity and unambiguously confirms reversible Br^0^/Br^+^ interconversion. Concurrent discharge voltage elevation to 3.7 V substantially boosted specific energy. The establishment of Br-mediated multi-electron transfer pathways culminates in a six-electron transfer Li–Br battery^25^.

### Integrated study of electrolyte/interface behaviors and performance

**Fig. 3:**
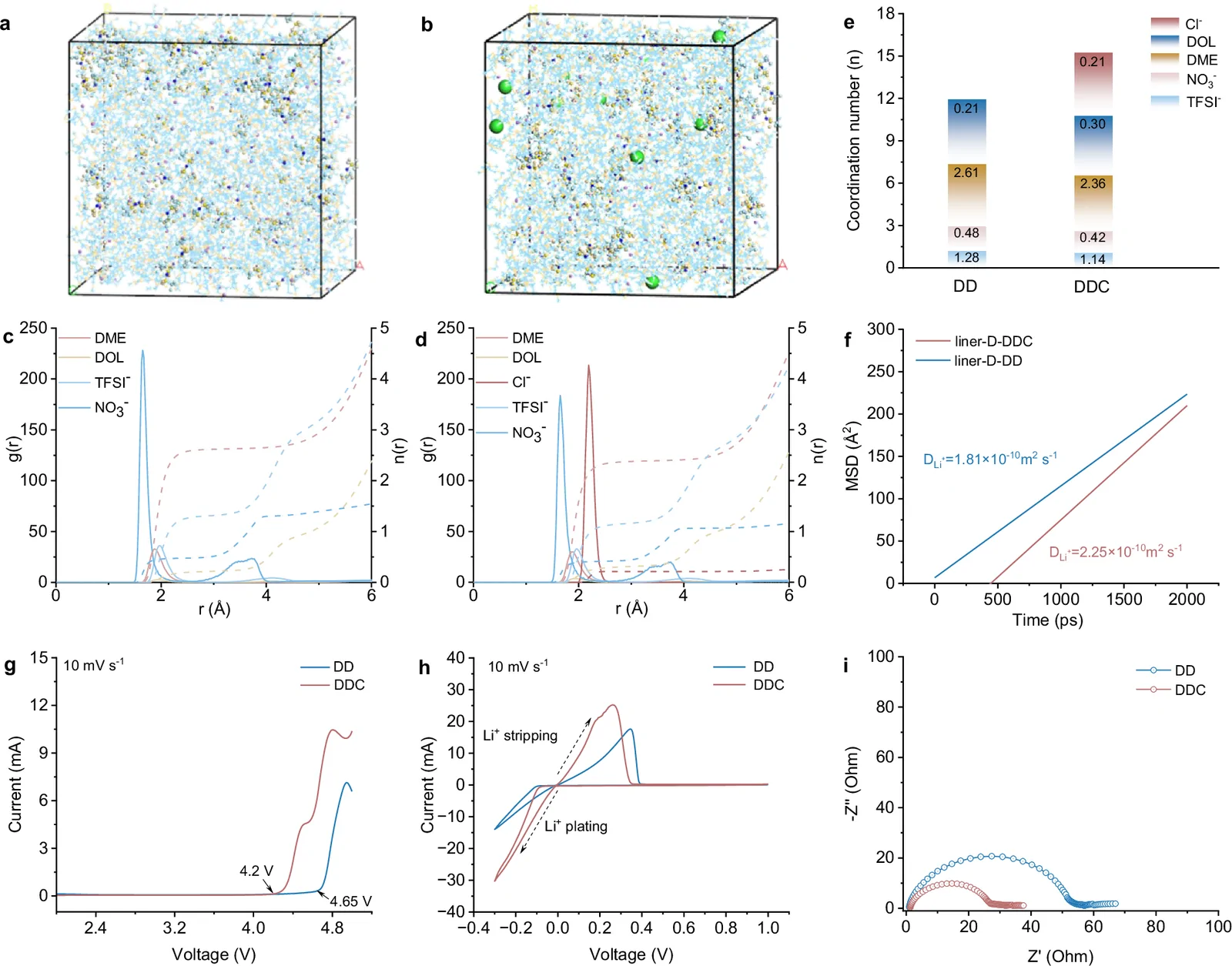
Physicochemical and electrochemical characterization of the electrolytes. **a** Molecular dynamics (MD) simulation snapshots of the DD electrolyte. **b** MD simulation snapshots of the DDC electrolyte. **c** Radial distribution functions (RDFs) of the DD electrolyte obtained from MD simulations. **d** RDFs of the DDC electrolyte obtained from MD simulations. **e** Coordination numbers of Li^+^ with various species (TFSI^−^, NO_3_^−^, DME, DOL, and Cl^−^) in the DD and DDC electrolytes. **f** Li^+^ diffusion coefficients derived from the mean square displacement (MSD) analysis. The simulation temperature was set 298 K. **g** Linear sweep voltammetry (LSV) curves of asymmetric Li | |SS (stainless steel) battery at 10 mV s^−1^. **h** CV curves of asymmetric Li | |SS battery at 10 mV s^−1^. **i** Electrochemical impedance spectroscopy (EIS) spectra of symmetric Li | |Li batteries with DD and DDC electrolytes. All electrochemical and characterization tests were conducted at 25 ± 1 °C. Source data are provided as a Source Data file.

To elucidate the underlying mechanisms, the structural evolution of the electrolyte was examined at the atomic scale^47^. Comparative molecular dynamics (MD) simulations of the bare and LiCl-added electrolytes suggest that the introduced Cl^−^ ions are homogeneously distributed without significant aggregation (Fig. 3a, b and Supplementary Data. 1). Analyses of radial distribution functions (RDFs) and coordination numbers indicate that Cl^−^ ions competitively enter the primary Li^+^ solvation sheath, partially displacing solvent molecules and weakening short-range interactions between Li^+^ and TFSI^−^/NO_3_^−^ (Fig. 3c–e). Consequently, the Li^+^ solvation structure transitions from a solvent-dominated configuration to a Cl^-^-participating coordination environment. Li^+^ exhibits a significant increase in mean square displacement upon LiCl addition, with the diffusion coefficient rising from 1.81 × 10^−10^ to 2.25 × 10^−10^ m^2^ s^−1^, indicating enhanced Li^+^ transport kinetics (Fig. 3f and Supplementary Fig. 16)^48^.

The compatibility of the DDC electrolyte with the lithium metal negative electrode and its resulting interfacial stability were systematically evaluated to assess whether the high electrochemical activity of EVBr_2_ can be sustained during long-term cycling. First, linear sweep voltammetry (LSV) of Li | |SS (stainless steel) batteries shows no significant anodic current up to 4.0 V (vs. Li^+^/Li) in either DD or DDC electrolytes, indicating a sufficiently wide electrochemical window for the Br^0^/Br^+^ redox couple with negligible decomposition (Fig. 3g)^49^. Second, CV curves demonstrate that, in DDC electrolyte, the onset potential for lithium deposition shifts positively with reduced polarization between deposition and stripping peaks, meaning that LiCl effectively improves interfacial kinetics and facilitates more homogeneous lithium deposition (Fig. 3h)^50^. Third, electrochemical impedance spectroscopy (EIS) reveals that Li | |Li batteries in DDC electrolyte exhibit a lower charge transfer resistance (*R*_ct_), signifying the formation of a more conductive electrode-electrolyte interface (Fig. 3i)^40^. Together, these improvements are directly reflected in symmetric Li | |Li batteries, which show reduced voltage polarization in DDC electrolyte during rate and cycling tests, confirming enhanced interfacial stability and kinetics (Supplementary Fig. 17).

To evaluate the shuttle behavior of EVBr_2_ and its impact on the lithium negative electrode, batteries were disassembled at various states of charge (SOCs) after ten cycles for post-cycling analysis. SEM reveals uniform lithium deposition/stripping morphologies, free of dendrites and agglomerates (Supplementary Fig. 18). EDS confirms the absence of bromine on the negative electrode, ruling out shuttle-mediated contamination (Supplementary Fig. 19). Together, these results demonstrate that EVBr_2_ does not undergo significant shuttle during cycling and induces no corrosion or parasitic reactions at the lithium negative electrode. Correspondingly, the separator shows no accumulation of EVBr_2_, confirming that its structure and function remain unaffected (Supplementary Fig. 20).

Transmission electron microscopy (TEM), combined with XPS surface analysis and depth profiling, reveals that the morphology and composition of the solid-electrolyte interphase (SEI) and cathode-electrolyte interphase (CEI) are strongly correlated with the stable cycling performance, underscoring the critical role of interfacial stability^47^. The SEI layer (~40 nm thick) is predominantly composed of electrolyte reduction products, including LiF, Li_3_N (from LiNO_3_ decomposition), Li_2_O/Li_2_CO_3_, and sulfur-containing species (e.g., Li_2_S) derived from the reductive degradation of TFSI^−^ (Supplementary Figs. 21 and 22). In contrast, the CEI is considerably thinner (~3–4 nm) and consists mainly of oxidation products: LiF, nitrogen-containing oxides (e.g., nitrates/nitrites) formed upon oxidative decomposition of LiNO_3_, and high-valence sulfur-oxygen species (such as sulfates) generated from the oxidative breakdown of TFSI^-^ at high potentials (Supplementary Figs. 23 and 24)^48^.

### Electrochemical performance of six-electron-transfer Li | |EVBr_2_ batteries

**Fig. 4:**
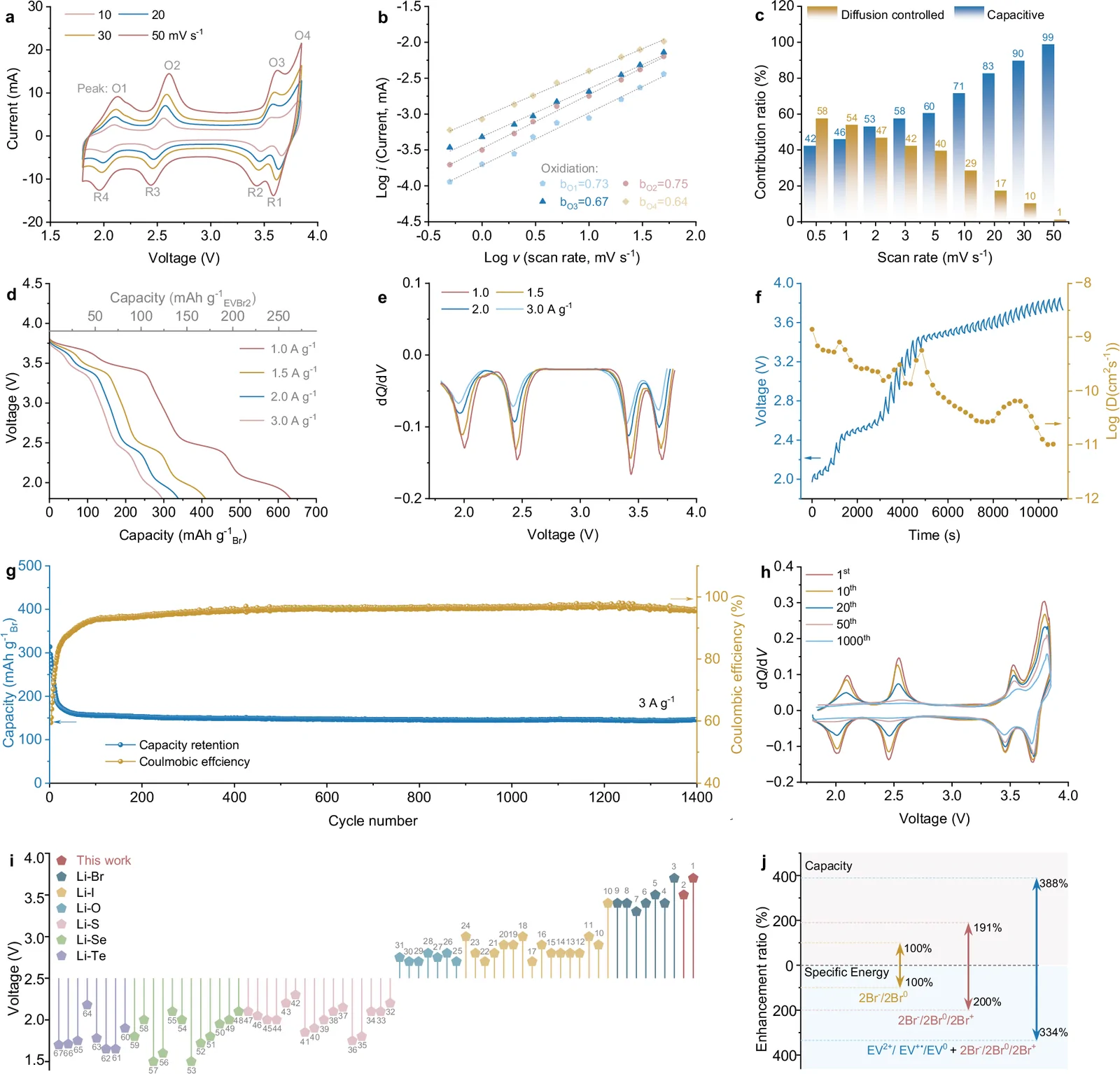
Electrochemical performance of six-electron transfer Li | |EVBr_2_ batteries. **a** CV curves of Li | |EVBr_2_ battery in DDC electrolyte ranging from 10 to 50 mV s^−1^. **b** Calculated *b* values of oxidation peaks. **c** Capacitive and diffusion-controlled contribution ratios at different scanning rates. **d** Discharging curves of Li | |EVBr_2_ battery in DDC electrolyte at different specific currents. **e** Differential capacity (d*Q*/d*V*) curves of Li | |EVBr_2_ battery in DDC electrolyte. **f** Galvanostatic intermittent titration technique (GITT) curves and calculated ion diffusion coefficients of Li | |EVBr_2_ battery in DDC electrolyte. **g** Long-term cycling performance of Li | |EVBr_2_ battery in DDC electrolyte at 3.0 A g^−1^. **h** The d*Q*/d*V* curves of Li | |EVBr_2_ battery in DDC electrolyte at 5.0 A g^−1^. **i** Performance comparison on output voltage between this work and reported conversion-type Li batteries. The source of the literature data shown in this figure can be found in Supplementary Information, Table 1. **j** Comparative analysis of capacity and specific energy improvements enabled by different redox mechanisms. All electrochemical and characterization tests were conducted at 25 ± 1 °C. Source data are provided as a Source Data file.

Kinetic profiling of high-voltage Br^0^/Br^+^ redox behavior was conducted through scan-rate-dependent CV curves in Li | |EVBr_2_ batteries employing DDC electrolyte. Four well-defined redox couples persisted from 0.5 to 5 mV s^−1^ without parasitic peaks, demonstrating high reaction efficiency and stability (Supplementary Fig. 25). Moreover, even at greatly elevated scan rates of up to 50 mV s^−1^, the CV profiles retained structural fidelity, characterized by significantly enhanced electrochemical reversibility compared to that of the DD electrolyte systems (Fig. 4a). Owing to the rapid reaction kinetics, the voltage hysteresis remained negligible as the scan rate increased. According to the power-law relationship *i* = *av*^*b*^, *b* = 0.5 signifies a diffusion-controlled process, while *b* = 1 indicates capacitive domination^51^. Experimentally derived *b*-values for the four redox pairs measure 0.73/0.84, 0.75/0.98, 0.67/0.83, and 0.64/0.90, evidencing synergistic interplay between surface capacitance and diffusion control (Fig. 4b and Supplementary Fig. 26). Quantitatively, through equation *i* (*v*) = *k*_1_*v* + *k*_2_*v*^1/2^, where term *k*_1_*v* quantifies capacitive-controlled current and *k*_2_*v*^1/2^ represents diffusion-limited current, mechanistic dominance was resolved (Fig. 4c)^52^. Specifically, diffusion processes governed 58% of charge storage at 0.5 mV s^−1^, while capacitive contributions exceeded the critical 50% threshold at 2 mV s^−1^.

Preservation of Br^0^/Br^+^ redox conversion kinetics at high scan rates underscores favorable rate capability. The Li | |EVBr_2_ battery delivered specific capacities of 632.8, 409.9, 338.4, and 296.3 mAh g^−1^_Br_ at specific currents of 1.0, 1.5, 2.0, and 3.0 A g^−1^, respectively, featuring four characteristic voltage plateaus (Fig. 4d and Supplementary Fig. 27). Twin high-voltage plateaus are assigned to the Br^+^/Br^0^/Br^−^ four-electron transfer sequence, where near-identical capacities unveil high completeness in Br^+^ to Br^0^ reduction, resolving historically intractable stability and reversibility challenges of Br^+^. The dual low-voltage plateaus are derived from stepwise EV^2+^/EV^+•^/EV^0^ redox reactions, with closely matched capacities, which manifest flawless reversibility devoid of side reactions. The differential capacity curve (d*Q*/d*V*) obtained through galvanostatic curve differentiation unambiguously identifies voltage plateaus and capacity contributions for each reaction step (Fig. 4e). Four resolved peaks correspond precisely to the discharge plateaus, with peak voltages reflecting the respective reduction potentials. Equivalent intensities of Br^+^/Br^0^ and Br^0^/Br^−^ peaks, coupled with analogous magnitudes for EV^2+^/EV^+•^ and EV^+•^/EV^0^ redox couples, demonstrate exact congruence with plateau capacity distributions. The absence of extraneous peaks verified that the electrochemical processes were free from side reactions or byproduct formation. Pronounced peak sharpness further attests to instantaneous charge transfer kinetics^53^.

Galvanostatic intermittent titration technique (GITT) analysis characterized the favorable ion diffusion kinetics of Li | |EVBr_2_ battery (Fig. 4f)^54^. Sequential charge plateaus measurement across four distinct redox stages revealed minimal polarization. Recorded overpotentials of 51 mV, 46 mV, 52 mV, and 122 mV confirm rapid ion transport (Supplementary Fig. 28). Calculated ion diffusion coefficients, ranging from 10^−9^ to 10^−11 ^cm^2 ^s^−1^, further substantiate rapid ionic mobility and good rate capability^55^. Concurrently, the battery maintained good cycling stability, with an average capacity decay rate of merely 0.03% per cycle over 1400 cycles at 3 A g^−1^ (Fig. 4g and Supplementary Fig. 29). Analysis of the GCD profiles and corresponding d*Q*/d*V* curves at 5 A g^−1^ shows that signatures of the EV^2+^/EV^+•^/EV^0^ redox couples fade over the initial cycles, while those associated with bromine redox persist without significant decay (Fig. 4h and Supplementary Fig. 30). The degradation of these organic redox signals is ascribed to repeated Li^+^ insertion/extraction, which induces cyclical volume changes and the accumulation of mechanical stress, thereby compromising the electrical contact between the active material and the conductive matrix. The robustness of the Br redox conversion can be attributed to the effective confinement of Br species within the EV^2+^ framework, a mechanism that suppresses dissolution and thereby maintains electrochemical activity (Supplementary Fig. 31 and Supplementary Data. 2). Furthermore, this strong Br adsorption capability also underlies the significantly mitigated self-discharge observed for the battery (Supplementary Fig. 32).

EVBr_2_ delivers a high operating voltage of 3.7 V through the Br^0^/Br^+^ redox couple, as established by systematic benchmarking in Fig. 4i. This value significantly exceeds that of conversion-type Li–S, Li–Se, and Li–Te systems (typically below 2.2 V) and also surpasses both all reported Li–I batteries and conventional two-electron (2Br^−^/Br_2_) Li–Br batteries. Both conventional two-electron Li–Br reactions (3.5 V) and Li–I_2_ batteries with either two-electron (2.9 V) or four-electron (3.4 V) operation demonstrate substantially lower performance metrics^15,29,56–67^. The developed six-electron transfer electrochemistry confers substantial enhancements in both capacity and specific energy. Figure 4j documents that four-electron Br transfer elevates discharge capacity to 191% of the baseline and specific energy to 200%, achieved through activation of a 3.7 V voltage plateau. Subsequent EV^2+^ participation further augmented both metrics to 388% and 334%, respectively. Consequently, the resulting Li | |EVBr_2_ battery delivered a specific energy of 1810 Wh kg^−1^_Br_ at a specific current of 1 A g^−1^, surpassing all previously reported two-electron (2Br^−^/Br_2_) Li–Br batteries^17,40^.

### Spectral analysis of six-electron-transfer electrochemistry

**Fig. 5:**
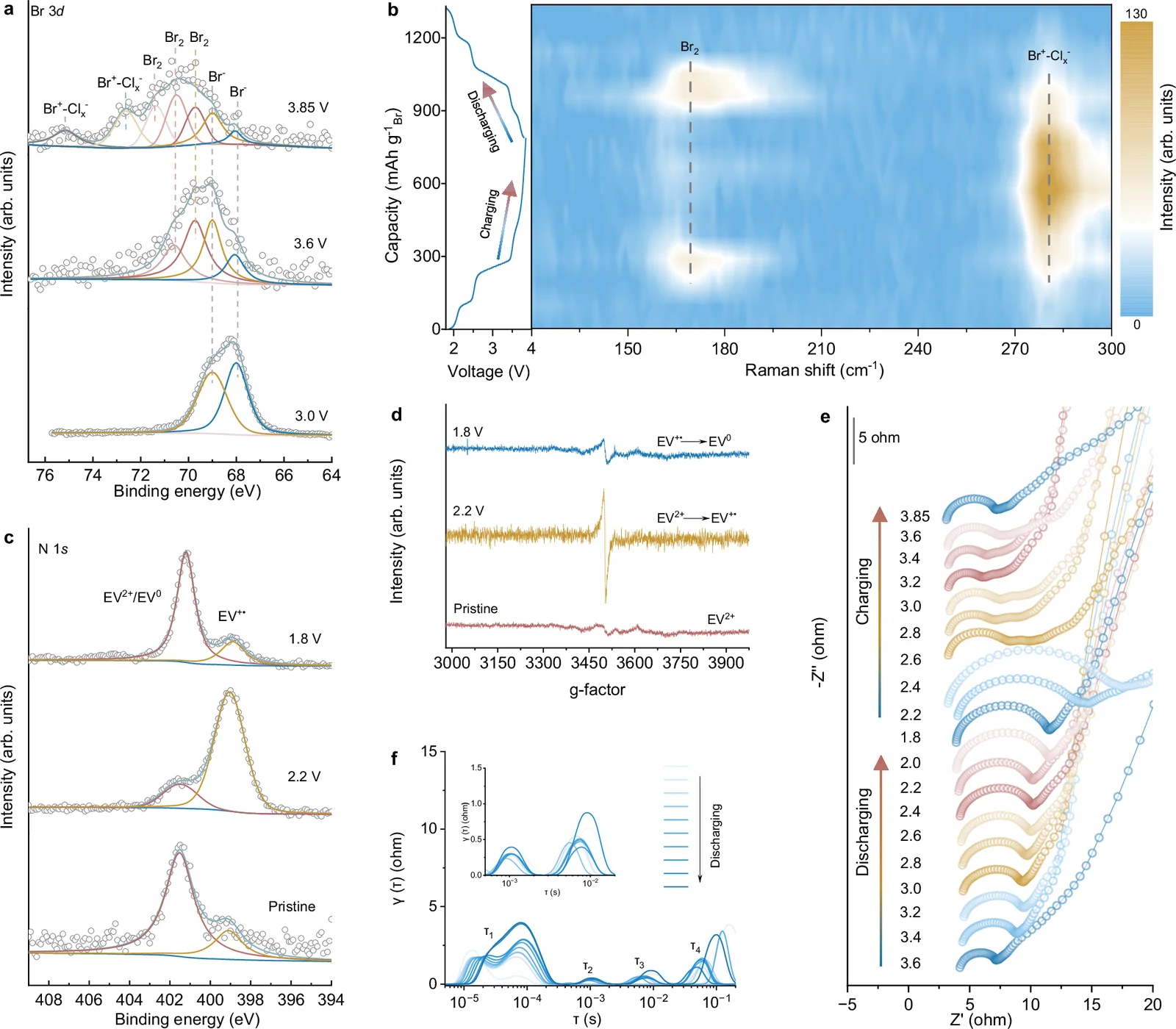
Spectral analysis of six-electron-transfer electrochemistry. **a** High-resolution Br 3 *d* XPS spectra at selected states of charge (SOCs). **b** Galvanostatic charge/discharge (GCD) curve of the battery cycled at 0.5 A g^−1^ during in situ Raman measurements (left) and the corresponding in situ Raman spectra recorded during charging and discharging (right). **c** High-resolution N 1 *s* XPS spectra at selected SOCs. **d** Electron paramagnetic resonance (EPR) spectra at selected SOCs. **e** In situ EIS spectra collected at selected SOCs during cycling at 1 A g^−1^. **f** Corresponding distribution of relaxation times (DRT) results for the discharging process. **a**, **c**, **d** The electrodes were obtained from batteries that were first cycled at 1 A g^−1^ for 10 cycles, then charged or discharged to the indicated SOCs, after which the batteries were disassembled. All electrochemical and characterization tests were conducted at 25 ± 1 °C. Source data are provided as a Source Data file.

Employing multimodal synergetic in situ and ex situ spectroscopy enables the tracking of reaction pathway evolution and interfacial reconfiguration, as well as the deciphering of chemisorption and halogen confinement to map six-electron transfer scenarios in EVBr_2_. XPS analysis in Fig. 5a directly captures the valence state evolution of Br species in the EVBr_2_-positive electrode across SOCs variations (Supplementary Fig. 33). During the charging phase, the deconvolution of the Br 3 *d* spectrum at 3.0 V revealed two peaks at 68.0 eV and 69.0 eV, which are characteristic of Br^−^. Progressive oxidation at 3.6 V generates four distinct signatures at 68.0, 69.0, 69.7, and 70.6 eV, assignable to Br_2_ species derived from the oxidation of Br^−^^27,40^. Further voltage elevation to 3.85 V yields fitting peaks at 72.6 eV and 75.1 eV, unambiguously diagnostic of Br^+^ formation via Br^0^ oxidation^40^.

In situ Raman spectroscopy was used to monitor the reversible four-electron Br conversion dynamics in real time (Fig. 5b and Supplementary Fig. 34). During charging to 3.6 V, the original Raman peak at 150–190 cm^−1^ progressively attenuates, while a nascent peak at 270–290 cm^−1^, characteristic of the Br^+^–Cl_x_^−^ species, emerges and intensifies with increasing voltage, reaching its maximum intensity at 3.85 V^25,28,40,68^. Upon subsequent discharge, the Br^+^–Cl_x_^−^ peak gradually decreases, and the intensity of the 150–190 cm^−1^ band recovers correspondingly^25,27,31,46^. The synchronous emergence and extinction of spectral features conclusively established Br^−^ redox reversibility.

Tracking the dynamic valence states during the EV^2+^ redox reactions, XPS interrogation leveraging an elemental N calibrator mapped the progressive electronic state transformations. High-resolution N 1 *s* spectra acquired at sequential discharge states are shown in Fig. 5c. At open-circuit voltage, a primary peak appeared at 401.2 eV in the XPS spectrum, which is characteristic of the dicationic EV^2+^ species (Supplementary Fig. 35). A secondary feature at 398.8 eV arises from the ethyl viologen radical cation (EV^+•^), induced transiently under X-ray irradiation in the analysis chamber. Upon discharge to 2.2 V, the radical-associated signal surged concomitantly with the significant decay of the EV^2+^ peak, unambiguously confirming the one-electron reduction of EV^2+^ to EV^+•^. Further discharging to 1.8 V and 1.7 V precipitated a striking spectral metamorphosis, defined by the dramatic intensification and sharpening of the 401.2 eV peak concurrent with the pronounced attenuation of the 398.8 eV radical marker, culminating in residual trace intensity (Supplementary Fig. 36). The observed spectral transformation denotes subsequent reduction of EV^+•^ to the neutral species EV^0^, a tertiary nitrogen compound exhibiting inherent binding energy at 401.2 eV. The weak residual peak at 398.8 eV originates from a minor amount of EV^+•^ formed via reaction of EV^0^ and EV^2+^^44,45,69^.

In addition, electron paramagnetic resonance (EPR) spectroscopy was used to track the spin dynamics of EV^2+^ species, linking radical fluxes to electrochemical states (Fig. 5d). In the absence of applied voltage, EPR measurements revealed no detectable signal, consistent with the diamagnetic character of the EV^2+^ species^70^. Discharging to 2.2 V manifested an intense paramagnetic resonance signal, which indicated the one-electron reduction of EV^2+^ to radical species EV^+•^. Further discharging to 1.8 V completely eliminated the radical signal, with only marginal spectral remnants observed. The described spectral progression reflects conversion of EV^+•^ to neutral EV^0^, with the weak residual response ascribed to minor EV^+•^ regeneration via EV^0^/EV^2+^ interactions.

Acting as a precise probe, in situ EIS resolved the reaction kinetics throughout a four-step redox process (Fig. 5e). The semicircles observed in the high-frequency and mid-frequency regions correspond to ion migration and *R*_ct_, respectively. During charging, *R*_ct_ surged from 9.3 Ω at 2.2 V to 13.0 Ω at 2.4 V, potentially reflecting Li^+^ extraction, SEI film formation, or limitations of electrolyte wetting^71^. Between 2.6 and 3.4 V, *R*_ct_ decreased with increasing voltage but slightly increased to 3.7 Ω at 3.6 V. The sharp decrease in *R*_ct_ to 3.6  Ω at 3.85 V demonstrates accelerated oxidation kinetics for Br^0^/Br^+^ versus Br^−^/Br^0^ (Supplementary Fig. 37). During discharge, *R*_ct_ progressively escalated with declining voltage, exhibiting a distinct variation at around 3.2 V, where *R*_ct_ initially rose and then fell. This behavior implies a mechanistic transition from Br conversion reactions to Li^+^ intercalation/extraction (Supplementary Fig. 38). Notably, the inverse correlation between *R*_ct_ and voltage fluctuations, coupled with lower *R*_ct_ values during discharging than during charging, confirms the enhanced electrode-electrolyte interfacial compatibility. The ohmic resistance (*R*_s_) maintained stable low values (3.3–4.9 Ω) throughout cycling, consistent with rapid reaction kinetics in the electrochemical system^40^.

Distribution of relaxation times (DRT) decodes interfacial charge transfer kinetics through identified time constants that discriminate multistep electrochemical processes. High-frequency γ(τ) signatures are assigned to SEI film formation or charge transfer reactions, whereas low-frequency components reflect promoted Li^+^ migration within the electrode material^38^. Across the 10^−5^–10^−1 ^s relaxation spectrum, four characteristic peaks (τ_1_ − τ_4_) were resolved. The τ_1_ peak intensity, a direct measure of the interfacial contact resistance, decreased during charging (Supplementary Fig. 39). Notably, the discharged-state values were consistently and substantially lower than the charged-state values throughout cycling (Fig. 5f). These observations demonstrate improved electrode-electrolyte interfacial wettability. Interphase resistance governed by τ_2_ regulates the Li^+^ flux across the electrode-electrolyte boundary, in contrast to the τ_3_-assigned charge transfer resistance in redox processes. The progressive decrease in τ_3_ peak intensity during charging indicates accelerated electrochemical kinetics. Although τ_4_ represents Cl^-^ diffusion through the electrolyte, the intensity of the τ_4_ peak only slightly increased, confirming the sustained suppression of the energy barrier governing polybromide conversion. The consistently attenuated γ(τ) profiles proclaim accelerated reaction kinetics that modulate both halogen conversion and Li^+^ intercalation mechanisms^42^.

### Theoretical simulations of the six-electron-transfer redox mechanism

**Fig. 6:**
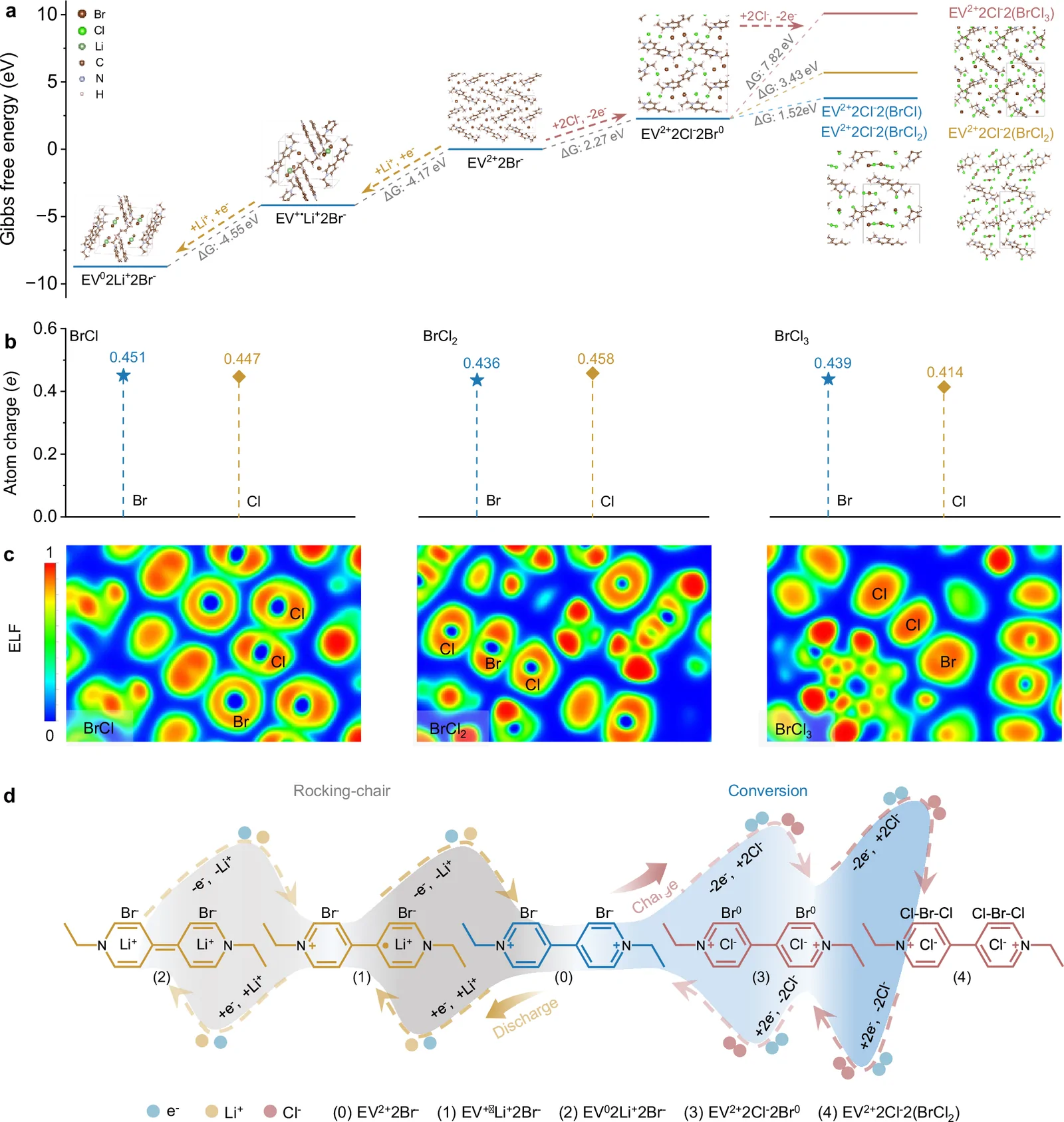
Theoretical simulations of a six-electron-transfer redox mechanism. **a** Gibbs free energy (Δ*G*) of possible redox products. **b** Atomic charges of BrCl, BrCl_2_, and BrCl_3_. **c** Electron localization function (ELF) of redox products. **d** Diagram of the six-electron reaction mechanism of Li | |EVBr_2_ battery. Source data are provided as a Source Data file.

Systematic DFT simulations offer potential atomic-level insight into the six-electron transfer pathways in the Li | |EVBr_2_ battery, delineating the coupled dynamics between interfacial charge reorganization and binding evolution. Following optimization of the EVBr_2_ crystal structure, the Gibbs free energy (Δ*G*) of the redox processes was calculated (Fig. 6a and Supplementary Fig. 40). During 3.0–1.8 V discharging, sequential reduction proceeds from EV^2+^ through EV^+•^ to EV^0^. As shown in Eqs. (1) and (2), electron acquisition processes synchronized with Li^+^ intercalation generated Δ*G* values of −4.17 eV and −4.55 eV (Supplementary Fig. 41). Equation (3) shows that bromide oxidation (Br^−^/Br^0^) during charging in the 3.0–3.6 V range requires a Δ*G* of 2.27 eV. Structural analyses suggested the absence of Br–Cl bonding configurations, indicating that Cl^-^ primarily functions as a charge compensator (Supplementary Fig. 42). Further oxidation at 3.85 V leads to Br^0^ to Br^+^ conversion and generates halogen coordination motifs including BrCl, BrCl_2_, and BrCl_3_, with corresponding Δ*G* values of 1.52, 3.43, and 7.82 eV (Supplementary Fig. 43 and Supplementary Data. 3).

Atomic charge distributions across BrCl_x_ systems were computed to help elucidate interactions among Br, Cl, and surrounding environments (Fig. 6b). Compared to BrCl and BrCl_2_, the average charge transfer from EV to Br and Cl remained relatively constant, while BrCl_3_ exhibited lower atomic charges on Br and Cl. Such attenuated charge transfer from EV to Br and Cl in BrCl_3_ suggests weakened bonding affinity, which may impede Br–Cl bond formation.

Bonding stability evolution in the BrCl_x_ (*x* = 1–3) series was further tracked through electron localization function (ELF) mapping, which helps to uncover distinct atomic-scale electron density redistribution phenomena. Figure 6c shows green regions between Cl and Br atoms in BrCl and BrCl_2_, suggesting intermediate electron localization and delocalization states. Uniform Br–Cl electron distribution may indicate partial covalent bonding character. In contrast, BrCl_3_ displayed no localized Cl-Br interatomic states. Distinct charge separation indicates the absence of bonding interactions, which is consistent with energetic instability in BrCl_3_^40^. However, the amorphous BrCl matrices incorporated both BrCl and BrCl_2_ species, as suggested by structural analysis (Supplementary Fig. 43a). Consequently, BrCl_2_ constitutes the thermodynamically favored Br–Cl structure, with a corresponding Δ*G* of 3.43 eV according to Eq. (4).

Although the DFT models do not fully capture the complexity of the real electrochemical interface under operating conditions, they provide useful insights into the intrinsic interaction mechanism. The four-step reaction model and exact equations of EVBr_2_ were reasonably constructed based on harvested experimental evidence and theoretical calculations (Fig. 6d). The established framework suggests a six-electron transfer mechanism orchestrated by intercalated charge-halogen coordination locking, outlining the fundamental reaction pathway of the Li||EVBr_2_ battery.

1 $${{\rm{E}}}{V}^{2+}2B{r}^{-}+L{i}^{+}+{e}^{-}\to E{V}^{+\cdot }L{i}^{+}2B{r}^{-}\varDelta G=-4.17\,eV$$

2 $${{\rm{E}}}{V}^{+\cdot }L{i}^{+}2B{r}^{-}+L{i}^{+}+{e}^{-}\to E{V}^{0}2L{i}^{+}2B{r}^{-}\varDelta G=-4.55\,eV$$

3 $${{\rm{E}}}{V}^{2+}2B{r}^{-}+2C{l}^{-}\to E{V}^{2+}2C{l}^{-}2B{r}^{0}+2{e}^{-}\varDelta G=2.27\,eV$$

4 $${{\rm{E}}}{V}^{2+}2C{l}^{-}2B{r}^{0}+2C{l}^{-}\to E{V}^{2+}2C{l}^{-}2(BrC{l}_{2})+2{e}^{-}\varDelta G=3.43\,eV$$

## Discussion

In summary, we introduce a six-electron transfer paradigm in Li–Br batteries, achieved through precise molecular engineering of EVBr_2_ positive electrodes. Tailored coordination chemistry triggers the sequential 2Br^−^/2Br^0^/2Br^+^ redox couple, delivering a specific capacity of 632.8 mAh g^−1^_Br_ alongside a remarkably high 3.7 V discharge plateau among conversion-type systems. Consecutive two-step Li^+^ storage via EV^2+^ ligands boosts the specific energy by 134% compared to state-of-the-art conversion positive electrodes. Mechanistic decoupling revealed that ligand-derived chemisorption creates molecular anchoring points that stabilize Br species, while coordination locking simultaneously mediates BrCl_2_ formation, constructing efficient charge transfer pathways for Br valence transitions. Such synergistic effects enable long-term cycling durability, with 0.03% per-cycle fading over 1400 cycles. Concerted energy barrier reconfiguration during conversion reactions and ordered ion transport during intercalation/extraction surmount the intrinsic limitations of conventional two-electron Br electrochemistry. Atomically elucidated Br transition pathways and halogen-bonded charge channels provide a universal design framework for energetic halogen batteries, cementing multi-electron transfer as the cornerstone of next-generation energy storage.

## Methods

### Materials

All reagents and starting materials were used as received without further purification: ethyl viologen dibromide (EVBr_2_, Macklin, 98%), ethyl viologen diperchlorate (EV(ClO_4_)_2_, Macklin, 98%), potRassium bromide (KBr, Macklin, 99%), Ketjen black (KB, ECP-600JD, DoDoChem), polyvinylidene fluoride (PVDF, Aladdin) binder, N-Methylpyrrolidone (NMP, 99%, Aladdin), lithium chloride (LiCl, Aladdin, 98%), commercial electrolyte (1.0 M LiTFSI in DME: DOL = 1: 1 vol% with 1% LiNO_3_, DoDoChem), lithium foil (Li, Canrd, >99.9%), and carbon cloth (HCP331, Canrd).

### Preparation of EVBr_2_-positive electrodes and electrolyte

EVBr_2_ powder, KB, and PVDF were mixed in NMP solvent with a mass ratio of 6:3:1. After vigorously stirring and grinding under ambient air at 25 ± 1 °C for 1 h, the homogeneous slurry was manually coated onto a flexible carbon cloth substrate (380 μm in thickness) using a doctor blade, and then vacuum-dried at 60 °C for 24 h. The areal mass loading of EVBr_2_ was adjusted to 1.5–2.0 mg cm^−2^ by controlling the slurry thickness. For comparison, EV(ClO_4_)_2_ and KBr positive electrodes were prepared under the same conditions. In addition, an inert electrode was fabricated by blending KB and PVDF in a mass ratio of 9:1. The electrolyte was prepared within an argon-filled glovebox at 25 ± 1 °C by dissolving LiCl at concentrations of 0.02 M, 0.06 M, and 0.1 M in a commercial organic electrolyte (1.0 M LiTFSI in DME: DOL = 1: 1 vol% with 1% LiNO_3_) under vigorous stirring until complete dissolution was achieved.

### Characterization

The sample morphology and microstructure were characterized using a field-emission scanning electron microscope (SEM, ZEISS Gemini 300) at an accelerating voltage of 3 kV and high-resolution transmission electron microscopy (HRTEM, Tecnai 12) at 200 kV. The elemental composition was analyzed by energy-dispersive X-ray spectroscopy (EDS) with an accelerating voltage of 20 kV. In situ Raman spectroscopy was performed on a Horiba LabRAM HR Evolution system (532 nm laser excitation) in combination with a dedicated electrochemical testing device. Surface chemical states were determined by X-ray photoelectron spectroscopy (XPS, Thermo Scientific K-Alpha) using monochromatic Al Kα radiation with a spot size of 400 μm, an operating voltage of 12 kV, a filament current of 6 mA, a pass energy of 150 eV for survey scans, and a pass energy of 50 eV for high-resolution spectra. Free radical dynamics were monitored by electron paramagnetic resonance spectroscopy (EPR, Bruker EMX-9.5/12). Thermogravimetric analysis (TGA) was performed on a HITACHI STA200 under nitrogen flow at a heating rate of 10 °C min^−1^ from 26 to 400 °C. The stability of the material was tested in an argon-filled glovebox (H_2_O and O_2_ < 0.1 ppm) at 25 ± 1 °C.

### Electrochemical measurements

The CR2032 coin-type battery was assembled in an argon-filled glovebox using the synthetic EVBr_2_ electrode described above, which was punched into disks of 10 mm in diameter, as the positive electrode, Celgard 2325 (diameter =  19 mm, thickness = 20 μm) as the separator, and commercial lithium metal foil (diameter = 10 mm, thickness = 300 μm) as the negative electrode. Li | |EV(ClO_4_)_2_ battery and Li | |KB battery were prepared under identical conditions. The electrolyte (40 μL) and Li metal negative electrode were used in excess to ensure the complete electrochemical conversion of the explored positive electrodes. The assembled batteries can be directly subjected to electrochemical testing without any resting period. The open-circuit voltage is approximately 3.0 V, with a fluctuation of 0.1 V. In symmetric Li | |Li batteries, two identical Li metal electrodes served as both the negative electrode and the positive electrode in different electrolytes. In asymmetric batteries, Li metal was used as the negative electrode, while stainless steel served as the positive electrode. LSV, CV, and EIS were performed using a CHI760E electrochemical workstation. For in situ EIS measurements, the battery was charged to selected voltages, after which the EIS spectra were recorded. DRT profiles was generated from EIS data via mathematical transformation algorithms implemented in MATLAB. Galvanostatic discharge and charge measurements, as well as GITT tests, were performed on a LAND CT3002A system at 25 ± 1 °C using a constant temperature chamber.

### Analysis of ion diffusion coefficients by the GITT method

The lithium-ion diffusion coefficient was determined via GITT and calculated using the following equation:

5 $${D}_{{{{{\rm{Li}}}}}^{+}}=\frac{4}{{{{\rm{\pi }}}}\tau }{\left(\frac{{m}_{{{{\rm{B}}}}}{V}_{{{{\rm{m}}}}}}{{M}_{{{{\rm{B}}}}}S}\right)}^{2}{\left(\frac{\varDelta {E}_{{{{\rm{S}}}}}}{\varDelta {E}_{{{{\rm{t}}}}}}\right)}^{2}$$

The symbols *τ*, *m*_B_, *S*, *M*_B_, and *V*_m_ denote relaxation time, active material mass, electrode area, molar mass, and molar volume of the electrode material, respectively. *∆E*_S_ and *∆E*_t_ represent the voltage drop during the relaxation period and galvanostatic charge-discharge step, respectively^72–74^.

### In situ Raman measurement

In situ Raman spectroscopy measurements were performed using a Horiba LabRAM HR Evolution system equipped with a 532 nm laser. Spectra were recorded every 120 s in the range of 120–300 cm^−1^. The top of the in situ Raman cell was made of transparent quartz. Inside the cell, an EVBr_2_-positive electrode, a separator, and a lithium disk were stacked from top to bottom, with an electrolyte volume of 40 μL. During the in situ Raman data acquisition, the battery was cycled at a specific current of 0.5 A g^−1^.

### In situ EIS measurement

EIS measurements were conducted at 25 ± 1 °C. The batteries were galvanostatically charged at a specific current of 1 A g^−1^ to different states of charge, followed by a 600 s rest period before EIS measurements. The impedance spectra were recorded in potentiostatic mode with an AC amplitude of 5 mV over a frequency range from 100 kHz to 0.01 Hz, with a total of 85 data points collected. The discharge process was carried out using the same procedure.

### Ex situ characterization

For ex situ XPS, EPR, SEM, and TEM measurements, the assembled Li | |EVBr_2_ batteries were cycled 10 times at 1 A g^−1^ and then charged or discharged to different states of charge. The batteries were subsequently transferred to an argon-filled glovebox maintained at 25 ± 1 °C for disassembly. The cycled EVBr_2_ positive electrodes were retrieved, rinsed three times with 1,2-dimethoxyethane (DME) to remove residual electrolyte, and then taken out of the glovebox for measurements without vacuum protection. In contrast, the lithium metal negative electrodes were also rinsed three times with DME, but were transferred out of the glovebox using a vacuum transfer vessel to minimize exposure to ambient air.

### MD and DFT calculations

MD simulations were performed using the GROMACS package with the OPLS force field to investigate the solvation structures of the electrolyte^75^. The simulations were carried out under periodic boundary conditions, with a 15 Å cut-off applied to both van der Waals and short-range electrostatic interactions, while long-range electrostatic forces were treated using the particle–particle particle–mesh (PPPM) method. A Velocity-Verlet integrator with a 1 fs time step was employed. Prior to production runs, each system underwent an initial energy minimization followed by a 5 ns equilibration in the NPT ensemble at 298.15 K using the Nosé-Hoover thermostat and barostat. Production simulations were subsequently performed in the NVT ensemble for 5 ns, and the resulting trajectories were used to analyze the solvation structures and coordination environments.

Geometry optimizations were carried out in CP2K using the Perdew–Burke–Ernzerhof (PBE) exchange-correlation functional, with dispersion interactions described by the Grimme D3 correction. Following a convergence test, we employed a plane-wave cut-off of 450 a.u. To perform the self-consistent field (SCF) calculations, we utilized the orbital transformation (OT) method^76^.

For all atomic species, we utilized the DZVP-MOLOPT-SR-GTH basis set in conjunction with GTH pseudopotentials^77^. The SCF convergence criterion was set to 1 × 10^−6^. For geometry optimizations, the maximum allowed displacement was set to 1 × 10^−3^, and the maximum allowed force was set to 4.5 × 10^−4^. All values are expressed in atomic units (a.u.).

The insertion positions for anions and cations were determined using a custom-developed code^78^. Initially, the Hartree potential of the EVBr_2_ system was calculated. Subsequently, anions and cations were randomly inserted into possible local maxima and minima of this potential, respectively. A constraint was applied to ensure that all inserted atoms maintained a minimum distance of 1.5 Å from other atoms in the system. For each stoichiometric composition, five distinct configurations were generated and optimized. The configuration exhibiting the lowest energy was selected for further analysis and discussion.

The structure and ELF were visualized using the VESTA software.

## Supplementary information

Supplementary Information

Description of Additional Supplementary Files

Supplementary Data 1-3

Transparent Peer Review file

## Source data

Source Data

## Data Availability

The source data generated in this study are provided in the Source Data file. Source data are provided with this paper.
