# Supplementary material for: Coordination electrochemistry taming reversible hypervalent bromine redox for energetic six-electron-transfer lithium-bromine battery
Source: Nat Commun. 2026 Jul 18;17:8799. doi: 10.1038/s41467-026-75860-6 (PMC13493775; doi:10.1038/s41467-026-75860-6)
Supplement: Supplementary file 1 [file 41467_2026_75860_MOESM1_ESM.pdf]

Supplementary Information

Coordination electrochemistry taming reversible hypervalent bromine redox for energetic six-electron-transfer lithium-bromine battery

Rumeng Feng[1], Wenyu Xu[1], Hongwei Wang[1], Binfen Wang[1], Jialin Li[1], Zelin Chang[1], Zhaodong Huang[2,3]*, Ze Chen[4]*, Xinliang Li[1,2]*

[1]School of Physics and Laboratory of Zhongyuan Light, Zhengzhou University, Zhengzhou 450052, China

[2]Department of Materials Science and Engineering, City University of Hong Kong, 83 Tat Chee Avenue, Hong Kong S.A.R., 999077, P. R. China

[3]Department of Chemical and Biological Engineering, Hong Kong University of Science and Technology, Clear Water Bay, Kowloon, Hong Kong 999077, China

[4]School of Interdisciplinary Studies, Lingnan University, 8 Castle Peak Road, Tuen Mun, Hong Kong, China

Correspondence authors: Prof. Li *E-mail: kezdhuang@ust.hk; zechen2@ln.edu.hk; xinlianli2-c@my.cityu.edu.hk

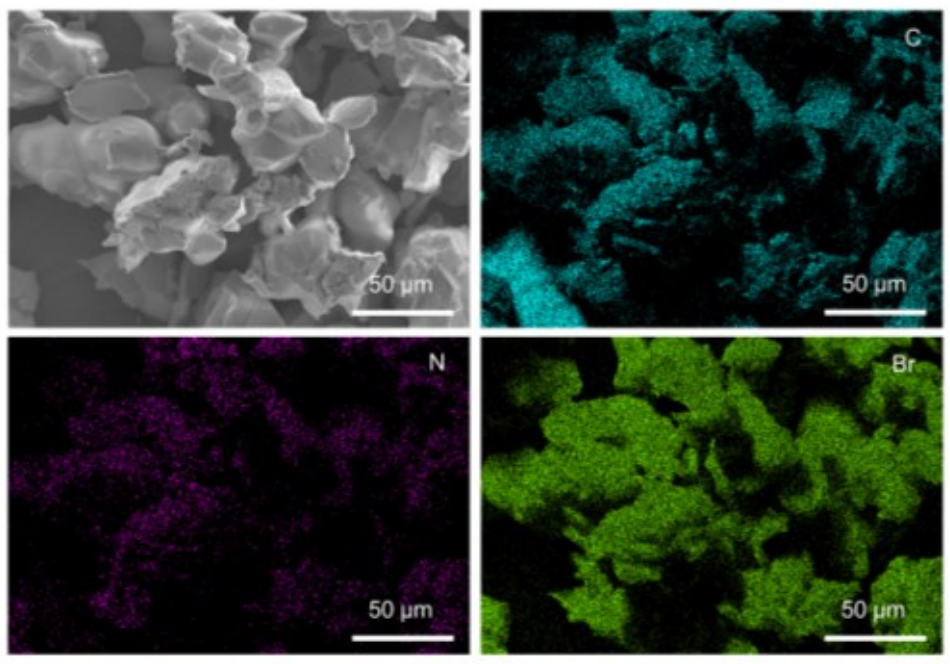

Supplementary Fig. 1 | Morphology and elemental distribution of ethyl viologen dibromide ($EVBr_2$). Scanning electron microscopy (SEM) image and Energy-dispersive X-ray spectroscopy (EDS) mapping of C, N, and Br in the $EVBr_2$ material.

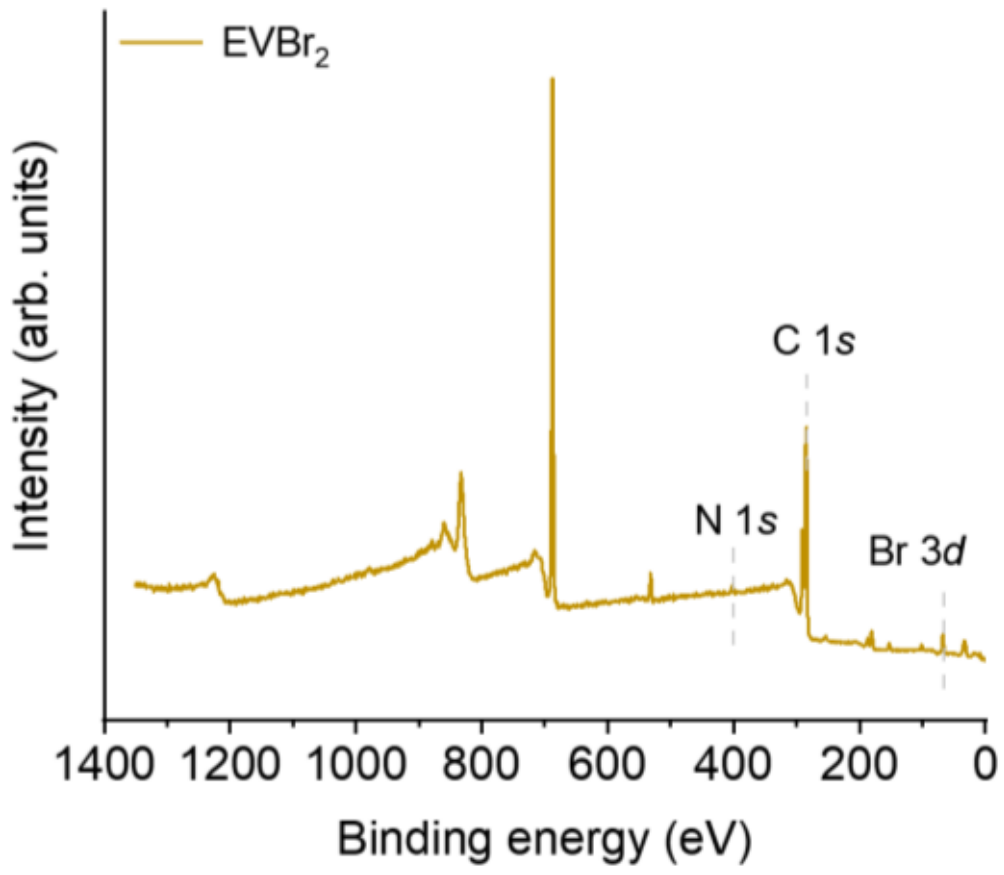

Supplementary Fig. 2 | X-ray photoelectron spectroscopy (XPS) results. The survey XPS spectrum of the $EVBr_2$ materials.

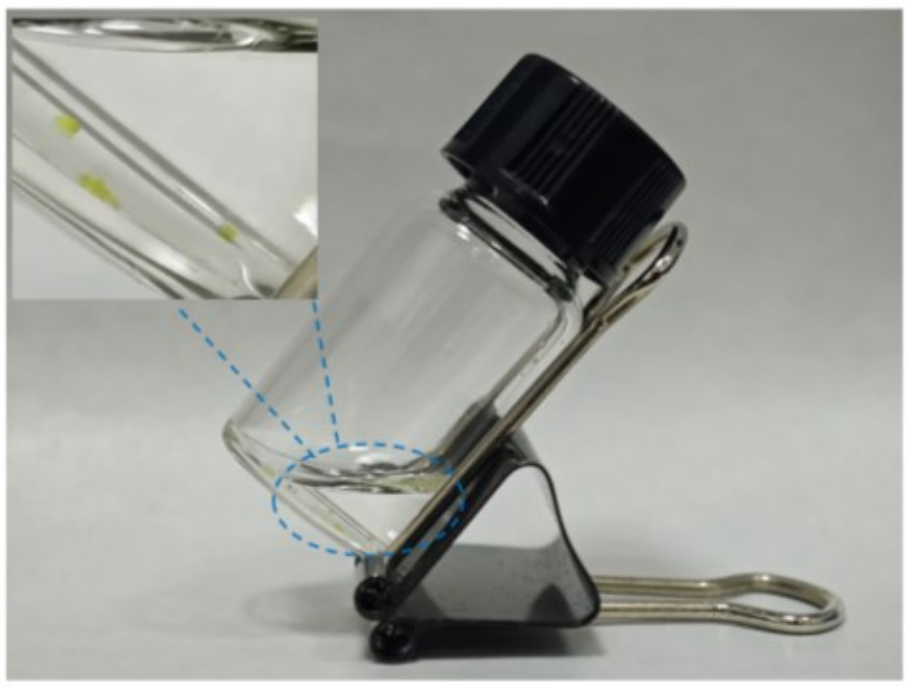

Supplementary Fig. 3 | $EVBr_2$ in DD (1.0 M LiTFSI in DME: DOL = 1:1 vol% with 1% $LiNO_3$) electrolyte. Photograph of $EVBr_2$ immersed in DD electrolyte.

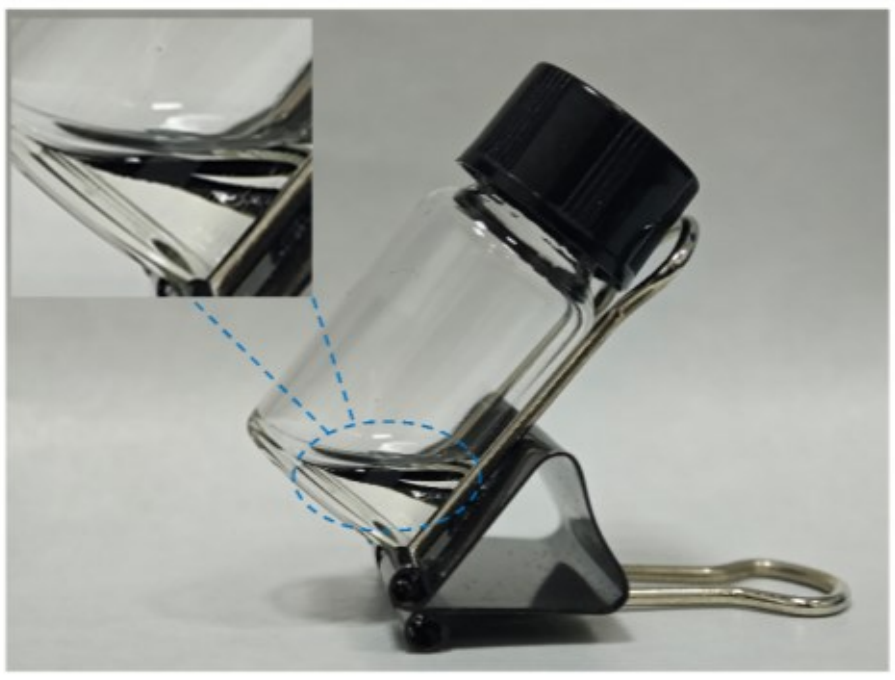

Supplementary Fig. 4 | $EVBr_2$ positive electrode in DD electrolyte. Photograph of $EVBr_2$ electrode immersed in DD electrolyte.

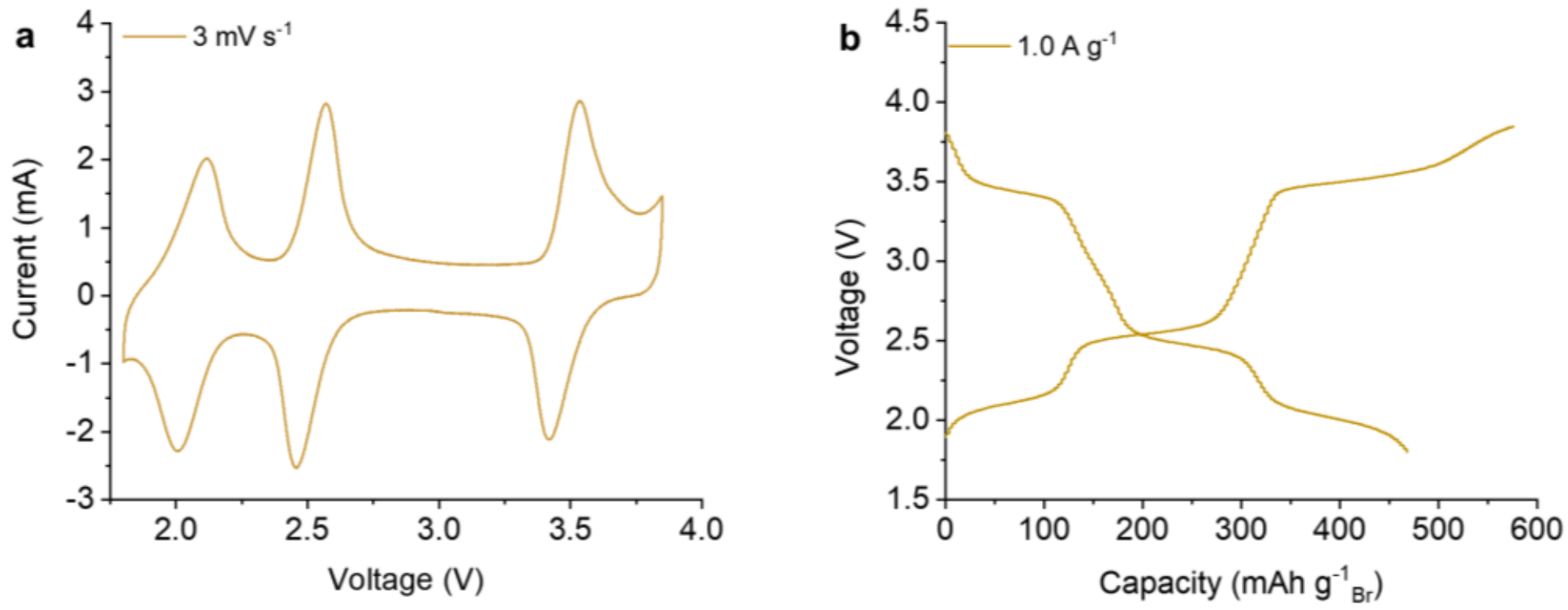

Supplementary Fig. 5 | Electrochemical behavior of Li||EVBr$_2$ battery in DD electrolyte. a Cyclic voltammetry (CV) curve of Li||EVBr$_2$ battery in DD electrolyte. **b** Galvanostatic charge/discharge (GCD) curve of Li||EVBr$_2$ battery in DD electrolyte. All batteries were tested at 25 ± 1 °C.

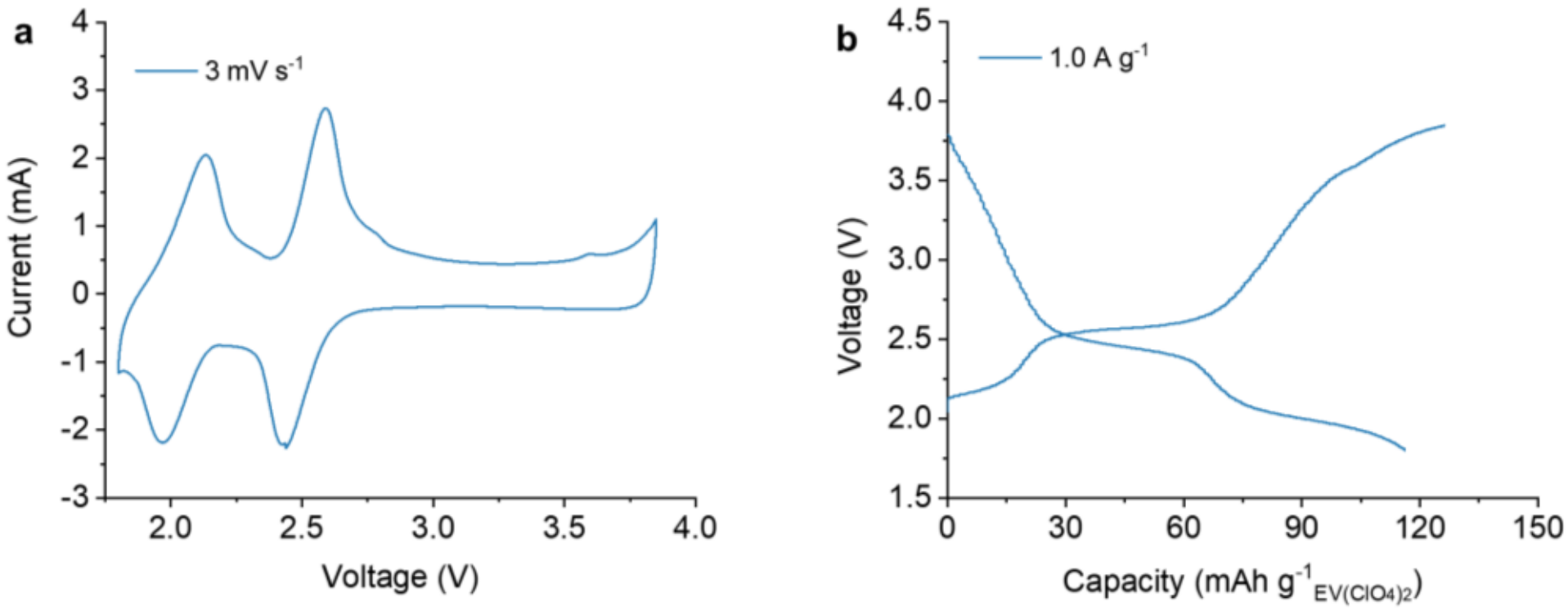

Supplementary Fig. 6 | Electrochemical behavior of Li||EV(ClO_4)$_2$ (ethyl viologen diperchlorate) battery in DD electrolyte. a CV curve of Li||EV(ClO_4)$_2$ battery in DD electrolyte. **b** GCD curve of Li||EV(ClO_4)$_2$ battery in DD electrolyte. All batteries were tested at 25 ± 1 °C.

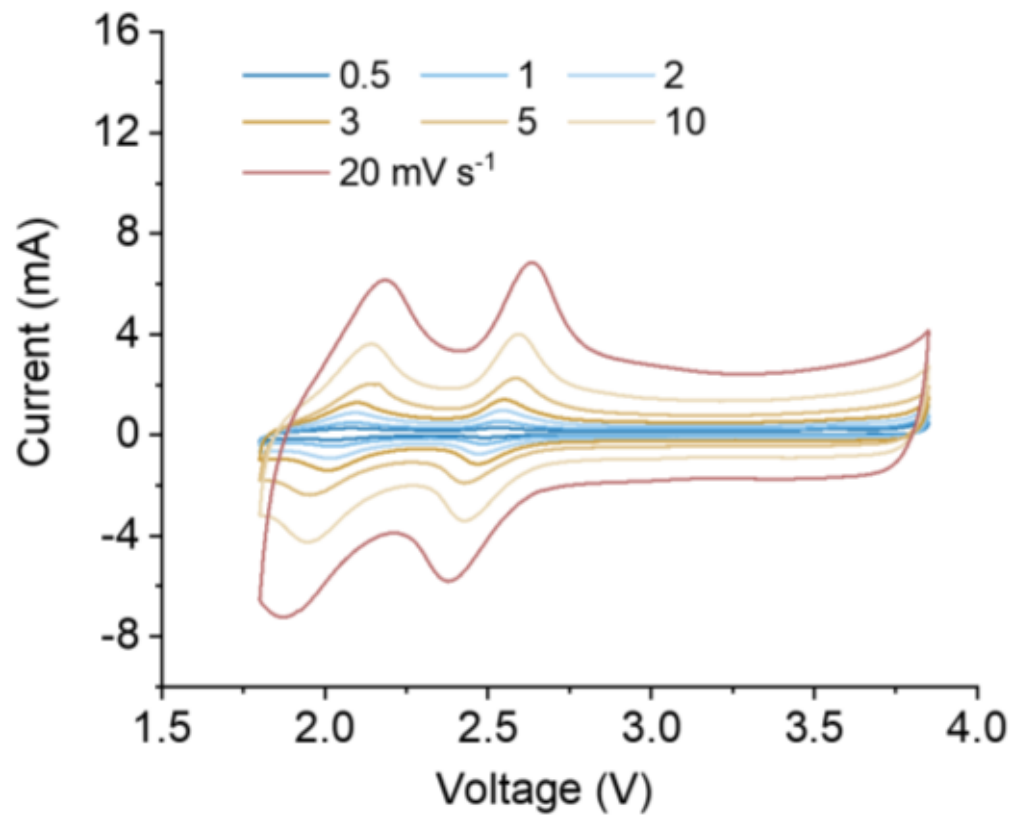

Supplementary Fig. 7 | Redox behavior of Li||EV(ClO_4)$_2$ battery. CV curves of the Li||EV(ClO_4)$_2$ battery in DD electrolyte. All batteries were tested at 25 ± 1 °C.

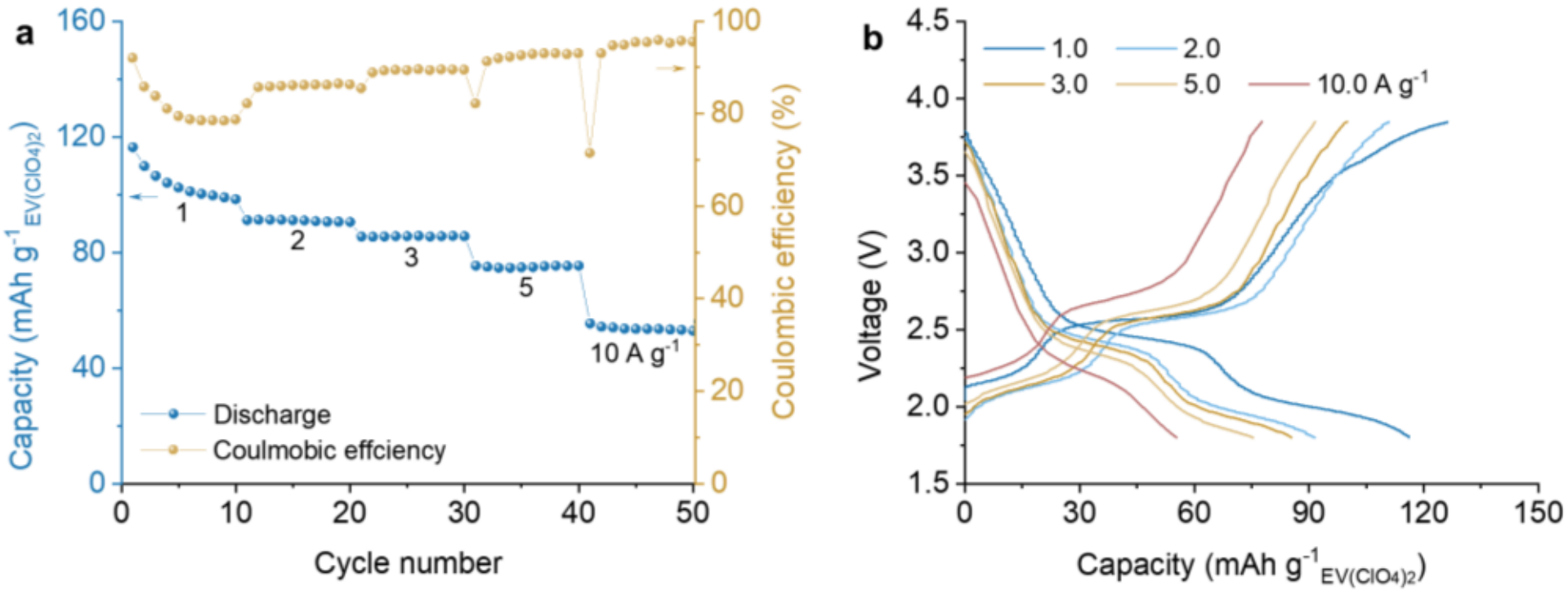

Supplementary Fig. 8 | Electrochemical performance of Li||EV(ClO_4)$_2$ battery in DD electrolyte. a Rate capability of the Li||EV(ClO_4)$_2$ battery in DD electrolyte. **b** GCD curves of the Li||EV(ClO_4)$_2$ battery in DD electrolyte. All batteries were tested at 25 ± 1 °C.

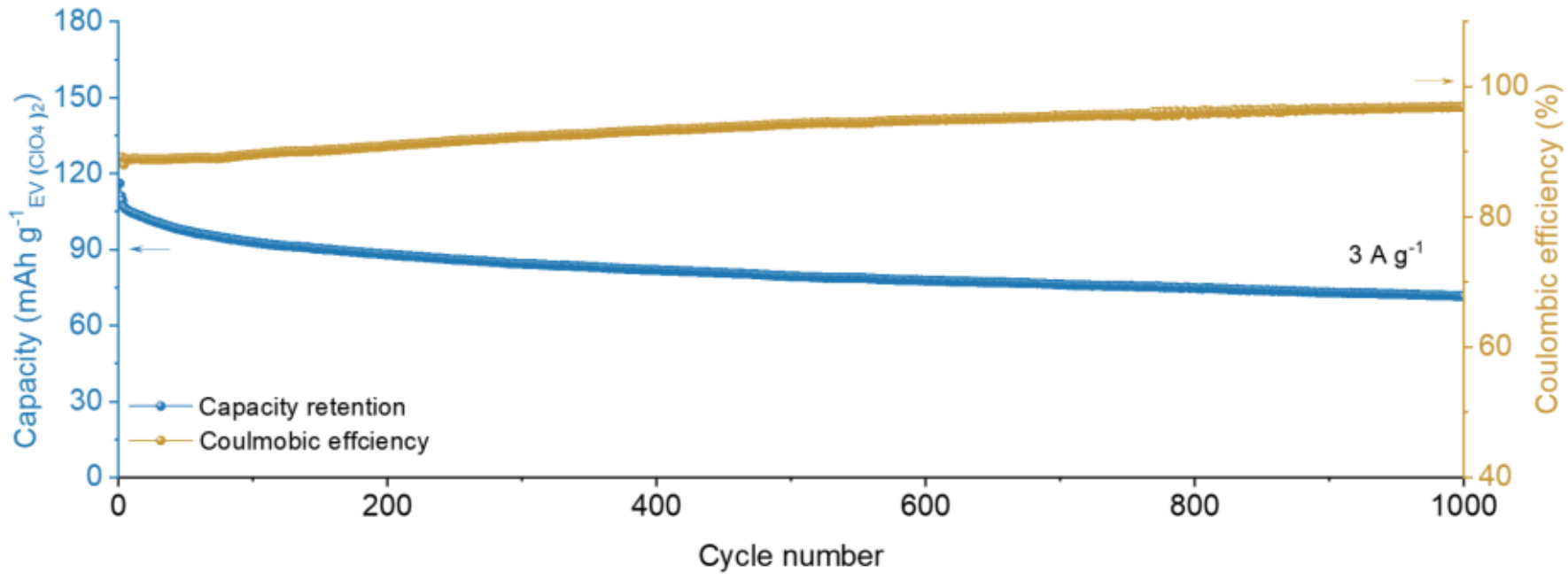

Supplementary Fig. 9 | Cycling stability of Li||EV(ClO_4)$_2$ battery in DD electrolyte. Prolonged cyclic performance of Li||EV(ClO_4)$_2$ battery in DD electrolyte at 3 A g^{-1}. All batteries were tested at 25 ± 1 °C.

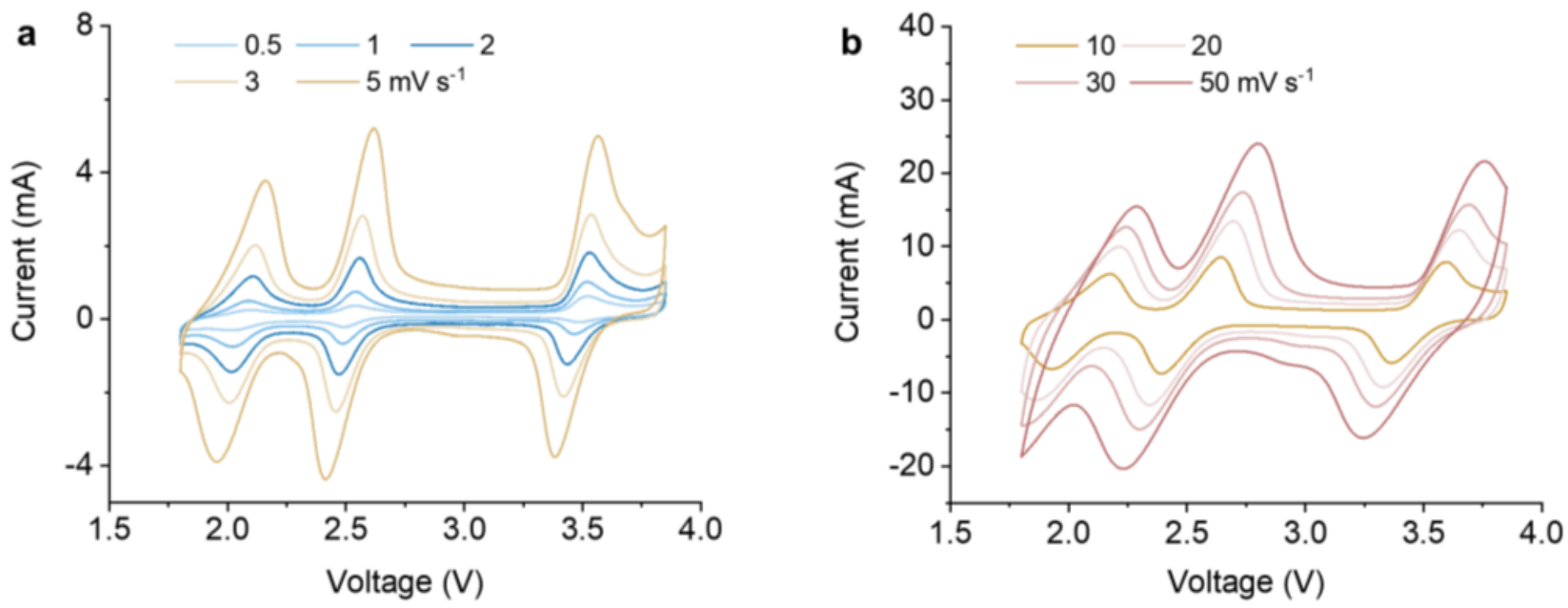

Supplementary Fig. 10 | Redox behavior of Li||EVBr$_2$ battery in DD electrolyte. a CV curves of Li||EVBr$_2$ battery in DD electrolyte ranging from 0.5 to 5 mV s^{-1}. **b** CV curves of Li||EVBr$_2$ battery in DD electrolyte ranging from 10 to 50 mV s^{-1}. All batteries were tested at 25 ± 1 °C.

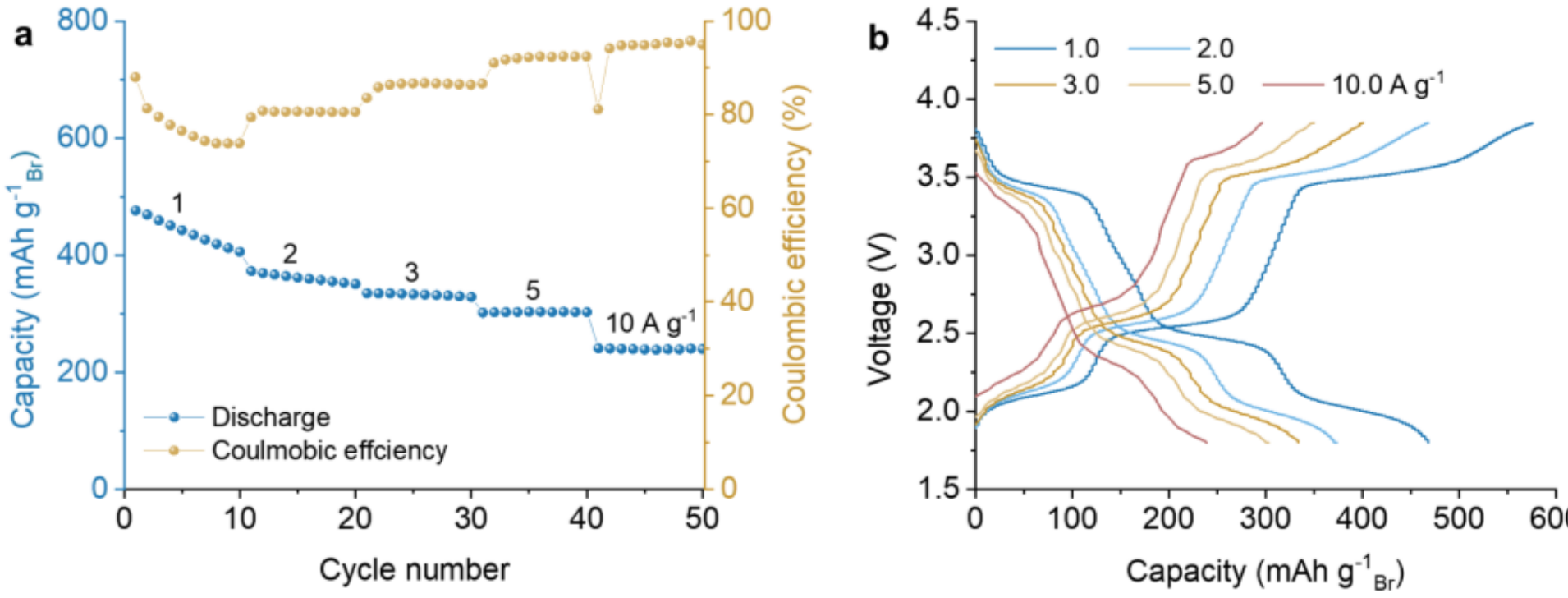

Supplementary Fig. 11 | Electrochemical performance of Li||$EVBr_2$ battery in DD electrolyte. a Rate capability of the Li||$EVBr_2$ battery in DD electrolyte. **b** GCD curves of the Li||$EVBr_2$ battery in DD electrolyte. All batteries were tested at 25 ± 1 °C.

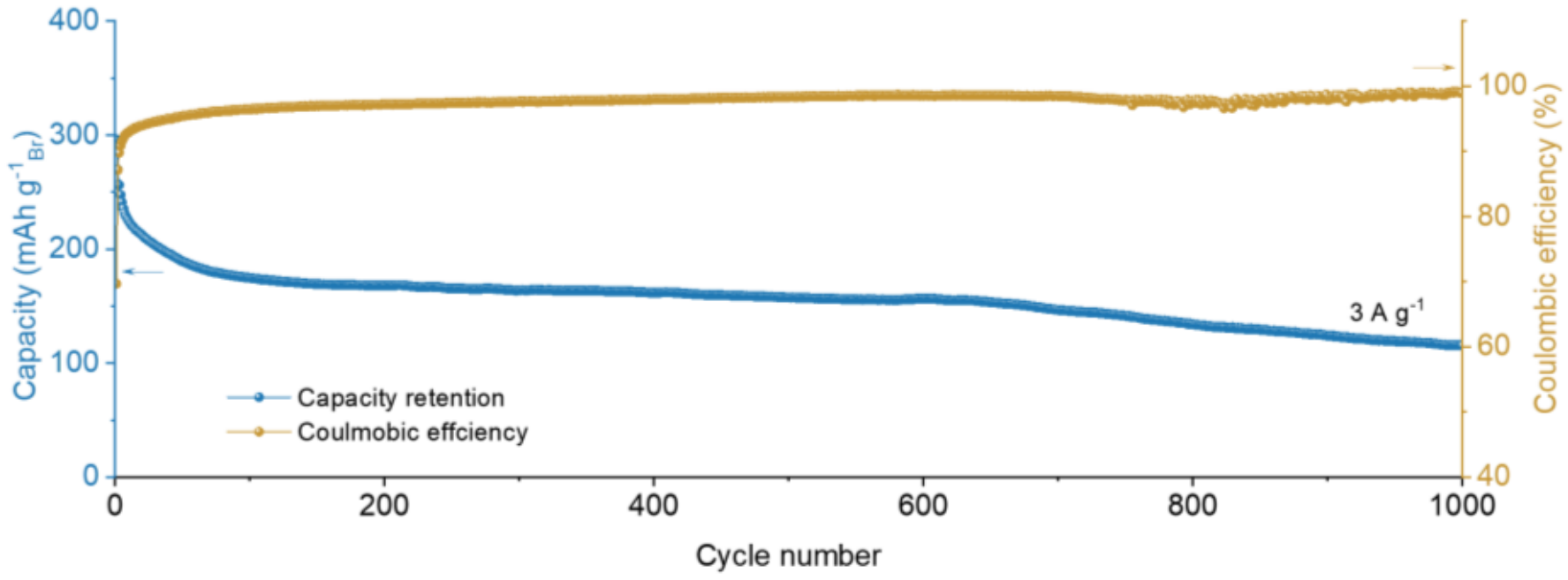

Supplementary Fig. 12 | Cycling stability of Li||EVBr$_2$ battery in DD electrolyte. Long-term cycling performance of Li||EVBr$_2$ battery in DD electrolyte at 3 A g^{-1}. All batteries were tested at 25 ± 1 °C.

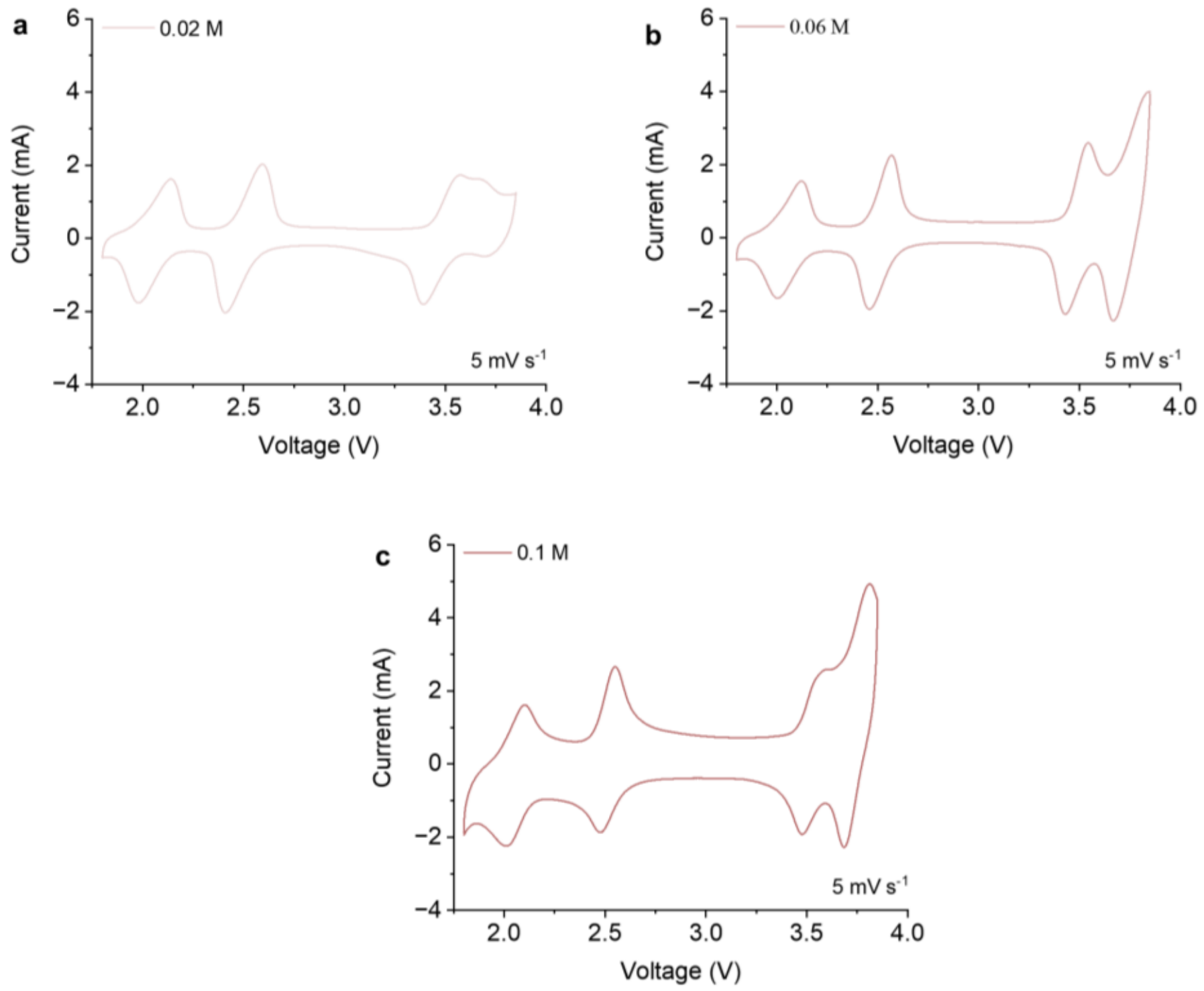

Supplementary Fig. 13 | Effect of lithium chloride (LiCl) concentration on the redox behavior of Li||$EVBr_2$ battery. CV curves of Li||$EVBr_2$ battery in DD electrolyte with different LiCl concentrations. **a** 0.02 M. **b** 0.06 M. **c** 0.1 M. All batteries were tested at 25 ± 1 °C.

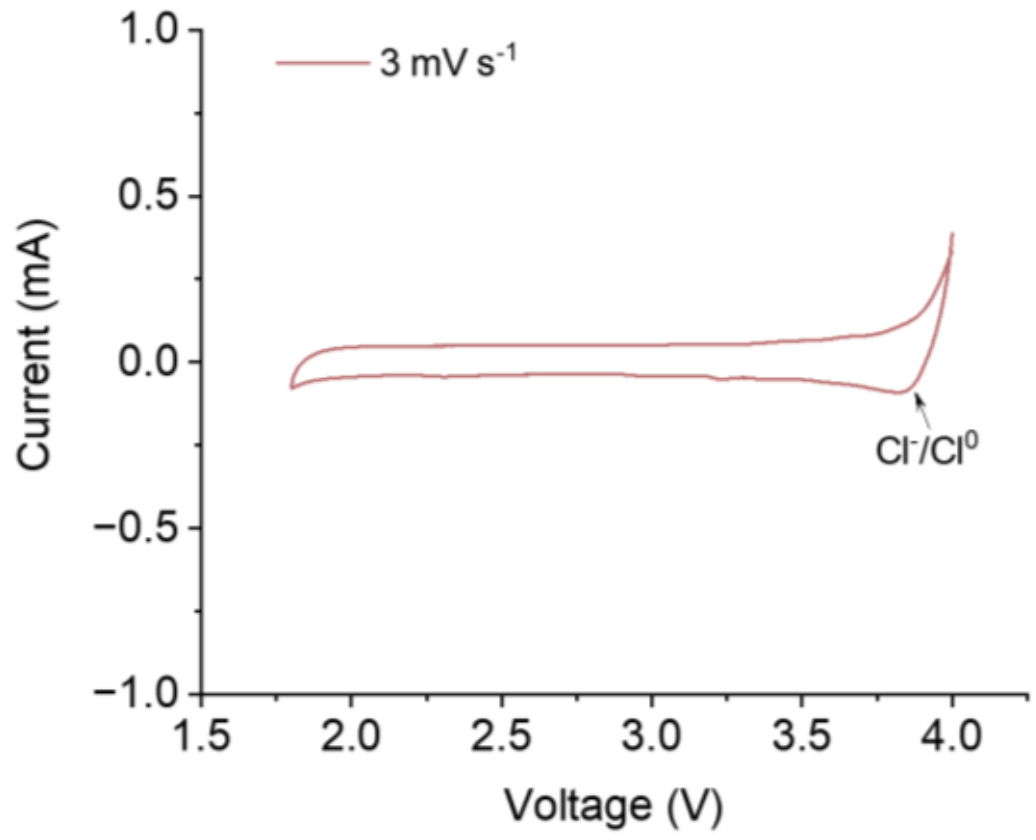

Supplementary Fig. 14 | Redox behavior of Li||KB (Ketjen black) battery. CV curve of Li||KB battery in DDC (0.1 M LiCl in DD) electrolyte. All batteries were tested at 25 ± 1 °C.

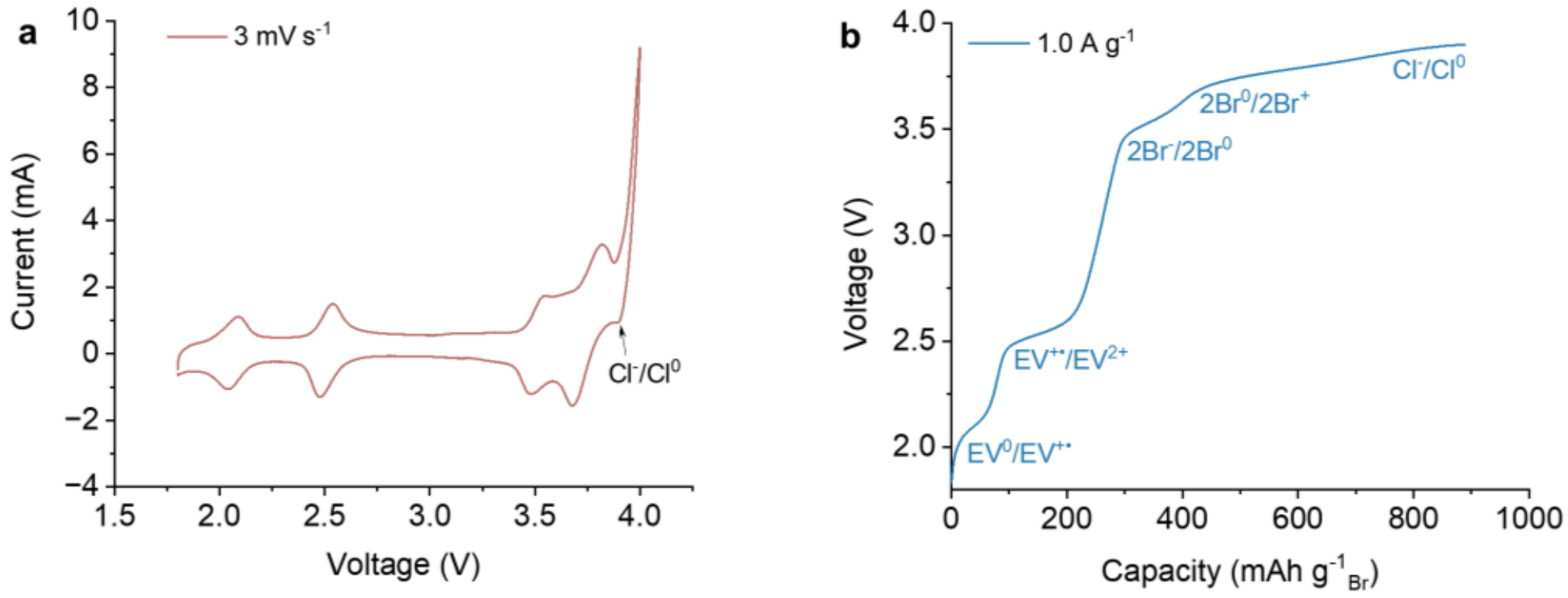

Supplementary Fig. 15 | Electrochemical behavior of Li||EVBr_2 battery in DDC electrolyte under wide potential window. a CV curve of Li|| EVBr_2 battery in DDC electrolyte. **b** Charge curve of Li||EVBr_2 battery in DDC electrolyte. All batteries were tested at 25 ± 1 °C.

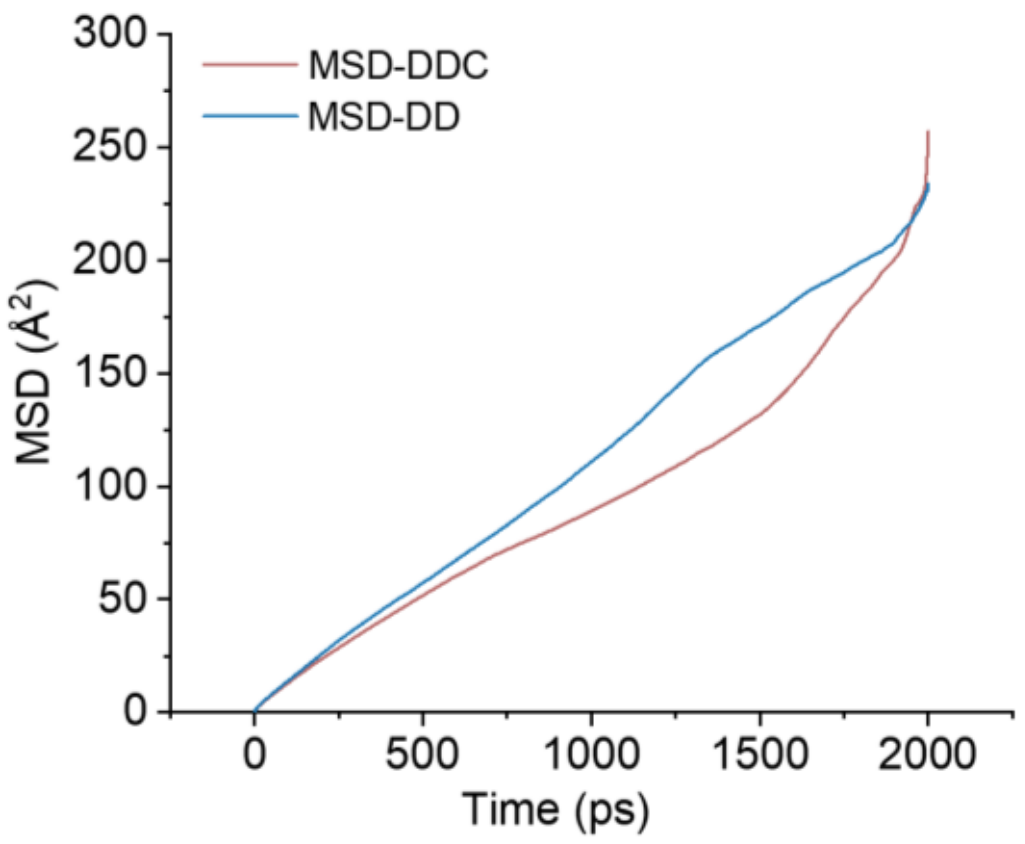

Supplementary Fig. 16 | Diffusion behavior of the electrolytes. Mean square displacement (MSD) curves of the electrolytes.

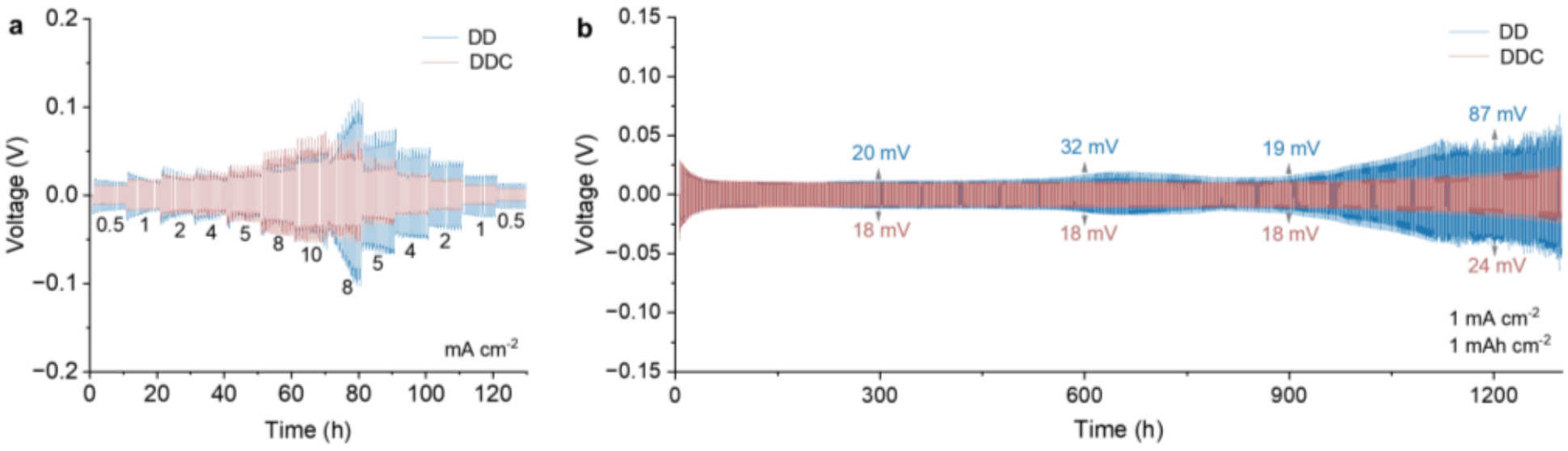

Supplementary Fig. 17 | Lithium plating/stripping stability in DD and DDC electrolytes. a Rate capability of a symmetric Li||Li battery with DD and DDC electrolytes. **b** Long-term cycling performance of a symmetric Li||Li battery with DD and DDC electrolyte at 1 mA cm^{-2} and 1 mAh cm^{-2}. All batteries were tested at 25 ± 1 °C.

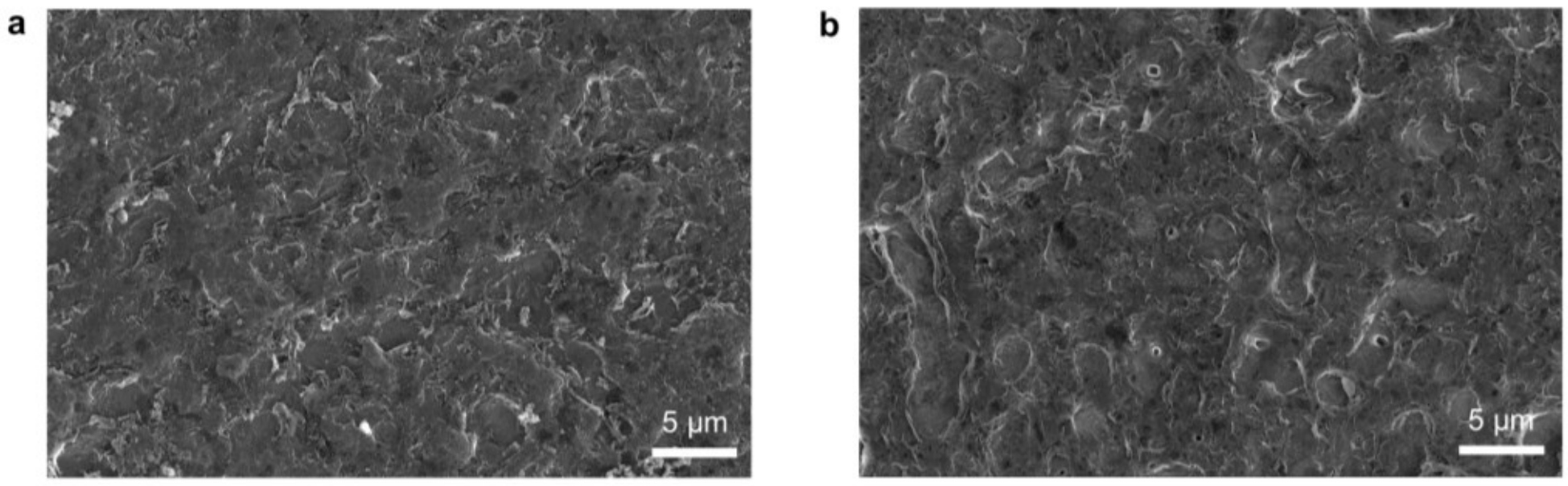

Supplementary Fig. 18 | Morphology evolution of lithium metal during cycling. a SEM image of lithium metal in the charged to 3.85 V. **b** SEM image of lithium metal in the discharged to 1.8 V. The electrodes were obtained from batteries that were first cycled at 1 A g^{-1} for 10 cycles, then charged or discharged to the indicated states of charge (SOCs), after which the batteries were disassembled, and the electrodes were transferred under vacuum for measurements. All electrochemical and characterization tests were conducted at 25 ± 1 °C.

a

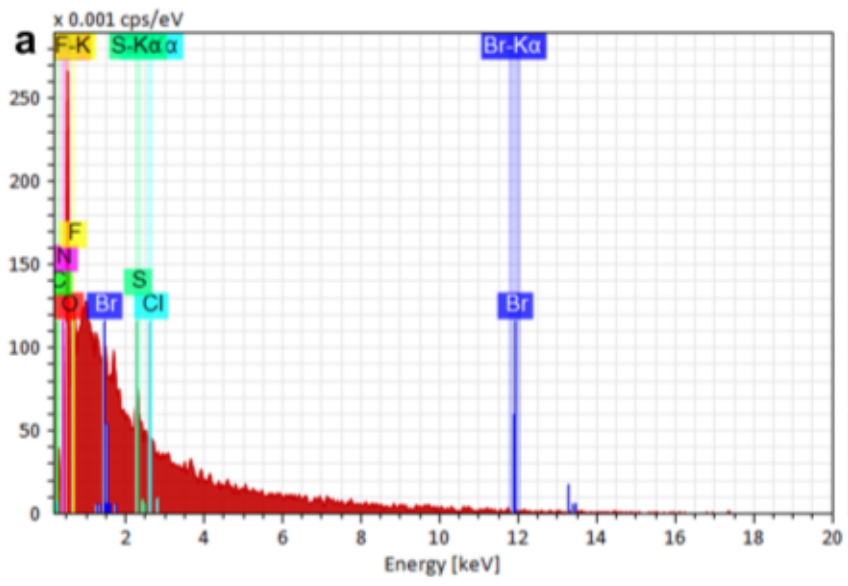

Element	Mass ratio (wt%)
C	26.06
N	0.00
O	69.92
F	0.00
S	3.20
Cl	0.82
Br	0.00
Total	100.000

b

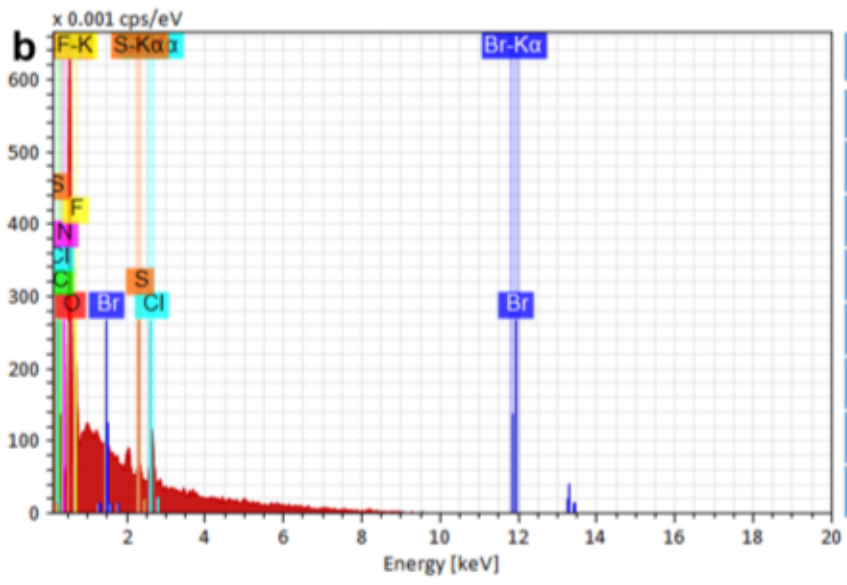

Element	Mass ratio (wt%)
C	19.26
N	0.00
O	51.47
F	25.42
S	0.72
Cl	3.13
Br	0.00
Total	100.000

Supplementary Fig. 19 | Elemental distribution of lithium metal during cycling. a EDS analysis of lithium metal in the charged to 3.85 V. **b** EDS analysis of lithium metal in the discharged to 1.8 V. The electrodes were obtained from batteries that were first cycled at 1 A g^{-1} for 10 cycles, then charged or discharged to the indicated SOCs, after which the batteries were disassembled, and the electrodes were transferred under vacuum for measurements. All electrochemical and characterization tests were conducted at 25 ± 1 °C.

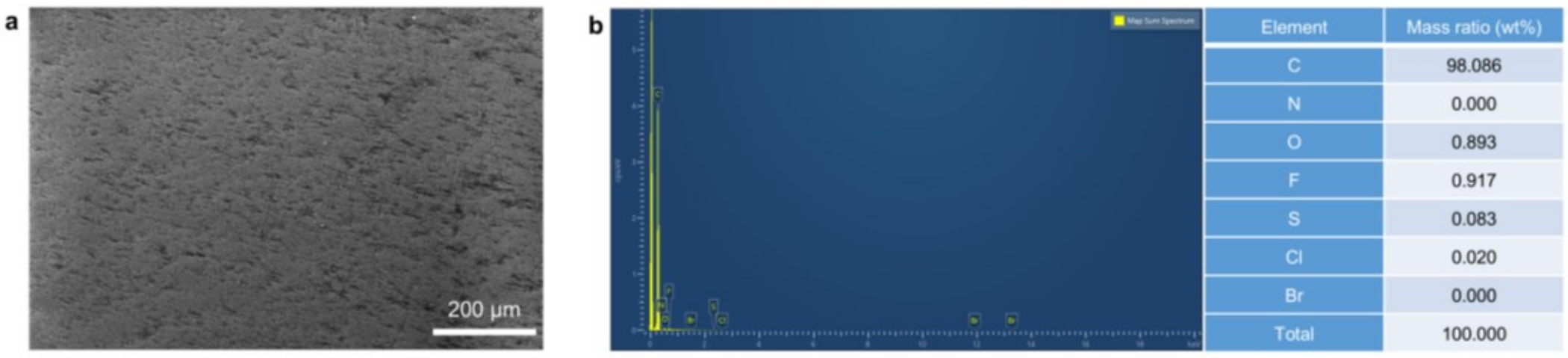

Element	Mass ratio (wt%)
C	98.086
N	0.000
O	0.893
F	0.917
S	0.083
Cl	0.020
Br	0.000
Total	100.000

Supplementary Fig. 20 | Separator morphology and elemental distribution after cycling. a SEM image of the separator after cycling. **b** EDS mapping of the separator after cycling. The separator was obtained from batteries that were first cycled at 1 A g^{-1} for 10 cycles and then disassembled. All electrochemical and characterization tests were conducted at 25 ± 1 °C.

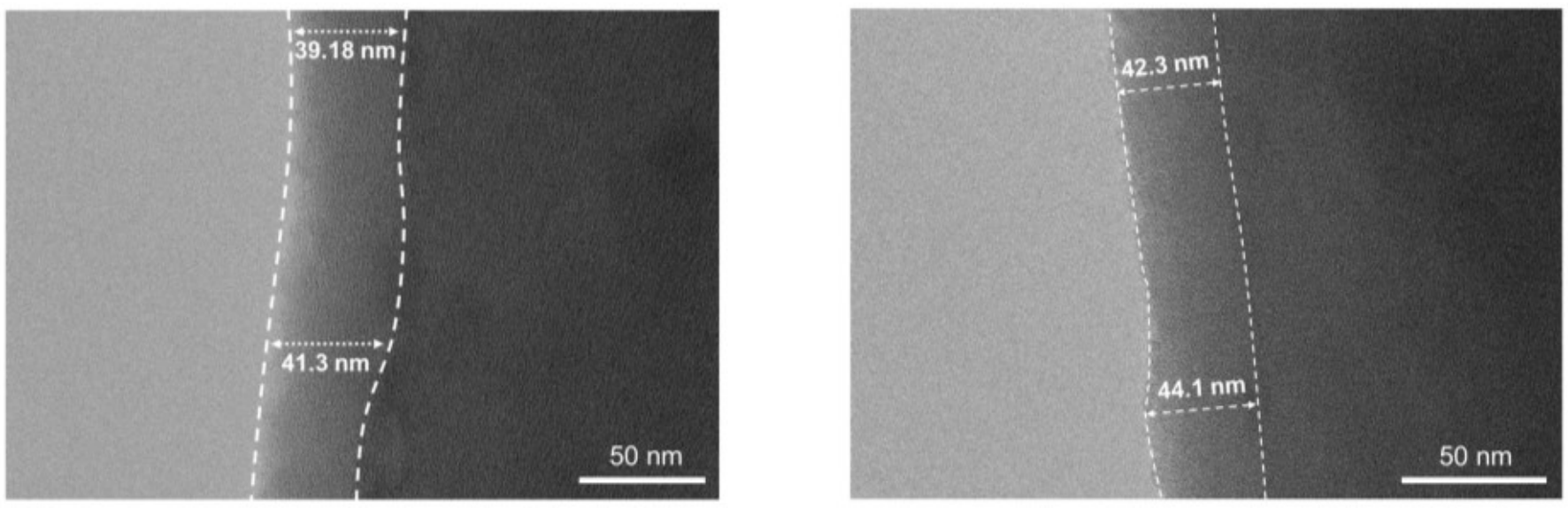

Supplementary Fig. 21 | Microstructure of the solid-electrolyte interphase (SEI) layer. Transmission electron microscopy (TEM) images of the SEI layer. The electrodes were obtained from batteries that were first cycled at 1 A g^{-1} for 10 cycles, then discharged to 1.8 V, after which the batteries were disassembled, and the electrodes were transferred under vacuum for measurements. All electrochemical and characterization tests were conducted at 25 ± 1 °C.

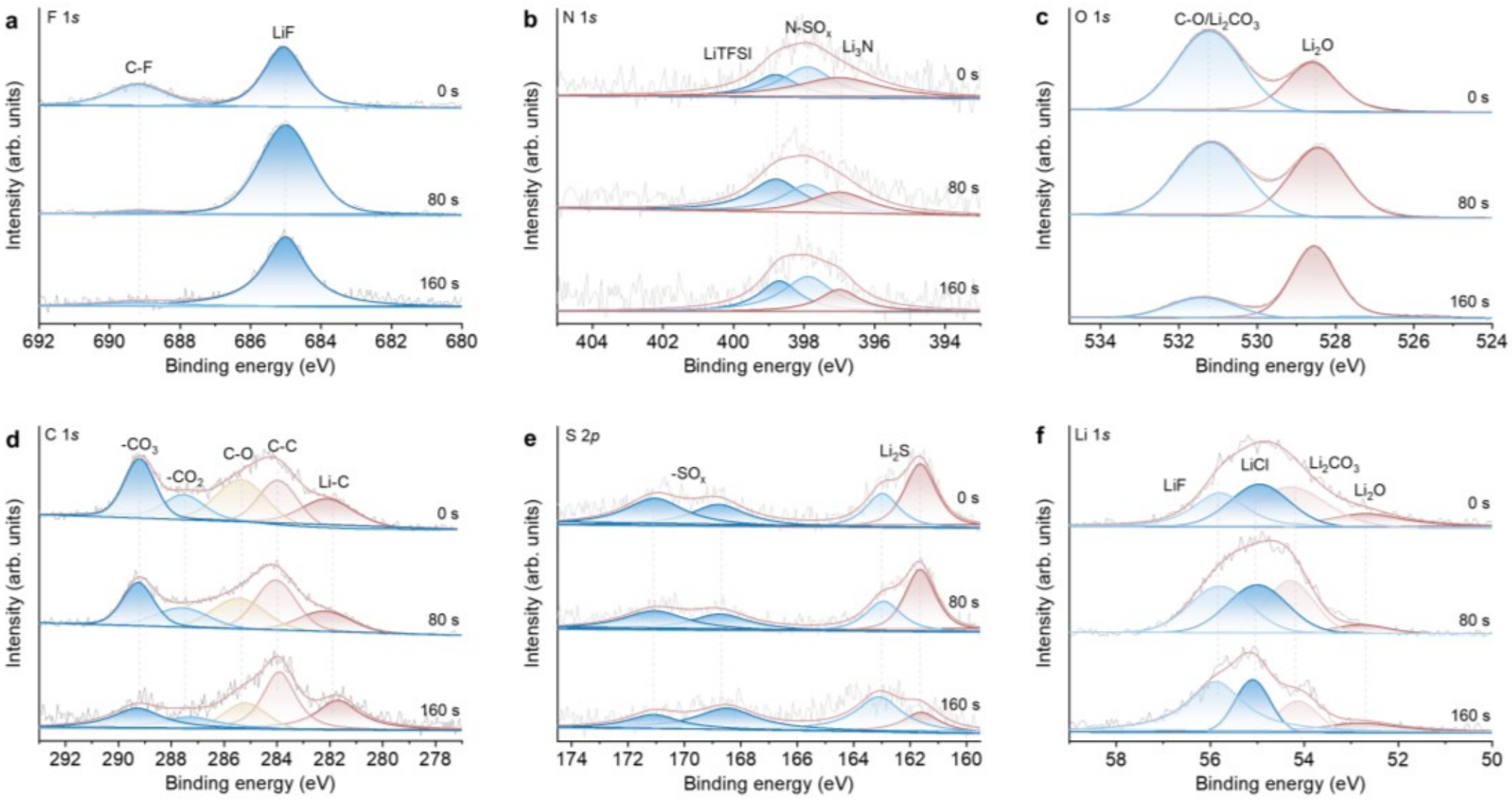

Supplementary Fig. 22 | Chemical states of the SEI after cycling. High-resolution XPS spectra of the SEI after cycling. **a** F 1*s*. **b** N 1*s*. **c** O 1*s*. **d** C 1*s*. **e** S 2*p*. **f** Li 1*s*. The electrodes were obtained from batteries that were first cycled at 1 A g^{-1} for 10 cycles, then discharged to 1.8 V, after which the batteries were disassembled, and the electrodes were transferred under vacuum for measurements. All electrochemical and characterization tests were conducted at 25 ± 1 °C.

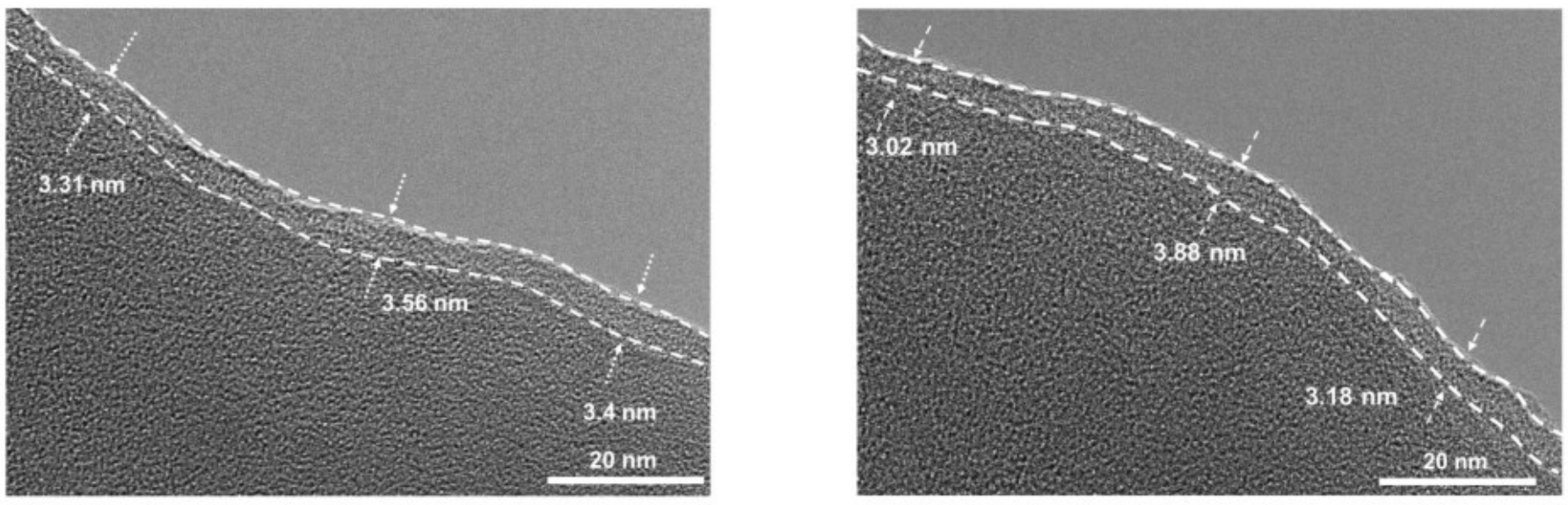

Supplementary Fig. 23 | Microstructure of the cathode-electrolyte interphase (CEI) layer. TEM images of the CEI layer. The electrodes were obtained from batteries that were first cycled at 1 A g^{-1} for 10 cycles, then charged to 3.85 V, after which the batteries were disassembled. All electrochemical and characterization tests were conducted at 25 ± 1 °C.

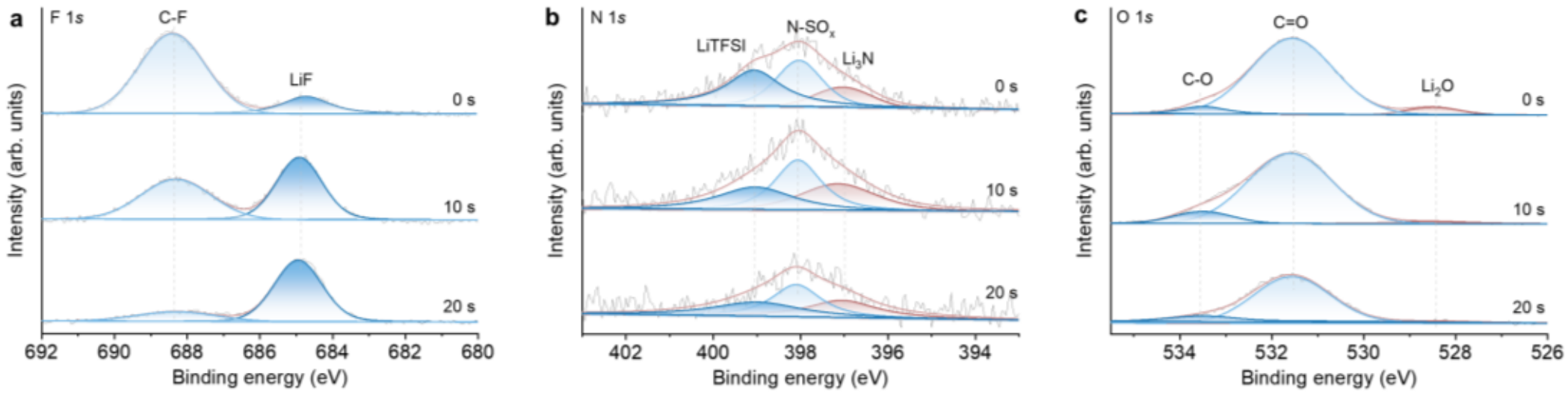

Supplementary Fig. 24 | Chemical states of the CEI after cycling. High-resolution XPS spectra of the CEI after cycling. **a** F 1*s*. **b** N 1*s*. **c** O 1*s*. The electrodes were obtained from batteries that were first cycled at 1 A g^{-1} for 10 cycles, then charged to 3.85 V, after which the batteries were disassembled. All electrochemical and characterization tests were conducted at 25 ± 1 °C.

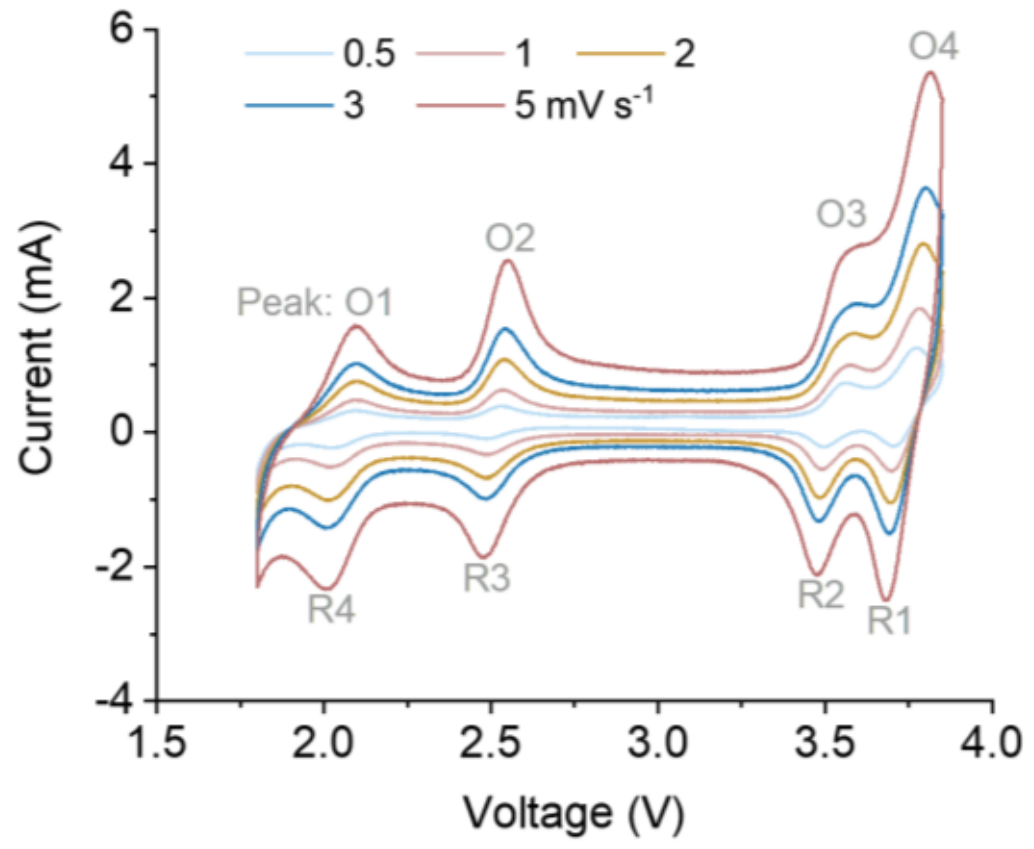

Supplementary Fig. 25 | Redox behavior of Li||$EVBr_2$ battery in DDC electrolyte. CV curves of Li||$EVBr_2$ battery in DDC electrolyte ranging from 0.5 to 5 mV s^{-1}. All batteries were tested at 25 ± 1 °C.

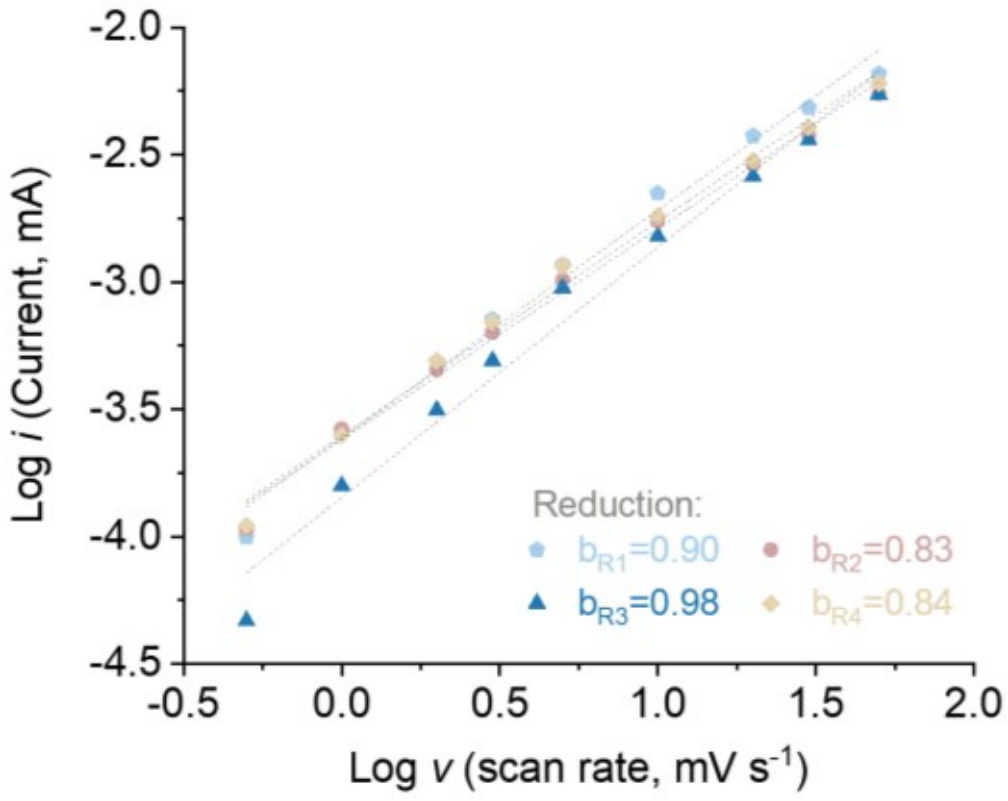

Supplementary Fig. 26 | Kinetic analysis of the reduction process. The calculated *b* values of reduction peaks.

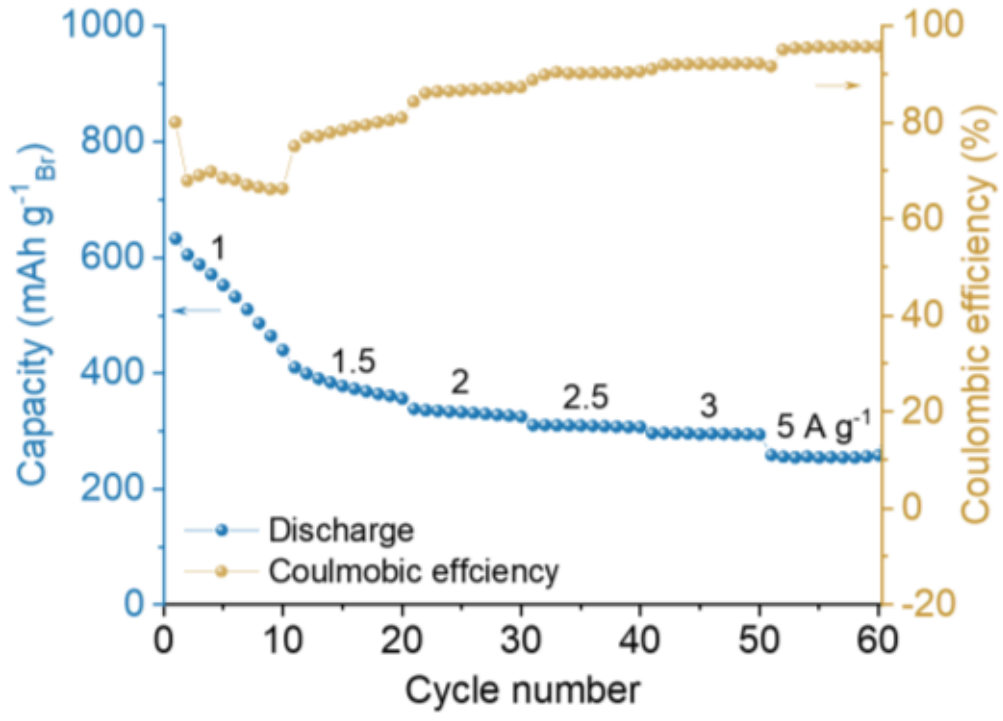

Supplementary Fig. 27 | Rate performance of Li||$EVBr_2$ battery. The rate capability of Li||$EVBr_2$ battery in DDC electrolyte. All batteries were tested at 25 ± 1 °C.

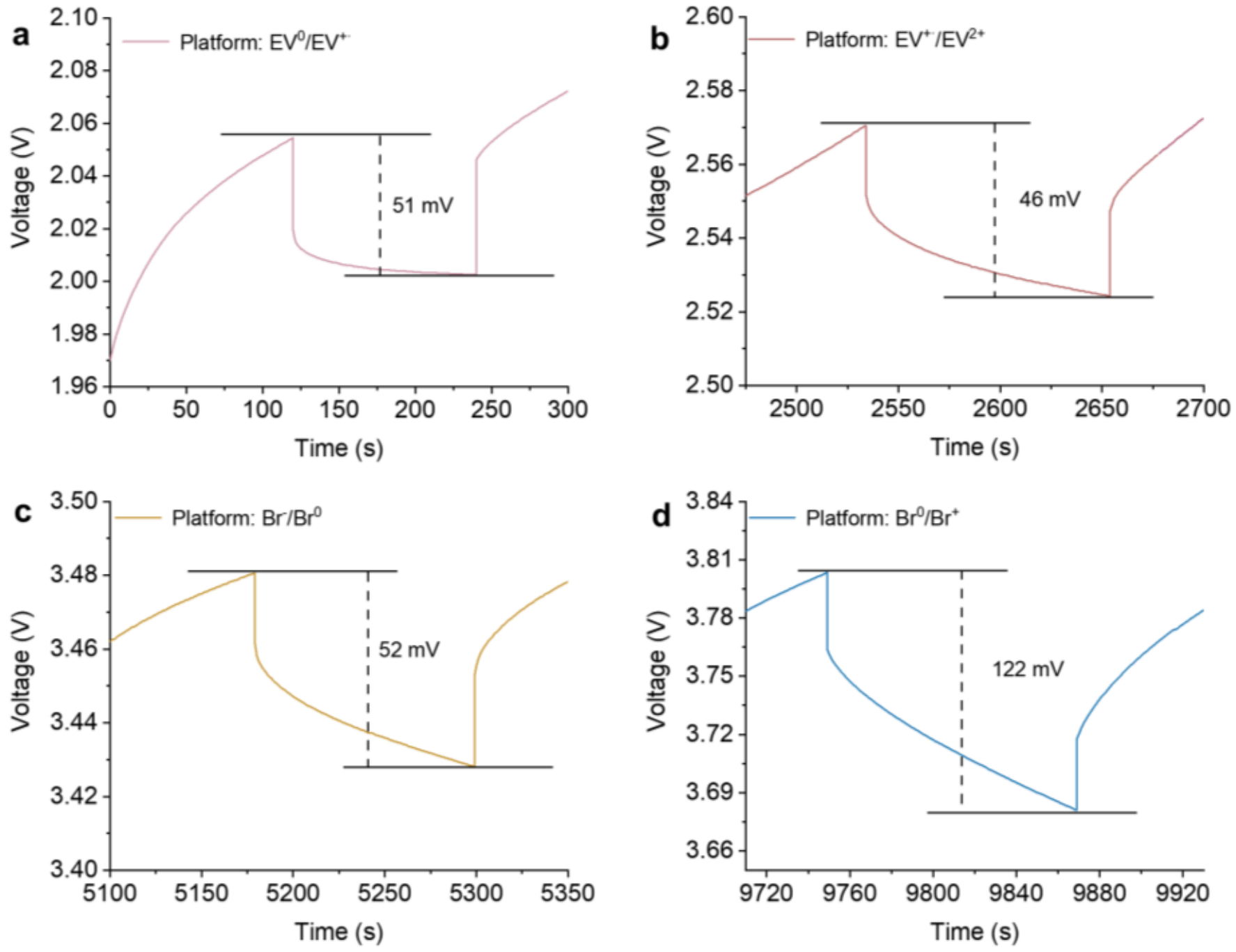

Supplementary Fig. 28 | Reaction polarization at different redox stages. Magnified galvanostatic intermittent titration technique (GITT) curves and measured overpotentials for different redox stages of Li||EVBr$_2$ battery. **a** GITT curve at the EV^0/EV$^{+\bullet}$ redox plateau, showing the voltage response during the initial stage of ethyl viologen oxidation. **b** GITT curve at the EV$^{+\bullet}$/EV^{2+} redox plateau, corresponding to the second electron-transfer step of the viologen moiety. **c** GITT curve at the Br$^-$/Br^0 redox plateau, representing the two-electron conversion of bromide to elemental bromine. **d** GITT curve at the Br^0/Br$^+$ redox plateau, corresponding to the high-valence bromine conversion. All batteries were tested at 25 ± 1 °C.

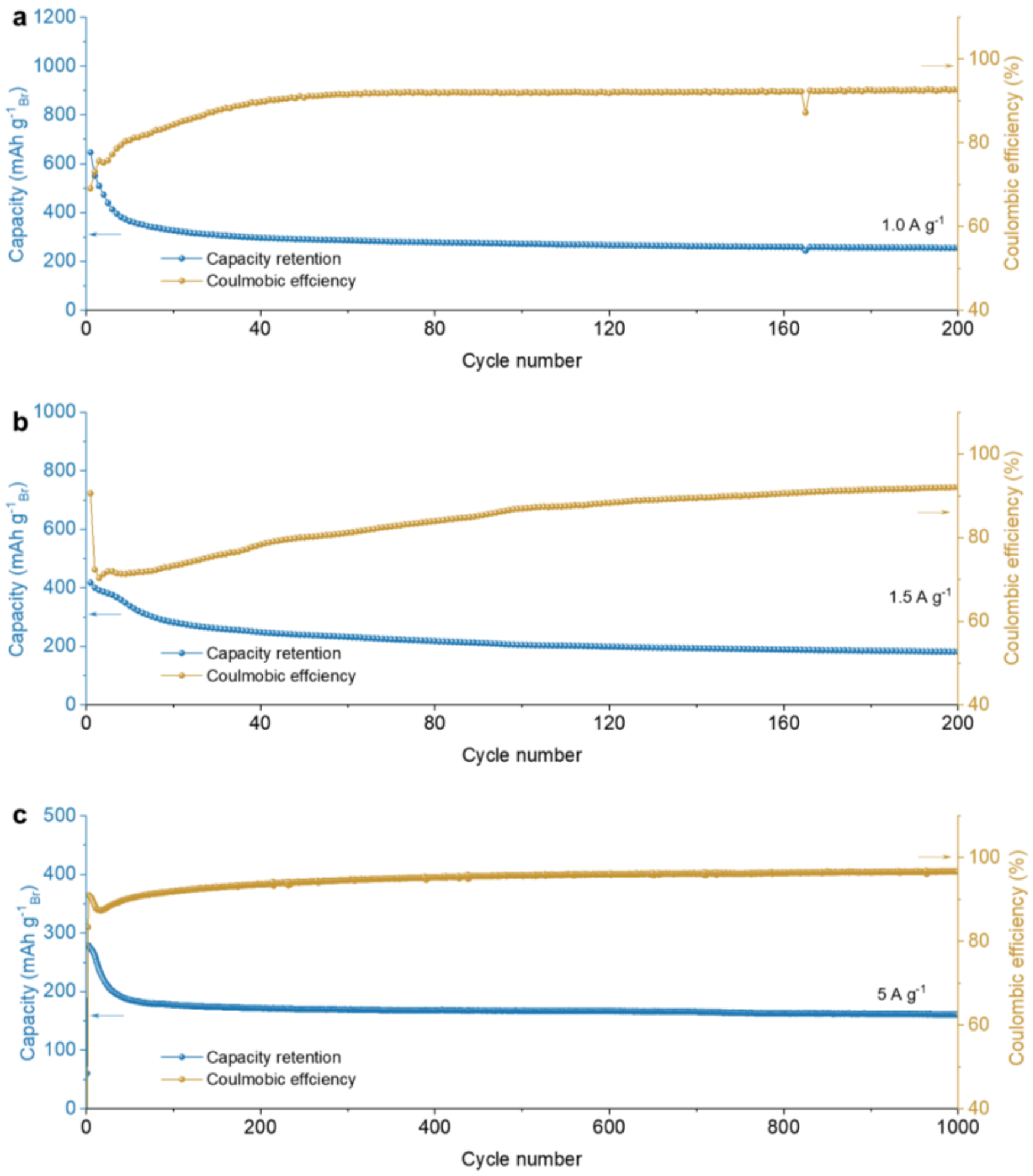

Supplementary Fig. 29 | Cycling stability. Long-term cycling performance of the Li||EVBr$_2$ battery in DDC electrolyte at different specific currents. **a** 1.0 A g^{-1}. **b** 1.5 A g^{-1}. **c** 5.0 A g^{-1}. All batteries were tested at 25 ± 1 °C.

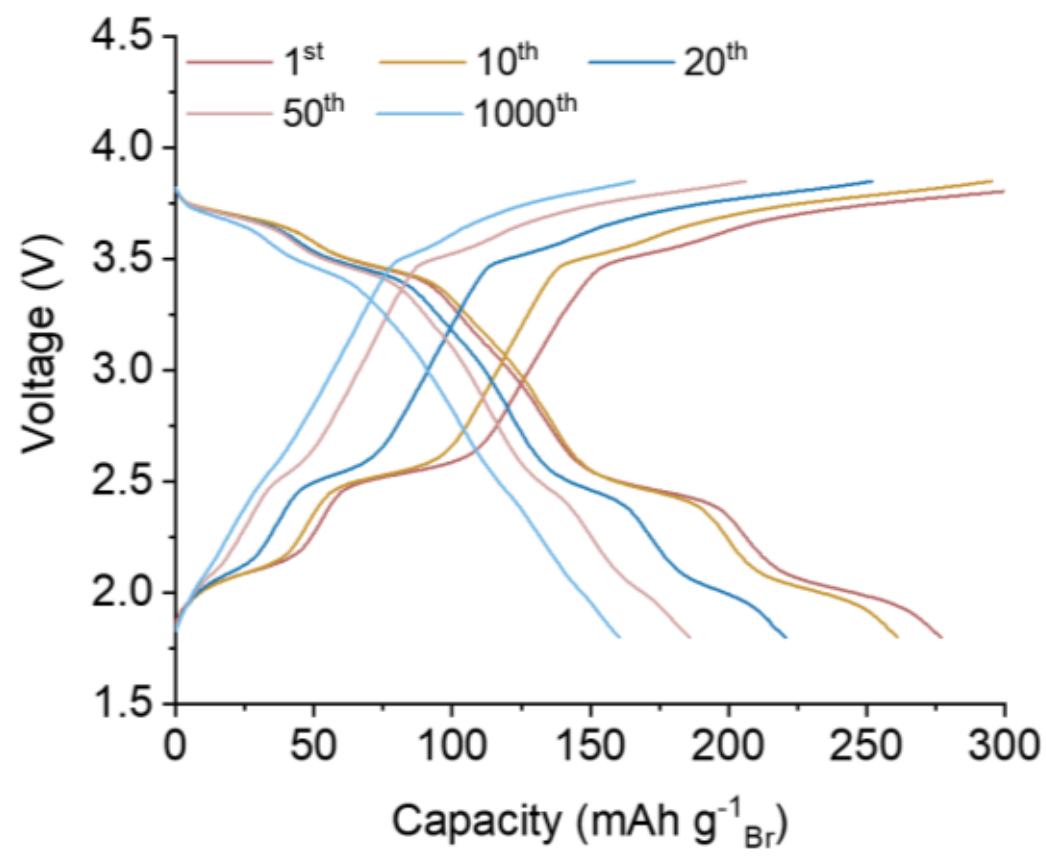

Supplementary Fig. 30 | GCD curves during cycling. GCD curves of Li||$EVBr_2$ battery in DDC electrolyte at 5.0 A g^{-1}. All batteries were tested at 25 ± 1 °C.

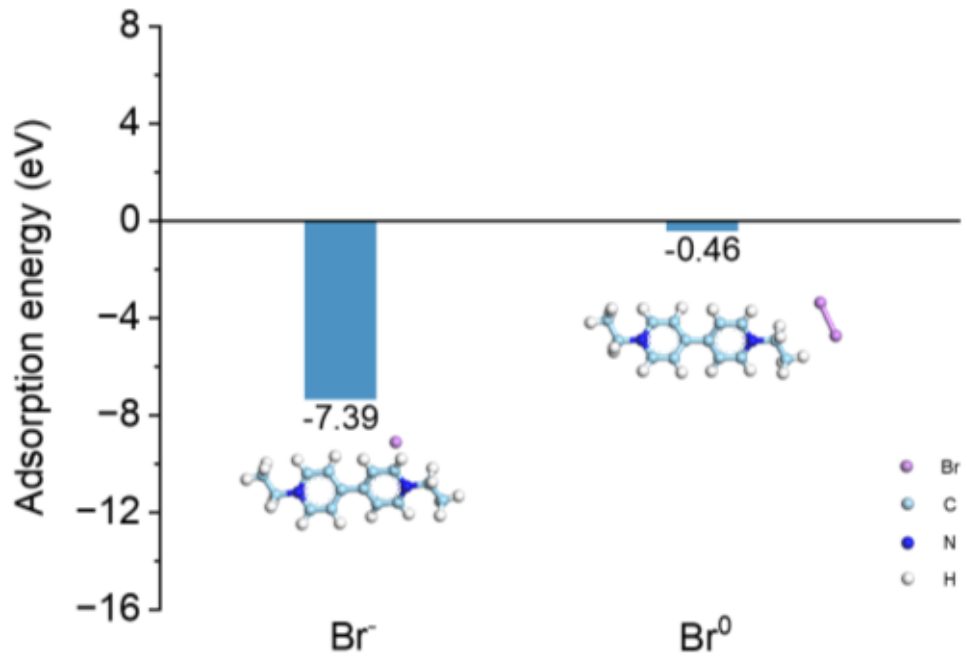

Supplementary Fig. 31 | Adsorption of bromine species on EV^{2+}. Adsorption energies of EV^{2+} for Br^- and Br^0.

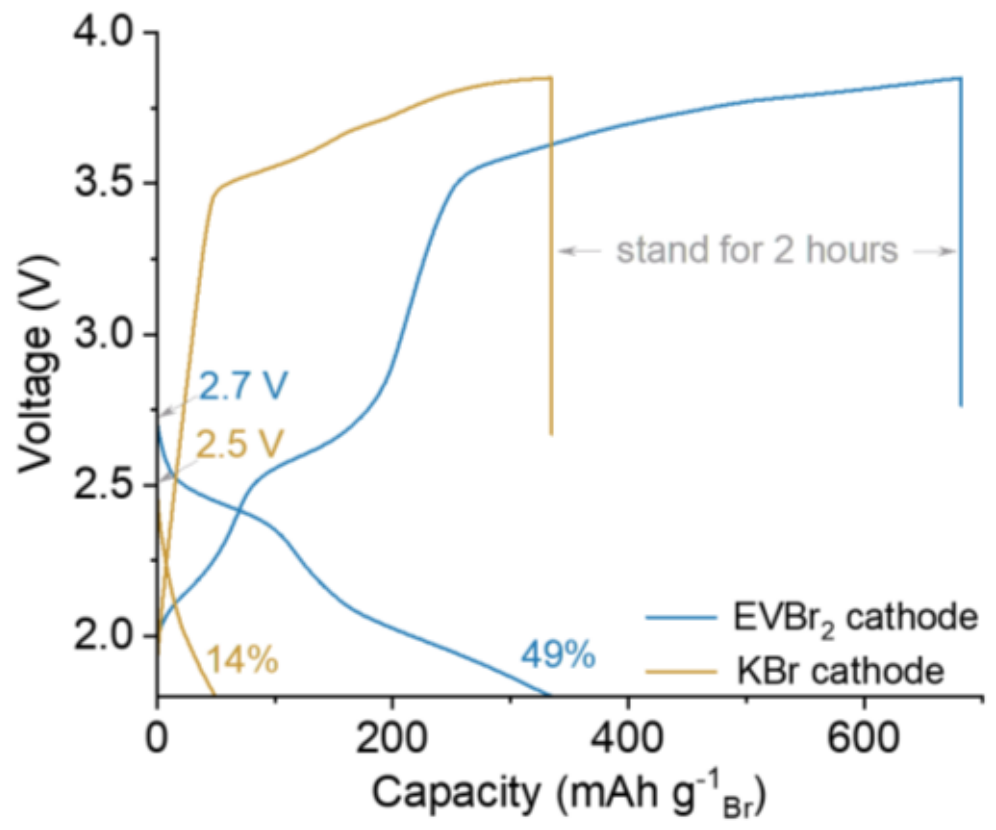

Supplementary Fig. 32 | Charge retention capability of Li||$EVBr_2$ and Li||KBr (potassium bromide) batteries. Comparison of the self-discharge behaviors for Li||$EVBr_2$ and Li||KBr batteries after charging at 1 A g^{-1}. All batteries were tested at 25 ± 1 °C.

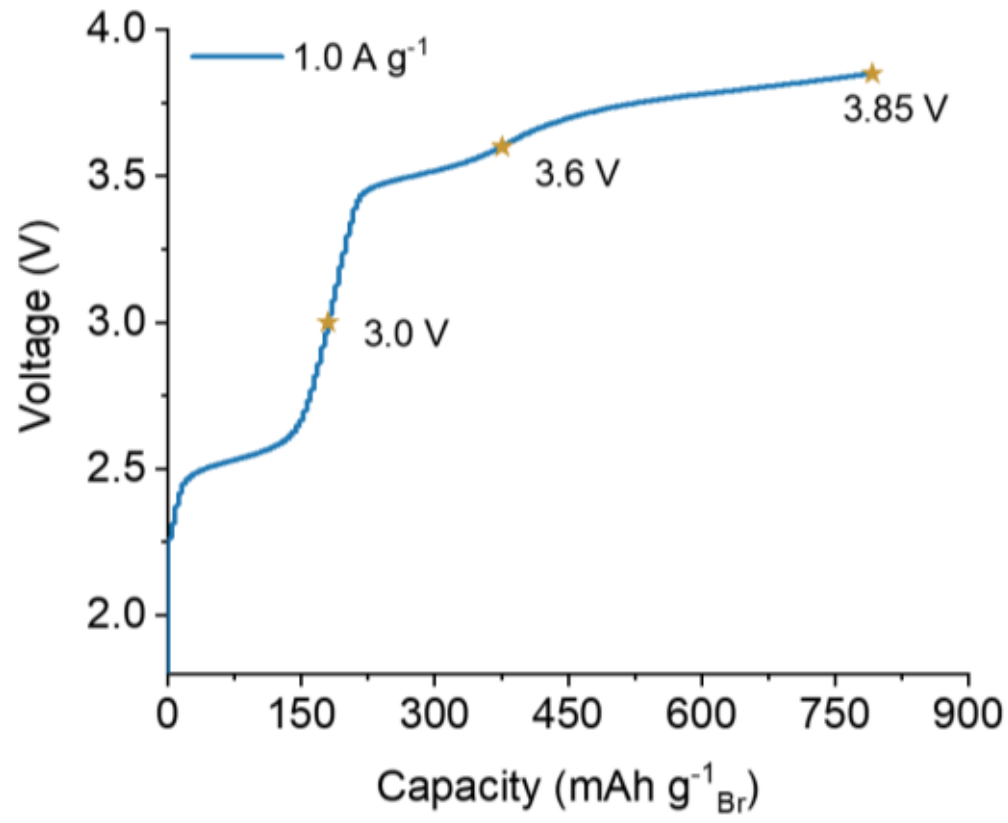

Supplementary Fig. 33 | Electrochemical states for XPS analysis. Charge curve corresponding to XPS tests. All batteries were tested at 25 ± 1 °C.

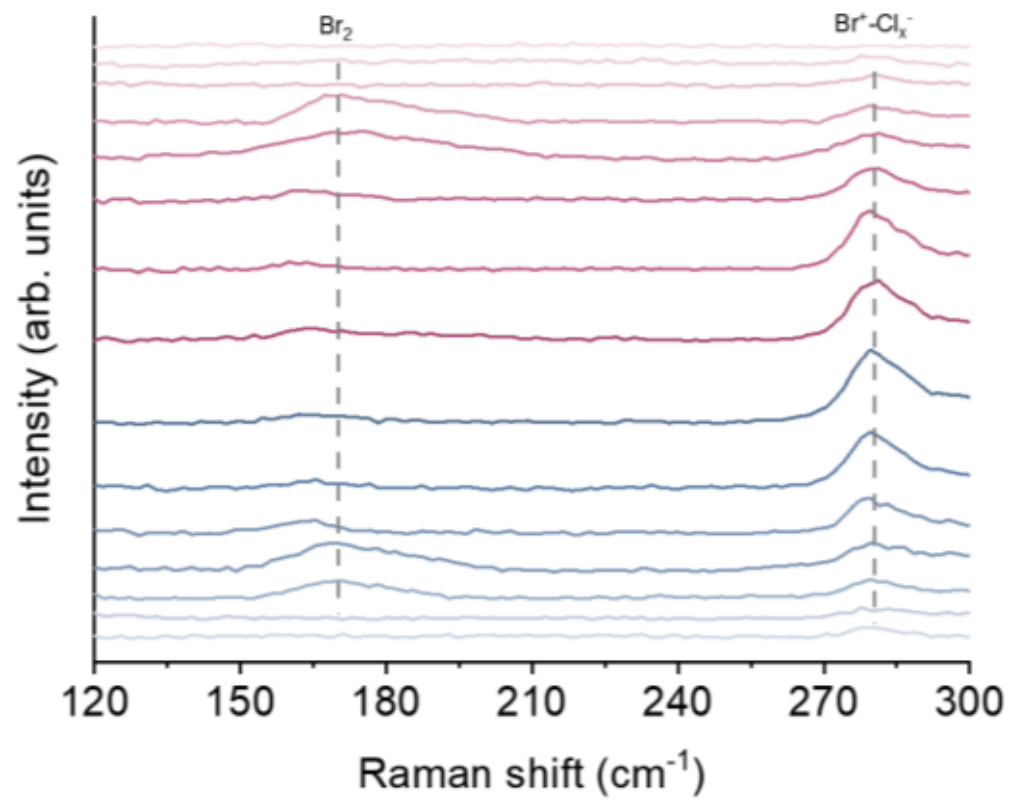

Supplementary Fig. 34 | In situ Raman characterization. In situ Raman spectra during charging and discharging at a specific current of 0.5 A g^{-1}. All electrochemical and characterization tests were conducted at 25 ± 1 °C.

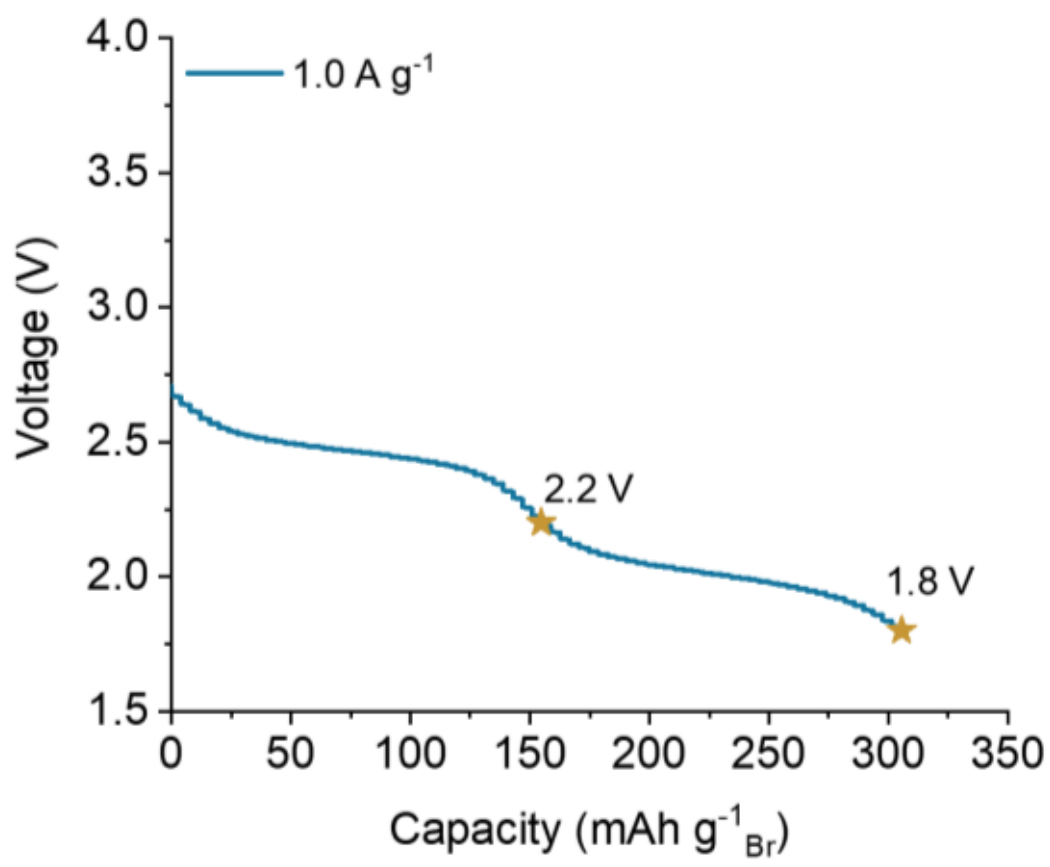

Supplementary Fig. 35 | Electrochemical states for XPS analysis. Discharge curve corresponding to XPS and electron paramagnetic resonance (EPR) tests. All batteries were tested at 25 ± 1 °C.

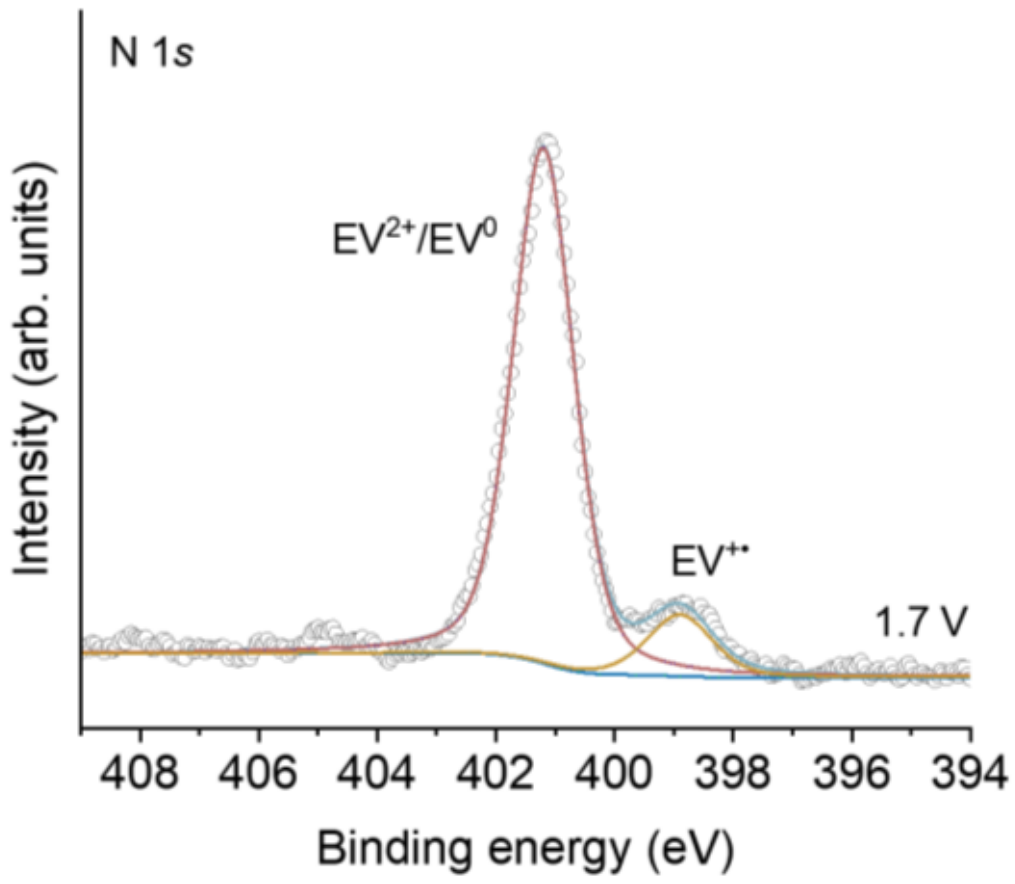

Supplementary Fig. 36 | Nitrogen chemical states at 1.7 V. High-resolution N 1*s* XPS spectra at 1.7 V. The electrodes were obtained from batteries that were first cycled at 1 A g^{-1} for 10 cycles, then discharged to 1.7 V, after which the batteries were disassembled. All electrochemical and characterization tests were conducted at 25 ± 1 °C.

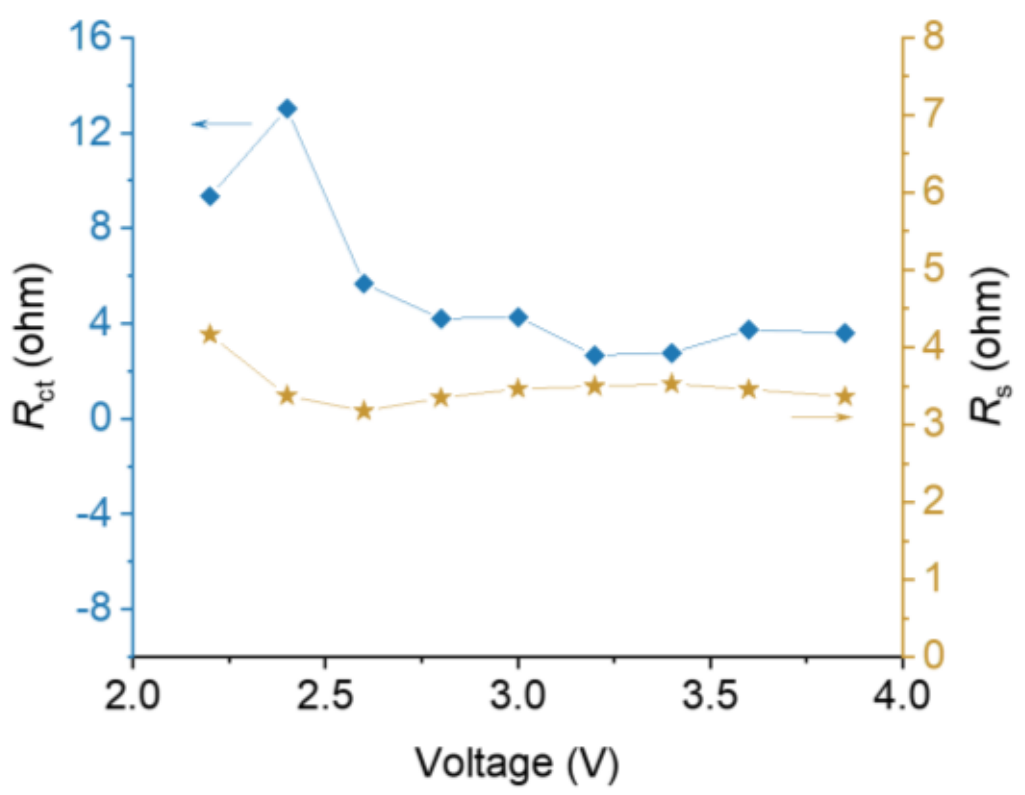

Supplementary Fig. 37 | Interfacial resistance evolution during charging. Curves of ohmic resistance (R_s) and charge transfer resistance (R_{ct}) values during the charging process as a function of SOCs.

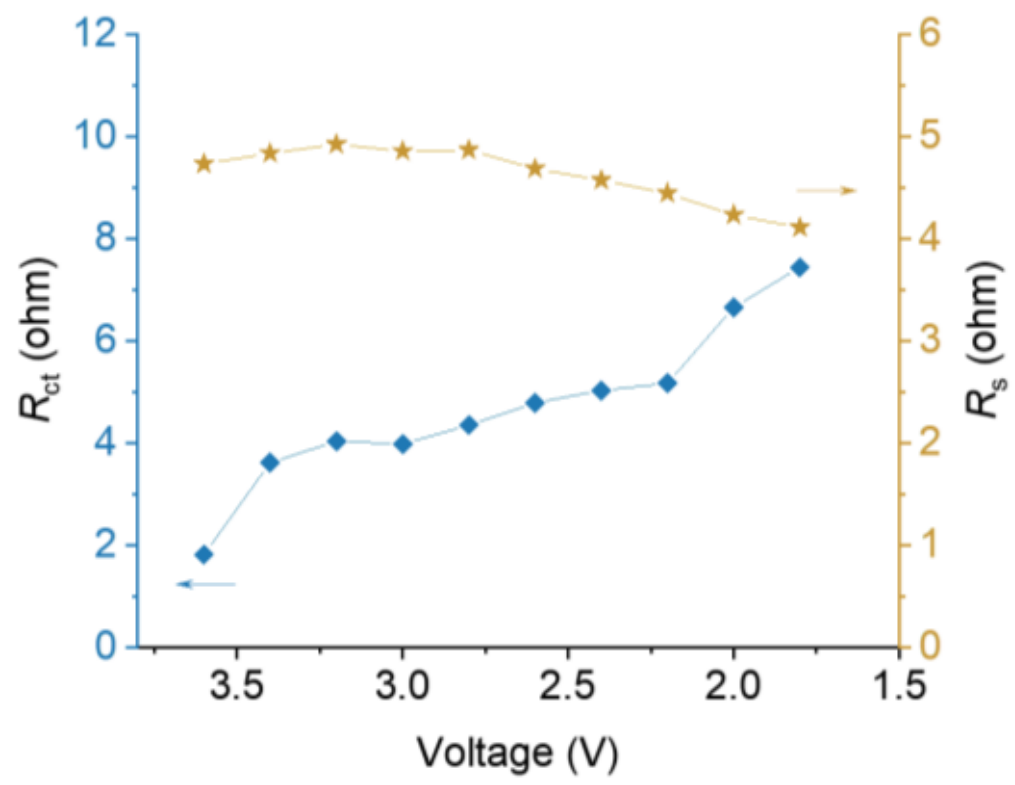

Supplementary Fig. 38 | Interfacial resistance evolution during discharging. Curves of R_s and R_{ct} values during the discharging process as a function of SOCs.

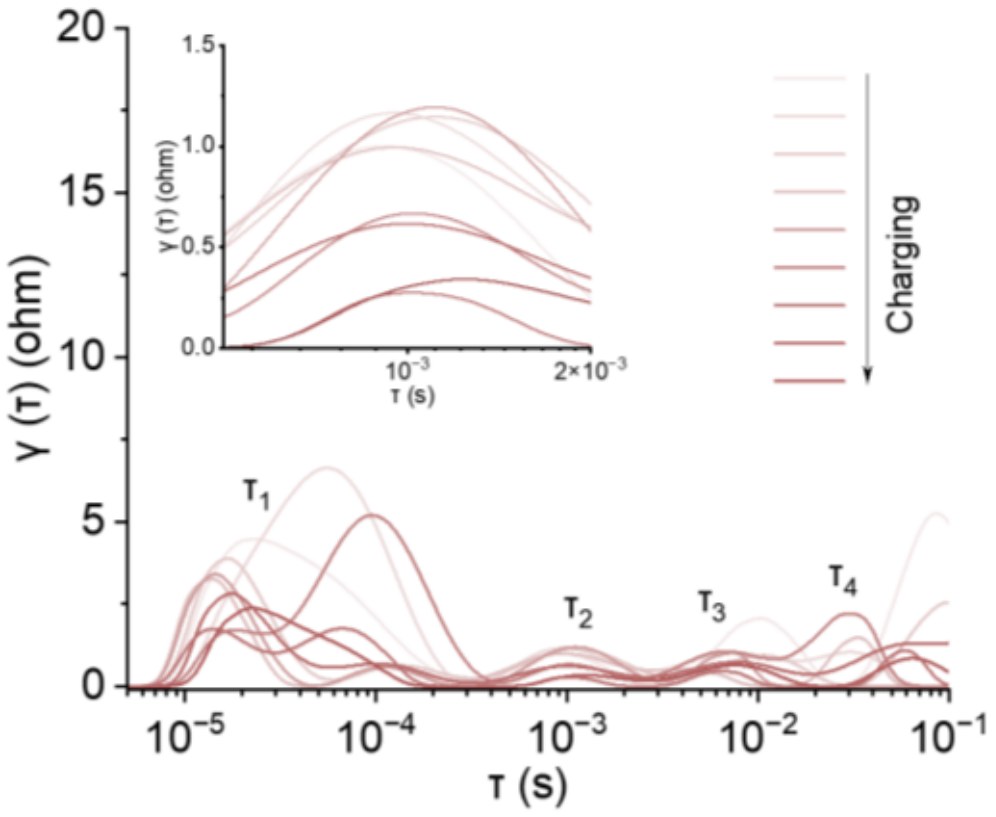

Supplementary Fig. 39 | Relaxation process evolution during charging. Corresponding distribution of relaxation times (DRT) results for the charging process.

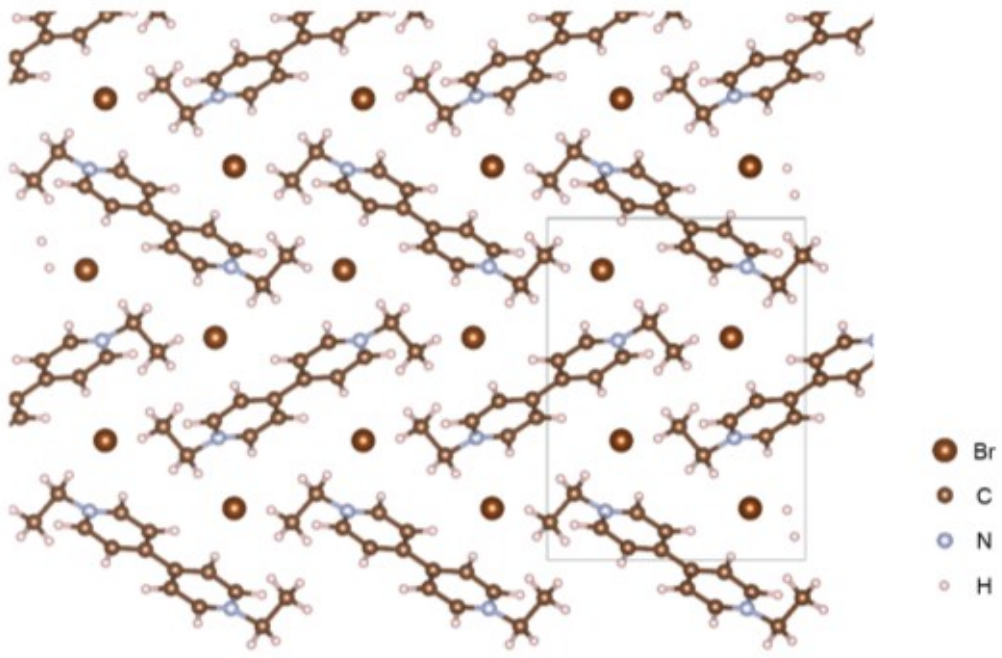

Supplementary Fig. 40 | Crystal structure model. Optimized crystal structure model of $EVBr_2$.

The crystal structure of $EVBr_2$ exhibits monoclinic symmetry, with optimized cell constants of a = 5.45 Å, b = 12.82 Å, and c = 9.97 Å, and a β angle of 103.88°. In the structural representation, carbon atoms are depicted as small brown spheres, nitrogen atoms as blue spheres, hydrogen atoms as white spheres, and bromine atoms as large brown spheres.

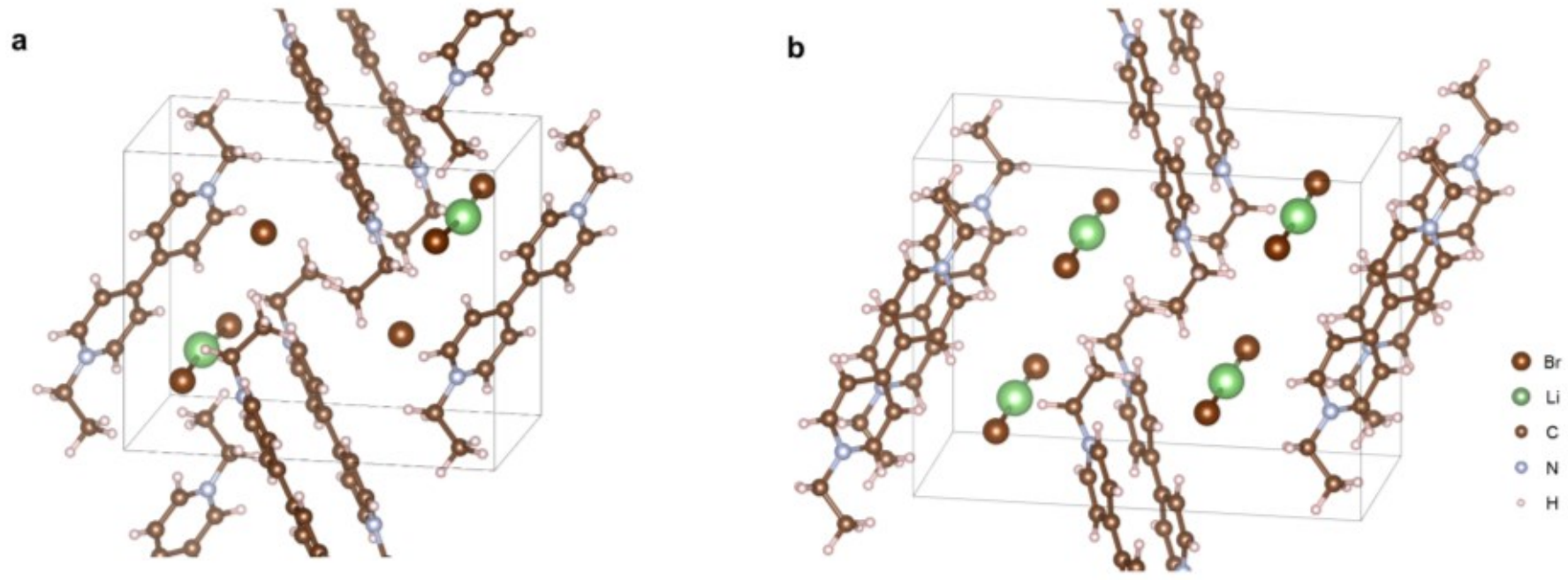

Supplementary Fig. 41 | Structural evolution of $EVBr_2$ upon Li^+ intercalation. Crystal structure model of lithium-ion intercalation. **a** $EV^{+\bullet}Li^+2Br^-$. **b** $EV^0 2Li^+2Br^-$.

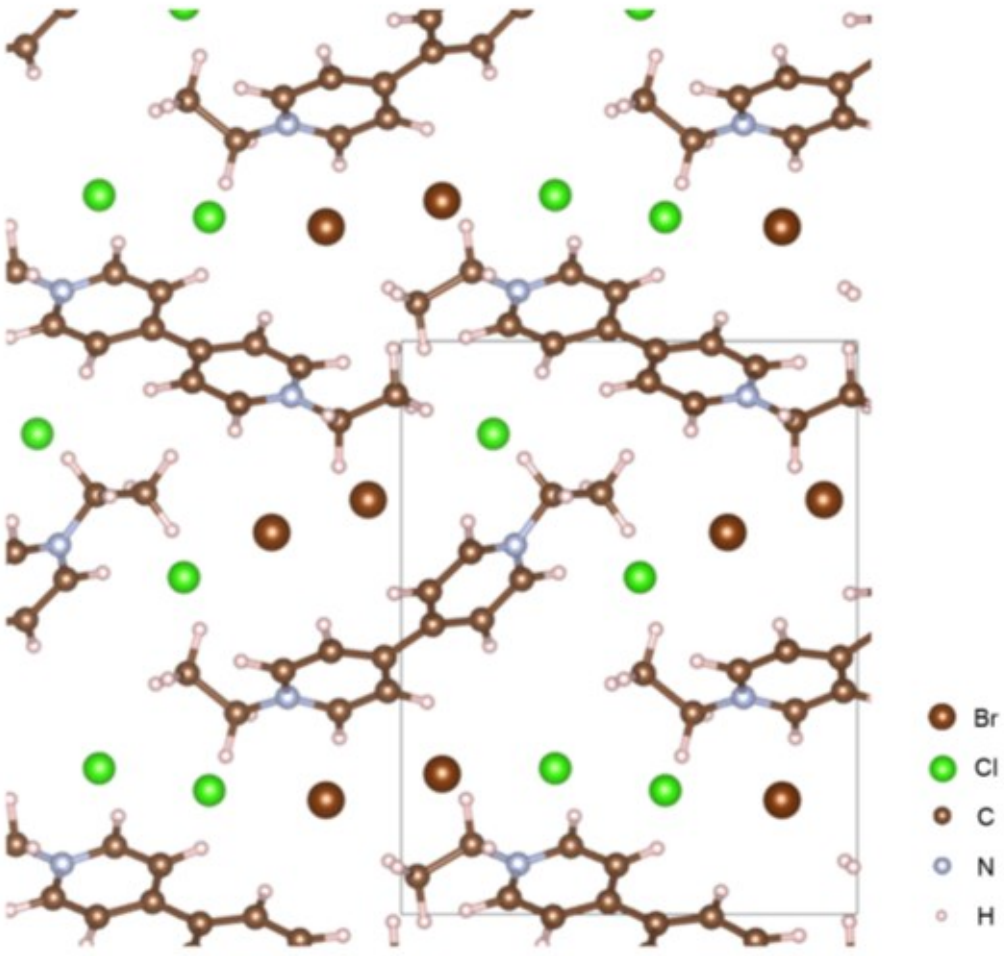

Supplementary Fig. 42 | Crystal structure model. Crystal structure model of $EV^{2+}2Cl^{-}2Br^{0}$.

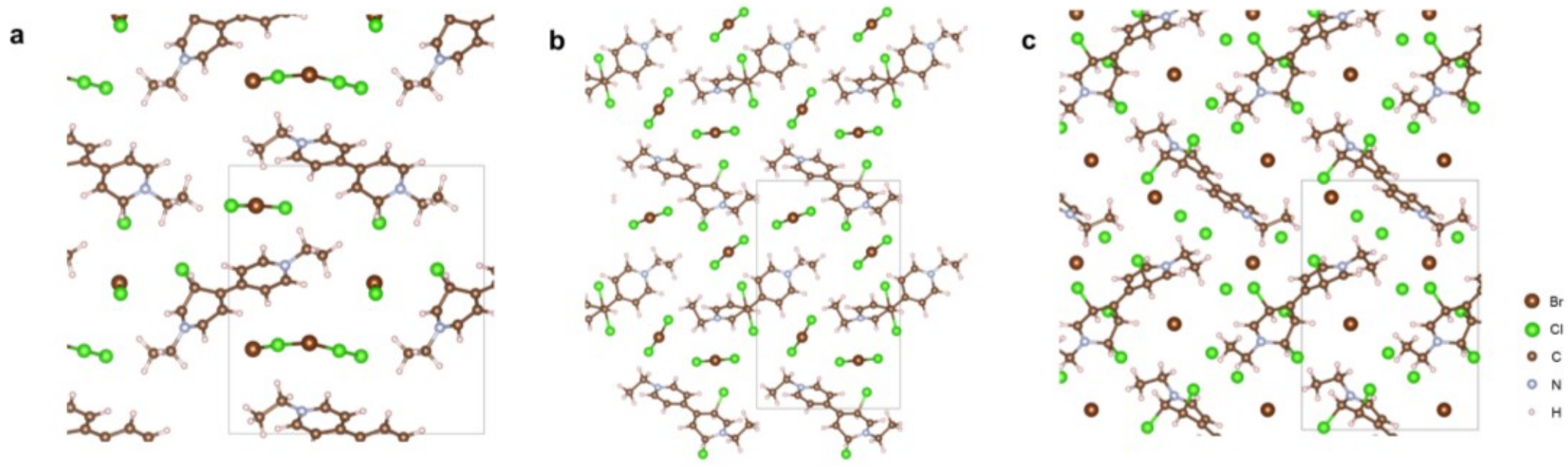

Supplementary Fig. 43 | Crystal structure model. Crystal structure model of materials. **a** $EV^{2+}2Cl^{-}2(BrCl)$. **b** $EV^{2+}2Cl^{-}2(BrCl_2)$. **c** $EV^{2+}2Cl^{-}2(BrCl_3)$.

Supplementary Table 1 | Comparison of the electrochemical performance between this work and state-of-the-art lithium-ion batteries.

Battery type	Number	Plateau voltage (V)	electrolyte volume	electrode mass loading	Reference
Li‖Br	1	3.7	40 μL	1.5-2 mg cm^{-2}	This work
	2	3.5	40 μL	1.5-2 mg cm^{-2}	This work
	3	3.7	overused	1.5 mg cm^{-2}	*Sci. Adv.*[1]
	4	3.4	80 μL	5 mg cm^{-2}	*Adv. Mater.*[2]
	5	3.5	30 μL	4.2 mg cm^{-2}	*Joule*[3]
	6	3.4	126 μL	2 mg	*J. Mater. Chem. A*[4]
	7	3.3	-	-	*J. Energy Chem.*[5]
	8	3.4	-	2 mg cm^{-2}	*Adv. Energy Mater.*[6]
	9	3.4	30 μL	-	*J. Power Sources*[7]
Li‖I	10	3.4	40 μL	1 mg	*J. Am. Chem. Soc.*[8]
	10	2.9	40 μL	1 mg	*J. Am. Chem. Soc.*[8]
	11	3	-	3 mg cm^{-2}	*Adv. Mater.*[9]
	12	2.8	-	2.4-2.8 mg cm^{-2}	*Energy Storage Mater.*[10]
	13	2.8	-	2.2-5.2 mg cm^{-2}	*ACS Nano*[11]
	14	2.8	-	3 mg cm^{-2}	*Angew. Chem. Int. Ed.*[12]
	15	2.8	-	6 mg cm^{-2}	*Energy Environ. Sci.*[13]
	16	2.9	15 μL mg^{-1}	1 mg cm^{-2}	*Adv. Funct. Mater.*[14]
	17	2.7	-	2 mg cm^{-2}	*Carbon Energy*[15]
	18	3	-	0.5 mg cm^{-2}	*Nat. Commun.*[16]
	19	2.9	-	0.6 mg cm^{-2}	*J. Mater. Chem. A*[17]
	20	2.9	-	1-1.5 mg cm^{-2}	*J. Power Sources*[18]
	21	2.8	25 μL	-	*J. Power Sources*[19]
	22	2.7	-	1-1.6 mg cm^{-2}	*J. Mater. Chem. A*[20]
	23	2.8	-	1 mg cm^{-2}	*Angew. Chem. Int. Ed.*[21]
	24	3	-	2 mg cm^{-2}	*Energy Storage Mater.*[22]
Li‖O	25	2.7	100 μL	4 mg cm^{-2}	*Nat. Mater.*[23]
	26	2.8	-	-	*Chem. Commun.*[24]
	27	2.75	180 μL	0.5-0.6 mg cm^{-2}	*Nat. Commun.*[25]
	28	2.8	200 μL	0.1 mg	*Nat. Commun.*[26]
	29	2.7	-	-	*Adv. Mater.*[27]
	30	2.7	100 μL	-	*Adv. Energy Mater.*[28]
	31	2.75	-	0.5 mg cm^{-2}	*Angew. Chem. Int. Ed.*[29]
Li‖S	32	2.2	45 μL	4 mg cm^{-2}	*Joule*[30]

	33	2.1	40 µL	1.2 mg cm^{-2}	*Adv. Sci.*[31]
	34	2.1	5 µL mg^{-1}	2 mg cm^{-2}	*Nat. energy*[32]
	35	1.8	-	-	*Adv. Mater.*[33]
	36	1.75	5 µL mg^{-1}	5 mg cm^{-2}	*Adv. Mater.*[34]
	37	2.15	7.1 µL mg^{-1}	3.5 mg cm^{-2}	*Energy Environ. Sci.*[35]
	38	2.1	7 µL mg^{-1}	4.3 mg cm^{-2}	*Angew. Chem. Int. Ed.*[36]
	39	2	4.8 µL mg^{-1}	1 mg cm^{-2}	*Angew. Chem. Int. Ed.*[37]
	40	1.9	-	1.5 mg cm^{-2}	*Nat. Mater.*[38]
	41	1.85	5 µL mg^{-1}	4 mg cm^{-2}	*Energy Storage Mater.*[39]
	42	2.3	5 µL mg^{-1}	4.2 mg	*ACS Nano*[40]
	43	2.2	-	-	*Adv. Mater.*[41]
	44	2	150 µL	21.6 mg cm^{-2}	*Adv. Mater.*[42]
	45	2	50 µL	8.2 mg cm^{-2}	*Adv. Funct. Mater.*[43]
	46	2.05	15 µL mg^{-1}	1.5-2 mg cm^{-2}	*Adv. Sci.*[44]
	47	2.1	10 µL mg^{-1}	0.7-0.9 mg cm^{-2}	*Adv. Funct. Mater.*[45]
Li||Se	48	2.1	30 µL mg^{-1}	0.8-4 mg cm^{-2}	*J. Mater. Chem. A*[46]
	49	2	-	3.47 mg cm^{-2}	*Adv. Mater.*[47]
	50	1.95	-	1.5 mg cm^{-2}	*Joule*[48]
	51	1.8	60 µL	0.6-0.8 mg cm^{-2}	*Adv. Funct. Mater.*[49]
	52	1.72	20 µL mg^{-1}	3 mg cm^{-2}	*J. Mater. Chem. A*[50]
	53	1.5	-	7.6 mg cm^{-2}	*ACS Nano*[51]
	54	2	-	3 mg cm^{-2}	*Energy Environ. Sci.*[52]
	55	2.1	40 µL	2 mg cm^{-2}	*Adv. Mater.*[53]
	56	1.6	60 µL	1.5 mg cm^{-2}	*Nano Energy*[54]
	57	1.5	40 µL	-	*Adv. Energy Mater.*[55]
	58	2	-	0.5-1 mg cm^{-2}	*Energy Environ. Mater.*[56]
	59	1.8	-	0.72 mg cm^{-2}	*Chem. Eng. J.*[57]
Li||Te	60	1.9	-	-	*Energy Storage Mater.*[58]
	61	1.65	-	1.1 mg cm^{-2}	*ACS Nano*[59]
	62	1.65	-	0.53 mg cm^{-2}	*J. Alloy. Compd.*[60]
	63	1.78	60 µL	1 mg cm^{-2}	*Nano Energy*[61]
	64	2.18	-	-	*Energy Storage Mater.*[62]
	65	1.75	-	-	*Carbon*[63]
	66	1.71	-	0.4 mg	*J. Power Sources*[64]
	67	1.7	-	1 mg cm^{-2}	*Nano Energy*[65]

Supplementary Reference

1. Li, X. L. *et al.* Constructing static two-electron lithium-bromide battery. *Sci. Adv.* **10**, 10 (2024).
2. Zheng, M. T. *et al.* Voltage-Induced Bromide Redox Enables Capacity Restoration of Fast-Charging Batteries. *Adv Mater.* **37**, 2414207 (2024).
3. Xu, J. J. *et al.* Lithium halide cathodes for Li metal batteries. *Joule* **7**, 83-94 (2023).
4. Wang, Y. L., Wang, X., Tian, L. Y., Sun, Y. Y., Ye, S. H. Fixing of highly soluble Br_2/Br^- in porous carbon as a cathode material for rechargeable lithium ion batteries. *J. Mater. Chem. A* **3**, 1879-1883 (2015).
5. Xi, X. L., *et al.* Non-aqueous lithium bromine battery of high energy density with carbon coated membrane. *J. Energy Chem.* **26**, 639-646 (2017).
6. Kim, E., Jamal, H., Jeon, I., Khan, F., Chun, S. E., Kim, J. H. Functionality of 1-Butyl-2,3-Dimethylimidazolium Bromide (BMI-Br) as a Solid Plasticizer in PEO-Based Polymer Electrolyte for Highly Reliable Lithium Metal Batteries. *Adv. Energy Mater.* **13**, 2301674 (2023).
7. Peterson, B. B., Andrews, E. M., Hung, F., Flake, J. C. Carbonized metal-organic framework cathodes for secondary lithium-bromine batteries. *J. Power Sources* **492**, 6 (2021).
8. Zhu, F., Li, Z., Wang, Z., Fu, Y., Guo, W. From Inorganic to Organic Iodine: Stabilization of I^+ Enabling High-Energy Lithium-Iodine Battery. *J. Am. Chem. Soc.* **146**, 11193-11201 (2024).
9. Li, K., Hu, Z., Ma, J., Chen, S., Mu, D., Zhang, J. A 3D and Stable Lithium Anode for High-Performance Lithium-Iodine Batteries. *Adv. Mater.* **31**, 1902399 (2019).
10. Wu, Z. Z. *et al.* LiI embedded meso-micro porous carbon polyhedrons for lithium iodine battery with superior lithium storage properties. *Energy Storage Mater.* **10**, 62-68 (2018).
11. Sun, C., Shi, X., Zhang, Y., Liang, J., Qu, J., Lai, C. $Ti_3C_2T_x$ MXene Interface Layer Driving Ultra-Stable Lithium-Iodine Batteries with Both High Iodine

Content and Mass Loading. *ACS Nano* **14**, 1176-1184 (2020).

12. Guo, C., Han, B., Sun, W., Cao, Y., Zhang, Y., Wang, Y. Hydrogen-Bonded Organic Framework for High-Performance Lithium/Sodium-Iodine Organic Batteries. *Angew. Chem. Int. Ed.* **61**, e202213276 (2022).
13. Wang, Y. L., Sun, Q. L., Zhao, Q. Q., Cao, J. S., Ye, S. H. Rechargeable lithium/iodine battery with superior high-rate capability by using iodine-carbon composite as cathode. *Energy & Environ. Sci.* **4**, 3947-3950 (2011).
14. Chen, Q., Chen, S., Ma, J., Ding, S., Zhang, J. Separation Effect of Graphene Sheets with N, O Codoping and Molybdenum Clusters for Reversible Lithium-Iodine Batteries. *Adv. Funct. Mater.* **34**, 2308272 (2023).
15. Jiang, Y. *et al.* Interpenetrating network-reinforced gel polymer electrolyte for ultra-stable lithium-iodine batteries. *Carbon Energy* **6**, e478 (2024).
16. Cheng, Z. *et al.* Achieving long cycle life for all-solid-state rechargeable Li-I_2 battery by a confined dissolution strategy. *Nat. Commun.* **13**, 9 (2022).
17. Yu, D., Liu, D., Shi, L., Qiu, J., Dai, L. High-performance metal-iodine batteries enabled by a bifunctional dendrite-free Li-Na alloy anode. *J. Mater. Chem. A* **9**, 538-545 (2021).
18. Zhang, Q., Zeng, Y.-H., Ye, S.-H., Liu, S. Inclusion complexation enhanced cycling performance of iodine/carbon composites for lithium-iodine battery. *J. Power Sources* **463**, 228212 (2020).
19. Ren, Y. X., Zhao, T. S., Jiang, H. R., Wu, M. C., Liu, M. A stabilized high-energy Li-polyiodide semi-liquid battery with a dually-protected Li anode. *J. Power Sources* **347**, 136-144 (2017).
20. Li, J. F. *et al.* Dual-functional iodine photoelectrode enabling high performance photo-assisted rechargeable lithium iodine batteries. *J. Mater. Chem. A* **10**, 7326-7332 (2022).
21. Gu, J. *et al.* Constructing Matching Interfaces by Amorphous Engineering for Enhanced Lithium Ion Transport in Quasi-Solid-State Lithium-Iodine Batteries.

Angew. Chem. Int. Ed. **64**, e202507184 (2025).

22. Su, Z., Wei, Z., Lai, C., Deng, H., Liu, Z., Ma, J. Robust pseudo-capacitive Li-I2 battery enabled by catalytic, adsorptive N-doped graphene interlayer. *Energy Storage Mater.* **14**, 129-135 (2018).
23. Ottakam Thotiyl, M. M., Freunberger, S. A., Peng, Z., Chen, Y., Liu, Z., Bruce, P. G. A stable cathode for the aprotic Li-O_2 battery. *Nat. Mater.* **12**, 1050-1056 (2013).
24. Xu, D., Wang, Z.-l., Xu, J.-j., Zhang, L.-l., Zhang, X.-b. Novel DMSO-based electrolyte for high performance rechargeable Li-O_2 batteries. *Chem. Commun.* **48**, 6948-6950 (2012).
25. Wang, P. *et al.* Atomically dispersed cobalt catalyst anchored on nitrogen-doped carbon nanosheets for lithium-oxygen batteries. *Nat. Commun.* **11**, 1576 (2020).
26. Zhang, Z., Xiao, X., Yan, A., Sun, K., Yu, J., Tan, P. Breaking the capacity bottleneck of lithium-oxygen batteries through reconceptualizing transport and nucleation kinetics. *Nat. Commun.* **15**, 9952 (2024).
27. Sun, Z. *et al.* Constructing an Interlaced Catalytic Surface via Fluorine-Doped Bimetallic Oxides for Oxygen Electrode Processes in Li-O_2 Batteries. *Adv. Mater.* **36**, e2404319 (2024).
28. Wu, X.*, et al.* Enhancing the Reaction Kinetics and Reversibility of Li-O_2 Batteries by Multifunctional Polymer Additive. *Adv. Energy Mater.* **13**, 2203089 (2022).
29. Zeng, Y., Zhou, Y., Liu, F., Li, H. Unlocking the Orbital Interaction Mode in Li-O_2 Batteries. *Angew. Chem. Int. Ed.* **65**, e20062 (2026).
30. Li, X.-Y. *et al.* Competitive anion coordination overcomes charge-transfer barriers for lithium-sulfur batteries. *Joule* **10**, 102259 (2026).
31. Yang, Z. *et al.* Exclusive Se-O Coordination and Fe-doping Complementation: A Catalytic Strategy for Enhanced Sulfur Redox in Li-S Batteries. *Adv. Sci.* e13049 (2025).
32. Pang, Q., Shyamsunder, A., Narayanan, B., Kwok, C. Y., Curtiss, L. A., Nazar, L. F. Tuning the electrolyte network structure to invoke quasi-solid state sulfur

conversion and suppress lithium dendrite formation in Li-S batteries. *Nat. Energy* **3**, 783-791 (2018).

33. Wang, J., Yang, J., Xie, J., Xu, N. A Novel Conductive Polymer-Sulfur Composite Cathode Material for Rechargeable Lithium Batteries. *Adv. Mater.* **14**, 963-965 (2002).
34. Peng, L. *et al.* Chemo-Electrochemical Tandem Catalysis Unlocking Sulfur Reaction in Practical Lithium-Sulfur Batteries. *Adv. Mater.* e21611 (2026).
35. Man, Q. *et al.* Electron push-pull engineering enables sustainable, anti-corrosive, and nonflammable phosphate electrolytes for long-lifespan lithium-sulfur batteries. *Energy & Environ. Sci.* 2404093 (2026).
36. Yao, X. *et al.* Advanced Self-Phase-Separating Electrolytes for High-Performance Lithium-Sulfur Batteries. *Angew. Chem. Int. Ed.* **64**, e202519315 (2025).
37. Peng, L. *et al.* Surface Charge-Modulated Electric-Double-Layer Structure on Pt Catalyst for Efficient and Durable Sulfur Reaction in Li-S Batteries. *Angew. Chem. Int. Ed.* **65**, e23287 (2026).
38. Yu, Z., Singh, B., Yu, Y., Nazar, L. F. Suppressing argyrodite oxidation by tuning the host structure for high-areal-capacity all-solid-state lithium-sulfur batteries. *Nat. Mater.* **24**, 1082-1090 (2025).
39. Chen, Z.-X. *et al.* Accelerating the sulfur redox kinetics in weakly-solvating lithium-sulfur batteries under lean-electrolyte conditions. *Energy Storage Mater.* **83**, 104502 (2025).
40. Hou, T. *et al.* Synergistic Adsorption-Catalysis by Co/Ce Dual Single Atoms Trapped in Hollow Carbon Spheres Boosts Li-S Battery Performance. *ACS Nano* **19**, 38654-38668 (2025).
41. Wang, X. *et al.* Dual-Conductivity Optimization Toward High-Rate and Ultralong Life All-Solid-State Lithium-Sulfur Batteries. *Adv. Mater.* e22976 (2026).
42. Zhang, Y. *et al.* Correlated Dual-Gradient Electrodes Enabling Spatially Synchronized Sulfur Redox in High-Mass-Loading Li-S Batteries Under High

Current Densities. *Adv. Mater.* e17190 (2025).

43. Yang, C. *et al.* High-Throughput Carbon Scaffold and Liquid Metal Enable High-Flux Li^+ Transport, Improved Interface Wettability, Dendrite Suppression, and Polysulfide Control in Li-S Batteries. *Adv. Funct. Mater.* e28304 (2026).
44. Qi, Z. *et al.* Co_3O_4/C-NFs Induced 3D Electric Field Enhancement for Dual-Regulation of Polysulfides and Li^+ Transport in Lithium-Sulfur Batteries. *Adv. Sci.* e20167 (2025).
45. Li, M. *et al.* Accelerating Lithium-Ion Transfer and Sulfur Conversion *via* Electrolyte Engineering for Ultra-Stable All-Solid-State Lithium-Sulfur Batteries. *Adv. Funct. Mater.* **35**, 2413580 (2024).
46. Cao, Y. *et al.* A MOF-derived carbon host associated with Fe and Co single atoms for Li-Se batteries. *J. Mater. Chem. A* **9**, 16196-16207 (2021).
47. Li, X*, et al.* Highly Stable Halide-Electrolyte-Based All-Solid-State Li-Se Batteries. *Adv. Mater.* **34**, 2200856 (2022).
48. Jin, Y. *et al.* High-Energy-Density Solid-Electrolyte-Based Liquid Li-S and Li-Se Batteries. *Joule* **4**, 262-274 (2020).
49. Lee, S., Lee, H., Ha, N., Lee, J. T., Jung, J., Eom, K. In Batteria Electrochemical Polymerization to Form a Protective Conducting Layer on Se/C Cathodes for High-Performance Li-Se Batteries. *Adv. Funct. Mater.* **30**, 2000028 (2020).
50. Dong, W.-D*, et al.* The chain-mail Co@C electrocatalyst accelerating one-step solid-phase redox for advanced Li-Se batteries. *J. Mater. Chem. A* **10**, 8059-8067 (2022).
51. Guo, B. Y. *et al.* Cryo-EM Revealing the Origin of Excessive Capacity of the Se Cathode in Sulfide-Based All-Solid-State Li-Se Batteries. *ACS Nano* **16**, 17414-17423 (2022).
52. Li, X. *et al.* High-performance all-solid-state Li-Se batteries induced by sulfide electrolytes. *Energy & Environ. Sci.* **11**, 2828-2832 (2018).
53. Wang, Z. *et al.* In Situ Organoselenization for Ultrastable Li-Se Batteries. *Adv.*

Mater. e23054 (2026).

54. Zhao, X., *et al.* Heteroatoms dual-doped hierarchical porous carbon-selenium composite for durable Li-Se and Na-Se batteries. *Nano Energy* **49**, 137-146 (2018).
55. Kim, S., Cho, M., Lee, Y. High-Performance Li-Se Battery Enabled *via* a One-Piece Cathode Design. *Adv. Energy Mater.* **10**, 1903477 (2020).
56. Mutlu-Cetinkaya, T. *et al.* MIL-91(Al) to Boost Solid-Solid Conversion Reactions in Li-Se Batteries. *Energy & Environ. Mater.* **8**, e70038 (2025).
57. Luo, X.-W. *et al.* 2D ZIF-8 derived N-doped porous carbon nanosheets for high-performance Li-Se batteries. *Chem. Eng. J.* **488**, 150885 (2024).
58. Li, Y. *et al.* Muscle-like electrode design for Li-Te batteries. *Energy Storage Mater.* **10**, 10-15 (2018).
59. He, J. R. *et al.* Three-Dimensional Hierarchical Reduced Graphene Oxide/Tellurium Nanowires: A High-Performance Freestanding Cathode for Li-Te Batteries. *ACS Nano* **10**, 8837-8842 (2016).
60. Liang, P. *et al.* Three-dimensional nanoporous tungsten supported tellurium cathode for Li-Te batteries. *J. Alloys Compd* **861**, 158459 (2021).
61. Rao, S., Wu, R., Zhu, Z., Wu, J., Ding, Y., Mai, L. Tellurium filled carbon nanotubes cathodes for Li-Te batteries with high capacity and long-term cyclability. *Nano Energy* **112**, 108462 (2023).
62. Ge, X., Yin, L. S-doping induced boosted electrochemical redox kinetics in $Te_{1-x}S_x$ nanorod cathodes for high volumetric capacity Li-Te batteries. *Energy Storage Mater.* **20**, 89-97 (2019).
63. Zhang, Y. *et al.* A durable lithium-tellurium battery: Effects of carbon pore structure and tellurium content. *Carbon* **173**, 11-21 (2021).
64. Huang, D. *et al.* Ultrafast synthesis of Te nanorods as cathode materials for lithium-tellurium batteries. *J. Power Sources* **371**, 48-54 (2017).
65. Zhu, J. *et al.* Universal low-temperature and template-free synthesis of sponge-like porous micron-sized elemental materials for high-performance

lithium/potassium storage. *Nano Energy* **95**, 106981 (2022).
