# Supplementary material for: Coordination electrochemistry taming reversible hypervalent bromine redox for energetic six-electron-transfer lithium-bromine battery
Source: Nat Commun. 2026 Jul 18;17:8799. doi: 10.1038/s41467-026-75860-6 (PMC13493775; doi:10.1038/s41467-026-75860-6)
Supplement: Supplementary file 2 [file 41467_2026_75860_MOESM2_ESM.pdf]

Description of Additional Supplementary Files

Supplementary Data 1. The initial and final configurations of the DD and DDC electrolytes.

Supplementary Data 2. Adsorption model of EV^{2+} toward Br^- and Br^0.

Supplementary Data 3. Crystal structure models of $EV^0 2Li^+ 2Br^-$, $EV^{+\bullet} Li^+ 2Br^-$, $EV^{2+} 2Br^-$, $EV^{2+} 2Cl^- 2Br^0$, $EV^{2+} 2Cl^- 2(BrCl)$, $EV^{2+} 2Cl^- 2(BrCl_2)$, and $EV^{2+} 2Cl^- 2(BrCl_3)$.
