# Supplementary material for: Coordination electrochemistry taming reversible hypervalent bromine redox for energetic six-electron-transfer lithium-bromine battery
Source: Nat Commun. 2026 Jul 18;17:8799. doi: 10.1038/s41467-026-75860-6 (PMC13493775; doi:10.1038/s41467-026-75860-6)
Supplement: Supplementary file 3 [file 41467_2026_75860_MOESM4_ESM.pdf]

nature portfolio

Peer Review File

Coordination electrochemistry taming reversible hypervalent bromine redox for energetic six-electron-transfer lithium-bromine battery

Corresponding Author: Professor Xinliang Li

This file contains all reviewer reports in order by version, followed by all author rebuttals in order by version.

Version 0:

Reviewer comments:

Reviewer #1

(Remarks to the Author)
This work represents a groundbreaking advancement in organic lithium-bromine battery systems through the rational design of ethyl viologen dibromide ($EVBr_2$) as an energetic active cathode. The proposed six-electron-transfer redox mechanism, driven by the synergy of intercalation and conversion reactions, constitutes a conceptual leap in the field. Of particular significance is the activation of the reversible hypervalent bromine redox couple (Br^0/Br^+), which yields a high discharge voltage plateau of 3.8 V. Concurrently, the ethyl viologen ligand provides additional capacity via reversible two-step Li^+ ion intercalation/extraction. The achieved ultrahigh energy density of 1810 Wh kg^{-1}Br is exceptional. The authors have conducted an in-depth investigation, and their conclusions are strongly supported by experimental evidence and theoretical calculations. Given the high quality and significance of this research, I am pleased to recommend publication in Nature Communications pending necessary revisions addressing the following points.
[1]The manuscript presents cycling stability data based on a six-electron transfer process. However, it is recommended that the authors additionally provide cycling performance data for the Li||$EVBr_2$ battery in the baseline electrolyte (1.0 M LiTFSI in DME: DOL = 1: 1 vol% with 1% $LiNO_3$), which involves only a four-electron transfer process. This comparison would help to clarify and highlight the contribution of the Br^+/Br^0 redox couple to the overall cycling stability.
[2]The volatility data comparison in Figure 2c demonstrates that $EVBr_2$ exhibits significantly enhanced stability compared to Br_2. However, volatility testing alone may not be sufficient to comprehensively assess the stability of $EVBr_2$. It is suggested that the authors provide additional characterization data to further substantiate the stability analysis.
[3]The new redox peaks observed at 3.82/3.68 V in Figure 2d are attributed to the Br^0/Br^+ conversion. Nevertheless, considering that the DDC electrolyte contains chloride ions, the possibility that this signal originates from the Cl^0/Cl^- redox reaction cannot be completely excluded. The authors are encouraged to provide further evidence to clearly identify the origin of this redox feature.
[4]The key breakthrough of this study lies in the activation of a high-voltage Br^0/Br^+ redox couple, which critically depends on the use of the LiCl-containing DDC electrolyte. A more in-depth analysis of the influence of the electrolyte is therefore recommended to strengthen the mechanistic understanding and validate the feasibility of the proposed system.
[5]The logical flow and data presentation related to the scan rate dependence and b-value analysis in Figures S11 and 3a require improvement. The authors are advised to reorganize the descriptions and sequence of these figures to enhance logical coherence and readability.
[6]The manuscript presents cycling stability data for both Li||$EV(ClO_4)_2$ and Li||$EVBr_2$ batteries, but the vertical axis labeling is inconsistent between these figures. It is recommended to unify the axis labels and units to ensure data consistency and clarity of presentation.
[7]Comparison with other battery system in the introduction is recommended for balance. such as eScience,2025,5,4,100331; Energy Mater 2024;4:400024; Energy Mater. 2025, 5, 500105.

Reviewer #2

(Remarks to the Author)
This article reports on the coupling of an ethyl viologen (EV) ligand with Br, and the introduction of Cl^- to achieve the oxidation of Br^- to Br^+, realizing high-valent halogen conversion. However, the key EV ligand employed by the authors has already been reported in zinc and magnesium batteries (Adv. Energy Mater. 2019, 9, 1903002). Furthermore, the use of Cl^-

to modulate the bromine reaction pathway is not novel either (Sci. Adv. 2024,10, eadl0587). Consequently, the innovation of this work is lacking. Moreover, the evaluation of the core electrochemical mechanisms lacks in-depth interpretation, especially regarding whether the ether-based electrolyte undergoes continuous oxidative decomposition at high voltage, and the formation of the key species Br-Cl lacks clear supporting evidence, casting doubt on the main conclusions. Specific issues are as follows:

1. EV is claimed to have coordination capability towards bromine in different states [Br], but no detailed explanation is provided. The strength of this strong coordination capability towards halogens in different valence and coordination states, the potential competitive relationships, and the proportion of free halogens need to be demonstrated.

2. The authors attribute capacity decay to minor side reactions. However, the reaction process in this system is highly complex. The investigation into capacity fade should analyze each electrochemical process individually. The cycling efficiency for each electrochemical reaction should be provided.

3. The Br0/Br+ redox reaction occurs at a high voltage of 3.85 V, which exceeds the typical anodic stability limit of conventional ether-based electrolytes (DOL/DME). The authors need to provide evidence, such as the oxidative current in CV or gas chromatography-mass spectrometry data, to prove the electrolyte's stability at this high voltage or the formation of an effective Cathode Electrolyte Interphase (CEI).

4. The solubility of the EV-based active material needs to be assessed, along with an evaluation of its shuttle effect and reactivity towards lithium metal.

5. The composition and morphological changes of the anode SEI need to be provided. Is the adsorption efficiency of the EV ligand for bromine 100%? If not, does it participate in and affect the composition of both the CEI and SEI?

6. The solubility of LiCl and LiBr in ether-based solvents is low. Please provide the concentrations of Cl^- and Br^- ions in the described battery system and discuss their potential changes.

7. Both Br0 and Br+ are strong oxidizing agents. How can they stably coexist with solvents like DME and DOL, as well as the separator?

8. The authors mention a four-step electrochemical process for this six-electron transfer system. Is there a rate-determining step among these?

9. The influence of the introduced EV ligand and halogens on the Li^+ solvation structure needs to be evaluated.

10. The depiction of Br^0 in Fig. 5 is confusing. The authors previously stated that Br0 exists in the form of Br_3^-, yet in Fig. 5, Br0 is shown coordinated/adsorbed to the EV ligand. Please clarify the existence state of Br0.

11. The authors claim extremely fast kinetics for this electrochemical system. However, kinetics are directly related to concentration. Given the presence of coordination and adsorption relationships, what are the threshold concentrations of Cl^-, Br^-, and EV required to achieve the nearly current-density-independent CV curves mentioned in the text?

12. A comparative analysis with recent advances in multi-electron bromine batteries is missing. The authors should include a performance comparison table in the Supplementary Information to clearly contextualize the advancements claimed in this work.

13. The assignment of the peak at 270 cm-1 in the in-situ Raman spectra to the key species Br-Cl requires support from literature references.
14. The text only provides a single-cycle curve for EV. The electrochemical performance of EV itself as an organic electrode material also needs to be provided.

15. Several abbreviations, such as "GITT" (Galvanostatic Intermittent Titration Technique), are not defined upon their first appearance in the text. This may hinder comprehension for readers unfamiliar with the terminology. It is recommended that all specialized terms or acronyms be spelled out in full at their first occurrence.

Reviewer #3

(Remarks to the Author)
I co-reviewed this manuscript with one of the reviewers who provided the listed reports. This is part of the Nature Communications initiative to facilitate training in peer review and to provide appropriate recognition for Early Career Researchers who co-review manuscripts.

Reviewer #4

(Remarks to the Author)
The authors present their scientific work in the manuscript entitled “Coordination electrochemistry taming reversible hypervalent bromine redox for energetic six-electron-transfer lithium-bromine battery”. This article extends the application of

ethyl viologen from magnesium and lithium-air batteries to utilise ethyl viologen dibromoide as a cathode for lithium-bromine batteries. It well-justifies the application based on the volatility, sluggish reaction kinetics, and energy density limitations of Br-based batteries. Although this article achieves a capacity of 632.8 mAh g-1Br and an energy density of 1810 Wh kg-1Br using a six-electron-transfer mechanism, several major issues need to be addressed:
1) The quality of English writing is poor and requires significant editing. To give just three examples of when the writing noticeably caused confusion and broke the flow of the manuscript.
There are contradictions, imprecise terminology, and poor grammar:
o "Nevertheless, the currently available obsolete two-electron redox mechanism of Br (2Br- /Br2) and the electrochemical inertia of nonhalogenated ligands impose the performance bottleneck." (Pg1)
Problems with formality, punctuation, non-sequitur, and tense:
o "Besides, current investigations on Br redox prioritize two-electron transfer reactions, while four-electron transfer systems delivering heightened voltage and augmented theoretical capacity receive insufficient attention.23, 31 The disproportionation of hypervalent Br+ poses significant challenge for reversible electrochemical process.32, 33" (Pg 3)
And commonplace use of unscientific hyperboles and superlatives:
o "Electrochemical benchmarking in Fig. 3g confirms the unparalleled voltage supremacy of EVBr2." (Pg 10)
2) The article claims that "Theoretical analyses indicate that realizing the four-electron redox process (2Br-/2Br+) would constitute a transformative advancement.21-23" (Pg 3) However, at the same charge rate, one of articles cited utilises two-electron-transfer to achieve a higher capacity of 653 mAh g−1Br and energy density of 2180 Wh kg−1Br than the six-electron-transfer claimed in this article. Why is six-electron-transfer delivering comparable performances? The argument of the article may need to be refined accordingly.
3) At ~2.2 V in Fig. 3a, a small shoulder for peak 1 is observable, also visible as a slight bump in Fig. 3c. What is attributable to this small peak? Can the argument that no side-reactions or byproducts are produced be reinforced using other techniques?
4) The article claims that Br+ Br0 is reversible when arguing that "Newly enabled Br0/Br+ redox chemistry doubles Br-derived capacity while confirming reversible conversion between Br0 and Br+". However, Fig. 3a appears to feature abrupt current increases at ~3.8 V, particularly at faster charge rates, indicative of irreversible Br redox. Can CV curves of slightly higher voltage cut-offs be provided to clarify this?
5) Relatedly, the article argues that four-electron Br transfer has resulted in "activation of a 3.8 V voltage plateau" however the article's CV, charge-discharge curves, and dQ/dV curve appear to have ~3.8 V voltage cut-offs. The fourth dQ/dV peak appears to occur at <3.75 V at all charge rates. Can this please be clarified? If the manuscript is arguing that EVBr2 in DDC enables "reversible hypervalent bromine redox", why was a voltage so proximate to the reversible voltage plateau chosen as the cut-off voltage?
6) As seen in Fig. 3b, there is an extremely sharp drop in capacity from 1.0 to 1.5 Ag-1. What causes this rapid capacity loss considering that the article argues the material has good reaction kinetics from GITT and in-situ EIS?
7) Long-term cycling data in Fig. 3e is only presented as a percentage retention at 3Ag-1. Specific capacity should be shown on the y-axis, like in Supplementary Fig. 7. Is there long-term cycling data at 1/1.5 Ag-1 to compare to the high-rate cycling data? Additionally, why does dramatic capacity fading appear to occur within the first 50 cycles before it stabilises?
8) The article states that "Consequently, the realized Li||EVBr2 battery reached 1810 Wh kg-1Br at a current density of 1 A g-1, exceeding all reported Br-based counterparts.15, 31" (Pg 9) However, the literature cited claims to reach an energy density of 2180 Wh kg−1Br at 1A g-1.
9) The main text states that, "During the charging phase, the deconvolution of the Br 3d spectrum at 3.0 V revealed two peaks at 68.3 eV and 69.3 eV, which are characteristic of Br- (Supplementary Fig. 15)." However, Supplementary Fig. 15 does not display the deconvolution of the Br 3d spectrum at 3.0 V.
10) From the N 1s XPS, the article argues that "The weak residual peak at 398.8 eV originates from a minor amount of EV+• formed via reaction of EV0 and EV2+.37, 38, 57" Based on the literature cited, further oxidation should enable complete oxidation to EV2+. Did the authors try a lower voltage cut-off to confirm this N 1s peak was EV+•? If there is residual EV+•, could a higher capacity not be achieved?
11) Self-discharge is widely recognised as a challenge for Br-based batteries, as identified by comments on the thermodynamic instability and volatility of bromine in the introduction. The results on improved shelf-life were clearly presented to address this challenge. However, shelf-life and self-discharge are not perfectly correlated. Could these results not be reinforced by a comparison of the self-discharge rates for lithium-bromine batteries before and after ethyl-viologen modification?
12) The introduction provides quality background info on the strengths and limitations of bromine for halogen batteries, but only briefly mentions lithium-bromine batteries and the role of ligands. Background on Cl- ligands, work done to stabilise two electron transfer lithium-bromine, and a more rigorous explanation of how "ligands simultaneously functioning as redox-active charge reservoirs via rocking-chair-type mechanism (Fig. 1c)" would help provide theoretical explanations and clarify decisions in the article (e.g. the role of LiCl in DDC).
13) The SEM image appears to be missing from Supplementary Fig. 1, with only EDS mapping provided.
14) The citations associated with Fig. 3g are not clear at first glance.

Reviewer #6

(Remarks to the Author)
There has been significant interest in new cathode materials for reversible Li-ion batteries. This paper explores a (relatively) novel concept in a 6e- reduction ethyl viologen dibromide cathode.

1. Bromine and viologens have been considered for lithium ion battery cathode reactions for some time. A cursory search for "ethyl viologen lithium bromine battery" returns over 600 somewhat related publications since 2021. The introduction includes some information related to the state of the art but the novelty of this approach is not clear relative to other related

publications included several (ACS Appl. Energy Mater. 2024, 7, 18, 7615–7623) that were referenced by the authors. In my view, the introduction is incomplete in terms of outlining the novelty and "state of the art."

10.1016/j.cej.2023.142624
10.1038/s41467-025-63272-x
10.1039/D4CS00585F
10.1021/acsaem.3c02025

2. The stability and CE @ 3A/g in Figure 3 are very good. It would be helpful to know this rate relative to the total cathode capacity. Likewise it would be useful to know the current density. How does the CE and capacity retention perform at higher current densities / rates?

3. At a practical level, the problem of multiple discharge potentials (Figure 3 b and c) limit the usefulness of such a cathode. Further, while these materials may be earth-abundant (lower cost than conventional Li-ion cathodes), the EV is organic and is subject to degradation over time (particularly at higher temperatures and higher current densities).

Version 1:

Reviewer comments:

Reviewer #1

(Remarks to the Author)
well update. It can be recommended for publication now.

Reviewer #2

(Remarks to the Author)
My questions have been well responded. Thank the authors.

Reviewer #3

(Remarks to the Author)
I co-reviewed this manuscript with one of the reviewers who provided the listed reports. This is part of the Nature Communications initiative to facilitate training in peer review and to provide appropriate recognition for Early Career Researchers who co-review manuscripts.

Reviewer #4

(Remarks to the Author)
The revised manuscript has provided a thorough response to the reviewers' comments. In particular, the language is noticeably more professional and the deconstruction of individual electrochemical processes with clear rationales for design decisions has significantly improved the rigorousness of the manuscript. The paper can be accepted for publication; however, several modifications could further improve the manuscript.
1) The analysis of SEI thickness in Supplementary Figures 21 and 23 is a little tenuous, particularly for the CEI due to the low difference in contrast and lack of variance in crystallinity. Further images reproducing this thickness or alternative techniques such as XPS depth profiling would significantly improve the confidence of this finding.
2) The experimental details are very brief. Important details that effect electrochemical performance (e.g. Li foil thickness, coating method, electrolyte volume) as well as sample preparation procedures and instrument parameters for characterisation techniques would aid in reproducibility.
3) The supplementary information requires editing. For example, KB is written as "Ketien blank" and NMP as "N-Methypyrrolidone" or energy-dispersive X-ray spectroscopy is abbreviated as EDX but subsequently referred to as EDS.

Reviewer #6

(Remarks to the Author)
The authors have addressed the majority of my concerns. This is good work and a good manuscript.

Open Access This Peer Review File is licensed under a Creative Commons Attribution 4.0 International License, which permits use, sharing, adaptation, distribution and reproduction in any medium or format, as long as you give appropriate credit to the original author(s) and the source, provide a link to the Creative Commons license, and indicate if changes were made.
In cases where reviewers are anonymous, credit should be given to 'Anonymous Referee' and the source.
The images or other third party material in this Peer Review File are included in the article's Creative Commons license, unless indicated otherwise in a credit line to the material. If material is not included in the article's Creative Commons license and your intended use is not permitted by statutory regulation or exceeds the permitted use, you will need to obtain permission directly from the copyright holder.
To view a copy of this license, visit https://creativecommons.org/licenses/by/4.0/

Response to Reviewers

Dear Reviewers:

Thanks a lot for your constructive comments and suggestions about our manuscript entitled "***Coordination electrochemistry taming reversible hypervalent bromine redox for energetic six-electron-transfer lithium-bromine battery***" (NCOMMS-25-81076-T). Those comments are valuable and helpful for improving the quality of our paper and promoting our further research work. **We have studied your comments very carefully and substantially revised our manuscript in the hope of addressing your concerns.** The comments are addressed point-by-point below, and changes have been highlighted in the revised main text and supporting information.

REVIEWER COMMENTS

To Reviewer #1:

This work represents a groundbreaking advancement in organic lithium-bromine battery systems through the rational design of ethyl viologen dibromide (EVBr$_2$*) as an energetic active cathode. The proposed six-electron-transfer redox mechanism, driven by the synergy of intercalation and conversion reactions, constitutes a conceptual leap in the field. Of particular significance is the activation of the reversible hypervalent bromine redox couple (*Br^0/Br^+*), which yields a high discharge voltage plateau of 3.8 V. Concurrently, the ethyl viologen ligand provides additional capacity via reversible two-step *Li^+* ion intercalation/extraction. The achieved ultrahigh energy density of 1810 Wh *kg^{-1}_{Br}* is exceptional. The authors have conducted an in-depth investigation, and their conclusions are strongly supported by experimental evidence and theoretical calculations. Given the high quality and significance of this research,* ***I am pleased to recommend publication in Nature Communications pending necessary revisions addressing the following points***.

Response: We sincerely thank the reviewer for the thorough evaluation and positive assessment of this work. These critical suggestions have been invaluable in improving our manuscript. We have addressed every point in detail, incorporating new supporting data and expanded analysis. Our responses are outlined below, and we hope the revised manuscript is now suitable for publication.

Comment 1: *The manuscript presents cycling stability data based on a six-electron transfer process. However, it is recommended that the authors additionally provide cycling performance data for the Li||EVBr*$_2$* battery in the baseline electrolyte (1.0 M LiTFSI in DME: DOL = 1: 1 vol% with 1% LiNO*$_3$*), which involves only a four-electron transfer process. This comparison would help to clarify and highlight the contribution of the *Br^+/Br^0* redox couple to the overall cycling stability.*

Response: Thank you for your constructive comments and valuable suggestions. In response, we performed additional control experiments. In the DD electrolyte, the electrochemistry of $EVBr_2$ is restricted to the viologen redox couples ($EV^{2+}/EV^{+\bullet}$ and $EV^{+\bullet}/EV^0$) along with the Br^-/Br^0 process, representing a theoretical four-electron

system. When tested under identical conditions, this system exhibits a specific capacity decay rate of 0.05% per cycle and a significantly lower capacity retention compared to the six-electron system (**Fig. R1**). This direct comparison confirms the superior cycling stability contributed by the Br^0/Br^+ redox couple.

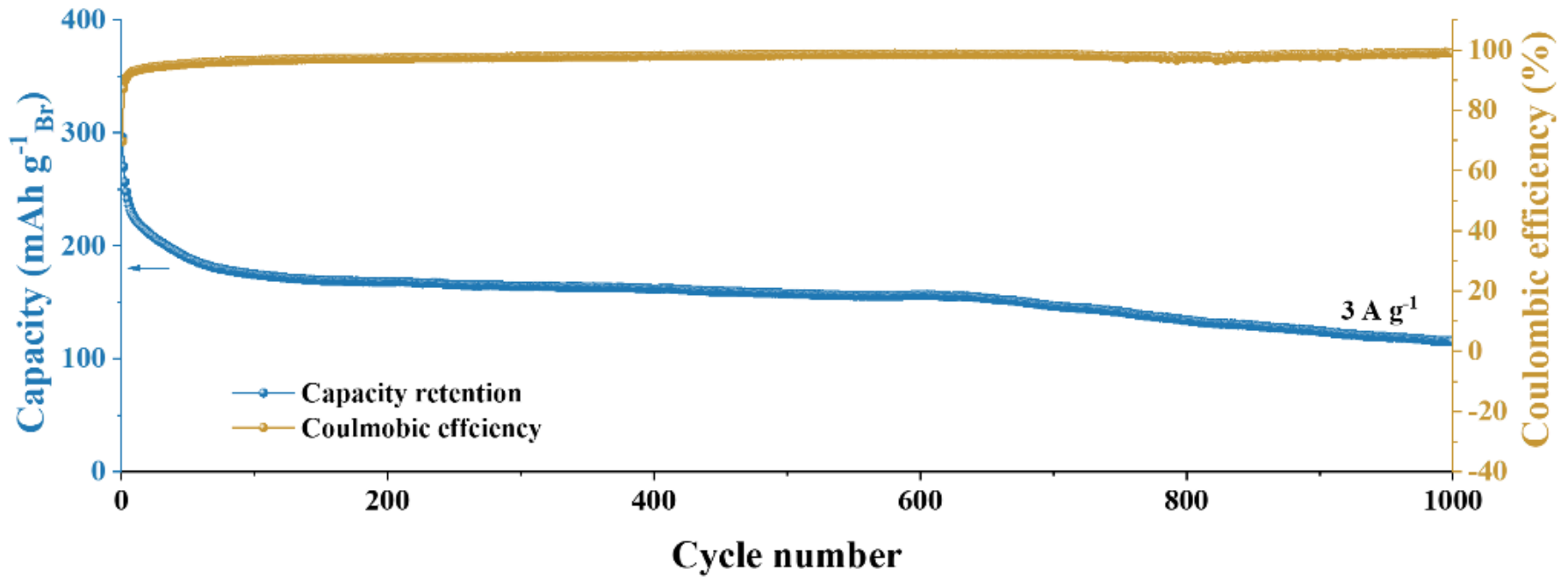

Fig. R1 | Long-term cycling performance of Li||$EVBr_2$ battery in DD electrolyte at 3 A g^{-1}.

Please check the newly added **Fig. S12** on **Page 8** in the supplementary information and the newly added discussion on **Page 7** in the main text.

Comment 2: *The volatility data comparison in Figure 2c demonstrates that $EVBr_2$ exhibits significantly enhanced stability compared to Br_2. However, volatility testing alone may not be sufficient to comprehensively assess the stability of $EVBr_2$. It is suggested that the authors provide additional characterization data to further substantiate the stability analysis.*

Response: Thank you for your constructive comments and valuable suggestions. We agree that relying solely on volatility measurements is not sufficient for a comprehensive assessment of the thermal stability of $EVBr_2$. In response, we performed additional thermogravimetric analysis (TGA). The results show no appreciable mass loss for $EVBr_2$ up to 250 °C (**Fig. R2**), confirming its excellent intrinsic thermal stability. This provides strong complementary evidence for the viability of $EVBr_2$ as an electrode material.

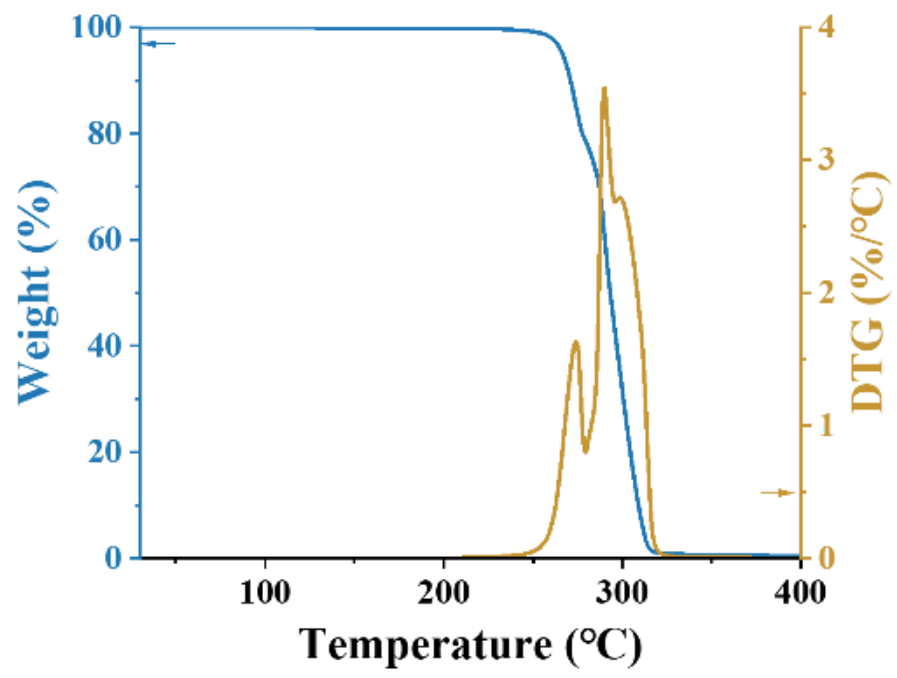

Fig. R2 | The TGA curve of $EVBr_2$ in the N_2 atmosphere.

Please check the newly added **Fig. 2d** on **Page 9** and the newly added discussion on **Page 5** in the main text.

Comment 3: *The new redox peaks observed at 3.82/3.68 V in Figure 2d are attributed to the Br^0/Br^+ conversion. Nevertheless, considering that the DDC electrolyte contains chloride ions, the possibility that this signal originates from the Cl^0/Cl^- redox reaction cannot be completely excluded. The authors are encouraged to provide further evidence to clearly identify the origin of this redox feature.*

Response: Thank you for your constructive comment and valuable suggestion. To unambiguously assign the redox peaks, a control experiment was performed by using an inert cathode of Ketjen Black (KB) and PVDF (mass ratio 9:1, without the active material) in the identical DDC electrolyte. The CV of this inert electrode reveals no discernible redox activity around 3.82/3.68 V within the 1.8-4.0 V window, indicating that the redox signals in this region originate exclusively from $EVBr_2$, not from chloride ions or the carbon substrate (**Fig. R3**).

A weak but observable redox feature was detected at ~4.0/3.85 V. This minor feature is assigned to the Cl^-/Cl^0 redox process, consistent with its higher theoretical oxidation potential compared to Br^0/Br^+. To verify this assignment, we extended the upper cut-off potential to 4.0 V for the $EVBr_2$ cathode (**Fig. R4**). This clearly led to the appearance of the corresponding redox pair at higher potentials, confirming the potential region for the Cl^-/Cl^0 reaction.

In summary, the redox peaks at 3.82/3.68 V in **Fig. 2d** are unequivocally assigned

to the Br^0/Br^+ conversion in $EVBr_2$. In contrast, the Cl^-/Cl^0 reaction occurs at higher potentials and gives a much weaker electrochemical response.

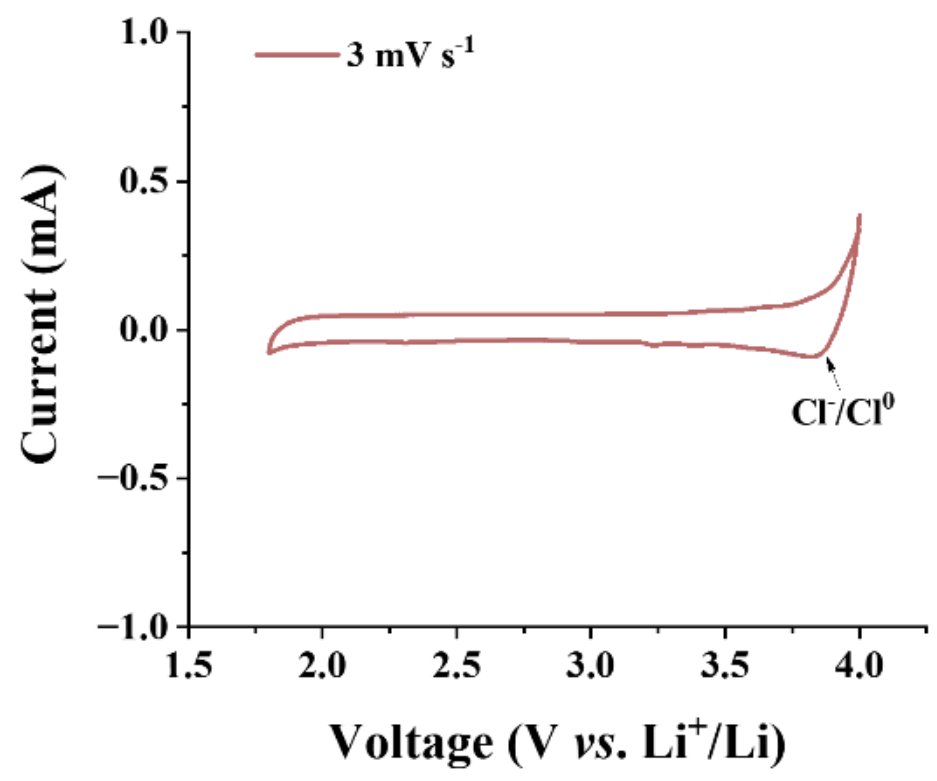

Fig. R3 | CV curve of Li||KB battery in DDC electrolyte.

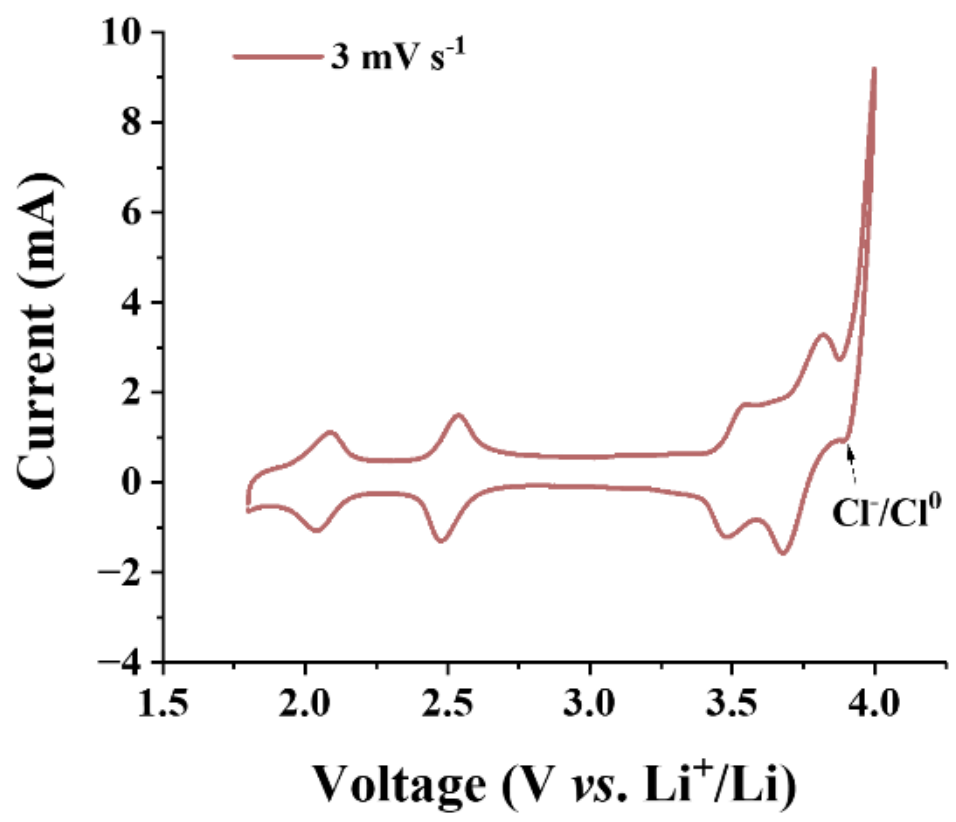

Fig. R4 | CV curve of Li||$EVBr_2$ battery in DDC electrolyte.

Please check the newly added **Figs. S14 and S15a** on **Pages 9-10** in the supplementary information, and the newly added discussion on **Page 8** in the main text.

Comment 4: *The key breakthrough of this study lies in the activation of a high-voltage* Br^0/Br^+ *redox couple, which critically depends on the use of the LiCl-containing DDC electrolyte. A more in-depth analysis of the influence of the electrolyte is therefore recommended to strengthen the mechanistic understanding and validate the feasibility of the proposed system.*

Response: Thank you for your constructive comments and valuable suggestions. To

gain deeper insights into the impact of the electrolyte on electrochemical performance, we performed an extensive set of supplementary experiments. First, LSV curves of Li//SS (stainless steel) batteries shows no significant anodic current up to 4.0 V (*vs*. Li^+/Li) in either DD or DDC electrolytes (**Fig. R5**). This indicates that both electrolytes provide a sufficiently wide electrochemical window to accommodate the operating potential of the Br^0/Br^+ redox couple, with negligible bulk decomposition in this voltage region. Second, CV curves of Li//SS batteries demonstrate that, in the DDC electrolyte, the onset potential for lithium deposition shifts positively, accompanied by reduced polarization between the deposition and stripping peaks (**Fig. R6**). This behavior indicates that the introduction of LiCl effectively improves the interfacial kinetics at the lithium metal anode, facilitating more homogeneous and reversible lithium deposition. Third, EIS analysis reveals that Li//Li batteries in the DDC electrolyte exhibit a lower charge transfer resistance (R_{ct}), signifying the formation of a lower-impedance electrode-electrolyte interface that enables more efficient interfacial charge transfer (**Fig. R7**). Together, these interfacial improvements are directly reflected in the performance of symmetric Li//Li batteries, which show reduced voltage polarization in the DDC electrolyte compared to the DD electrolyte during rate capability and long-term cycling tests, confirming enhanced interfacial stability and kinetics (**Fig. R8**).

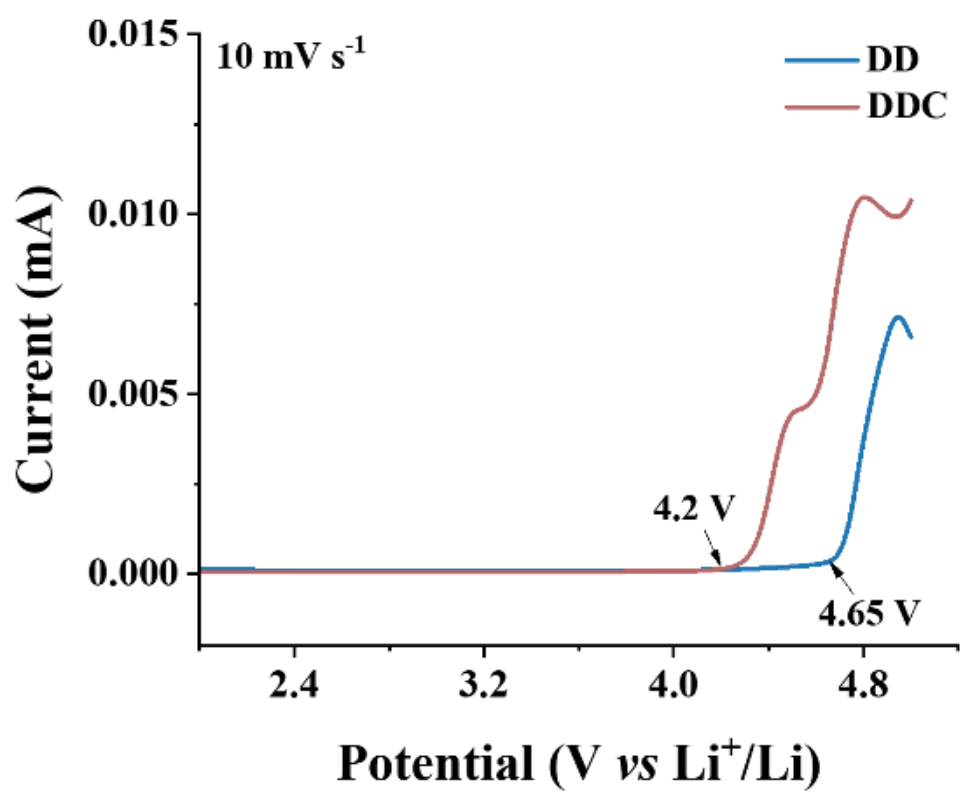

Fig. R5 | LSV curves of asymmetric Li||SS battery at 10 mV s^{-1}.

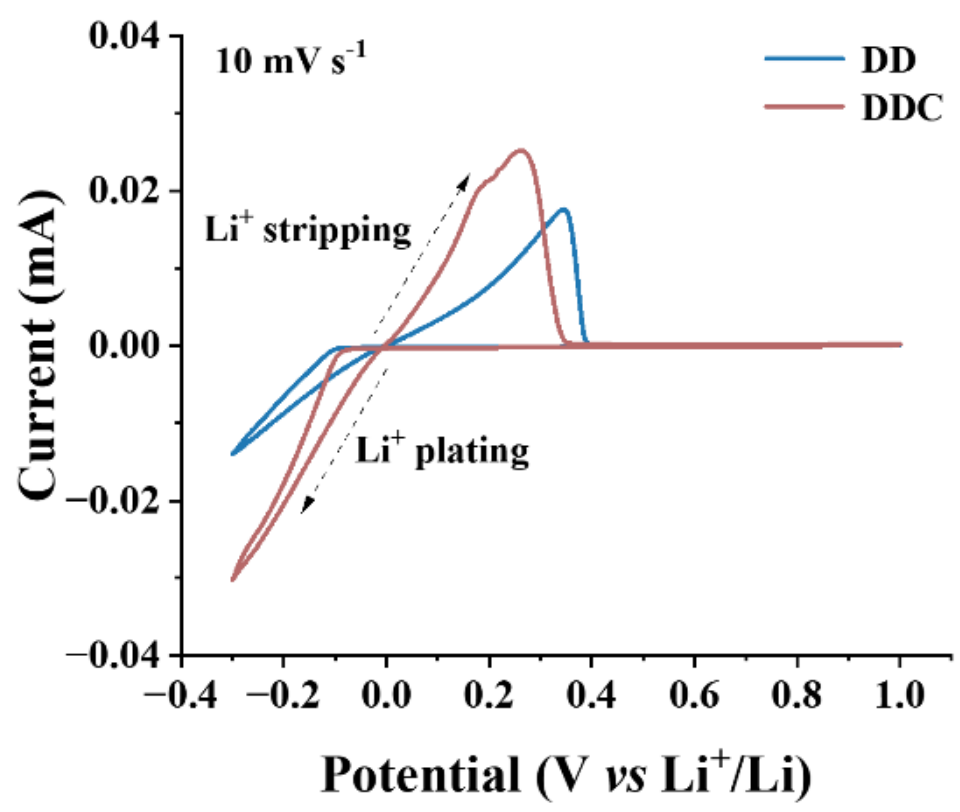

Fig. R6 | CV curves of asymmetric Li||SS steel battery at 10 mV s^{-1}.

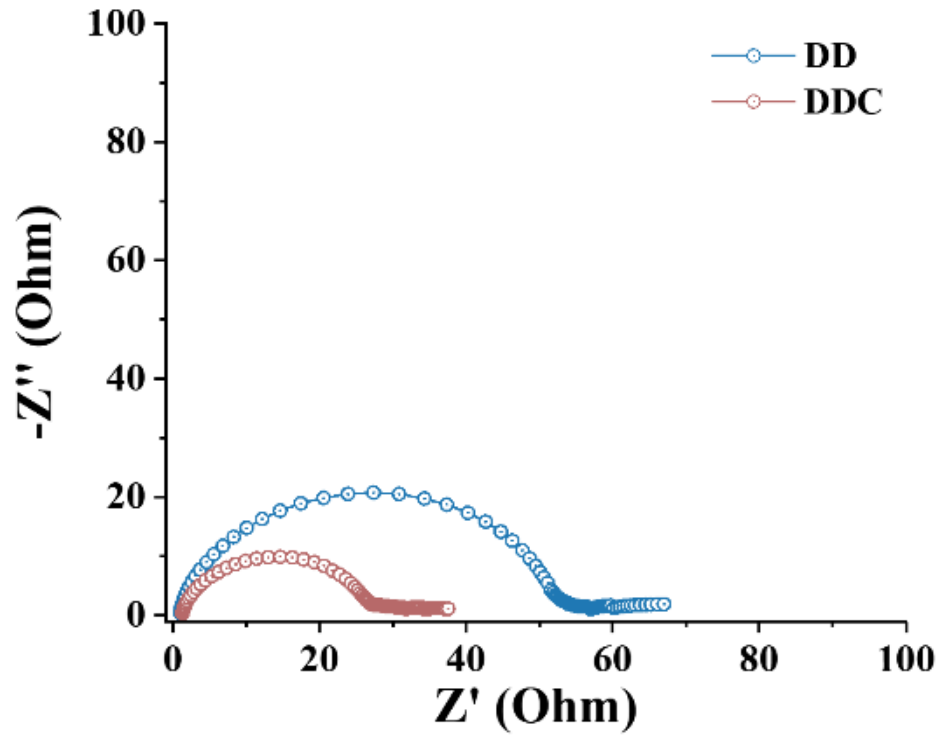

Fig. R7 | EIS patterns of symmetric Li||Li batteries with DD and DDC electrolytes.

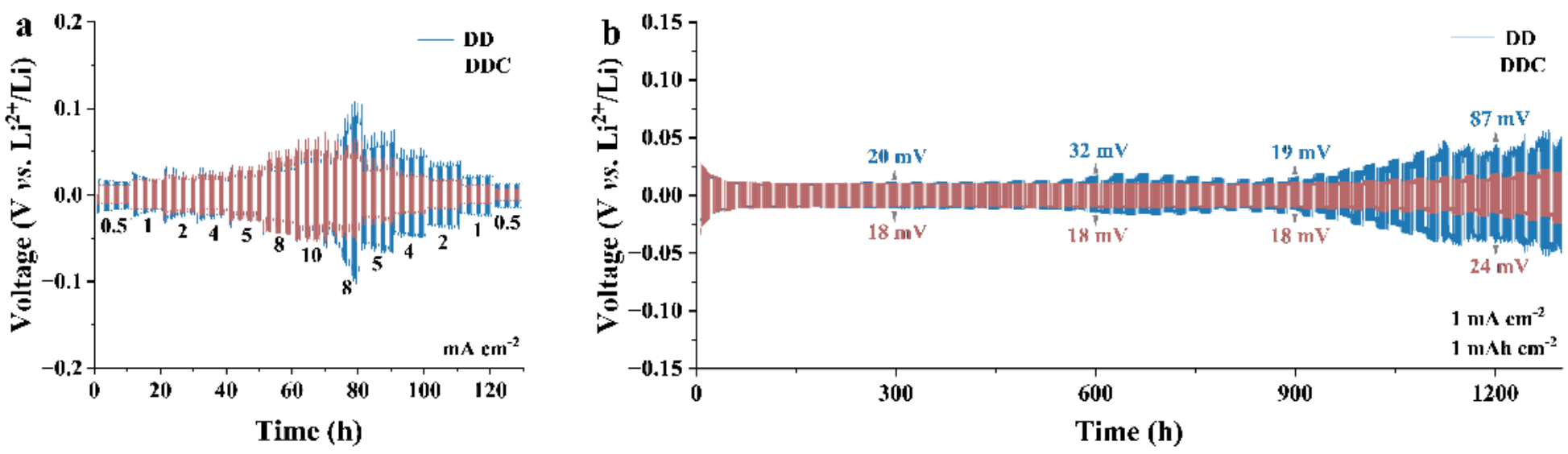

Fig. R8 | (a) Rate capability of a symmetric Li||Li battery with DD and DDC electrolytes. **(b)** Long-term cycling performance of a symmetric Li||Li battery with DD and DDC electrolyte at 1 mA cm^{-2} and 1 mAh cm^{-2}.

Please check the newly added **Figs. 3g-i** on **Page 11** and the newly added discussion on **Page 10** in the main text, and **Fig. S17** on **Page 10** in the supplementary information.

Comment 5: *The logical flow and data presentation related to the scan rate dependence and b-value analysis in Figures S11 and 3a require improvement. The authors are advised to reorganize the descriptions and sequence of these figures to enhance logical coherence and readability.*

Response: Thank you for your constructive comment and valuable suggestion. In response, we have revised the layout of the scan-rate-dependent CV and *b*-value analyses and updated the related descriptions to improve clarity and logical flow.

Please check the improved discussion on **Page 12** in the main text.

Comment 6: *The manuscript presents cycling stability data for both Li||EV(ClO_4)$_2$ and Li||EVBr_2 batteries, but the vertical axis labeling is inconsistent between these figures. It is recommended to unify the axis labels and units to ensure data consistency and clarity of presentation.*

Response: Thank you for your constructive comments and valuable suggestions. In response, we carefully examined all cycling curves and standardized their vertical axes to ensure consistent labels and units.

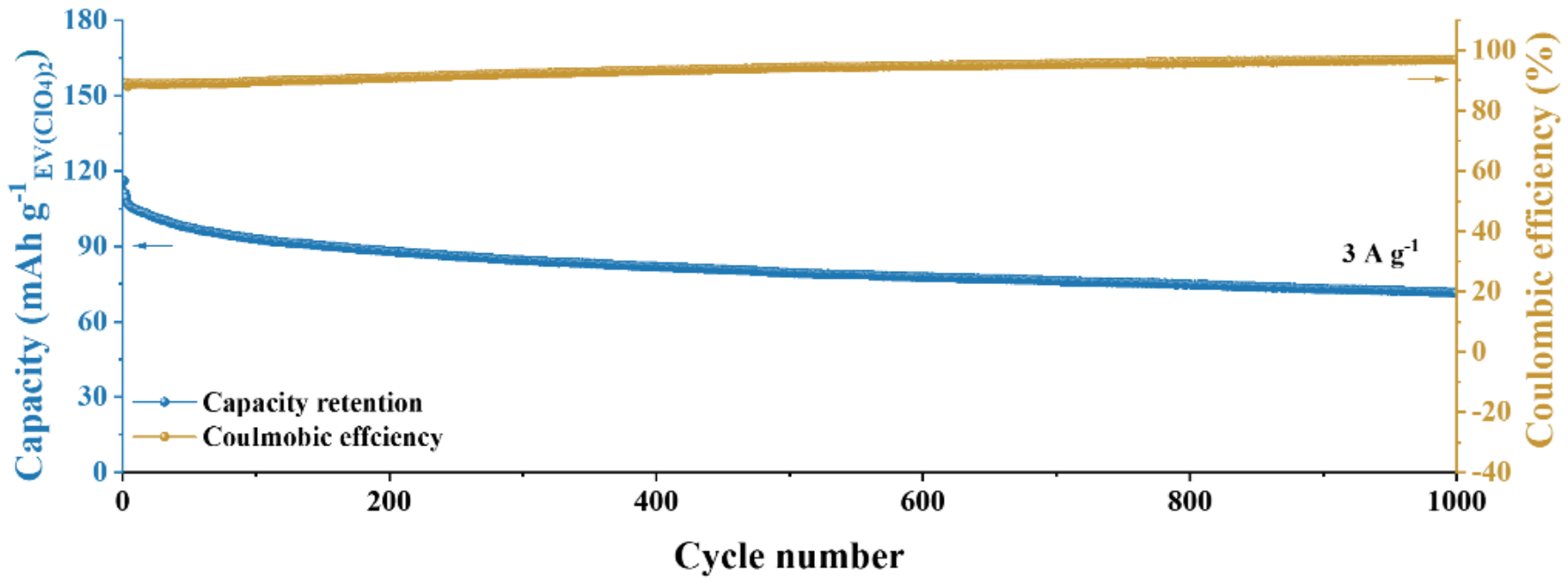

Fig. R9 | Long-term cycling performance of Li||EV(ClO_4)$_2$ battery in DD electrolyte at 3 A g^{-1}.

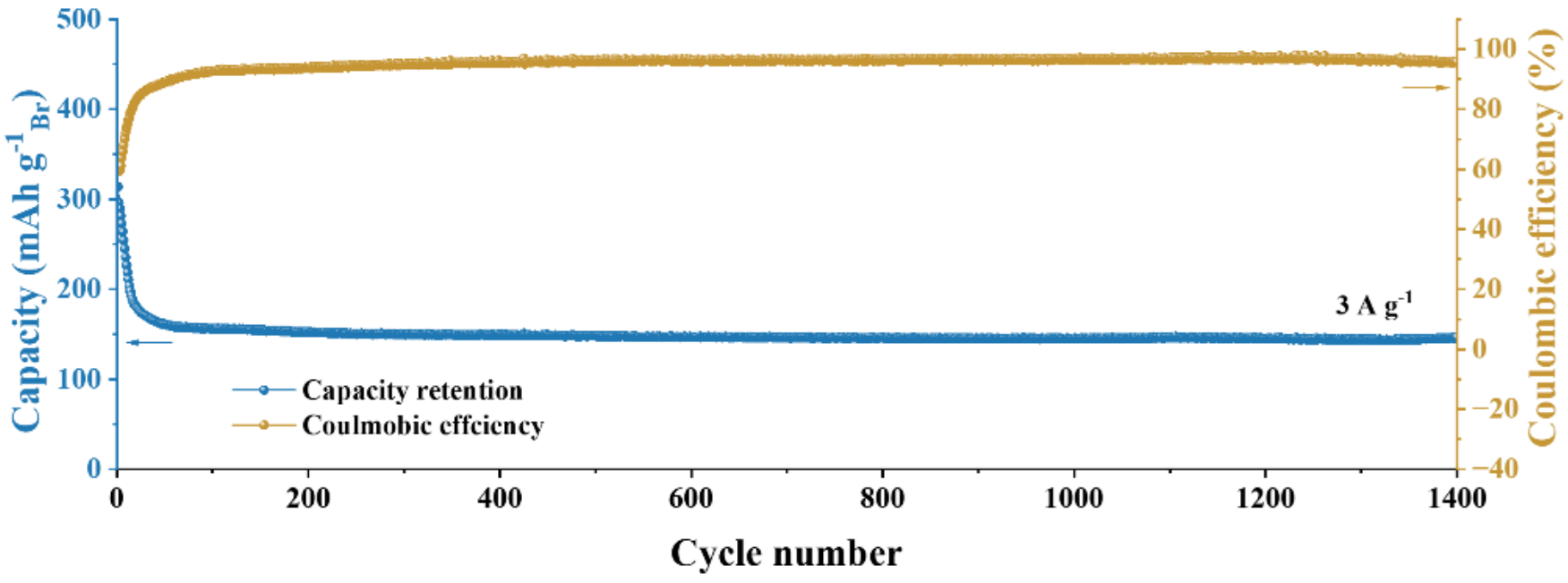

Fig. R10 | Long-term cycling performance of Li||$EVBr_2$ battery in DDC electrolyte at 3.0 A g^{-1}.

Please check the modified **Fig. 4g** on **Page 14** in the main text, and the modified **Fig. S9** on **Page 7** in the supplementary information.

Comment 7: *Comparison with other battery system in the introduction is recommended for balance. such as eScience,2025,5,4,100331; Energy Mater 2024;4:400024; Energy Mater. 2025, 5, 500105.*

Response: Thank you for your constructive comments and valuable suggestions. The recommended references have been incorporated and discussed in the revised manuscript.

Please check the newly added **references 4, 10, and 33** on **Pages 22-23 and 25** in the main text.

To Reviewer #2:

This article reports on the coupling of an ethyl viologen (EV) ligand with Br, and the introduction of Cl^- to achieve the oxidation of Br^- to Br^+, realizing high-valent halogen conversion. However, the key EV ligand employed by the authors has already been reported in zinc and magnesium batteries (Adv. Energy Mater. 2019, 9, 1903002). Furthermore, the use of Cl^- to modulate the bromine reaction pathway is not novel either (Sci. Adv. 2024,10, eadl0587). Consequently, the innovation of this work is lacking. Moreover, the evaluation of the core electrochemical mechanisms lacks in-

depth interpretation, especially regarding whether the ether-based electrolyte undergoes continuous oxidative decomposition at high voltage, and the formation of the key species Br-Cl lacks clear supporting evidence, casting doubt on the main conclusions. Specific issues are as follows:

Response: We sincerely thank you for reviewing our manuscript and providing such in-depth and constructive comments. Your precise grasp of the research direction and insightful identification of key scientific issues have been highly inspiring. We fully agree that innovation and depth of mechanistic interpretation are central to high-level research. After a thorough review, we fully acknowledge the foundational value of *Adv. Energy Mater. 2019, 9, 1903002* and *Sci. Adv. 2024, 10, eadl0587*. However, the core contribution of our work lies in transcending the individual scope of these studies to establish, for the first time, a synergistic electrochemical paradigm.

The *Adv. Energy Mater. 2019, 9, 1903002* study demonstrated the potential of EV^{2+} as a host material in magnesium batteries coupled with I^- to achieve four-electron storage. Its mechanism is essentially a simple functional superposition of "cation intercalation" and "anion oxidation." Constrained by the narrow electrochemical window of the magnesium electrolyte, its operating voltage is below 3.0 V. In this system, I^- serves only as an additional capacity contributor and suffers from the polyiodide shuttle effect. At high voltages, it readily approaches the electrolyte decomposition limit, resulting in low Coulombic efficiency.

The *Sci. Adv. 2024, 10, eadl0587* study insightfully revealed the modulating role of Cl^- in the bromine reaction pathway. However, the cation in that system is electrochemically inert, confining the system to a single-function conversion reaction. This fails to harness the energy storage potential of the cationic moiety, resulting in a relatively low gravimetric/volumetric energy density.

The fundamental breakthrough of our work lies in the unified design integrating an electrochemically active cation with Cl^--stabilized high-valent anion redox chemistry. By synergistically combining the reversible two-electron intercalation of EV^{2+} with the Cl^--mediated four-electron $Br^-/Br^0/Br^+$ conversion reaction, we have constructed a unique "dual-center" synergistic platform, namely a coupling of an

"active intercalation center" and a "high-valent conversion center." This design not only unlocks a six-electron transfer mechanism that far exceeds a simple additive effect, but more importantly, resolves the long-standing challenge of Br^+ instability through the coordination locking effect of Cl^- and the effective adsorption by EV^{2+}. Benefiting from this synergistic mechanism, our work achieves a high discharge plateau of 3.7 V, a rarity in conversion-type systems, marking a leap from "low-voltage multi-electron" to "high-voltage multi-electron" energy storage technology. Therefore, this work is not merely an optimization of existing systems, but rather redefines the design principles for high-energy halogen batteries, advancing the research paradigm from "single-center redox" to a new dimension of "multi-center synergistic electrochemistry."

We acknowledge that, in our initial submission, some issues may have arisen and obscured the innovative aspects of the study because key experimental data and mechanistic discussions were not presented with sufficient detail or clarity. To eliminate all your doubts, we have systematically augmented the revised manuscript with substantial additional experimental evidence and theoretical analysis, and have not made any evasive handling. We once again extend our sincere gratitude for your rigorous and insightful review, which has greatly helped us in refining this study. We kindly invite you to review the revised manuscript and hope it meets with your approval.

Comment 1: *EV is claimed to have coordination capability towards bromine in different states [Br], but no detailed explanation is provided. The strength of this strong coordination capability towards halogens in different valence and coordination states, the potential competitive relationships, and the proportion of free halogens need to be demonstrated.*

Response: Thank you for your constructive comments and valuable suggestions. Following your suggestion, we performed additional calculations on the adsorption energies of EV^{2+} toward bromine species in different oxidation states. The results show that the adsorption energies of EV^{2+} for Br^- and Br^0 are -7.39 eV and -0.46 eV, respectively (**Fig. R11**). As $EVBr_2$ functions as the cathode-active material, free Br^- species are not expected to be present in the system under operating conditions.

Given the introduction of LiCl into the electrolyte, we further quantitatively analyzed the concentration of free Cl^- species. Automated potentiometric titration reveals that the Cl^- concentration in the pristine electrolyte is 0.1 M. After electrochemical cycling, the Cl^- concentration remains unchanged in the uncharged state, while a slight decrease to 0.06 M is observed in the fully charged state.

Combined with the DFT results shown in **Fig. S12**, the decrease in Cl^- concentration can be attributed to two factors: (i) partial adsorption of Cl^- onto the EV^{2+} framework during charging, and (ii) participation of Cl^- in the Br^0-to-Br^+ conversion process. Together, these results elucidate the dynamic consumption and functional role of Cl^- during the electrochemical process.

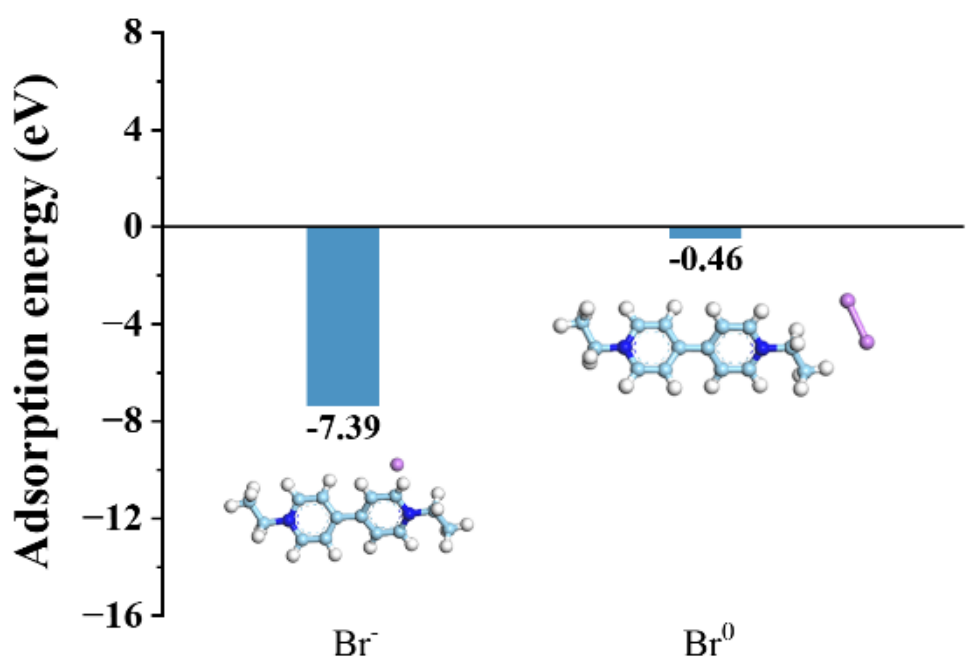

Fig. R11 | Adsorption energies of EV^{2+} for Br^- and Br^0.

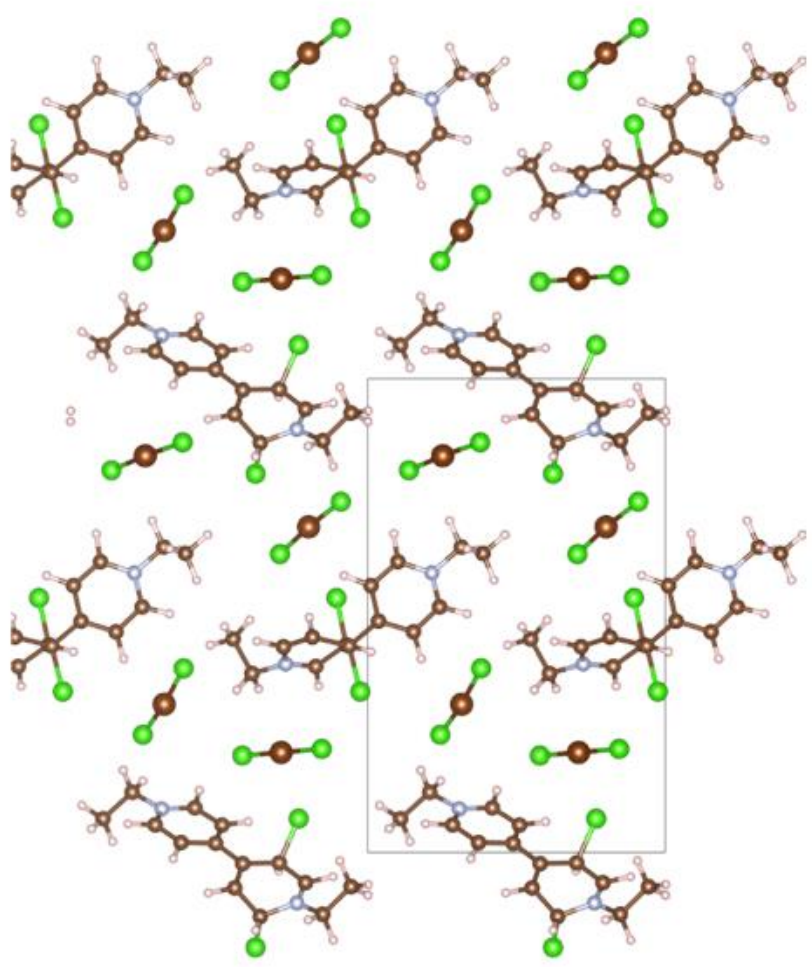

Fig. R12 | Crystal structure model of $EV^{2+}2Cl^-2(BrCl_2)$.

Please check the newly added **Fig. S31** on **Page 16** in the supplementary information, and the newly added discussion on **Page 13** in the main text.

Comment 2: *The authors attribute capacity decay to minor side reactions. However, the reaction process in this system is highly complex. The investigation into capacity fade should analyze each electrochemical process individually. The cycling efficiency for each electrochemical reaction should be provided.*

Response: Thank you for your constructive comments and valuable suggestions. To elucidate the cycling stability of individual electrochemical processes, we included additional long-term cycling data at 5 A g^{-1} and systematically analyzed the capacity decay of each process based on charge-discharge profiles and corresponding *dQ/dV* curves at different cycle numbers (**Fig. R13**). The first cycle displays four well-defined voltage plateaus, corresponding to the $EV^{2+}/EV^{+\bullet}$, $EV^{+\bullet}/EV^{0}$, Br^{0}/Br^{-}, and Br^{+}/Br^{0} redox processes. These plateaus remain clearly discernible at the 10th cycle with only a slight capacity decrease. By the 50th cycle, the plateaus associated with the $EV^{2+}/EV^{+\bullet}/EV^{0}$ processes show pronounced attenuation, and after 1000 cycles they largely disappear (**Fig. R14**). The evolution of the *dQ/dV* curves exhibits a similar trend: four well-defined redox peaks appear in the initial cycle and remain evident at the 10th cycle; the peaks associated with the $EV^{2+}/EV^{+\bullet}/EV^{0}$ processes diminish noticeably from the 50th cycle onward and are nearly absent after 1000 cycles (**Fig. R15**).

Quantitative analysis of the integrated *dQ/dV* features reveals that after 1000 cycles, the capacity decay ratios associated with the individual redox processes are approximately 25% for Br^{+}/Br^{0}, 23% for Br^{0}/Br^{-}, 83% for $EV^{2+}/EV^{+\bullet}$, and 99% for $EV^{+\bullet}/EV^{0}$. These results show that the capacity fade is predominantly due to the degradation of the $EV^{2+}/EV^{+\bullet}/EV^{0}$ processes. This degradation is likely caused by material volume expansion and structural stress from repeated Li^{+} insertion and extraction.

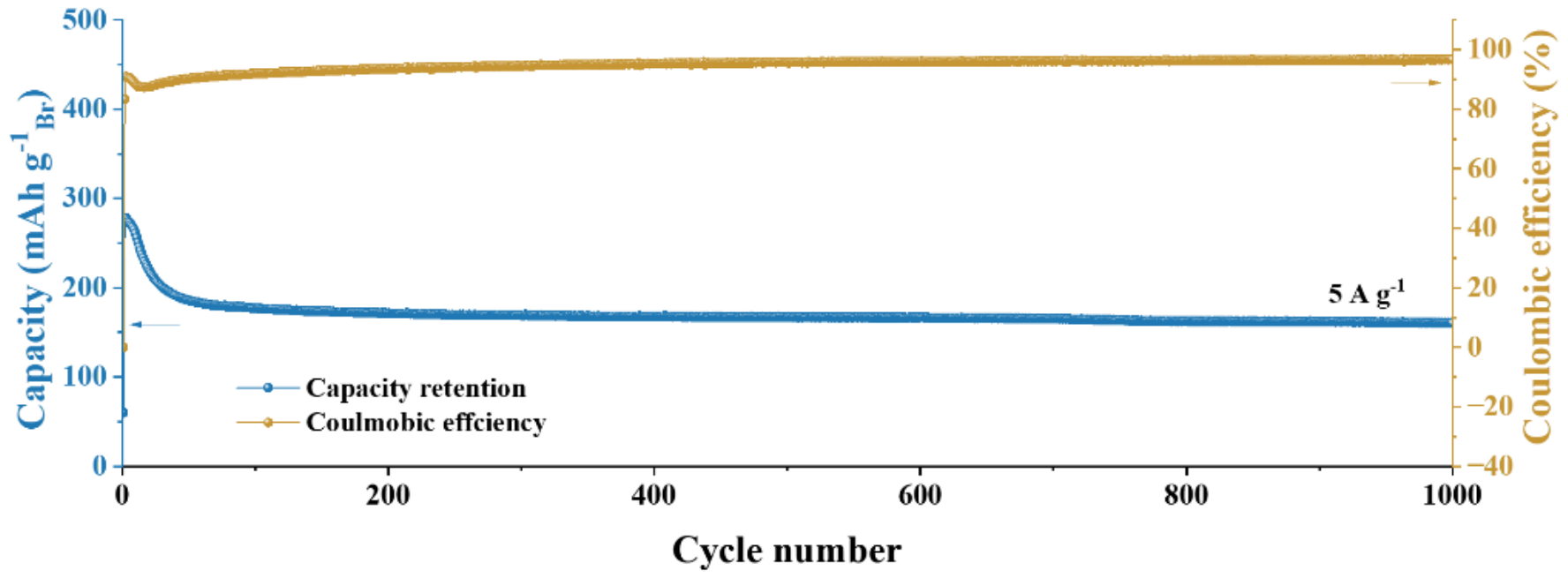

Fig. R13 | Long-term cycling performance of Li||$EVBr_2$ battery in DDC electrolyte at 5.0 A g^{-1}.

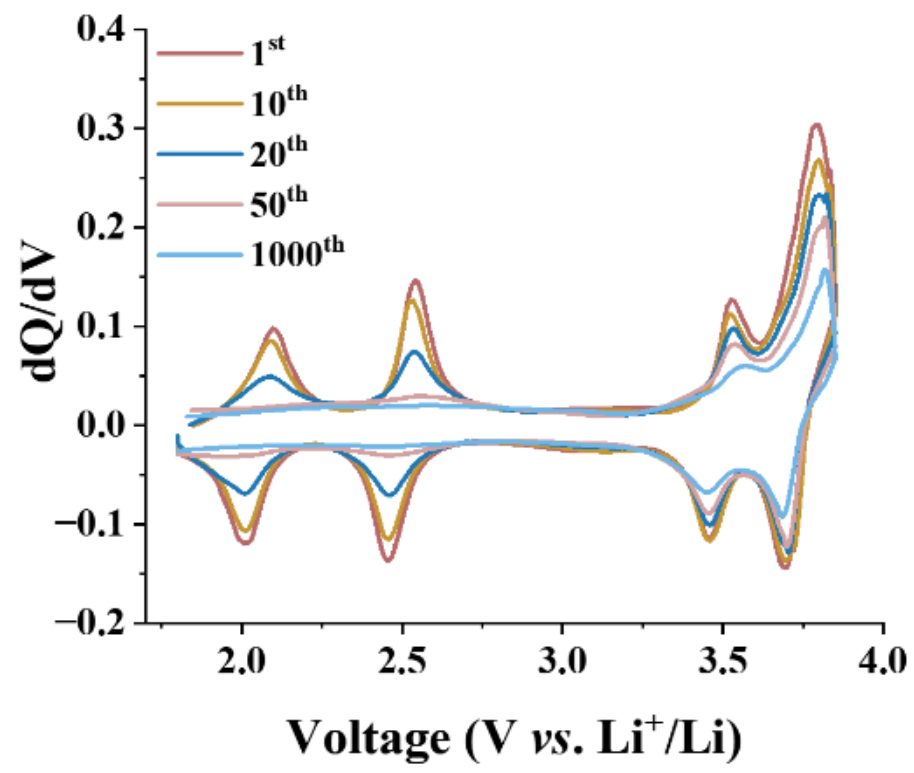

Fig. R14 | The *dQ/dV* curves of Li||$EVBr_2$ battery in DDC electrolyte at 5.0 A g^{-1}.

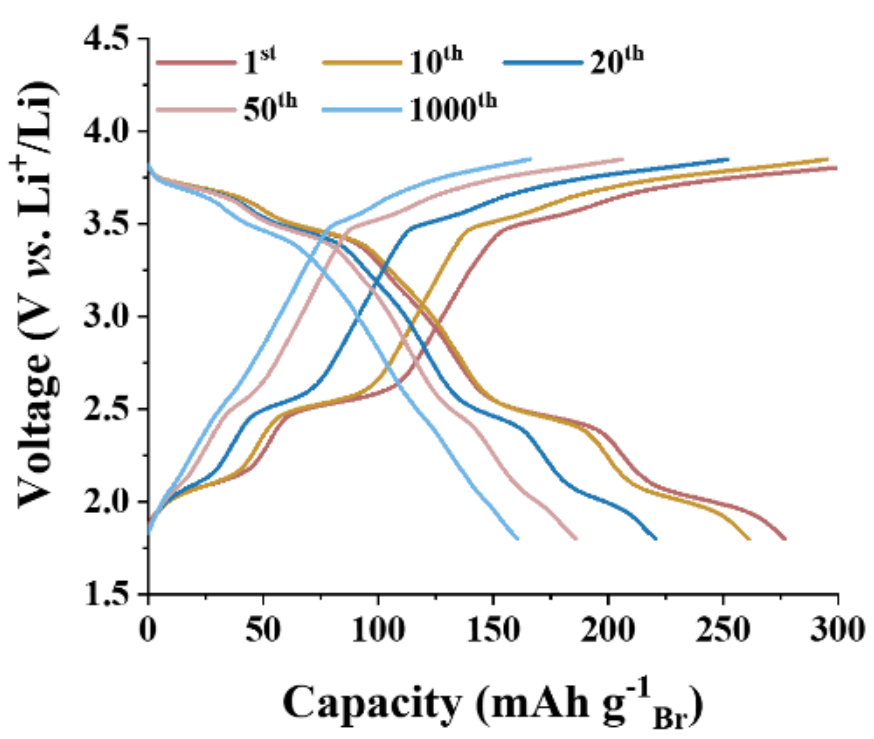

Fig. R15 | GCD curve of Li||$EVBr_2$ battery in DDC electrolyte at 5.0 A g^{-1}.

Please check the newly added **Fig. 4h** on **Page 14** and the newly added discussion on **Page 13** in the main text, and newly added **Figs. S29-30** on **Page 15** in the supplementary information.

Comment 3: *The Br^0/Br^+ redox reaction occurs at a high voltage of 3.85 V, which exceeds the typical anodic stability limit of conventional ether-based electrolytes (DOL/DME). The authors need to provide evidence, such as* ***the oxidative current in CV*** *or gas chromatography-mass spectrometry data, to prove the electrolyte's stability at this high voltage or the formation of an effective Cathode Electrolyte Interphase (CEI).*

Response: Thank you for your constructive comments and valuable suggestions. In response, we conducted a series of complementary experiments to comprehensively evaluate the stability of the electrolyte under high-voltage operation. First, LSV curves of Li||SS (stainless steel) batteries show no significant anodic current up to 4.0 V in either the DD or DDC electrolyte, indicating that both electrolytes provide sufficiently wide electrochemical stability windows to accommodate the operating potential of the Br^0/Br^+ redox couple (**Fig. R5**). Second, to examine whether the DDC electrolyte undergoes decomposition and forms an effective cathode electrolyte interphase (CEI) in a practical Li||$EVBr_2$ battery, transmission electron microscopy (TEM) was performed on the cycled cathode. A uniform interfacial layer with a thickness of approximately 3.5 nm was observed on the electrode surface, indicating that limited and controlled electrolyte decomposition occurs during the initial cycles, leading to the *in-situ* formation of a compact CEI (**Fig. R16**). Finally, the operating voltage window of the Li||$EVBr_2$ battery was extended to 4.0 V to further probe the electrolyte behavior at high potentials. The CV curve revealed a reversible redox couple at approximately 4.0/3.85 V, which is assigned to the Cl^-/Cl^0 reaction rather than to irreversible electrolyte decomposition (**Fig. R4**). This observation indicates that, after the initial CEI formation, the electrolyte remains stable across the entire operating window, an outcome that is likely facilitated by the protective nature of the CEI.

In summary, through LSV, TEM, and extended-window CV studies, we provide multi-faceted evidence supporting the high-voltage stability of the DDC electrolyte and the *in-situ* formation of a protective CEI.

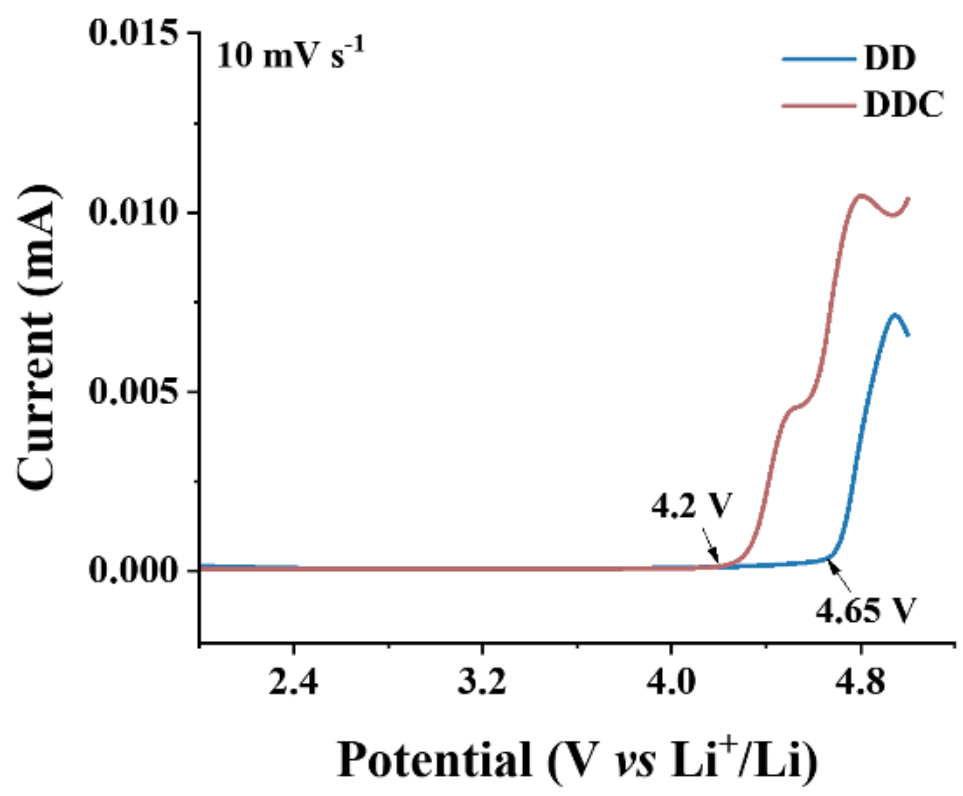

Fig. R5 | LSV curves of asymmetric Li||SS battery at 10 mV s^{-1}.

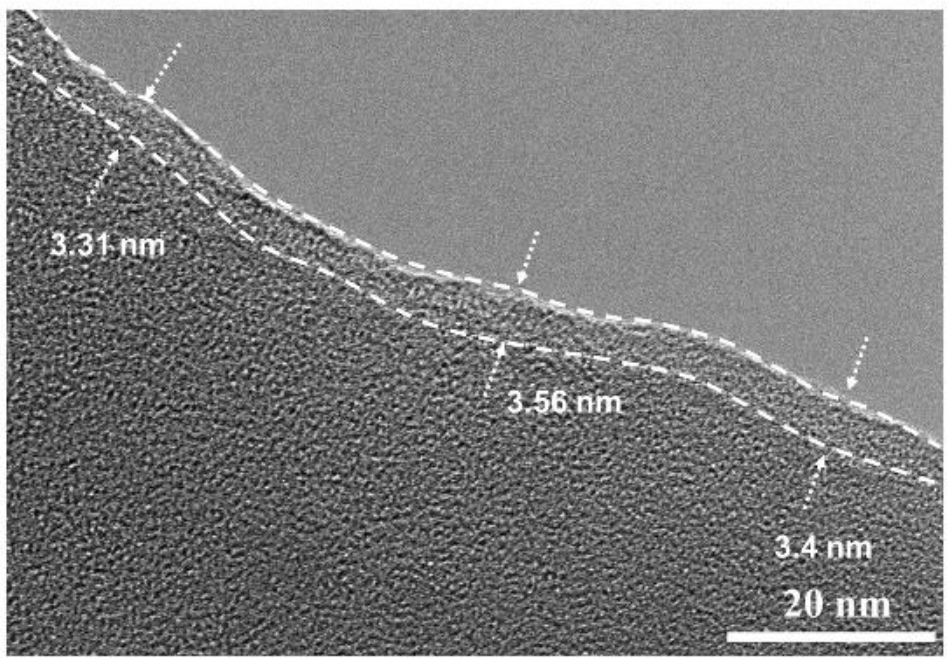

Fig. R16 | TEM image of the CEI layer.

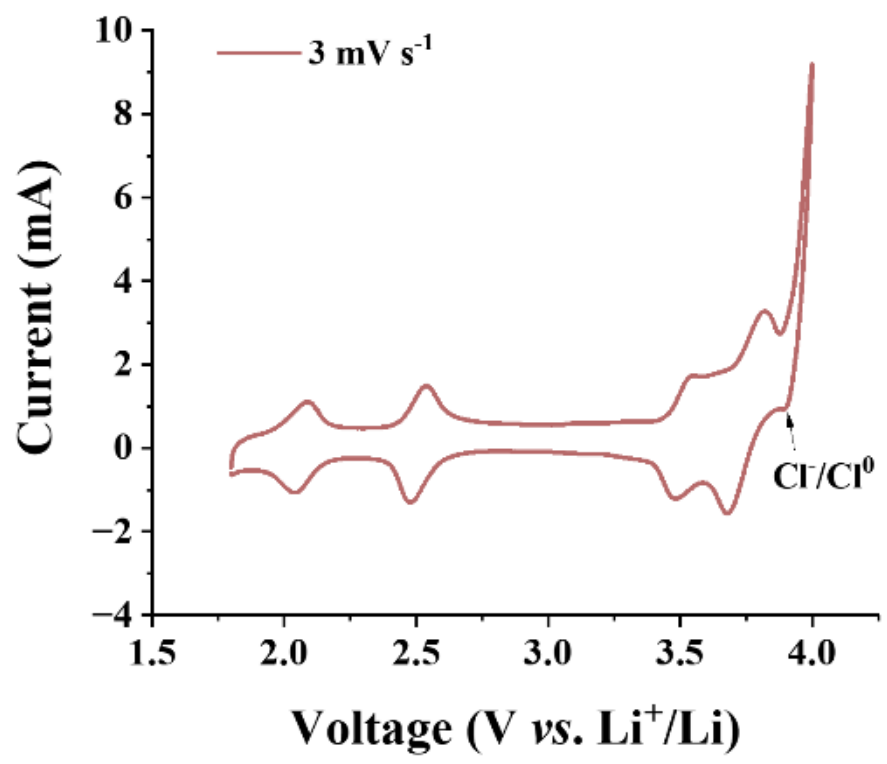

Fig. R4 | CV curve of Li||EVBr2 battery in DDC electrolyte.

Please check the newly added **Fig. 3g** on **Page 11** and the newly added discussion on **Pages 8 and 10-11** in the main text, and newly added **Fig. S15a** and **S23** on **Pages 10 and 12** in the supplementary information.

Comment 4: *The solubility of the EV-based active material needs to be assessed, along with an evaluation of its shuttle effect and reactivity towards lithium metal.*

Response: Thank you for your constructive comments and valuable suggestions. To assess the dissolution behavior of $EVBr_2$ in the electrolyte, we performed a soaking test. Specifically, 2 mg of $EVBr_2$ powder and a freshly prepared $EVBr_2$ electrode were each immersed in 2 mL of DD electrolyte and allowed to stand for 6 hours (**Figs. R17-18**). The DD electrolyte was selected to avoid potential Cl^- interference during quantitative analysis. Visual inspection revealed no noticeable color change in the electrolyte after soaking, and the $EVBr_2$ solid particles remained clearly visible.

Subsequently, the clarified supernatant was quantitatively analyzed using automated potentiometric titration. The results indicate that the solubility of $EVBr_2$ powder in the DD electrolyte is 0.002 M, whereas that of the $EVBr_2$ electrode is further reduced to 0.001 M. These results indicate that, although $EVBr_2$ exhibits limited intrinsic solubility, its dissolution is effectively suppressed after electrode fabrication due to encapsulation by the conductive carbon and polymer binder.

To evaluate bromine shuttle and anode compatibility, Li||$EVBr_2$ batteries were cycled 10 times and disassembled at both the fully charged (3.85 V) and discharged (1.8 V) states for post-mortem analysis. The lithium metal anodes were examined by SEM and EDS. SEM images show compact and uniform lithium morphologies, consistent with reversible deposition/stripping and free of dendrites or large agglomerates (**Fig. R19**). Critically, EDS analysis detected no bromine signal on the lithium surface (**Fig. R20**). Together, these analyses demonstrate that the $EVBr_2$ cathode effectively suppresses bromine shuttle and maintains good compatibility with the lithium metal anode, without inducing corrosion or parasitic reactions.

Overall, $EVBr_2$ exhibits low solubility in the electrode state and negligible shuttle behavior. Even in the presence of trace dissolution, the dissolved species can be reversibly reincorporated into the cathode during charging, thereby rendering their impact on the overall electrochemical performance negligible.

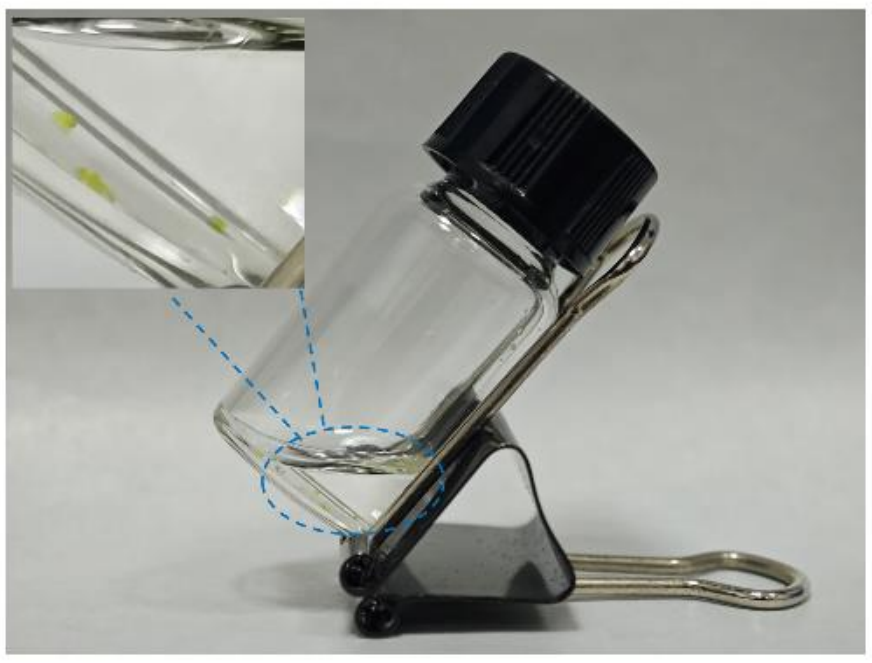

Fig. R17 | Photograph of $EVBr_2$ immersed in the DD electrolyte.

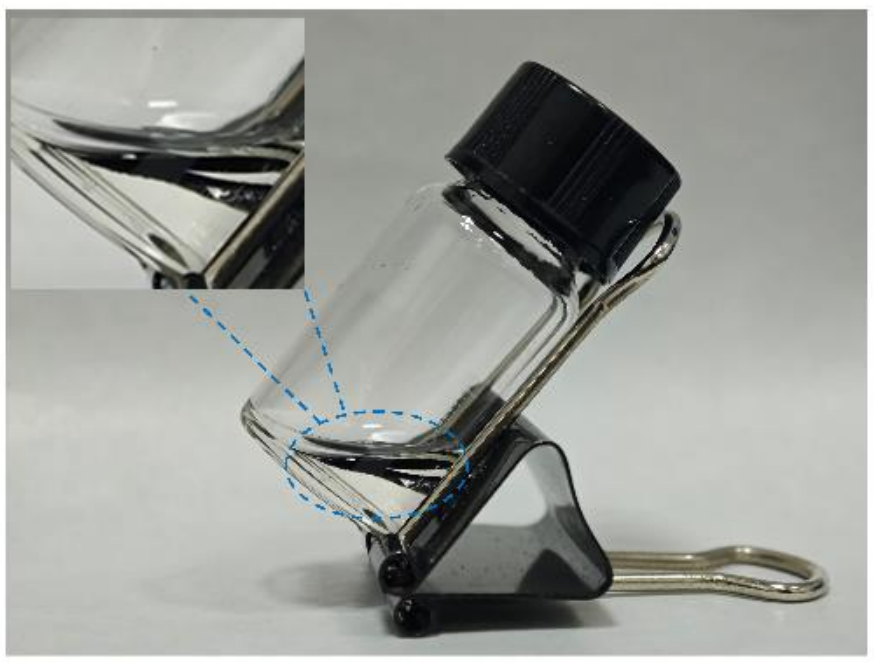

Fig. R18 | Photograph of $EVBr_2$ electrode immersed in the DD electrolyte.

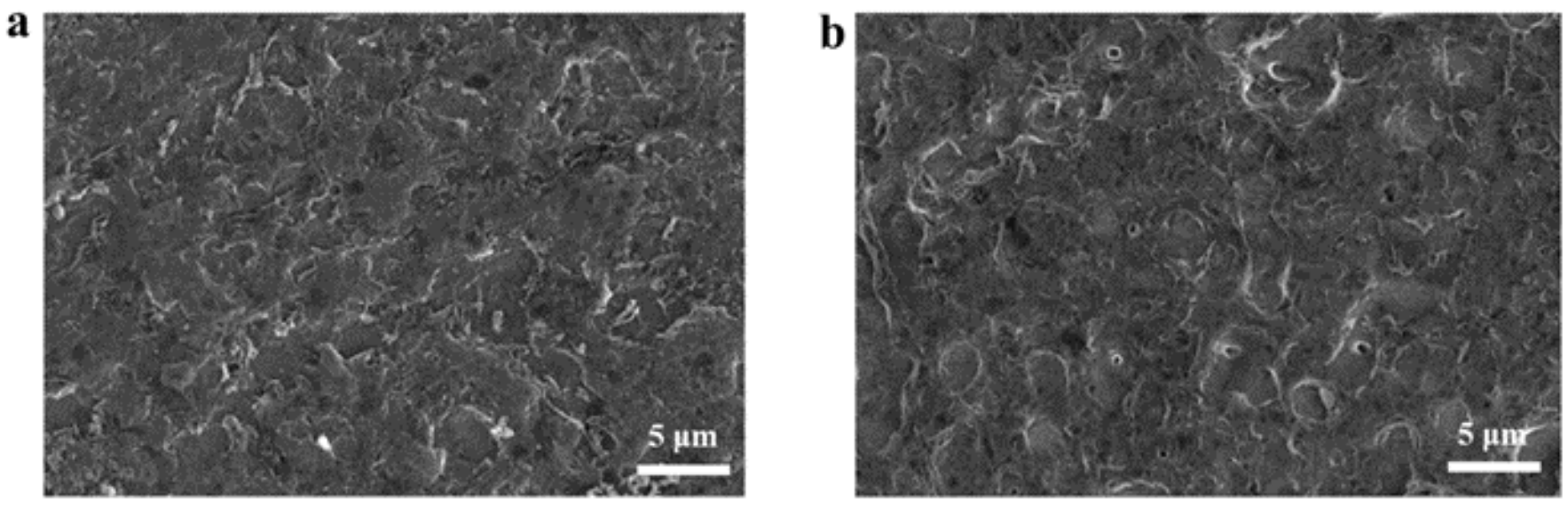

Fig. R19 | (a) SEM image of lithium metal in the charged to 3.85 V. **(b)** SEM image of lithium metal in the discharged to 1.8 V.

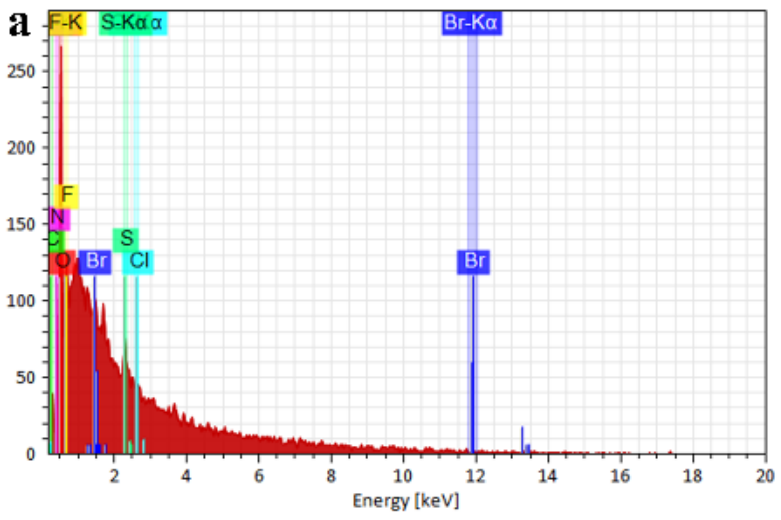

Element	Mass ratio (wt%)
C	26.06
N	0.00
O	69.92
F	0.00
S	3.20
Cl	0.82
Br	0.00
Total	100.000

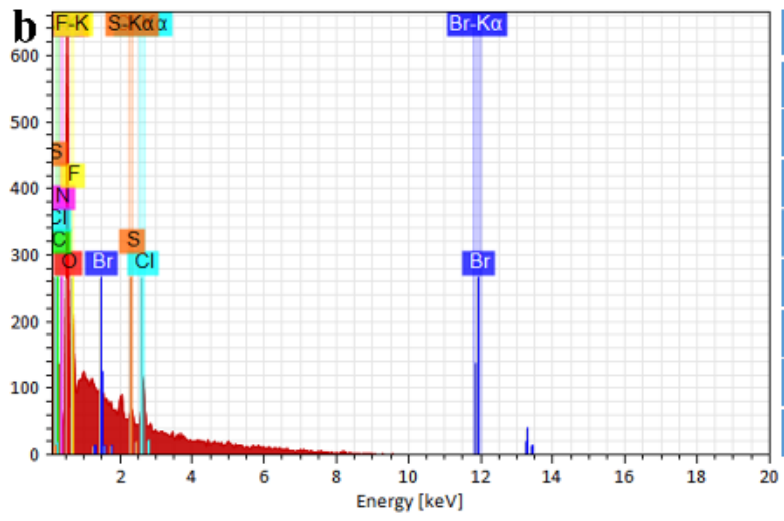

Element	Mass ratio (wt%)
C	19.26
N	0.00
O	51.47
F	25.42
S	0.72
Cl	3.13
Br	0.00
Total	100.000

Fig. R20 | (a) EDS analysis of lithium metal in the charged to 3.85 V. **(b)** EDS analysis of lithium metal in the discharged to 1.8 V.

Please check the newly added **Figs. S3-4** on **Pages 5-6** and **Figs. 18-19** on **Page 11** in the supplementary information, and the newly added discussion on **Pages 5-6 and 10** in the main text.

Comment 5: *The composition and morphological changes of the anode SEI need to be provided. Is the adsorption efficiency of the EV ligand for bromine 100%? If not, does it participate in and affect the composition of both the CEI and SEI?*

Response: Thank you for your constructive comments and valuable suggestions. Based on adsorption energy calculations and post-cycling EDS characterization of the lithium anode, although the adsorption/confinement efficiency of the EV ligand toward bromine species is not 100%, it remains sufficiently high (**Fig. R11** and **Figs. R19-20**). During the charging process, the small fraction of dissolved bromine species (e.g., polybromides) is preferentially oxidized to Br^0 or Br^+ and remains confined to the cathode side, where it participates in reversible redox reactions rather than undergoing long-range shuttling to the anode. Consequently, no detectable impact on the SEI composition is observed.

The interfacial layers were analyzed by TEM for morphology and XPS for

chemistry (**Fig. R16 and Figs. R21-23**). The SEI is primarily composed of electrolyte reduction products, including LiF, Li_3N (derived from $LiNO_3$), Li_2O/Li_2CO_3, and sulfur species (e.g., Li_2S) from $TFSI^-$ reduction (**Fig. R22**). Conversely, the CEI mainly contains LiF, nitrogen oxides (e.g., nitrates/nitrites) from $LiNO_3$ oxidation, and sulfur species (e.g., sulfates) from $TFSI^-$ oxidation at high voltage (**Fig. R23**). These results indicate that $EVBr_2$ does not measurably influence the formation or chemical composition of either the SEI or the CEI.

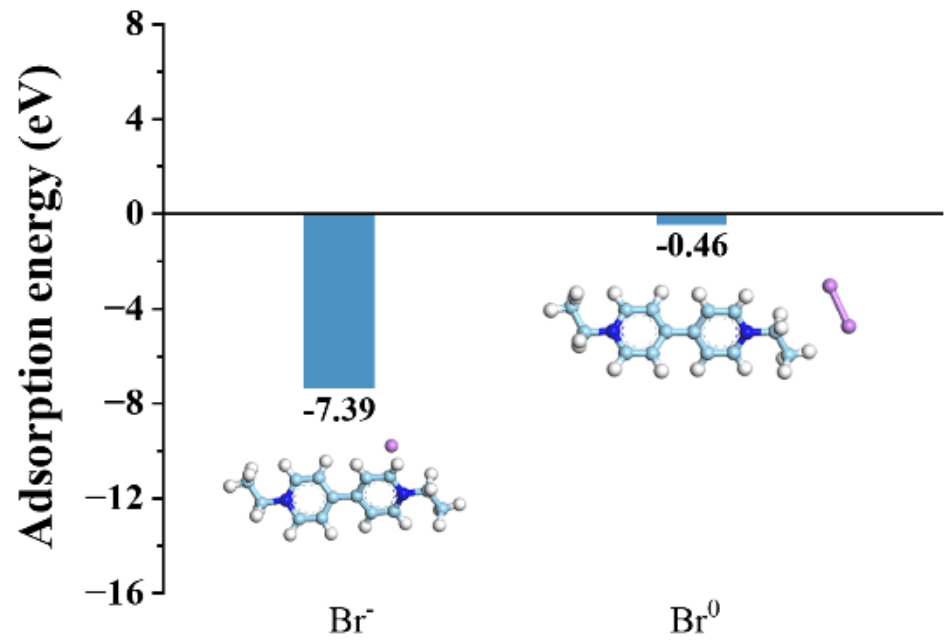

Fig. R11 | Adsorption energies of EV^{2+} for Br^- and Br^0.

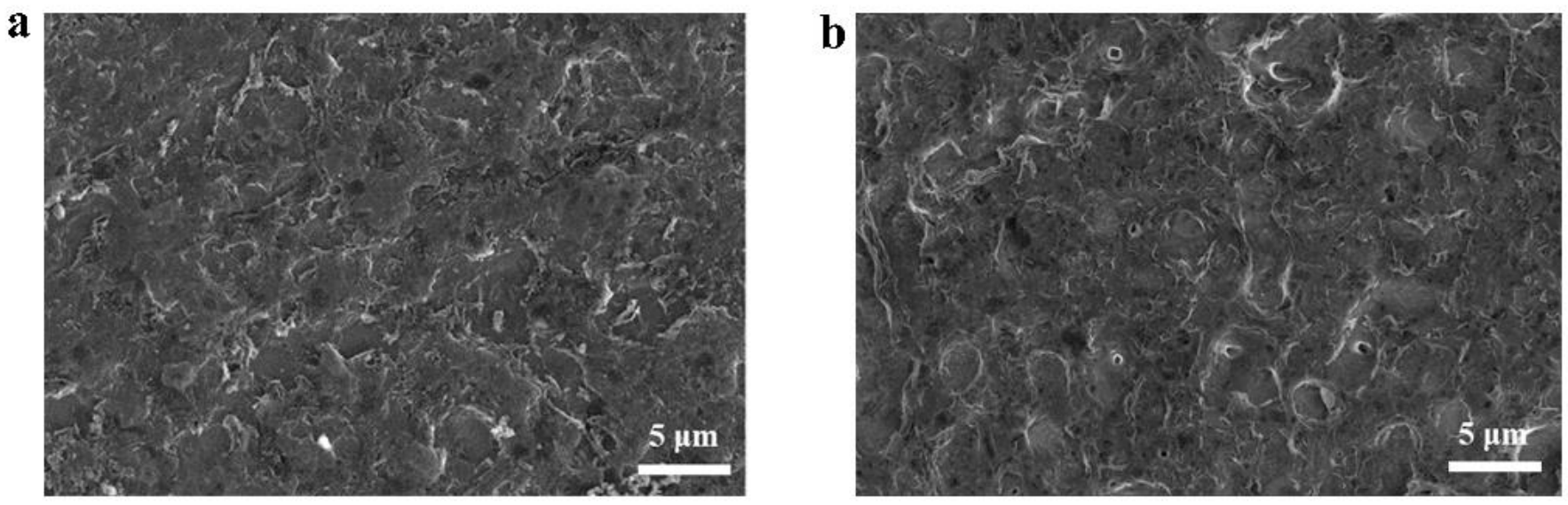

Fig. R19 | (a) SEM image of lithium metal in the charged to 3.85 V. **(b)** SEM image of lithium metal in the discharged to 1.8 V.

a

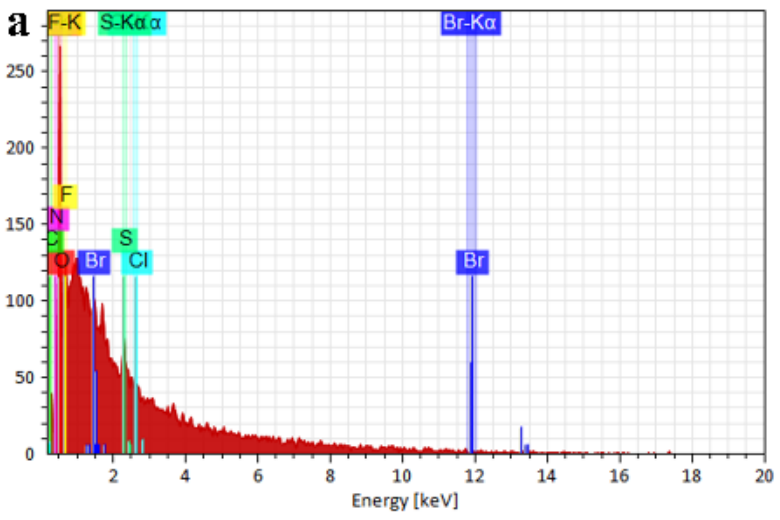

Element	Mass ratio (wt%)
C	26.06
N	0.00
O	69.92
F	0.00
S	3.20
Cl	0.82
Br	0.00
Total	100.000

b

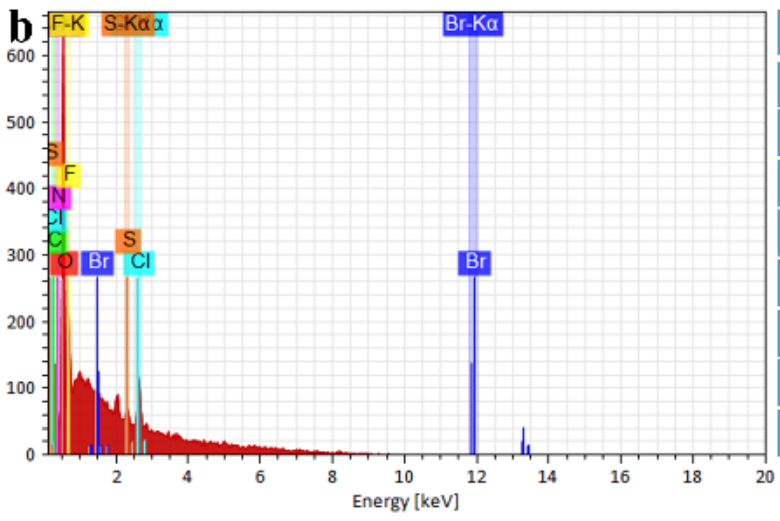

Element	Mass ratio (wt%)
C	19.26
N	0.00
O	51.47
F	25.42
S	0.72
Cl	3.13
Br	0.00
Total	100.000

Fig. R20 | (a) EDS analysis of lithium metal in the charged to 3.85 V. **(b)** EDS analysis of lithium metal in the discharged to 1.8 V.

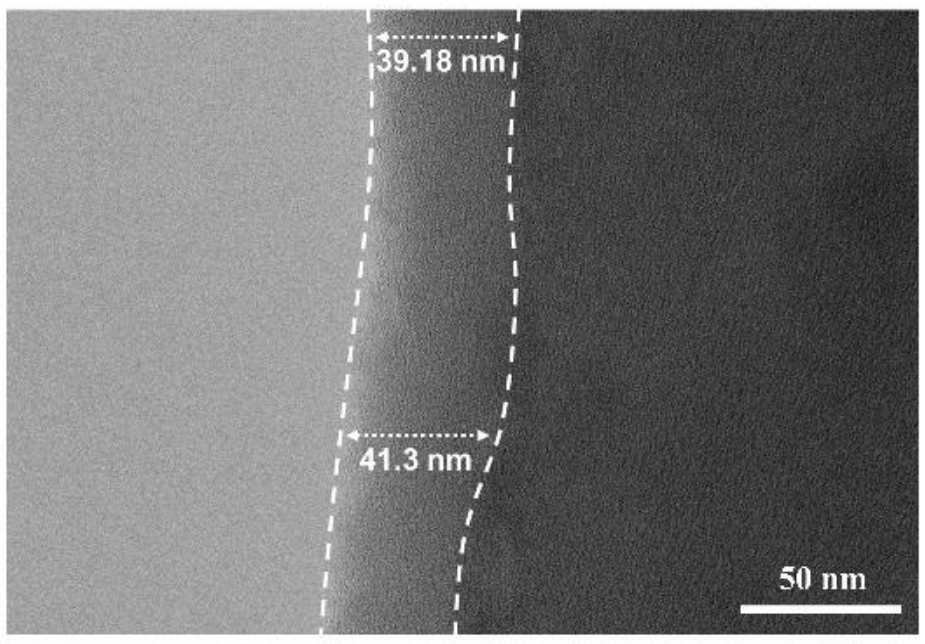

Fig. R21 | TEM image of the SEI layer.

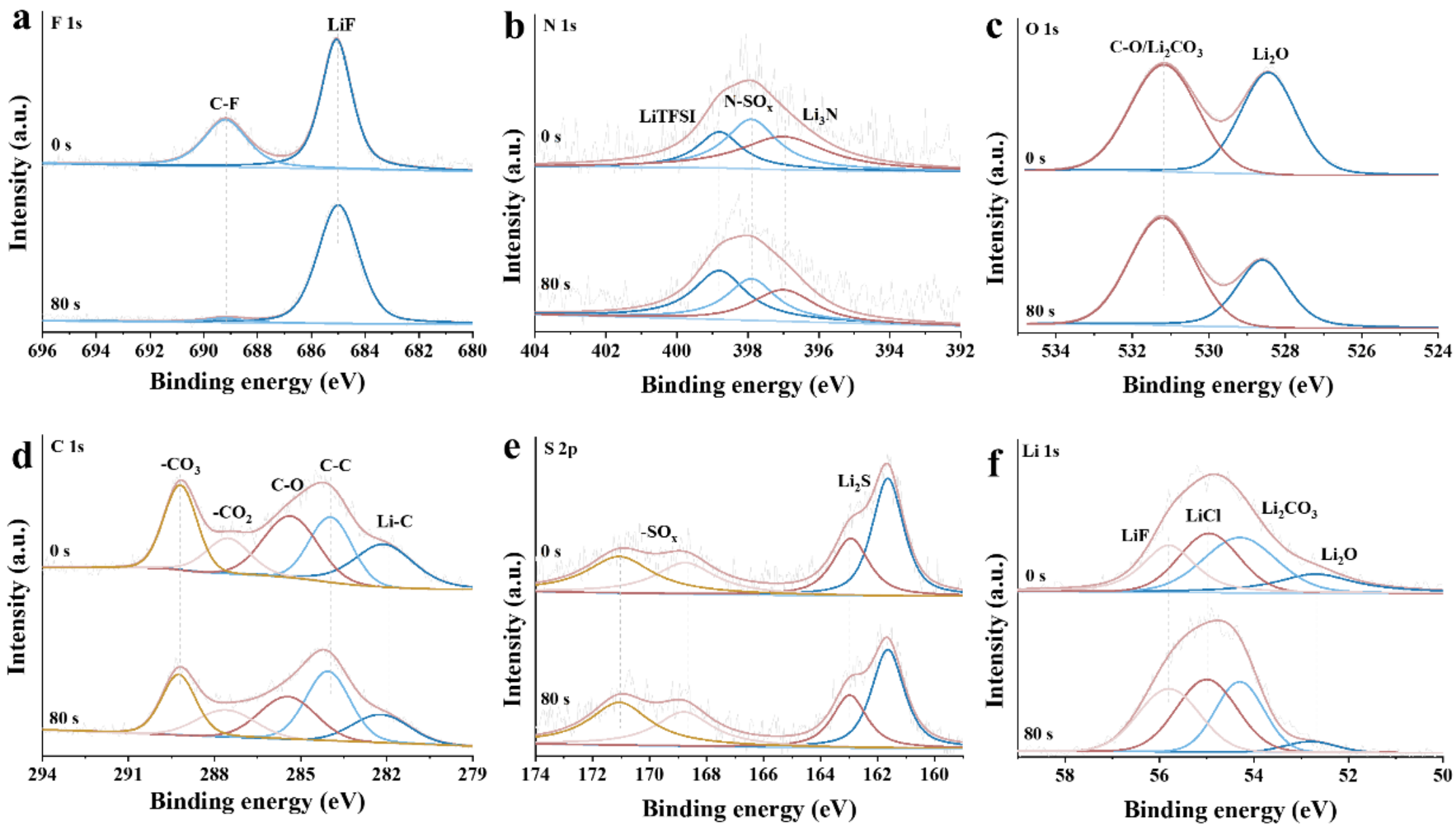

Fig. R22 | High-resolution XPS spectra of the SEI after cycling: F (a), N (b), O (c), C(d), S(e), and Li (f).

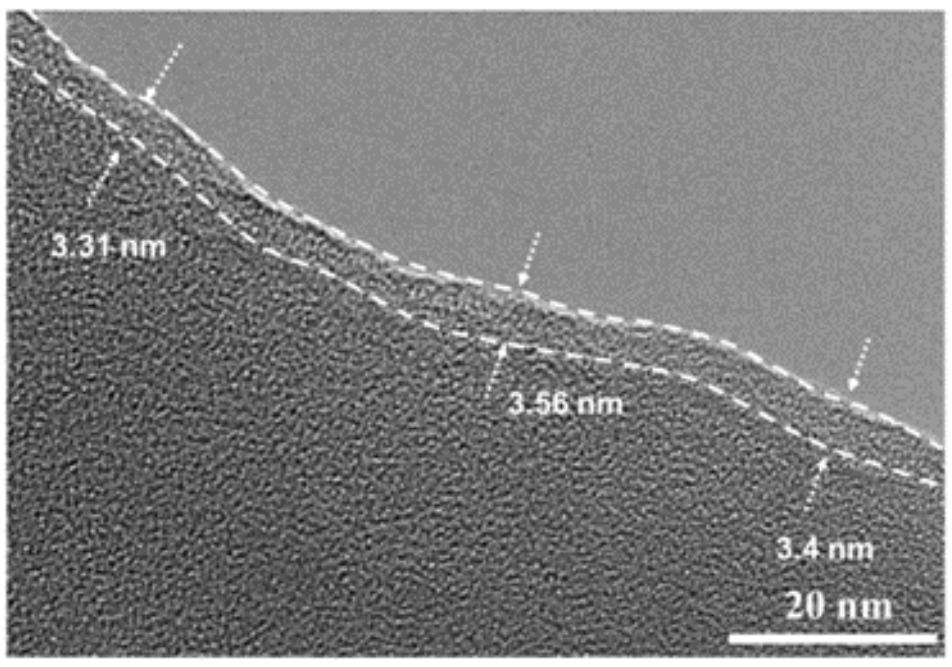

Fig. R16 | TEM image of the CEI layer.

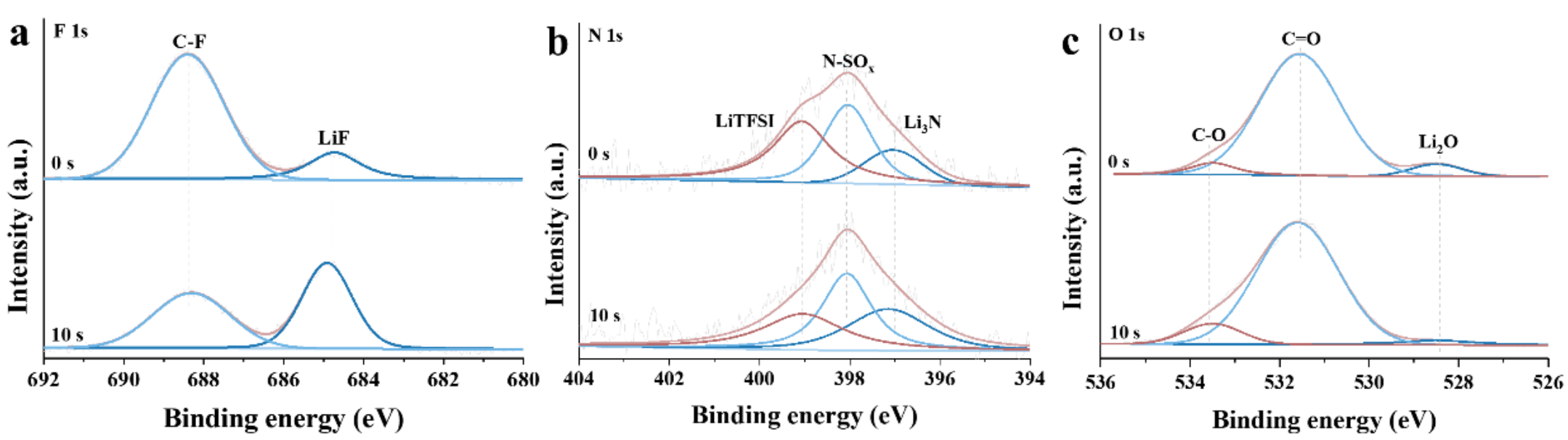

Fig. R23 | High-resolution XPS spectra of the CEI after cycling: F (a), N (b), and O (c).

Please check the newly added **Figs. S18-19** and **S21-24** on **Pages 11-13** in the

supplementary information, and the newly added discussion on **Pages 10-11** in the main text.

Comment 6: *The solubility of LiCl and LiBr in ether-based solvents is low. Please provide the concentrations of Cl^- and Br^- ions in the described battery system and discuss their potential changes.*

Response: Thank you for your constructive comments and valuable suggestions. As $EVBr_2$ functions as the cathode-active material, free Br^- species are not expected to be present in the system under operating conditions. Given the introduction of LiCl into the electrolyte, we further quantitatively analyzed the concentration of free Cl^- species. Automated potentiometric titration reveals that the Cl^- concentration in the pristine electrolyte is 0.1 M. After electrochemical cycling, the Cl^- concentration remains unchanged in the uncharged state, while a slight decrease to 0.06 M is observed in the fully charged state.

Combined with the DFT results shown in **Fig. R12**, the decrease in Cl^- concentration can be attributed to two factors: (i) partial adsorption of Cl^- onto the EV^{2+} framework during charging, and (ii) participation of Cl^- in the Br^0-to-Br^+ conversion process. Together, these results elucidate the dynamic consumption and functional role of Cl^- during the electrochemical process.

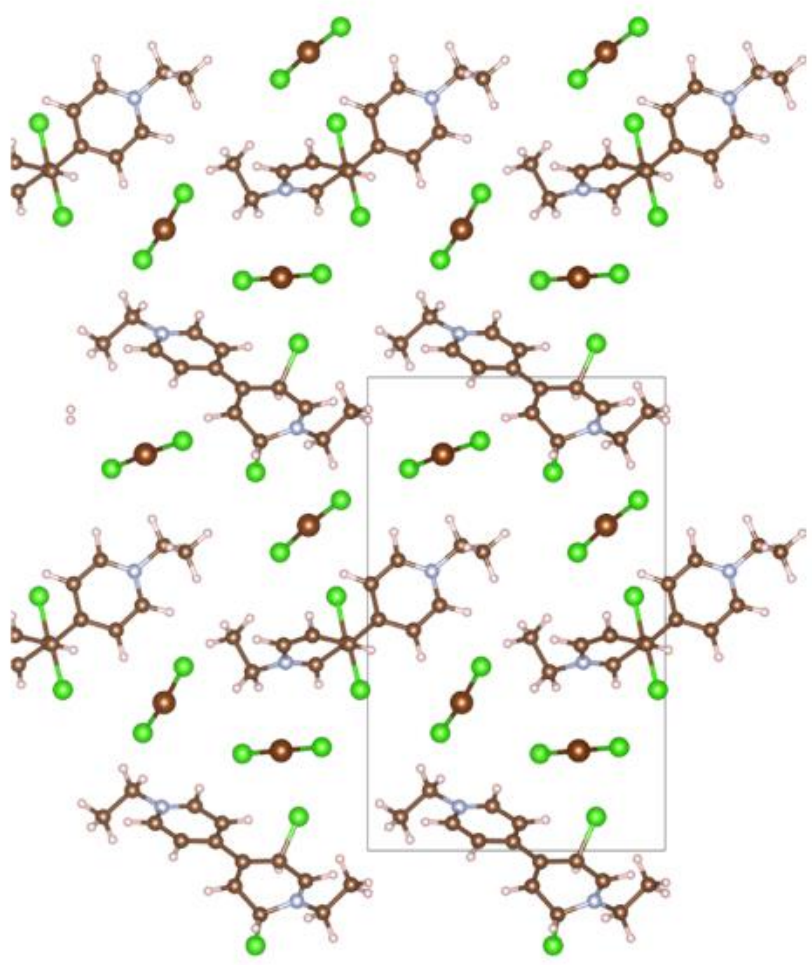

Fig. R12 | Crystal structure model of $EV^{2+}2Cl^-2(BrCl_2)$.

Comment 7: *Both Br^0 and Br^+ are strong oxidizing agents. How can they stably coexist with solvents like DME and DOL, as well as the separator?*

Response: Thank you for your constructive comments and valuable suggestions. Br^0 and Br^+ species do not coexist with the DME/DOL electrolyte. Instead, they remain confined within the cathode and do not dissolve into the electrolyte. Furthermore, these species are stabilized through interaction with Cl^- ions, leading to the formation of an electrically neutral $EV^{2+}2Cl^-2(BrCl_2)$ complexed (**Fig. R12**).

In response, we conducted a series of complementary experiments to comprehensively evaluate the stability of the electrolyte under high-voltage operation. First, LSV curves of Li||SS (stainless steel) batteries show no significant anodic current up to 4.0 V in either the DD or DDC electrolyte, indicating that both electrolytes provide sufficiently wide electrochemical stability windows to accommodate the operating potential of the Br^0/Br^+ redox couple (**Fig. R5**). Second, to examine whether the DDC electrolyte undergoes decomposition and forms effective solid electrolyte interphase (SEI) and cathode electrolyte interphase (CEI) layers in Li||$EVBr_2$ batteries, TEM was performed on both the cycled lithium metal anode and the cathode. The images revealed a solid electrolyte interphase (SEI) of ~ 40 nm on the anode and a much thinner cathode electrolyte interphase (CEI) of ~ 3.5 nm on the cathode (**Fig. R21** and **Fig. R16**). These observations indicate that limited and controlled electrolyte decomposition occurs during the early cycles, leading to the *in-situ* formation of compact SEI and CEI films. Finally, the operating voltage window of the Li||$EVBr_2$ battery was extended to 4.0 V to further probe the electrolyte behavior at high potentials. Cyclic voltammetry revealed a reversible redox couple at approximately 4.0/3.85 V, which is assigned to the Cl^-/Cl^0 reaction rather than to irreversible electrolyte decomposition (**Fig. R4**). These results indicate that, after the initial formation of the SEI and CEI, the electrolyte remains stable across the entire operating window, likely due to the protective nature of these interfacial layers.

In addition, the cycled separator was examined by SEM and EDS. SEM images show that the separator surface remains intact and uniform, without observable

structural damage, while EDS confirms the absence of bromine (**Fig. R24**). These observations demonstrate the strong bromine-confining capability of the EV framework, which effectively suppresses dissolution and shuttling of active species, preventing their accumulation on the separator. Consequently, the separator remains structurally and functionally stable, indicating good compatibility with the $EVBr_2$ cathode material.

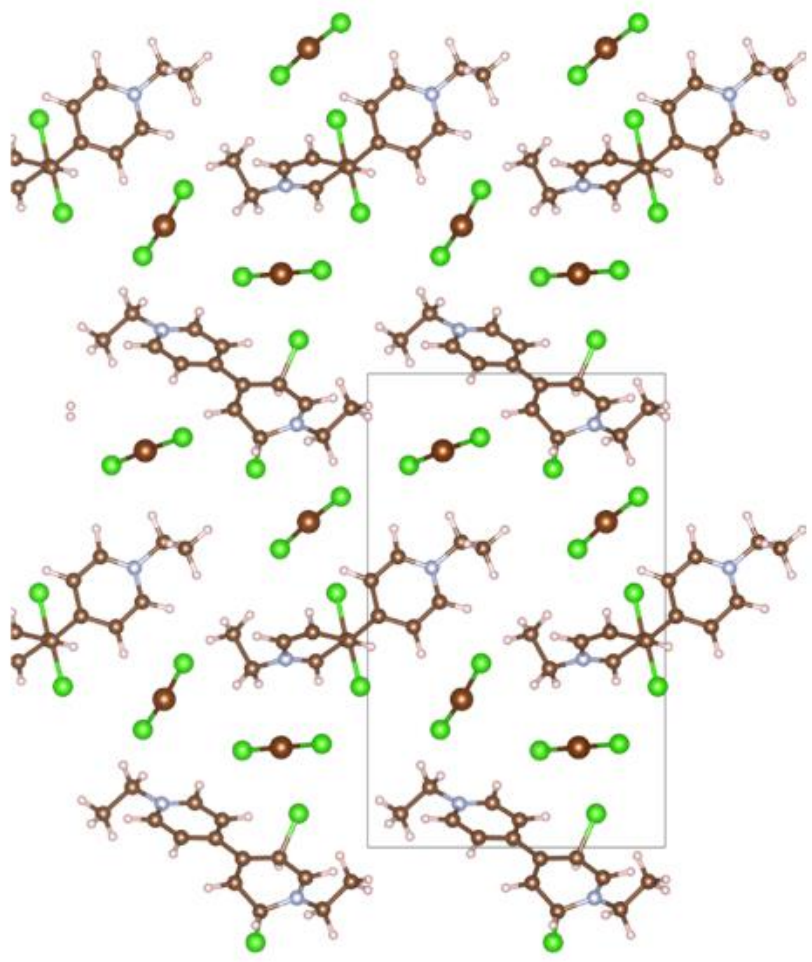

Fig. R12 | Crystal structure model of $EV^{2+}2Cl^{-}2(BrCl_2)$.

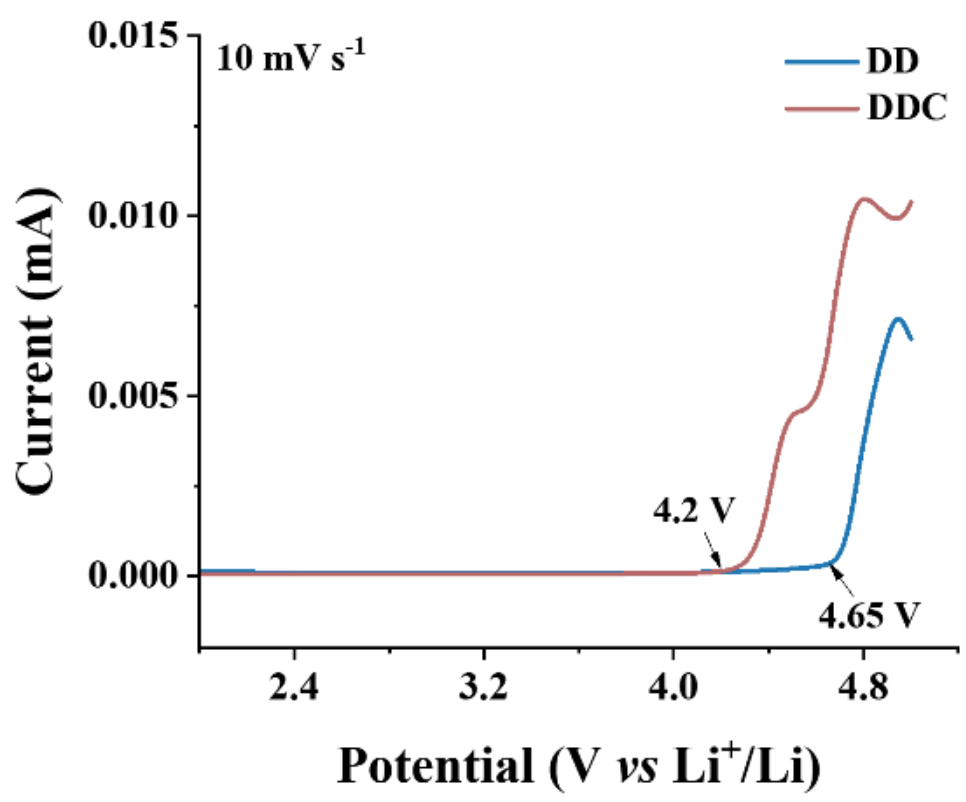

Fig. R5 | LSV curves of asymmetric Li||SS battery at 10 mV s^{-1}.

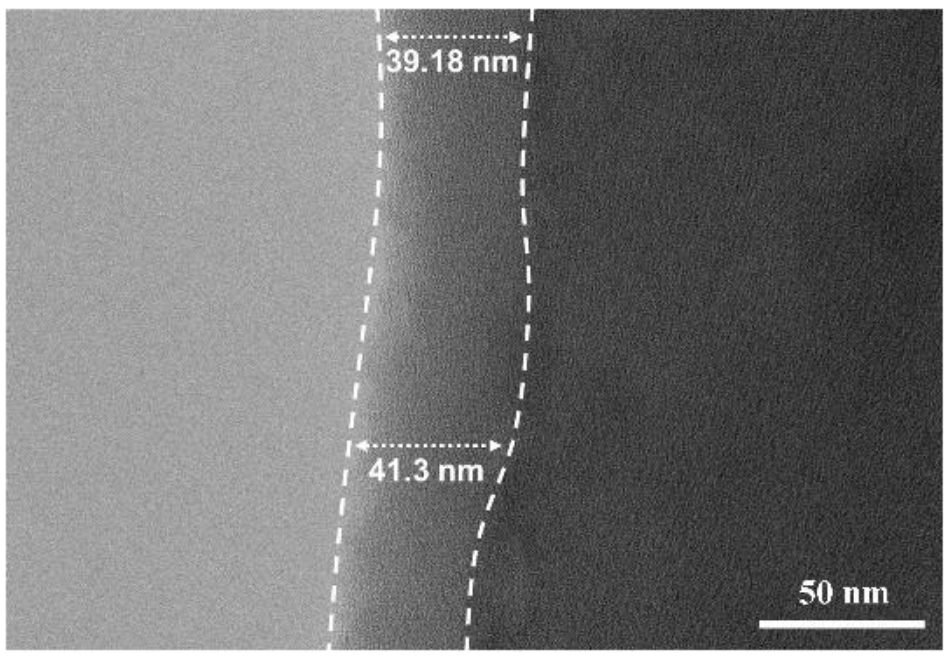

Fig. R21 | TEM image of the SEI layer.

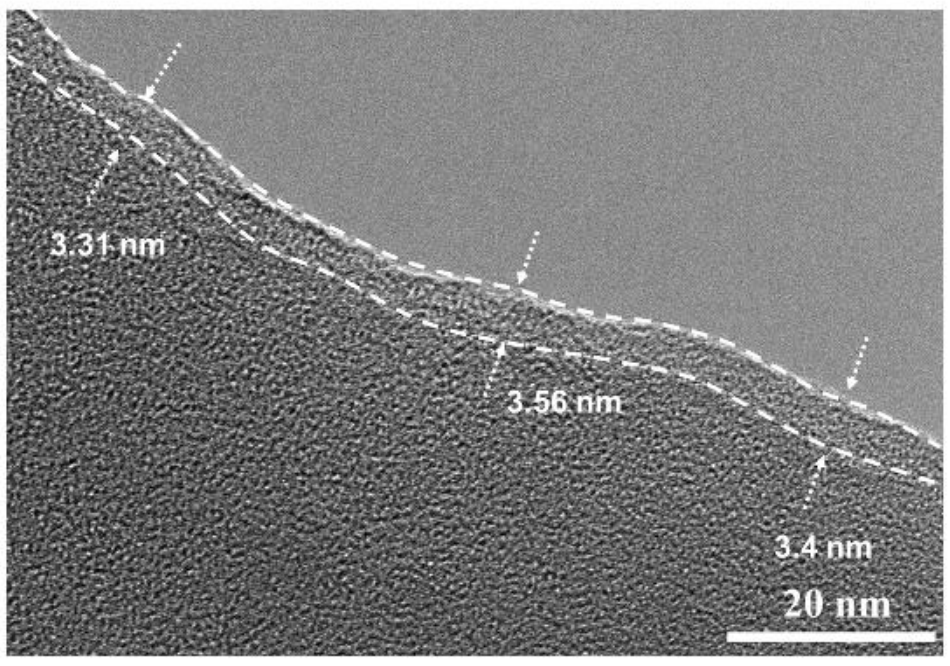

Fig. R16 | TEM image of the CEI layer.

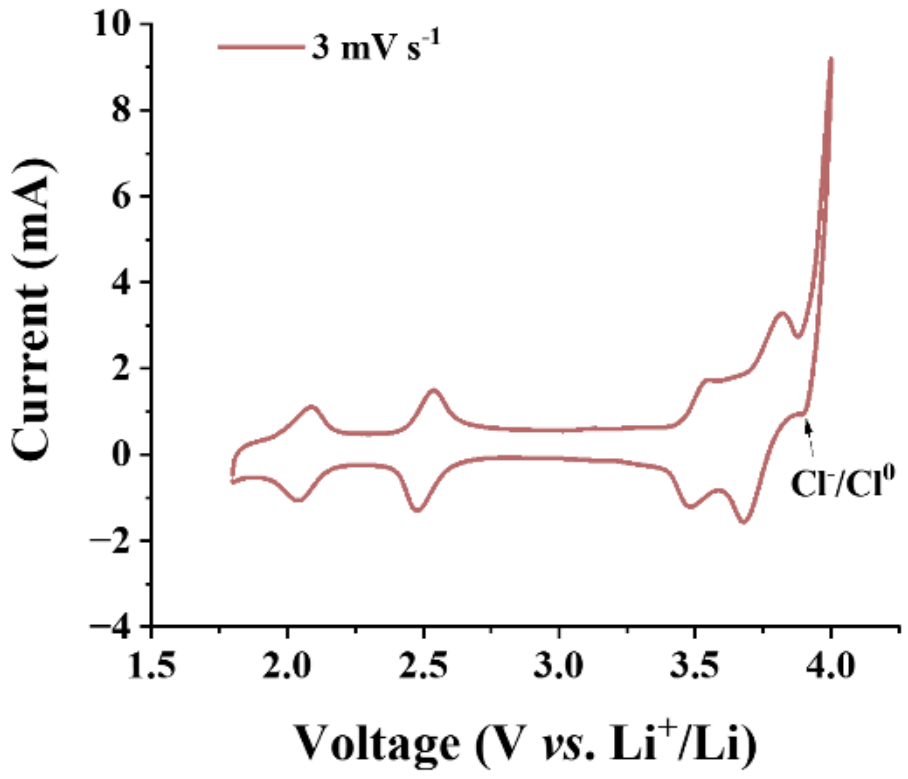

Fig. R4 | CV curve of Li||EVBr$_2$ battery in DDC electrolyte.

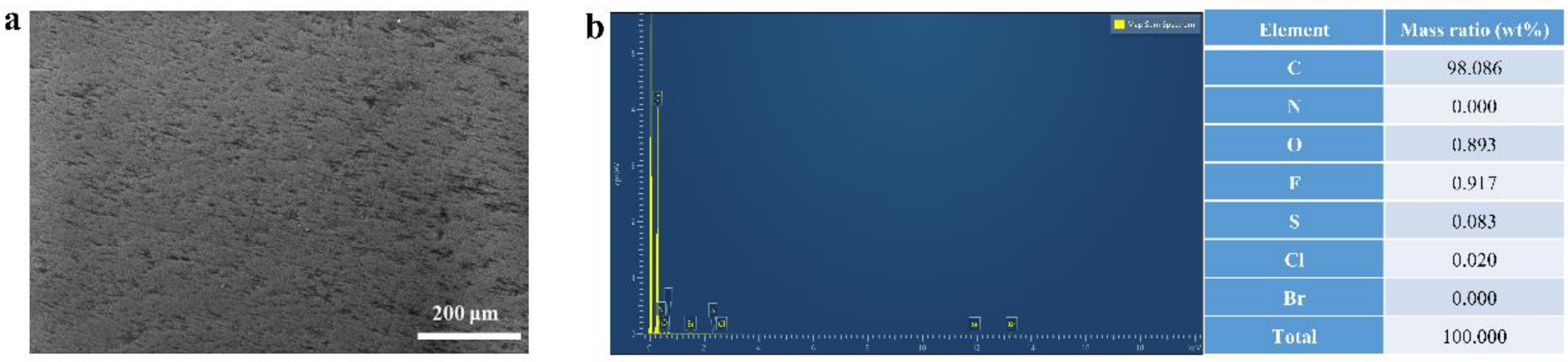

Element	Mass ratio (wt%)
C	98.086
N	0.000
O	0.893
F	0.917
S	0.083
Cl	0.020
Br	0.000
Total	100.000

Fig. R24 | (a) SEM image of the separator after cycling. **(b)** EDS mapping of the

separator after cycling.

Please check the newly added **Fig. 3g on Page 11** and the newly added discussion on **Pages 10 and 11** in the main text, and the newly added **Figs. S15a, S20-21, S23, and S43** on **Pages 10-12 and 20** in the supplementary information.

Comment 8: *The authors mention a four-step electrochemical process for this six-electron transfer system. Is there a rate-determining step among these?*

Response: Thank you for your constructive comments and valuable suggestions. As shown in **Fig. R25**, the EV^{2+} redox proceeds via Li^+ insertion/extraction, in contrast to the conversion-type mechanism of bromine. To identify the rate-controlling steps, scan-rate-dependent CV curves were analyzed (**Figs. R26-27**). The derived *b* values offer critical insight: a value near 0.5 indicates a diffusion-controlled process, while a value approaching 1.0 signifies a surface-controlled capacitive process.

In our system, *b*-value analysis identifies the Br^0 to Br^+ conversion with a *b* value nearest to 0.5 (**Figs. R29-30**), indicating that this step is diffusion-controlled and thus constitutes the rate-determining step in the overall multi-electron process.

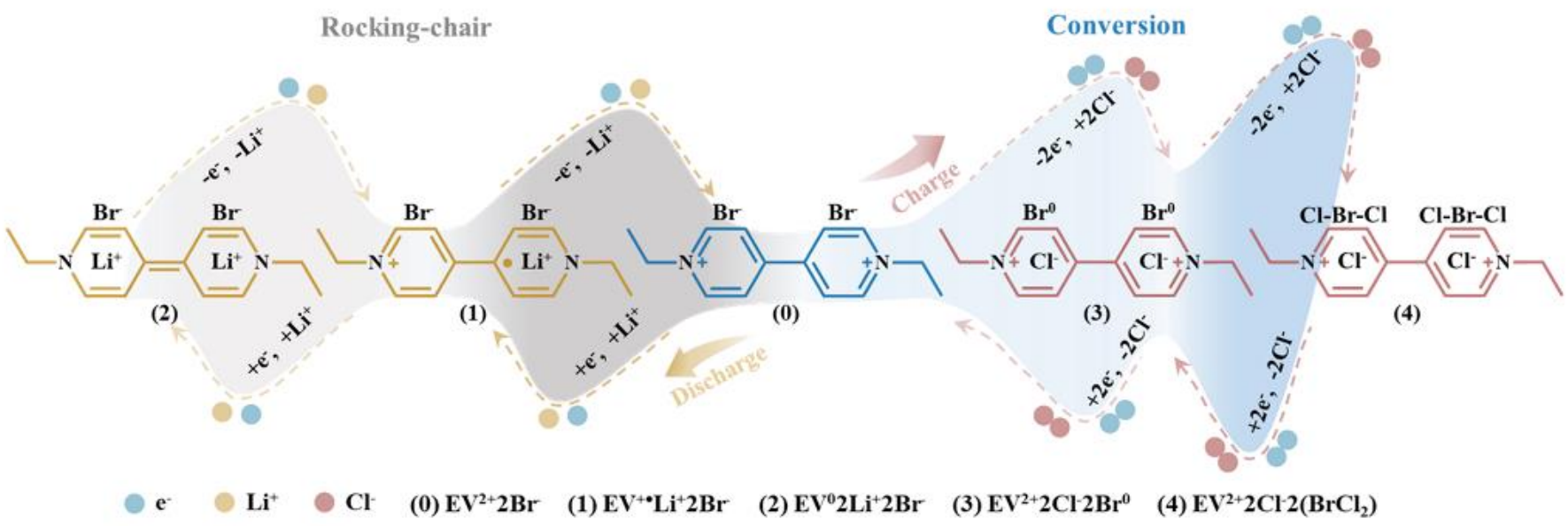

Fig. R25 | Diagram of six-electron reaction mechanism of $Li||EVBr_2$ battery.

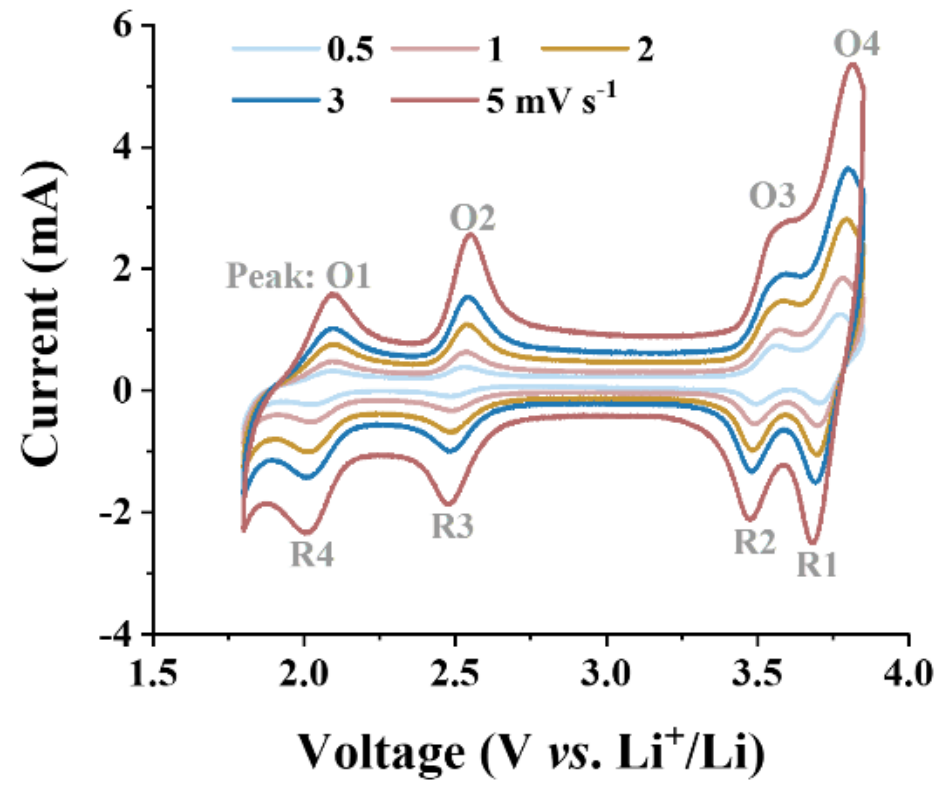

Fig. R26 | CV curves of Li||$EVBr_2$ battery in DDC electrolyte ranging from 0.5 to 5 mV s^{-1}.

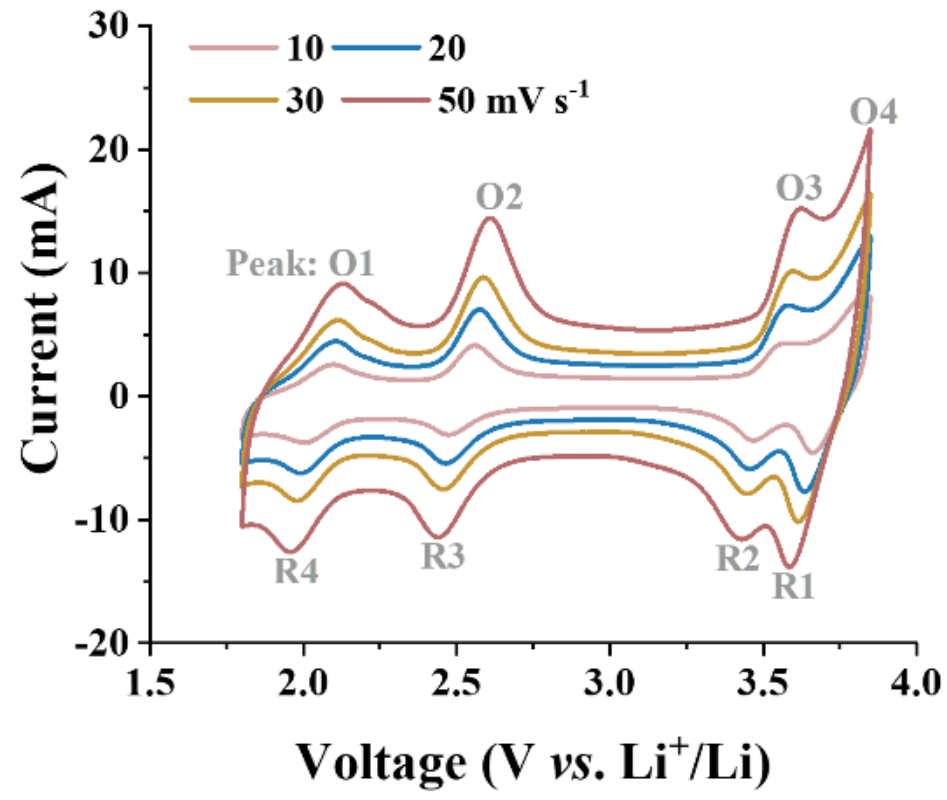

Fig. R27 | CV curves of Li||$EVBr_2$ battery in DDC electrolyte ranging from 10 to 50 mV s^{-1}.

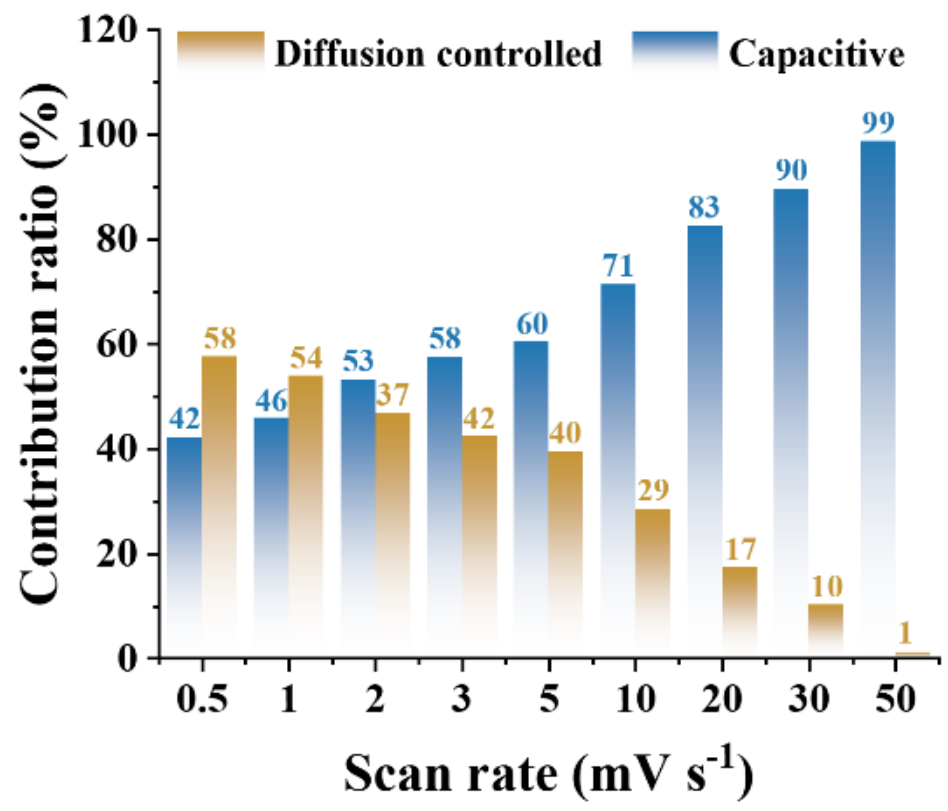

Fig. R28 | Capacitive and diffusion-controlled contribution ratios at different scanning rates.

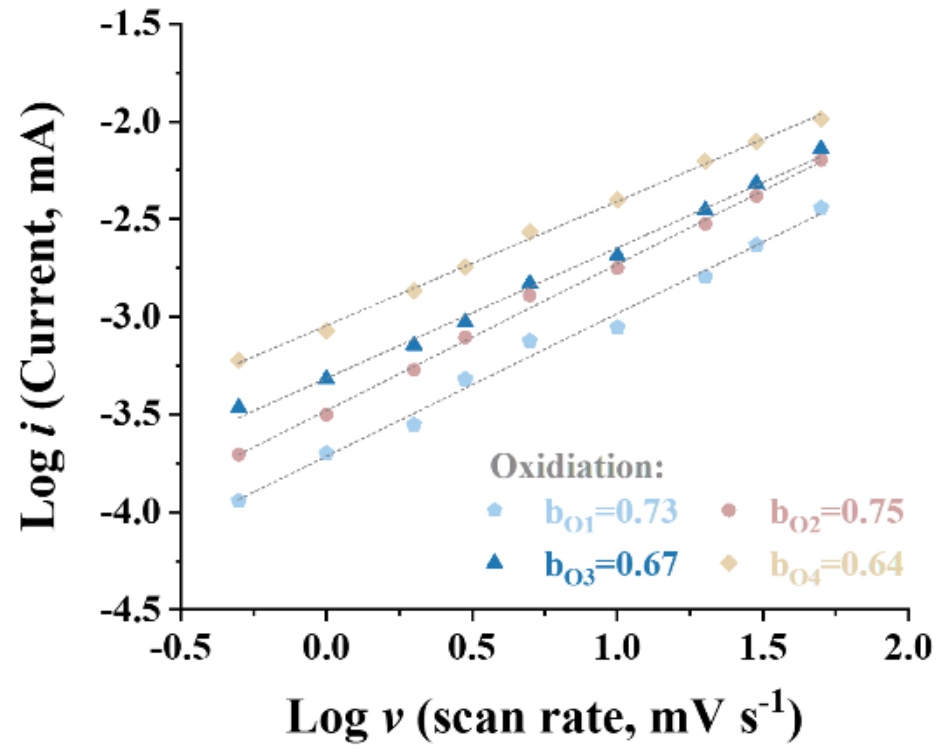

Fig. R29 | The calculated *b* values of oxidation peaks.

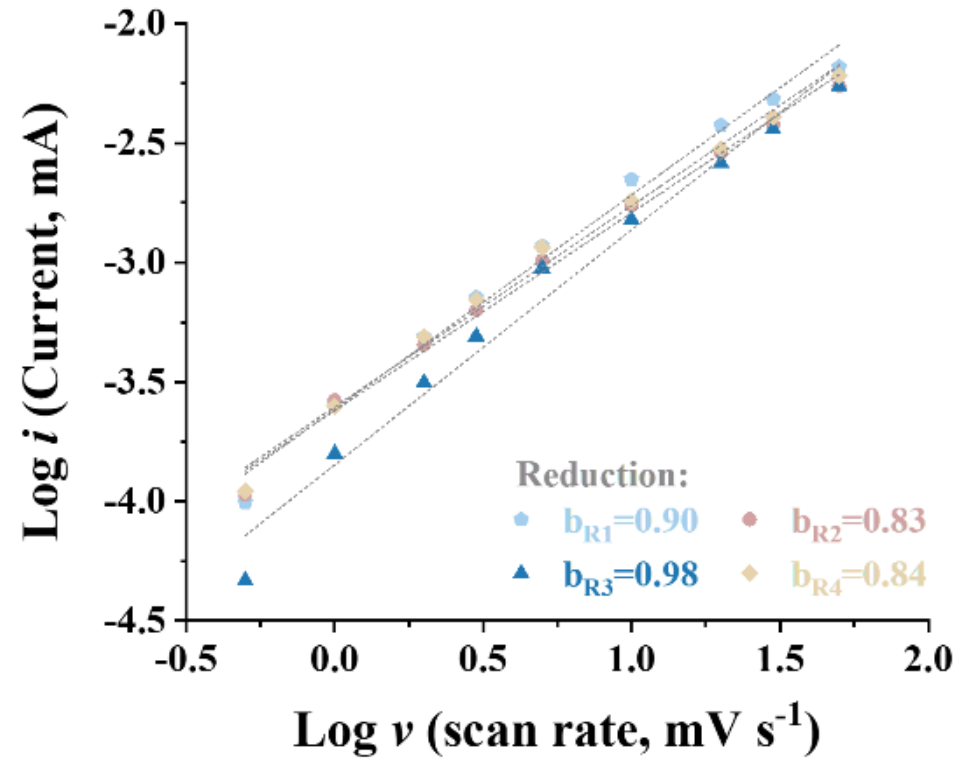

Fig. R30 | The calculated *b* values of reduction peaks.

Please check the modified **Figs. 4a-c** on **Pages 14** and **Fig. 6d** on **Page 20**, along with the improved discussion on **Page 12** in the main text, and the newly added **Figs. S25-26** on **Page 13** in the supplementary information.

Comment 9: *The influence of the introduced EV ligand and halogens on the Li^+ solvation structure needs to be evaluated.*

Response: Thank you for your constructive comments and valuable suggestions. In accordance with your comment, we have systematically evaluated the influence of the introduced ethyl viologen (EV) ligand and halogen species on the Li^+ solvation structure. In the present system, $EVBr_2$ is immobilized as the cathode active material within the electrode and does not dissolve into the electrolyte. Therefore, it does not

directly participate in the Li^+ solvation process, and its electrochemical reaction follows a static conversion mechanism. In contrast, the LiCl additive introduced into the electrolyte can directly interact with Li^+ ions, thereby modifying the Li^+ solvation environment. Accordingly, molecular dynamics (MD) simulations were performed to explicitly investigate the effect of LiCl addition on the Li^+ solvation sheath structure, coordination environment, and ion-transport behavior.

Comparative molecular dynamics (MD) simulations of the bare and LiCl-added electrolytes reveal that the introduced Cl^- ions are homogeneously distributed without significant aggregation (**Fig. R31**). Analyses of radial distribution functions (RDFs) and coordination numbers indicate that Cl^- ions competitively enter the primary Li^+ solvation sheath, partially displacing solvent molecules and weakening short-range interactions between Li^+ and $TFSI^-/NO_3^-$ (**Fig. R32**). Consequently, the Li^+ solvation structure transitions from a solvent-dominated configuration to a Cl^--participating coordination environment. Li^+ exhibits a significant increase in mean square displacement upon LiCl addition, with the diffusion coefficient rising from 1.81×10^{-10} to 2.25×10^{-10} m^2 s^{-1}, indicating enhanced Li^+ transport kinetics (**Fig. R33**).

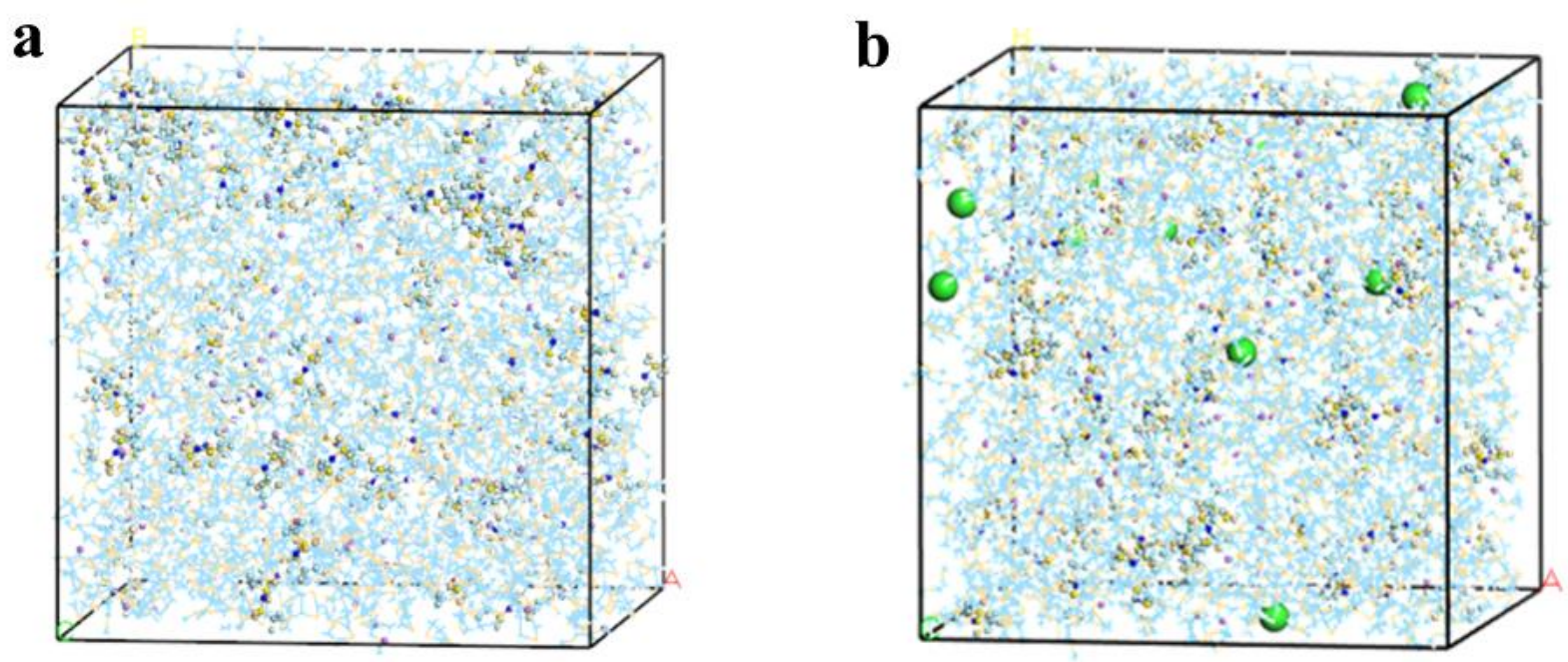

Fig. R31 | (a) MD simulation snapshots of the DD electrolyte. **(b)** MD simulation snapshots of the DDC electrolyte.

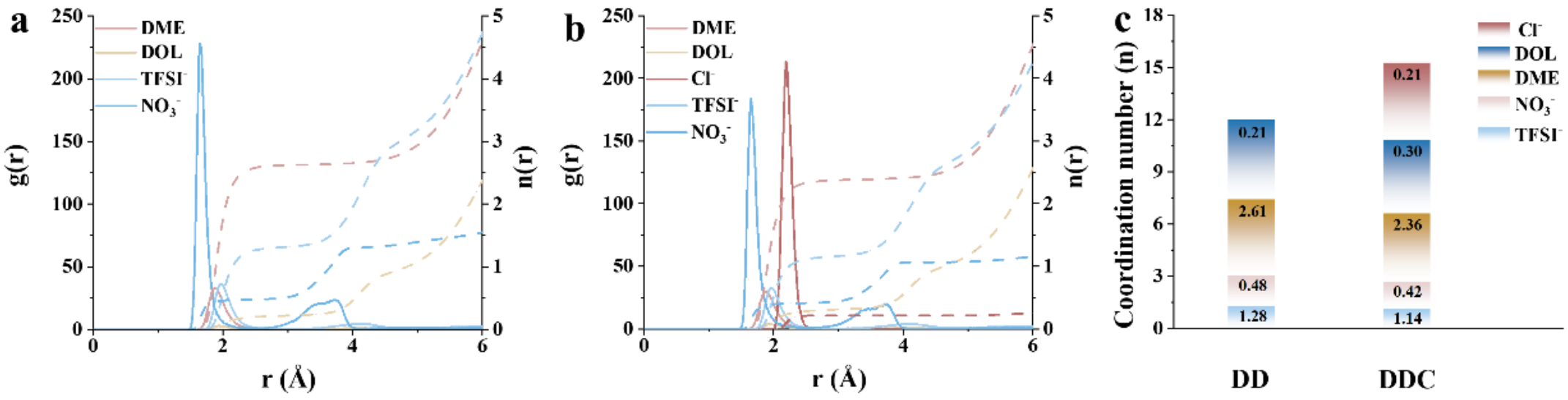

Fig. R32 | (a) RDFs of the DD electrolyte obtained from MD simulations. **(b)** RDFs of the DDC electrolyte obtained from MD simulations. **(c)** Coordination numbers of Li^+ with various species ($TFSI^-$, NO_3^-, DME, DOL, and Cl^-) in the DD and DDC electrolytes.

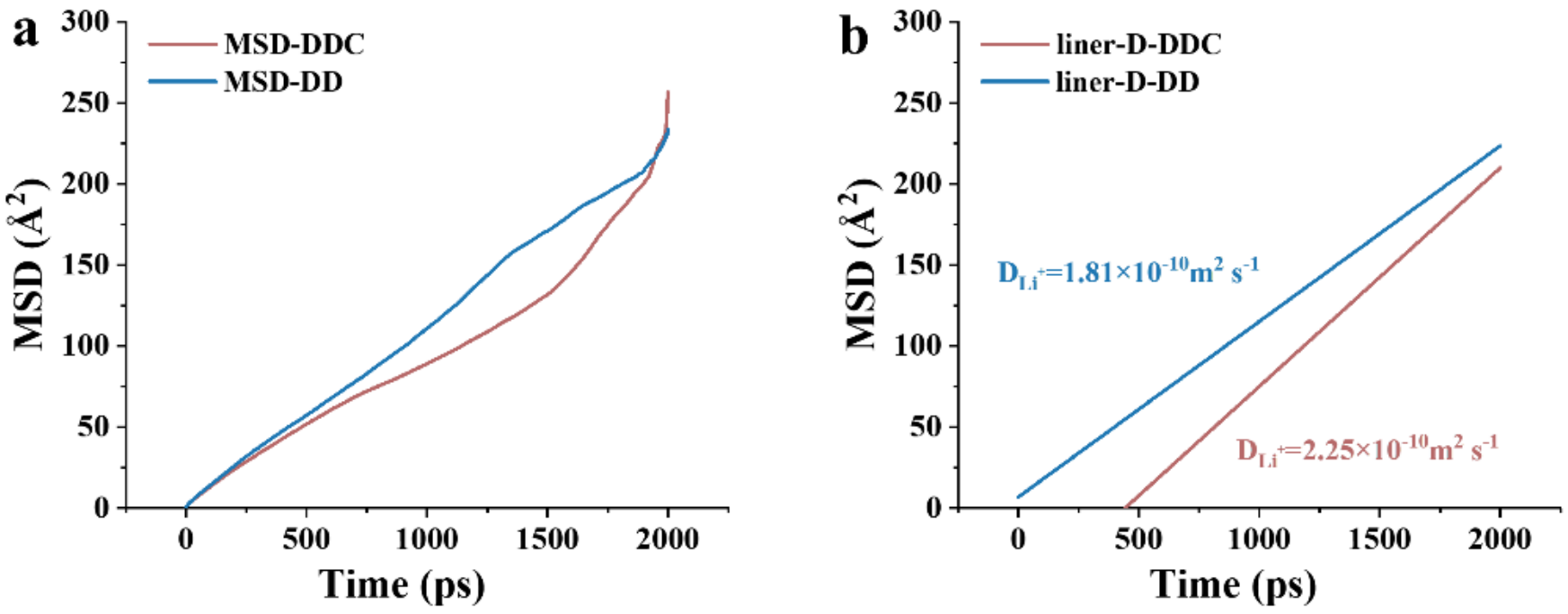

Fig. R33 | (a) MSD curves of the electrolytes. **(b)** The Li^+ diffusion coefficients derived from the MSD analysis.

Please check the newly added **Figs. 2a-f** on **Page 11** and the newly added discussion on **Pages 9-10** in the main text, and the newly added **Fig. S16** on **Page 10** in the supplementary information.

Comment 10: *The depiction of Br^0 in Fig. 5 is confusing. The authors previously stated that Br^0 exists in the form of Br_3^-, yet in Fig. 5, Br^0 is shown coordinated/adsorbed to the EV ligand. Please clarify the existence state of Br^0.*

Response: Thank you for your constructive comments and valuable suggestions. Br_3^- consists of two Br^0 units and one Br^-. In the previous version of the manuscript, multiple notations (e.g., Br^-, Br_3^-, and Br^0) were used to describe bromine species, which may have caused confusion for readers. We apologize for this lack of clarity. To more clearly

illustrate the reaction pathway and avoid ambiguity, we have unified the notation in the revised manuscript: the oxidation state generated by the one-electron oxidation of Br^- is consistently denoted as Br^0, thereby ensuring notational consistency and improving readability. We thank the reviewer for this careful and helpful comment.

Comment 11: *The authors claim extremely fast kinetics for this electrochemical system. However, kinetics are directly related to concentration. Given the presence of coordination and adsorption relationships, what are the threshold concentrations of Cl^-, Br^-, and EV required to achieve the nearly current-density-independent CV curves mentioned in the text?*

Response: Thank you for your constructive comments and valuable suggestions. We agree that reaction kinetics are closely related to the concentration of active species, and therefore we have carried out a systematic concentration-dependent study to clarify this point.

First, it should be clarified that, in the static electrode configuration employed in this work, the active material $EVBr_2$ is immobilized within the cathode, and no free Br^- species dissolve into the electrolyte. Consequently, the effective concentration of bromine species participating in interfacial electrochemical reactions is determined by the cathode loading rather than by the dissolved concentration in the electrolyte.

In response to the reviewer's question regarding a possible Cl^- concentration threshold, we systematically investigated the influence of LiCl concentration on the Br^0/Br^+ conversion reaction. The results confirm that 0.1 M LiCl (the concentration used in the main text) effectively activates and sustains the complete Br^0/Br^+ conversion. To further define the threshold, electrolytes with lower LiCl concentrations (0.06 M and 0.02 M) were examined. Low-scan-rate cyclic voltammetry (5 mV s^{-1}) reveals that at 0.06 M LiCl, well-defined Br^0/Br^+ redox peaks are still observed, indicating that the conversion remains feasible. In contrast, at 0.02 M LiCl, the characteristic Br^0/Br^+ redox peaks nearly disappear, indicating that the Br^0/Br^+ conversion cannot be driven when the Cl^- concentration is insufficient (**Fig. R34**).

These results confirm that the Br^0/Br^+ conversion is highly dependent on the Cl^-

concentration in the electrolyte. Accordingly, the use of a slight excess of Cl^- (e.g., 0.1 M) is necessary to ensure complete conversion and stable high-rate performance, consistent with the near current-density-independent fast kinetics observed in this work.

We thank the reviewer again for helping us to further clarify the underlying reaction mechanism.

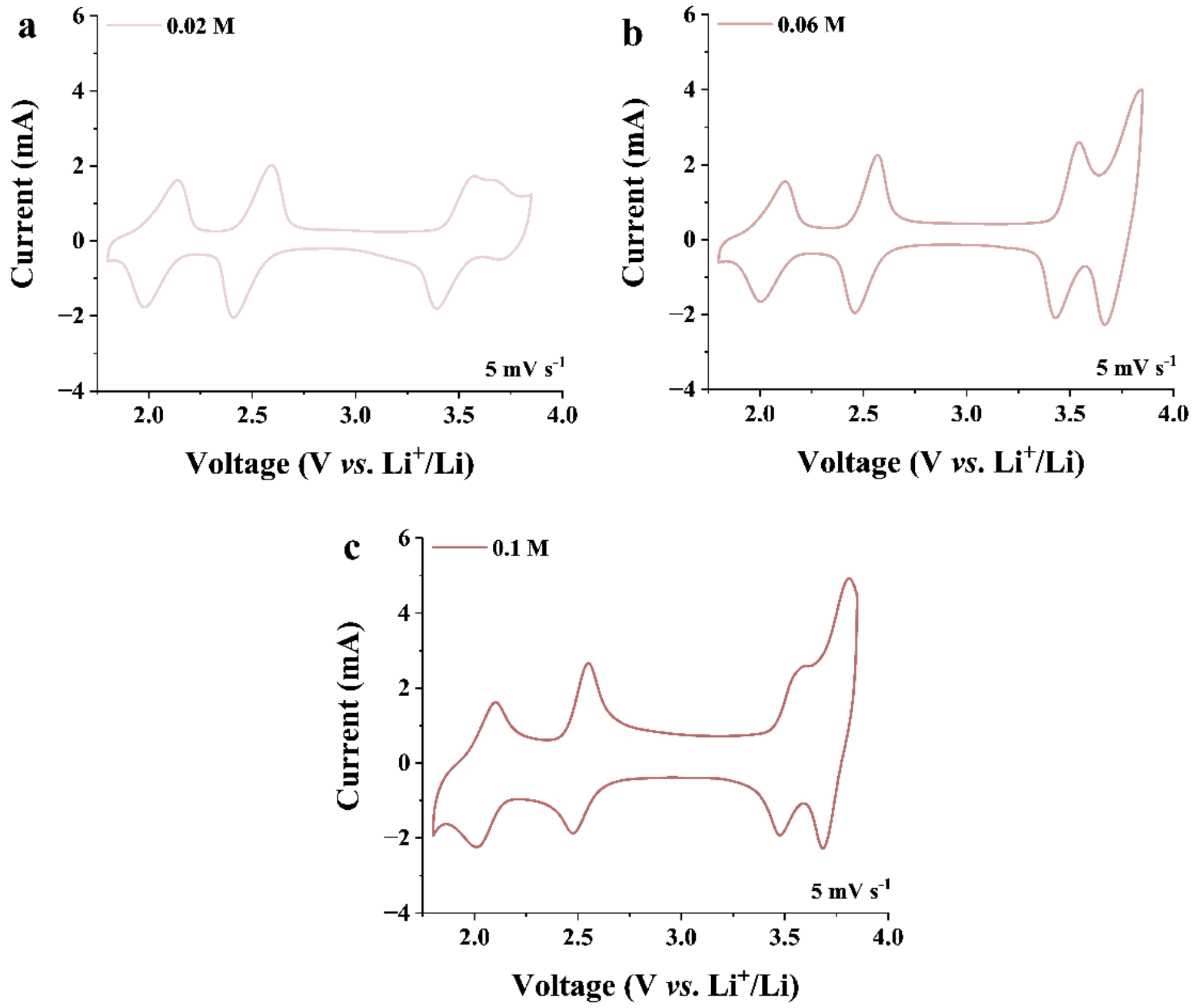

Fig. R34 | CV curve of Li||$EVBr_2$ battery in DD electrolyte with different LiCl concentrations. **(a)** 0.02 M. **(b)** 0.06 M. **(c)** 0.1 M.

Please check the newly added **Figs. S13** on **Page 9** in the supplementary information and the newly added discussion on **Page 7** in the main text.

Comment 12: *A comparative analysis with recent advances in multi-electron bromine batteries is missing. The authors should include a performance comparison table in the Supplementary Information to clearly contextualize the advancements claimed in this work.*

Response: Thank you for your constructive comments and valuable suggestions. In response, we have added a comprehensive comparison with recent advances in multi-electron bromine battery systems (**Fig. R35**). A detailed performance comparison table has been included in the Supplementary Information (**Table R1**). This comparison more clearly contextualizes the performance achieved in this work relative to state-of-the-art multi-electron bromine batteries and highlights the advancements enabled by our strategy.

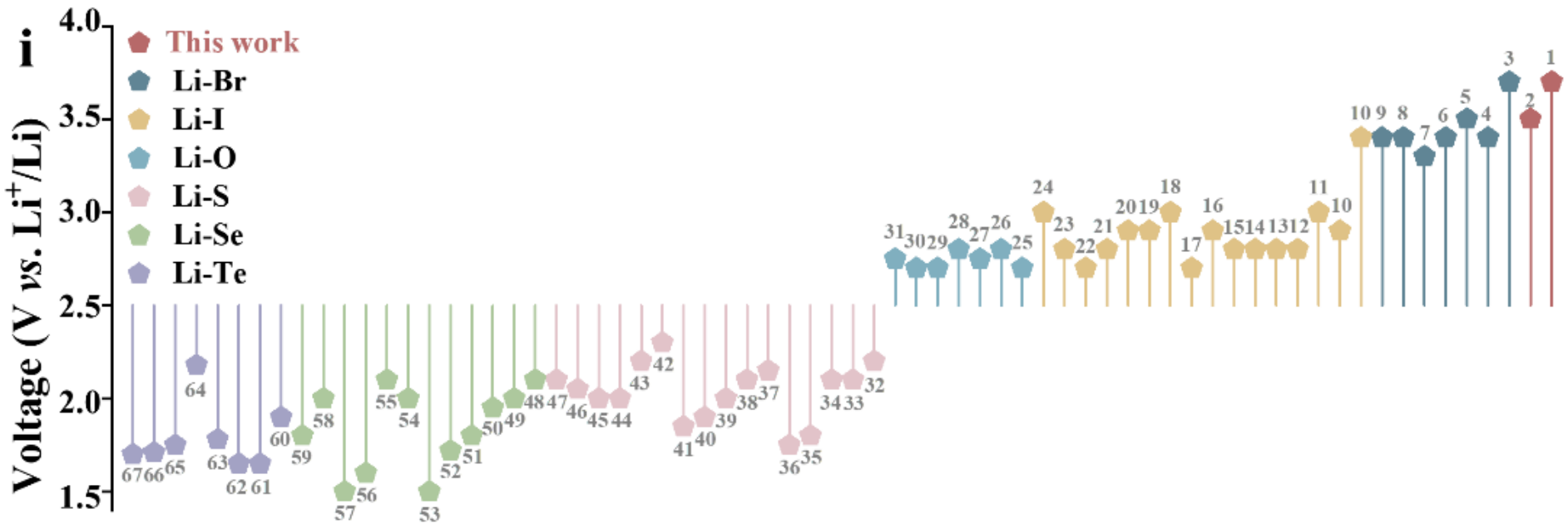

Fig. R35 | Performance comparison on output voltage between this work and reported conversion-type Li batteries.

Table R1. Comparison of the electrochemical performance between this work and state-of-the-art lithium-ion batteries.

Battery type	Number	Anode	Plateau voltage (V)	Reference
Br	1	Li	3.7	**This work**
	2	Li	3.5	**This work**
	3	Li	3.7	*Sci. Adv.*[7]
	4	Li	3.4	*Adv. Mater.*[8]
	5	Li	3.5	*Joule*[9]
	6	Li	3.4	*J. Mater. Chem. A*[10]
	7	Li	3.3	*J. Energy Chem.*[11]
	8	Li	3.4	*Adv. Energy Mater.*[12]
	9	Li	3.4	*J. Power Sources*[13]
I	10	Li	3.4	*J. Am. Chem. Soc.*[14]
	10	Li	2.9	*J. Am. Chem. Soc.*[14]
	11	Li	3	*Adv. Mater.*[14]

	12	Li	2.8	*Energy Storage Mater.*[15]
	13	Li	2.8	*ACS Nano*[16]
	14	Li	2.8	*Angew. Chem. Int. Ed.*[17]
	15	Li	2.8	*Energy Environ. Sci.*[18]
	16	Li	2.9	*Adv. Funct. Mater.*[19]
	17	Li	2.7	*Carbon Energy*[20]
	18	Li	3	*Nat. Commun.*[21]
	19	Li	2.9	*J. Mater. Chem. A*[22]
	20	Li	2.9	*J. Power Sources*[23]
	21	Li	2.8	*ACS Nano*[16]
	22	Li	2.7	*J. Mater. Chem. A*[24]
	23	Li	2.8	*J. Mater. Chem. A*[25]
	24	Li	3	*Energy Storage Mater.*[26]
O	25	Li	2.7	*Nat. Mater.*[27]
	26	Li	2.8	*Chem. Rev.*[28]
	27	Li	2.75	*Nat. Commun.*[29]
	28	Li	2.8	*Nat. Commun.*[30]
	29	Li	2.7	*Adv. Mater.*[31]
	30	Li	2.7	*Adv. Energy Mater.*[32]
	31	Li	2.75	*Angew. Chem. Int. Ed.*[33]
S	32	Li	2.2	*Joule*[34]
	33	Li	2.1	*Adv. Sci.*[35]
	34	Li	2.1	*Adv. Energy Mater.*[36]
	35	Li	1.8	*Nano-Micro Lett.*[37]
	36	Li	1.75	*Adv. Mater.*[38]
	37	Li	2.15	*Energy Environ. Sci.*[39]
	38	Li	2.1	*Angew. Chem. Int. Ed.*[40]
	39	Li	2	*Angew. Chem. Int. Ed.*[41]
	40	Li	1.9	*Nat. Mater.*[42]
	41	Li	1.85	*Energy Storage Mater.*[43]
	42	Li	2.3	*ACS Nano*[44]
	43	Li	2.2	*Adv. Mater.*[45]
	44	Li	2	*Adv. Mater.*[46]
	45	Li	2	*Adv. Funct. Mater.*[47]
	46	Li	2.05	*Adv. Sci.*[48]
	47	Li	2.1	*Adv. Funct. Mater.*[49]
Se	48	Li	2.1	*J. Mater. Chem. A*[50]
	49	Li	2	*Adv. Mater.*[51]

	50	Li	1.95	*Joule*[52]
	51	Li	1.8	*Adv. Funct. Mater.*[53]
	52	Li	1.72	*J. Mater. Chem. A*[54]
	53	Li	1.5	*ACS Nano*[55]
	54	Li	2	*Energy Environ. Sci.*[56]
	55	Li	2.1	*Adv. Mater.*[57]
	56	Li	1.6	*Nano Energy*[58]
	57	Li	1.5	*Adv. Energy Mater.*[59]
	58	Li	2	*Energy Environ. Mater.*[60]
	59	Li	1.8	*Chem. Eng. J.*[61]
Te	60	Li	1.9	*Energy Storage Mater.*[62]
	61	Li	1.65	*ACS Nano*[63]
	62	Li	1.65	*J. Alloy. Compd.*[64]
	63	Li	1.78	*Nano Energy*[65]
	64	Li	2.18	*Energy Storage Mater.*[66]
	65	Li	1.75	*Carbon*[67]
	66	Li	1.71	*J. Power Sources*[68]
	67	Li	1.7	*Nano Energy*[69]

Please check the modified **Fig. 4i** on **Page 14** in the main text and the newly added **Table S1** on **Pages 21-22** in the supplementary information.

Comment 13: *The assignment of the peak at 270 cm^{-1} in the in-situ Raman spectra to the key species Br-Cl requires support from literature references.*

Response: Thank you for your constructive comments and valuable suggestions. In the revised manuscript, to more clearly present the spectral evidence, we have redrawn the corresponding figure and supplemented it with the relevant raw spectral data. Furthermore, we have added appropriate literature references to support the assignment of the Raman band at 270-290 cm^{-1} to Br-Cl species. This band has been previously identified as the characteristic Br-Cl stretching vibration [*Acs Energy Lett. 7, 1034-1039 (2022), Sci. Adv. 10, 10 (2024), Joule 8, 461-481 (2024), Angew. Chem. Int. Ed. 64, e202421905 (2025)*]. The related discussion in the text has been updated accordingly.

Please check the modified **Fig. 5b** on **Page 17** and the improved discussion on **Pages 15-16,** as well as the newly added **references 25, 28, 40, and 68 (on Pages 24, 25, and 28)** in the main text, and the newly added **Fig. S34** on **Page 17** in the supplementary information**.**

Comment 14: *The text only provides a single-cycle curve for EV. The electrochemical performance of EV itself as an organic electrode material also needs to be provided.*

Response: Thank you for your constructive comments and valuable suggestions. Following your suggestion, we have supplemented additional electrochemical characterizations of EV^{2+}, including cyclic voltammetry (CV) curves collected at scan rates from 0.5 to 20 mV s^{-1}, rate capability tests at current densities of 1, 2, 3, 5, and 10 A g^{-1}, the corresponding galvanostatic charge-discharge profiles, and cycling performance at a current density of 3 A g^{-1}. A detailed analysis of these results is provided below.

Experimental validation leveraged halogen-free $EV(ClO_4)_2$ as a model compound to decipher EV^{2+} electrochemistry in the same DD electrolyte. At 3 mV s^{-1}, the Li||$EV(ClO_4)_2$ battery displayed accurately aligned redox peaks at identical potentials, while synchronized charge/discharge plateaus affirmed electrochemical consistency in the GCD curves (**Supplementary Fig. 6**).[43] Thus, the peak at 2.60/2.43 V corresponds to the single-electron reduction of EV^{2+} coupled with concerted Li^+ ion intercalation, yielding monovalent radical species ($EV^{+\bullet}$). Subsequent electron transfer at 2.14/1.97 V drives the progressive reduction of $EV^{+\bullet}$, accompanied by secondary Li^+ ion intercalation, which culminates in the formation of neutral EV^0. Oxidation pathways precisely retrace the original reaction coordinate in reverse sequence (**Fig. 2e**).[44, 45] Variable-scan-rate CV analysis establishes unambiguous redox peak resolution even at 20 mV s^{-1}, with voltammetric profiles maintaining essential morphological invariance across scan rates, attesting to superior redox kinetics and electrochemical reversibility throughout EV^{2+} bivalent transfer (**Fig. R36**). Multiscale current density evaluation revealed sustained two-electron plateau signatures persisting under 10 A g^{-1} high-rate

operation (**Fig. R37**). Cycling stability assessment, synergized with the acquired electrochemical evidence, collectively substantiates the functional viability of EV^{2+} as a cathode electrode material **(Fig. R9)**.

The corresponding data have been added to the revised manuscript. We again thank the reviewer for this valuable suggestion, which has helped to further strengthen and improve our work.

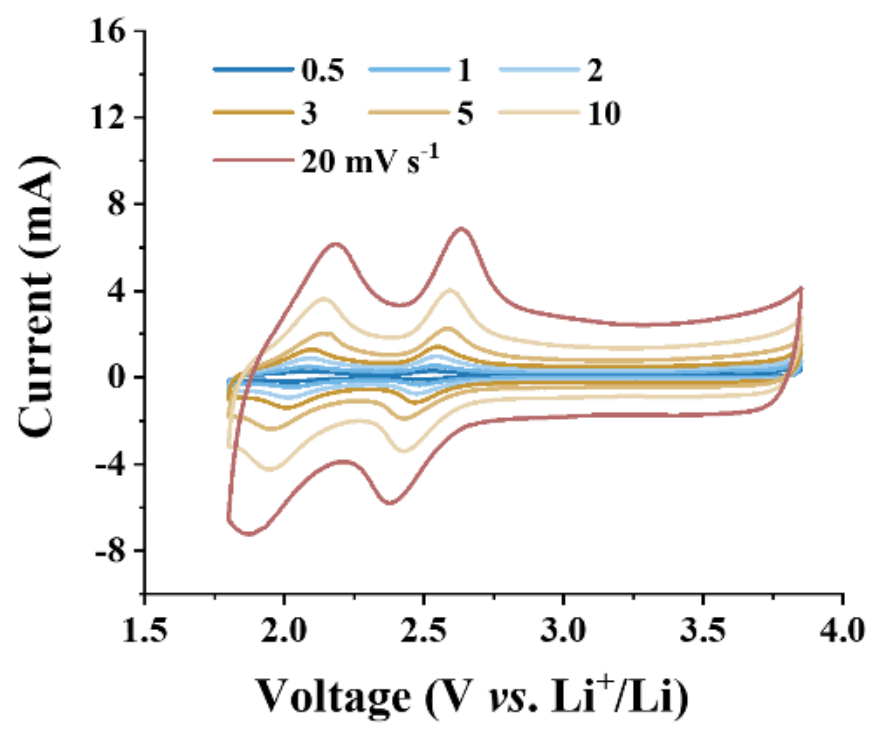

Fig. R36 | CV curves of the Li||$EV(ClO_4)_2$ battery in DD electrolyte.

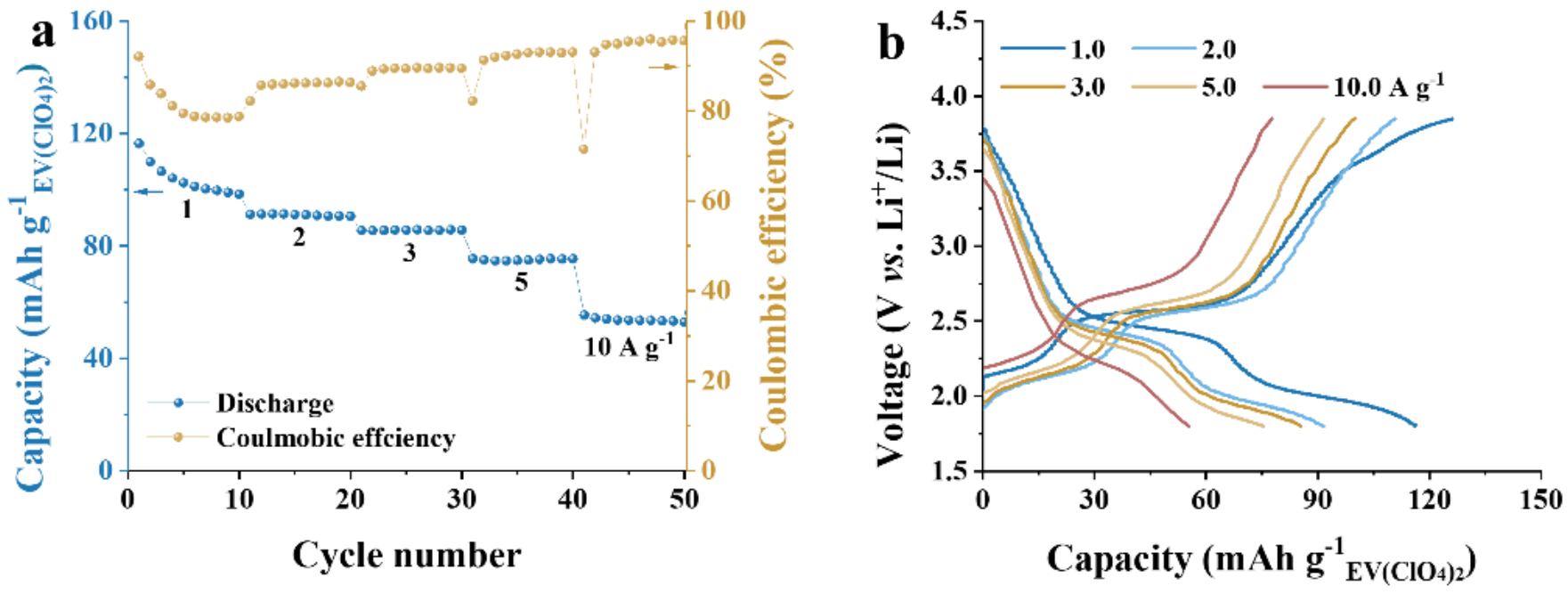

Fig. R37 | (a) Rate capability of the Li||$EV(ClO_4)_2$ battery in DD electrolyte. **(b)** GCD curves of the Li||$EV(ClO_4)_2$ battery in DD electrolyte.

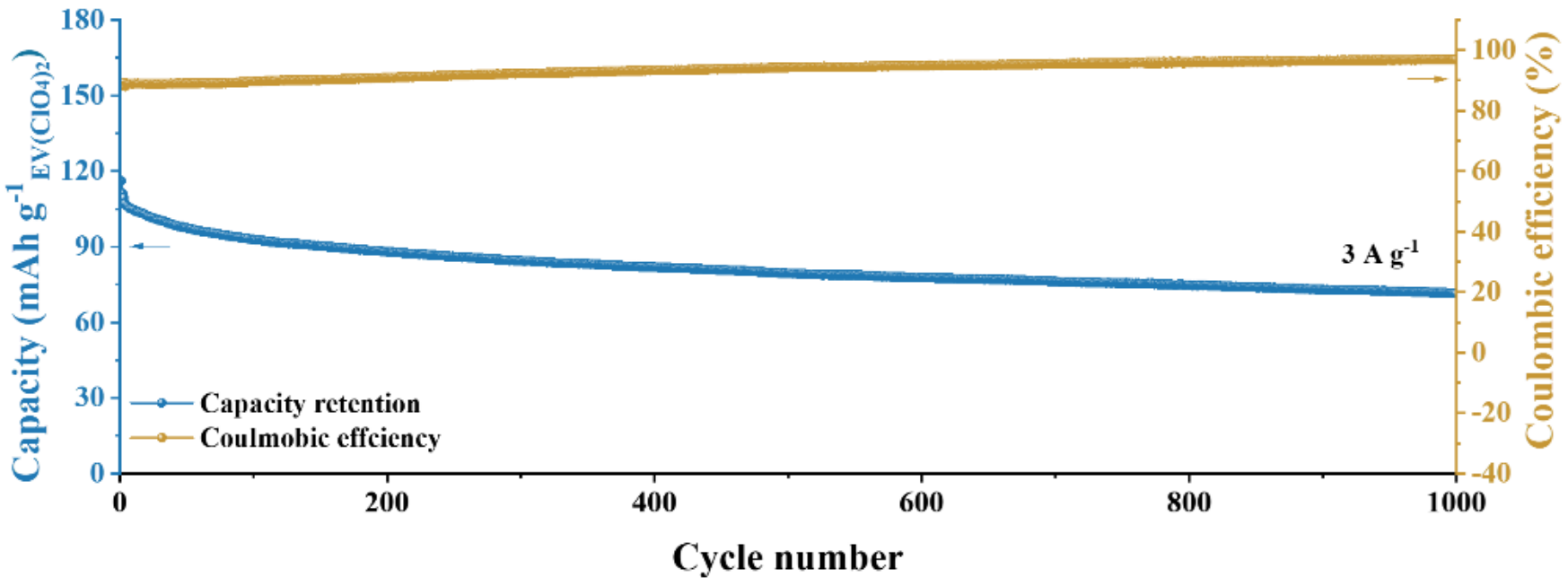

Fig. R9 | Long-term cycling performance of Li||EV(ClO_4)$_2$ battery in DD electrolyte at 3 A g^{-1}.

Please check the modified **Figs. S7-9** on **Page 7** in the supplementary information and the improved discussion on **Pages 6 and 7** in the main text.

Comment 15: *Several abbreviations, such as "GITT" (Galvanostatic Intermittent Titration Technique), are not defined upon their first appearance in the text. This may hinder comprehension for readers unfamiliar with the terminology. It is recommended that all specialized terms or acronyms be spelled out in full at their first occurrence.*

Response: Thank you for your constructive comments and valuable suggestions. We have carefully reviewed the entire manuscript and ensured that all abbreviations and acronyms (e.g., GITT) are spelled out in full at their first occurrence. The corresponding corrections have been made in the revised manuscript.

To Reviewer #3:

I co-reviewed this manuscript with one of the reviewers who provided the listed reports. This is part of the Nature Communications initiative to facilitate training in peer review and to provide appropriate recognition for Early Career Researchers who co-review manuscripts.

Response: We sincerely thank the reviewer for the careful evaluation of our manuscript and for providing valuable and professional comments. All comments have been addressed point by point in the revised manuscript. These insightful suggestions have substantially improved the clarity, rigor, and overall quality of our work.

To Reviewer #4:

The authors present their scientific work in the manuscript entitled "Coordination electrochemistry taming reversible hypervalent bromine redox for energetic six-electron-transfer lithium-bromine battery". This article extends the application of ethyl viologen from magnesium and lithium-air batteries to utilise ethyl viologen dibromoide as a cathode for lithium-bromine batteries. It well-justifies the application based on the volatility, sluggish reaction kinetics, and energy density limitations of Br-based batteries. Although this article achieves a capacity of 632.8 mAh $g^{-1}{}_{Br}$ and an energy density of 1810 Wh $kg^{-1}{}_{Br}$ using a six-electron-transfer mechanism, several major issues need to be addressed:

Response: We sincerely thank the reviewer for the positive assessment and supportive comments on our manuscript. We are greatly encouraged that the reviewer acknowledges our work, particularly our extension of the ethyl viologen application and its underlying research rationale, which they affirm as significant. In response, we have meticulously addressed each of the raised points through thorough analysis, supplementing the study with necessary experimental validations and supporting data. We believe the corresponding revisions have further improved the quality of our manuscript.

Comment 1: *The quality of English writing is poor and requires significant editing. To give just three examples of when the writing noticeably caused confusion and broke the flow of the manuscript.*

There are contradictions, imprecise terminology, and poor grammar:

o "Nevertheless, the currently available obsolete two-electron redox mechanism of Br ($2Br^-/Br_2$) and the electrochemical inertia of nonhalogenated ligands impose the performance bottleneck." (Pg1)

Problems with formality, punctuation, non-sequitur, and tense:

o "Besides, current investigations on Br redox prioritize two-electron transfer reactions, while four-electron transfer systems delivering heightened voltage and augmented

theoretical capacity receive insufficient attention.[23, 31] *The disproportionation of hypervalent* Br^+ *poses significant challenge for reversible electrochemical process.*[32, 33]*" (Pg 3)*

And commonplace use of unscientific hyperboles and superlatives:

o "Electrochemical benchmarking in ***Fig. 3g*** *confirms the unparalleled voltage supremacy of* $EVBr_2$*." (Pg 10)*

Response: Thank you for your constructive comments and valuable suggestions. We apologize for the oversight and thank the reviewer for pointing it out. We have carefully re-examined the entire manuscript and thoroughly revised it to ensure that spelling, grammar, and overall readability meet high academic standards.

Comment 2: *The article claims that "Theoretical analyses indicate that realizing the four-electron redox process* ($2Br^-/2Br^+$) *would constitute a transformative advancement.*[21-23]*" (Pg 3) However, at the same charge rate, one of articles cited utilises two-electron-transfer to achieve a higher capacity of 653 mAh* $g^{-1}{}_{Br}$ *and energy density of 2180 Wh* $kg^{-1}{}_{Br}$ *than the six-electron-transfer claimed in this article. Why is six-electron-transfer delivering comparable performances? The argument of the article may need to be refined accordingly.*

Response: Thank you for your constructive comments and valuable suggestions. Although both this work and the cited literature report specific capacity and energy density measured at a current density of 1 A g^{-1}, the normalization bases differ. In our work, the current density is normalized to the total mass of the $EVBr_2$ composite, whereas in the literature it is normalized only to the mass of active bromine. Consequently, the higher capacity and energy density reported in the literature effectively correspond to a lower intrinsic current density when calculated on a per-mass-of-bromine basis.

Based on this understanding, we recognize that our original wording may have caused confusion for readers. We have therefore revised the relevant discussion in the manuscript, reframing the arguments to present the advantages of multi-electron redox processes in a more balanced and precise manner. We thank the reviewer once again for

this careful and constructive comment, which has greatly enhanced the rigor and clarity of our work.

Please check the modified discussion on **Page 3** in the main text.

Comment 3: *At ~2.2 V in* ***Fig. 3a****, a small shoulder for peak 1 is observable, also visible as a slight bump in* ***Fig. 3c****. What is attributable to this small peak? Can the argument that no side-reactions or byproducts are produced be reinforced using other techniques?*

Response: Thank you for your constructive comments and valuable suggestions. We have carefully considered the reviewer's comment regarding the small shoulder observed at approximately 2.2 V in **Fig. 3a** (**Fig. 4a** in the revised manuscript), which also appears as a slight bump in **Fig. 3c** (**Fig. 4e** in the revised manuscript). This feature is not associated with side reactions or the formation of new phases, but instead arises from kinetic limitations at relatively high scan rates, where slight hysteresis in the electrochemical response and incomplete synchronization of ion insertion/extraction lead to a minor current fluctuation.

To clarify this point, we have performed cyclic voltammetry measurements at lower scan rates, allowing the redox processes to proceed more completely. As shown in **Fig. R26**, the shoulder feature disappears under these conditions, and only two well-defined redox couples are observed. The redox peaks at 2.60/2.43 V can be assigned to the $EV^{2+}/EV^{+\bullet}$ redox transition, while those at 2.14/1.97 V correspond to the $EV^{+\bullet}/EV^{0}$ conversion. These results further confirm that the shoulder feature originates from kinetic effects rather than from side reactions or byproduct formation.

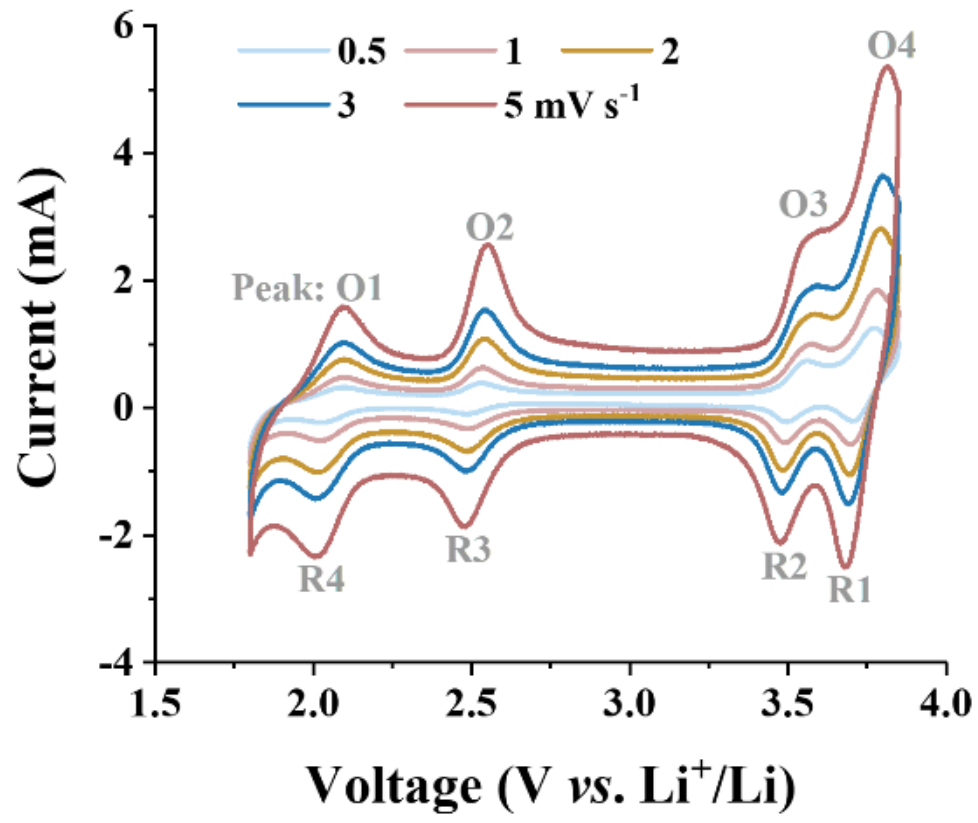

Fig. R26 | CV curves of Li||$EVBr_2$ battery in DDC electrolyte ranging from 0.5 to 5 mV s^{-1}.

Please check the modified **Fig. S25** on **Page 13** in the supplementary information and improved discussion on **Page 12** in the main text.

Comment 4: *The article claims that Br^+/Br^0 is reversible when arguing that "Newly enabled Br^0/Br^+ redox chemistry doubles Br-derived capacity while confirming reversible conversion between Br^0 and Br^+". However,* ***Fig. 3a*** *appears to feature abrupt current increases at ~3.8 V, particularly at faster charge rates, indicative of irreversible Br redox. Can CV curves of slightly higher voltage cut-offs be provided to clarify this?*

Response: Thank you for your constructive comments and valuable suggestions. We thank the reviewer for drawing attention to the anomalous current increase observed at approximately 3.8 V in **Fig. 4a**, which prompted further examination of the reversibility of the Br^0/Br^+ redox process. Our additional experiments and analyses indicate that this feature does not originate from a thermodynamically irreversible reaction, but instead reflects a kinetically controlled electrochemical response. Details are provided below.

We performed additional cyclic voltammetry (CV) measurements at significantly lower scan rates (0.5-5 mV s^{-1}). As shown in **Fig. R26**, a well-defined and symmetric pair of redox peaks corresponding to the Br^0/Br^+ couple is observed at low scan rates, supporting the thermodynamic reversibility of this redox process. In contrast, the apparent current increase and the lack of well-resolved redox peaks at higher scan rates

(10-50 mV s^{-1}) in **Fig. 4a** can be attributed to pronounced kinetic hysteresis. Under fast potential sweeps, charge-transfer and ion insertion/extraction processes lag behind the applied potential, resulting in a pronounced positive shift of the oxidation peak and a negative shift of the reduction peak. Consequently, the peak separation increases, and the full redox features cannot be completely captured within the preset voltage window. This behavior is characteristic of kinetically limited electrode processes rather than thermodynamic irreversibility.

Furthermore, following the reviewer's suggestion, we extended the CV voltage window to 1.8-4.0 V (**Fig. R4**). The resulting CV curves continue to exhibit stable Br^0/Br^+ redox signals, further supporting the reversibility of this redox couple. However, when the voltage is extended beyond approximately 3.9 V, an additional oxidation current associated with chloride oxidation (Cl^-) becomes apparent, corresponding to an undesired side reaction. To avoid interference from this process and to ensure a reliable evaluation of the Br^0/Br^+ redox chemistry, the working voltage window in the main text was therefore restricted to 1.8-3.85 V.

Taken together, the low-scan-rate CV measurements and the extended voltage-window analysis consistently demonstrate that the Br^0/Br^+ redox couple exhibits clear electrochemical reversibility within the selected potential range. The abrupt current increase and peak distortion observed at high scan rates primarily arise from kinetic polarization effects rather than from side reactions or irreversible bromine redox processes. The corresponding discussion and additional figures have been incorporated into the revised manuscript.

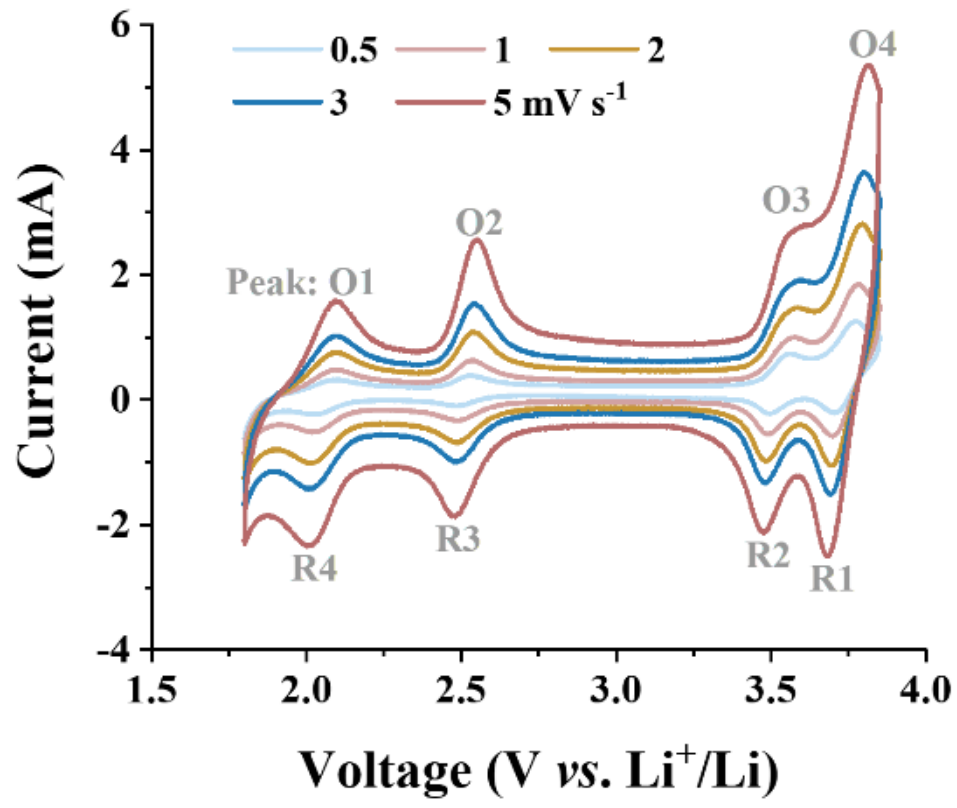

Fig. R26 | CV curves of Li||$EVBr_2$ battery in DDC electrolyte ranging from 0.5 to 5 mV s^{-1}.

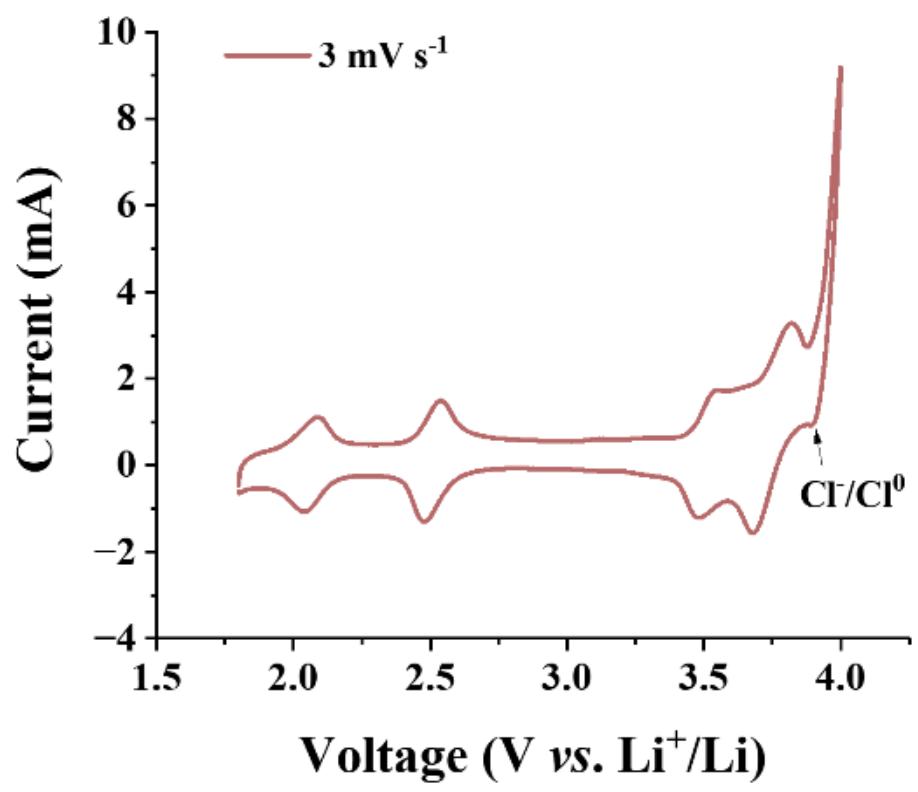

Fig. R4 | CV curve of Li||$EVBr_2$ battery in DDC electrolyte.

Please check the newly added discussion on **Page 8** in the main text, and the newly added **Fig. S15a and Fig. S25** on **Page 10 and 13** in the supplementary information.

Comment 5: *Relatedly, the article argues that four-electron Br transfer has resulted in "activation of a 3.8 V voltage plateau" however the article's CV, charge-discharge curves, and dQ/dV curve appear to have ~3.8 V voltage cut-offs. The fourth dQ/dV peak appears to occur at <3.75 V at all charge rates. Can this please be clarified? If the manuscript is arguing that $EVBr_2$ in DDC enables "reversible hypervalent bromine redox", why was a voltage so proximate to the reversible voltage plateau chosen as the cut-off voltage?*

Response: Thank you for your constructive comments and valuable suggestions. First,

in the original version, the onset voltage of the discharge curve (~3.8 V) was described as the discharge plateau to reflect the practical discharge behavior. Following the reviewer's helpful suggestion, and to ensure a more rigorous correspondence with the electrochemical signatures, we have revised the definition of the plateau voltage in the revised manuscript. Specifically, the plateau is now defined as the potential corresponding to the most pronounced peak in the *dQ/dV* differential curve (~ 3.7 V). This revision improves the consistency between the textual description and the electrochemical data presented in the figures, thereby facilitating clearer interpretation by readers. We appreciate the reviewer's correction.

Second, the choice of a cut-off voltage (3.85 V), which is close to the reversible voltage plateau, was primarily guided by the need to enable reversible Br^0/Br^+ conversion while avoiding parasitic reactions associated with chloride oxidation. Additional wide-window CV measurements and galvanostatic charging tests reveal that further extension of the upper cut-off voltage to 4.0 V gives rise to pronounced chloride-related oxidation currents (**Fig. R4** and **Fig. R38**). Such processes are likely accompanied by chlorine evolution, leakage, and enhanced corrosivity, which would substantially compromise the stability and safety of the battery system. On this basis, the voltage window of 1.8-3.85 V represents an optimal compromise between electrochemical reversibility and operational safety.

We thank the reviewer for the careful reading of the manuscript and for noting the discrepancy between the described voltage plateau and the experimental data. In the revised manuscript, we have revised the description of the voltage plateau and added supporting experimental data to clarify the rationale for the selected voltage window.

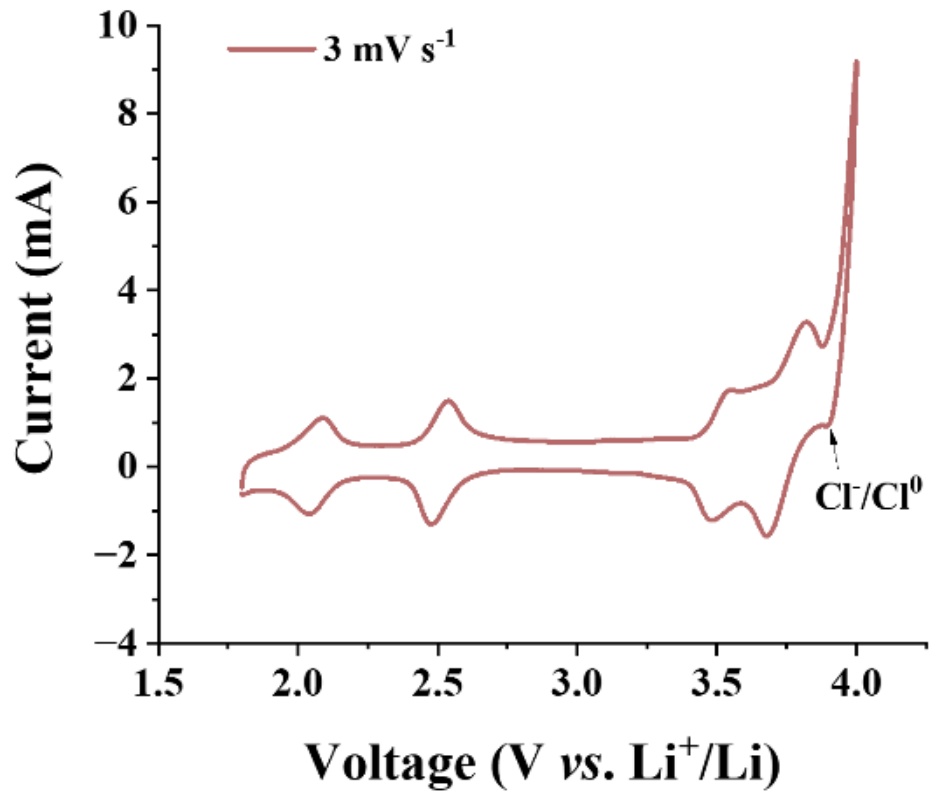

Fig. R4 | CV curve of Li||$EVBr_2$ battery in DDC electrolyte.

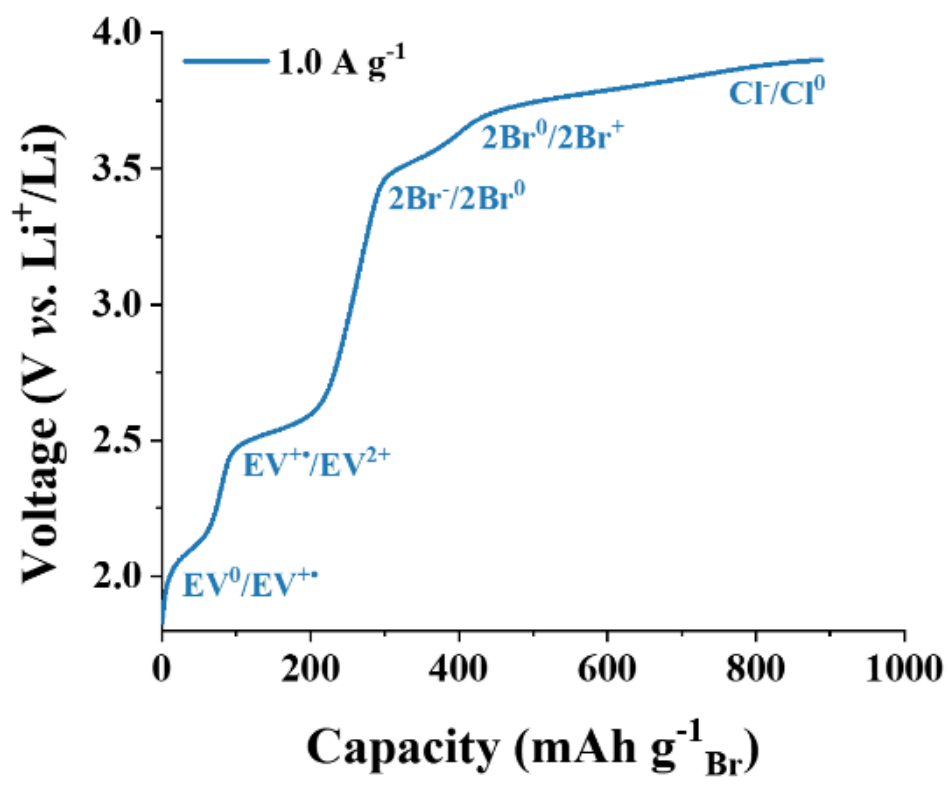

Fig. R38 | The charge curve of Li||$EVBr_2$ battery in DDC electrolyte.

Please check the newly added discussion on **Page 8** in the main text, and the newly added **Fig. S15** on **Page 10** in the supplementary information.

Comment 6: *As seen in* ***Fig. 3b****, there is an extremely sharp drop in capacity from 1.0 to 1.5 A* g^{-1}*. What causes this rapid capacity loss considering that the article argues the material has good reaction kinetics from GITT and in-situ EIS?*

Response: Thank you for your constructive comments and valuable suggestions. Although GITT and *in-situ* EIS measurements indicate favorable intrinsic kinetics, they are typically performed at lower current densities and do not fully reflect the limitations of the entire electrode at high rates. The capacity decline observed at elevated current densities is primarily due to ion diffusion limitations within the electrode, incomplete utilization of the active material, and increased electrode polarization under rapid charge/discharge. Such behavior is commonly observed in similar conversion-type

electrodes and does not contradict the material's intrinsic kinetics [*Nat. Commun.* ***14***, *1856 (2023), J. Am. Chem. Soc.* ***146***, *26855-26862 (2024)*].

Comment 7: *Long-term cycling data in* ***Fig. 3e*** *is only presented as a percentage retention at 3 A* g^{-1}*. Specific capacity should be shown on the y-axis, like in Supplementary* ***Fig.*** *7. Is there long-term cycling data at 1/1.5 A* g^{-1} *to compare to the high-rate cycling data? Additionally, why does dramatic capacity fading appear to occur within the first 50 cycles before it stabilises?*

Response: Thank you for your constructive comments and valuable suggestions. Following the reviewer's suggestion, the y-axis of **Fig. R10** has been updated to specific capacity, consistent with other cycling data, allowing a more direct quantitative comparison. Furthermore, we have added long-term cycling data at 1.0 and 1.5 A g^{-1}, which demonstrate stable capacity retention over 200 cycles (**Fig. R39**).

To elucidate the cycling stability of individual electrochemical processes, long-term cycling data at 5 A g^{-1} were added and the capacity decay of each process was systematically analyzed based on the galvanostatic profiles and corresponding *dQ/dV* curves at different cycle numbers (**Fig. R13**). The initial cycle exhibits four well-defined voltage plateaus, corresponding to the $EV^{2+}/EV^{+\bullet}$, $EV^{+\bullet}/EV^{2+}$, Br^0/Br^-, and Br^+/Br^0 redox processes. These plateaus remain clearly discernible at the 10th cycle with only a slight capacity loss. By the 50th cycle, the plateaus associated with the $EV^{2+}/EV^{+\bullet}/EV^0$ processes show pronounced fading and are largely absent after 1000 cycles (**Fig. R15**). The evolution of the *dQ/dV* curves follows a closely parallel trend: four distinct redox peaks appear in the first cycle and remain evident at the 10th cycle; the peaks related to the $EV^{2+}/EV^{+\bullet}/EV^0$ processes diminish markedly from the 50th cycle onward and nearly disappear after 1000 cycles (**Fig. R14**). These results demonstrate that the capacity fade is predominantly due to the degradation of the $EV^{2+}/EV^{+\bullet}/EV^0$ processes, which is attributed to the volume expansion and structural stress induced by repeated Li^+ insertion/extraction.

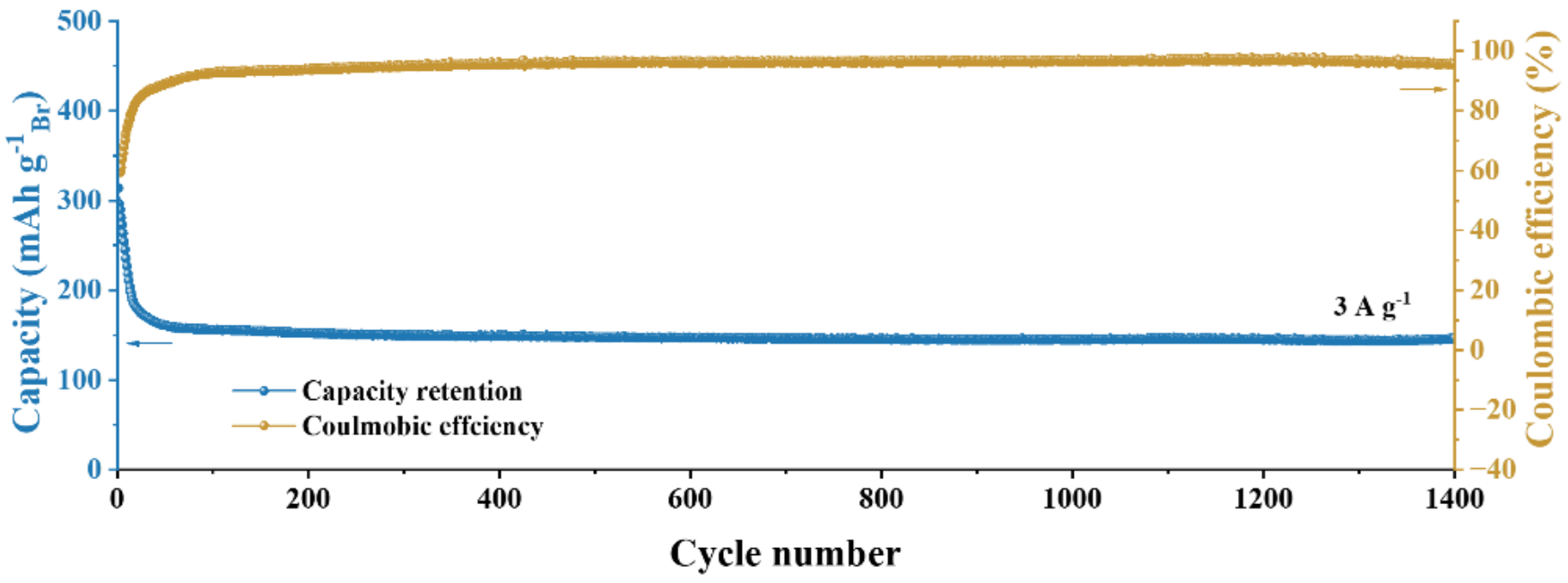

Fig. R10 | Long-term cycling performance of Li||$EVBr_2$ battery in DDC electrolyte at 3.0 A g^{-1}.

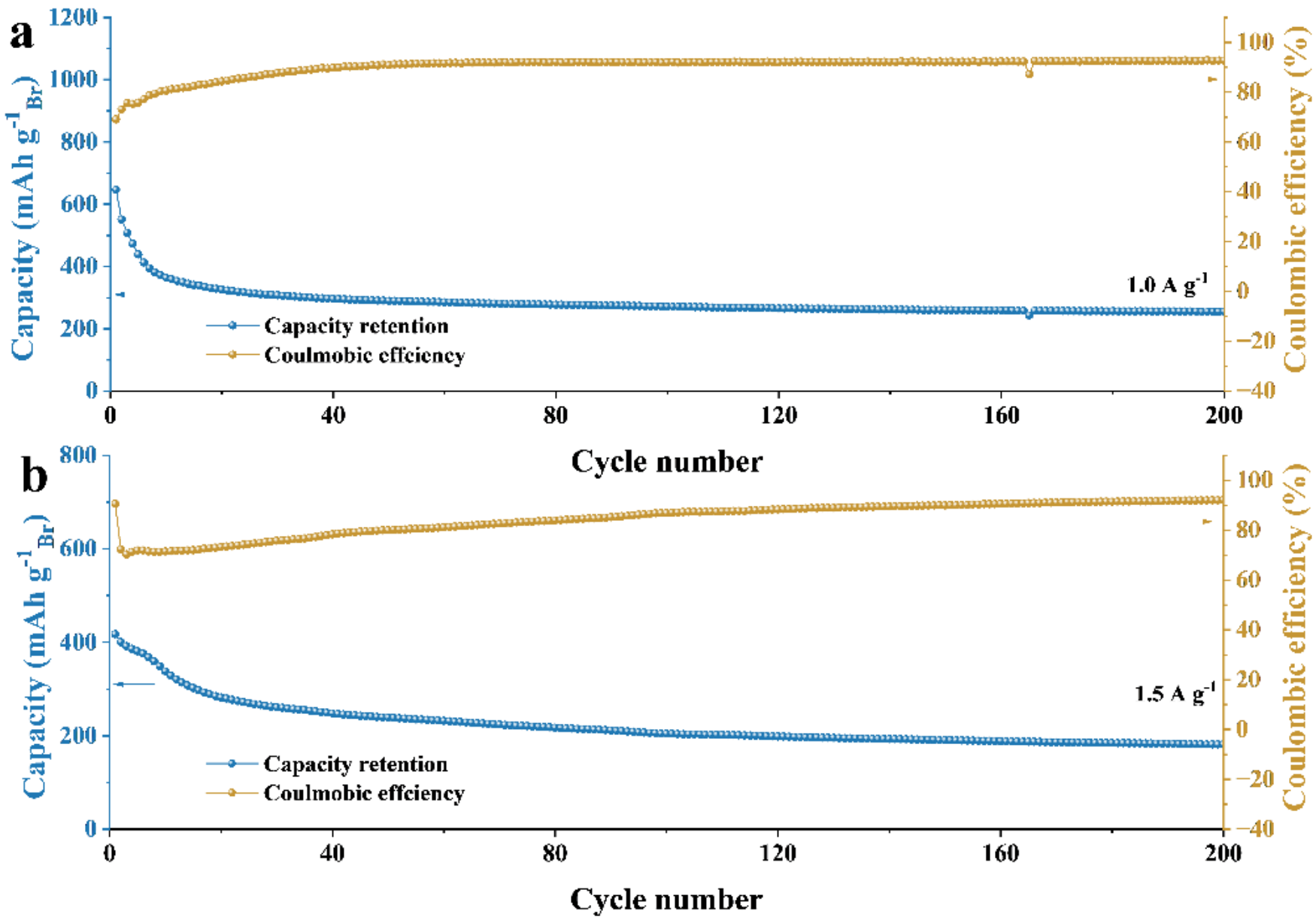

Fig. R39 | Long-term cycling performance of the Li||$EVBr_2$ battery in DDC electrolyte at different current densities. (a) 1.0 A g^{-1}. (b) 1.5 A g^{-1}.

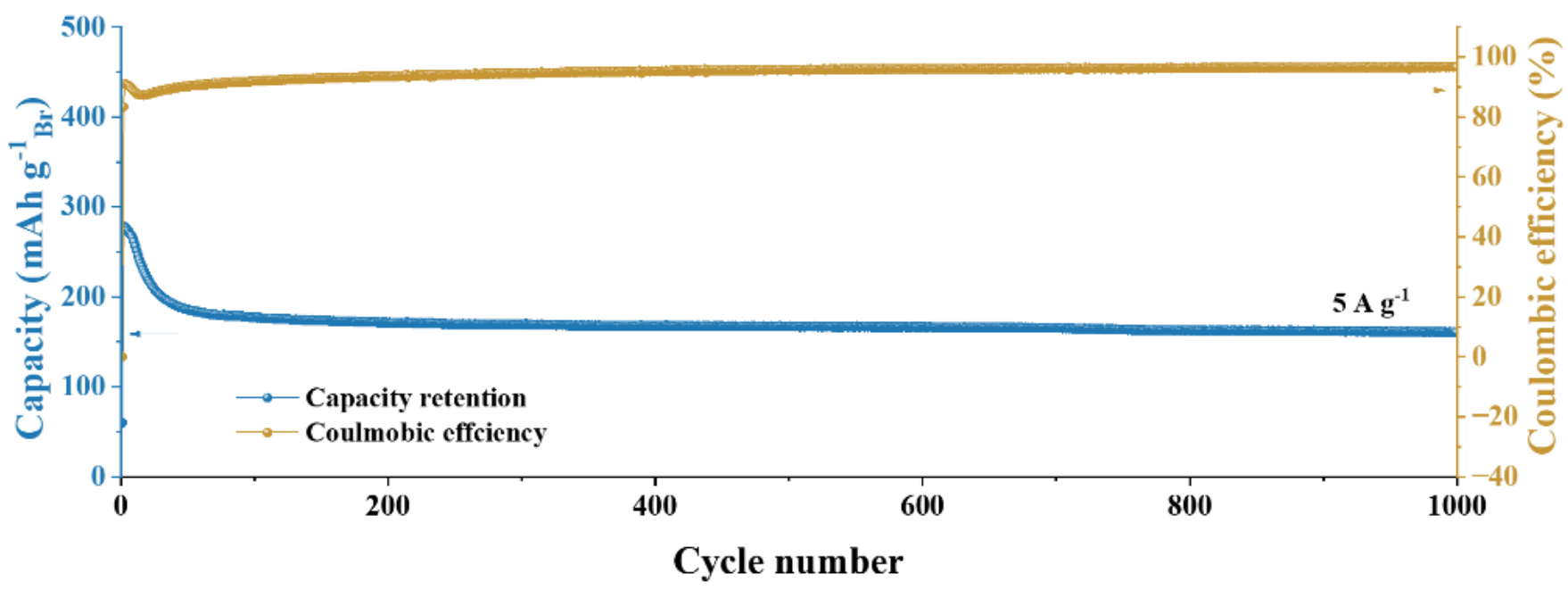

Fig. R13 | Long-term cycling performance of Li||$EVBr_2$ battery in DDC electrolyte at 5.0 A g^{-1}.

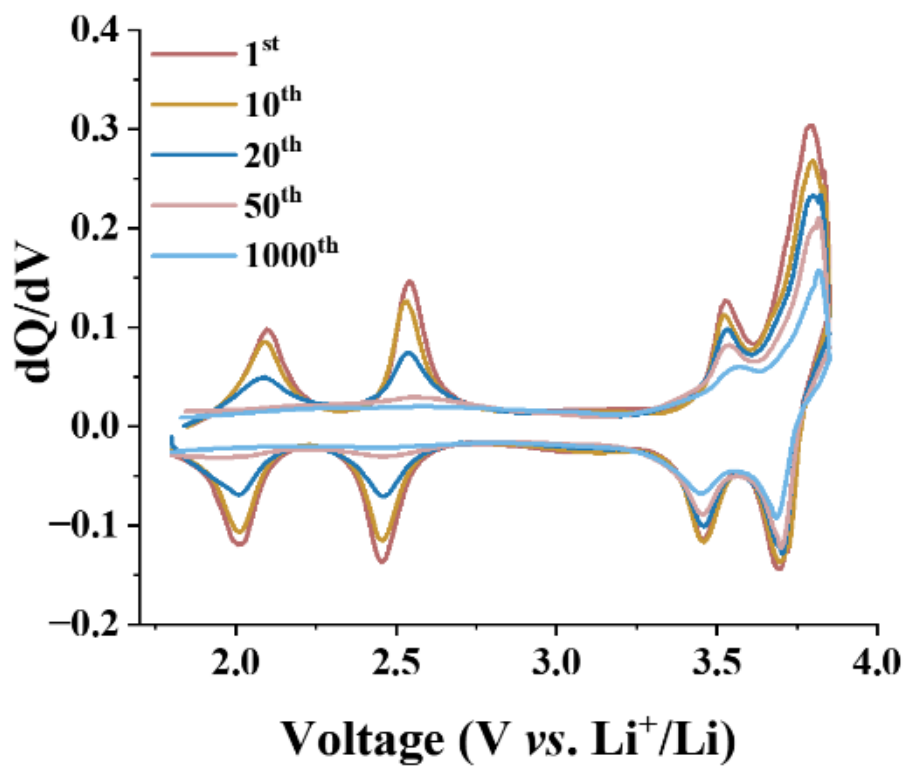

Fig. R14 | The *dQ/dV* curves of Li||$EVBr_2$ battery in DDC electrolyte at 5.0 A g^{-1}.

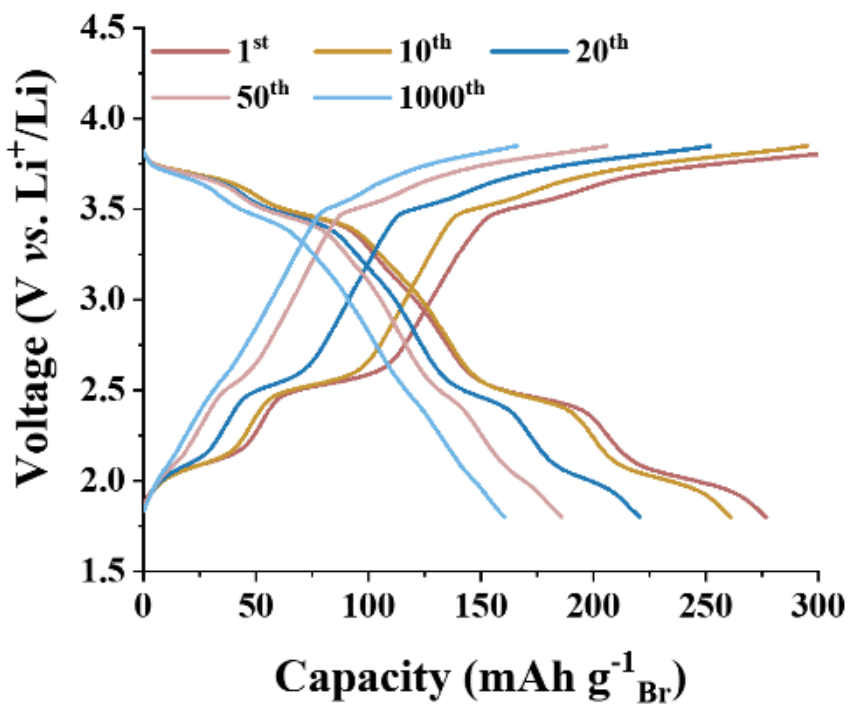

Fig. R15 | GCD curve of Li||$EVBr_2$ battery in DDC electrolyte at 5.0 A g^{-1}.

Please check the newly added **Figs. 4g-h** on **Page 14** and the newly added discussion on **Page 13** in the main text, and the newly added **Figs. S29-30** on **Page 15** in the supplementary information.

Comment 8: *The article states that "Consequently, the realized Li||$EVBr_2$ battery reached 1810 Wh kg^{-1}_{Br} at a current density of 1 A g^{-1}, exceeding all reported Br-based counterparts.15, 31" (Pg 9) However, the literature cited claims to reach an energy density of 2180 Wh kg^{-1}_{Br} at 1A g^{-1}.*

Response: Thank you for your constructive comments and valuable suggestions. We thank the reviewer for pointing out this important discrepancy. Upon careful examination, we find that the difference between the energy density reported in our work (1810 Wh kg^{-1} $_{Br}$) and that reported in the cited literature (2180 Wh kg^{-1} $_{Br}$)

primarily originates from the different mass bases used to define the applied current density. In our study, a current density of 1 A g^{-1} is calculated based on the total mass of the $EVBr_2$ composite, whereas in the cited literature the same nominal current density is normalized to the mass of active bromine only. Consequently, the higher energy density reported in that work is effectively achieved under a lower intrinsic current density when referenced to the bromine mass.

It should also be noted that the cited study primarily focuses on bromine conversion chemistry, whereas our system involves a more complex synergistic mechanism that combines insertion-type redox reactions of the EV^{2+} ligand with hypervalent bromine redox.

We fully agree that the original wording in the manuscript suggesting that our performance "exceeds all reported Br-based counterparts" was insufficiently rigorous. Accordingly, we have revised the relevant statement in the manuscript to ensure a more objective and accurate presentation. We thank the reviewer for this careful and constructive comment.

Please check the improved discussion on **Page 15** in the main text.

Comment 9: *The main text states that, "During the charging phase, the deconvolution of the Br 3d spectrum at 3.0 V revealed two peaks at 68.3 eV and 69.3 eV, which are characteristic of* Br^- *(Supplementary* ***Fig. 15****)." However, Supplementary Fig. 15 does not display the deconvolution of the Br 3d spectrum at 3.0 V.*

Response: Thank you for your constructive comments and valuable suggestions. In the revised manuscript, we have added the Br 3d spectrum and its deconvoluted fitting results at 3.0 V in **Fig. R40**, and have revised the corresponding text to ensure full consistency between the discussion and the newly included data.

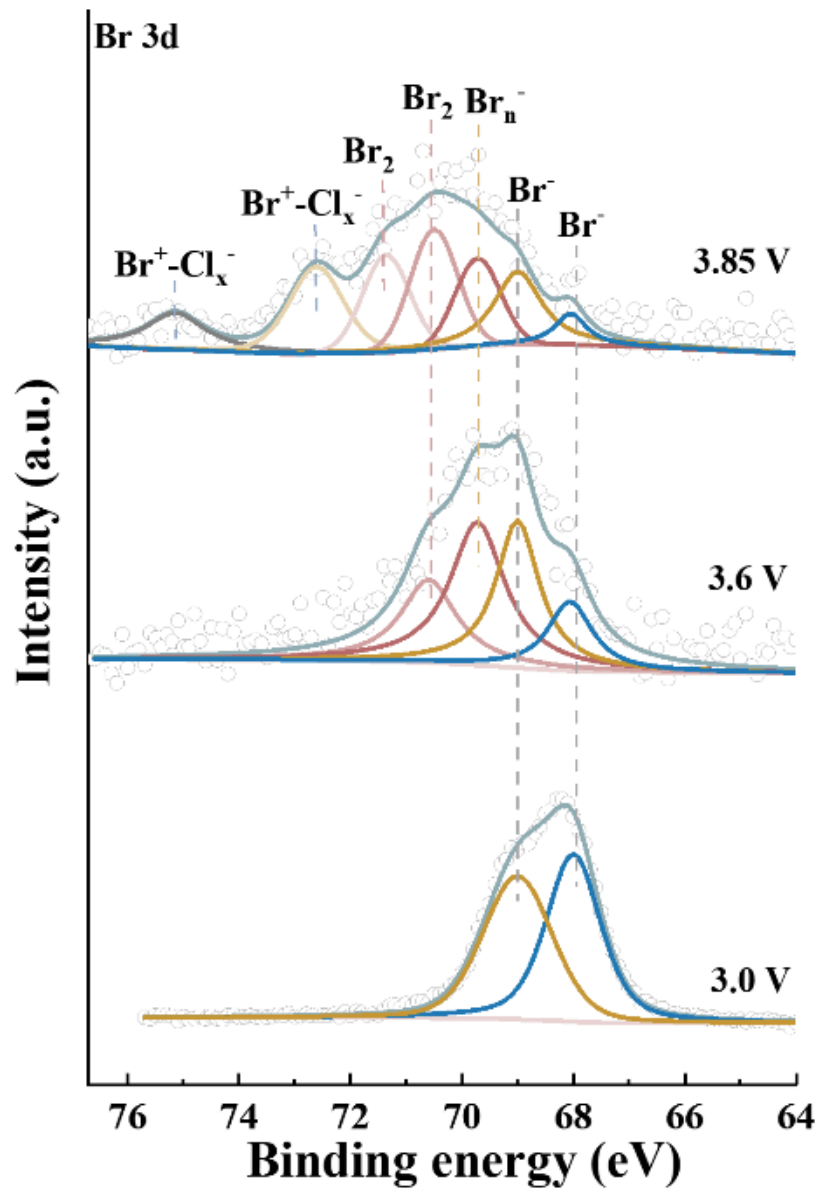

Fig. R40 | High-resolution Br 3d XPS spectra at selected SOC points.

Please check the modified **Fig. 5a** on **Page 17** and the improved discussion on **Page 15** in the main text.

Comment 10: *From the N 1s XPS, the article argues that "The weak residual peak at 398.8 eV originates from a minor amount of $EV^{+\bullet}$ formed via reaction of EV^0 and EV^{2+}.[37, 38, 57]" Based on the literature cited, further oxidation should enable complete oxidation to EV^{2+}. Did the authors try a lower voltage cut-off to confirm this N 1s peak was $EV^{+\bullet}$? If there is residual $EV^{+\bullet}$, could a higher capacity not be achieved?*

Response: Thank you for your constructive comments and valuable suggestions. The electrochemical reduction pathway of EV^{2+} is described in the manuscript. The reduction of EV^{2+} proceeds *via* two consecutive one-electron steps: first to $EV^{+\bullet}$ at ~2.60/2.43 V, accompanied by Li^+ insertion, and then to EV^0 at ~2.14/1.97 V with further Li^+ uptake. The corresponding oxidation follows the reverse pathway, confirming good electrochemical reversibility (**Fig. R25 and Fig. R41**).

The residual $EV^{+\bullet}$ signal observed in the N 1s XPS is not attributed to incomplete electrochemical conversion during battery operation, but is instead ascribed to partial reduction induced by X-ray irradiation under vacuum during *ex-situ* XPS measurements,

a phenomenon that has been commonly reported [*Nat. Commun.* ***16***, *7980 (2025), Green Chem.* ***26***, *10876-10885 (2024)*].

Following the reviewer's suggestion, we have supplemented the XPS measurement of the N 1s region at a lower discharge voltage. Even when discharged to a lower cutoff voltage (1.7 V), the N 1s peak remained nearly identical to that at 1.8 V, confirming that the peak at 398.8 eV corresponds to $EV^{+\bullet}$ (**Fig. R42**).

With respect to whether the presence of residual $EV^{+\bullet}$ implies unrealized capacity, we note that this inference is not directly applicable to the electrochemical operation of the present system. During battery operation, electron transfer is governed by the applied voltage and current, such that the electrode material undergoes complete and reversible redox transitions within the relevant potential windows, thereby delivering its theoretical capacity. Consequently, the small amount of $EV^{+\bullet}$ detected by ex situ XPS does not reflect the electrochemical state of the electrode during cell operation and therefore does not impose a limitation on the practically achievable capacity.

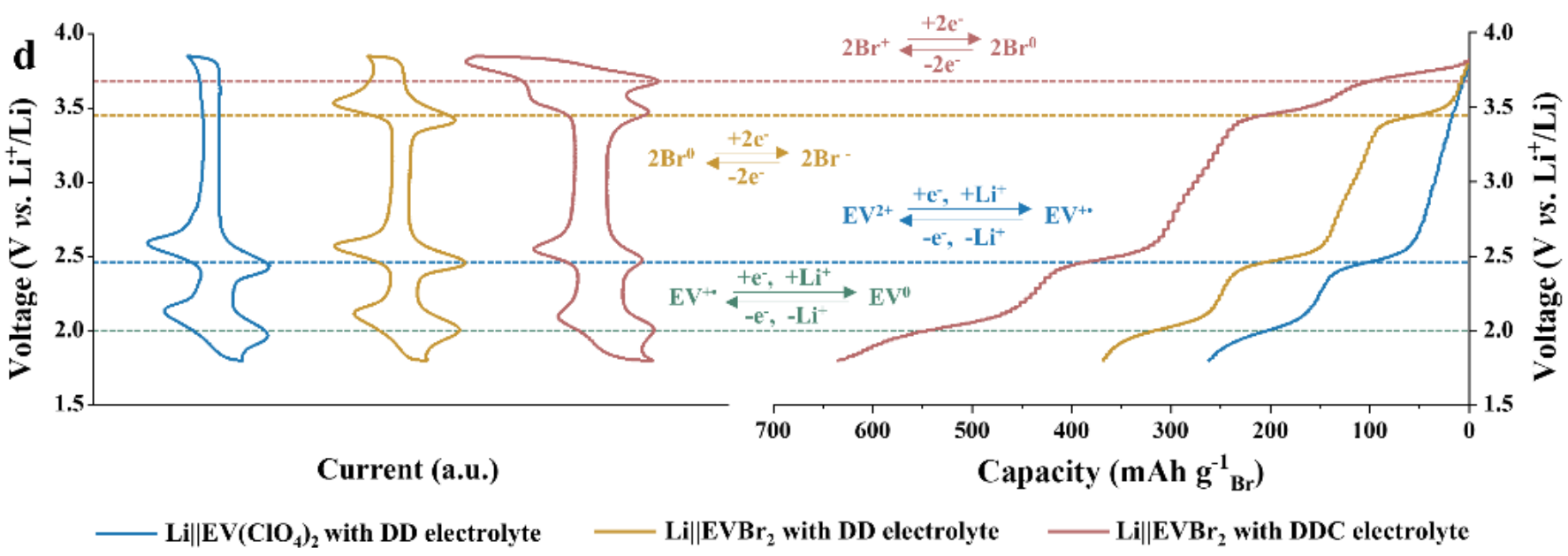

Fig. R41 | CV and discharge curves of $EV(ClO_4)_2$ in DD electrolyte (blue), $EVBr_2$ in DD electrolyte (yellow), and $EVBr_2$ in DDC electrolyte (pink).

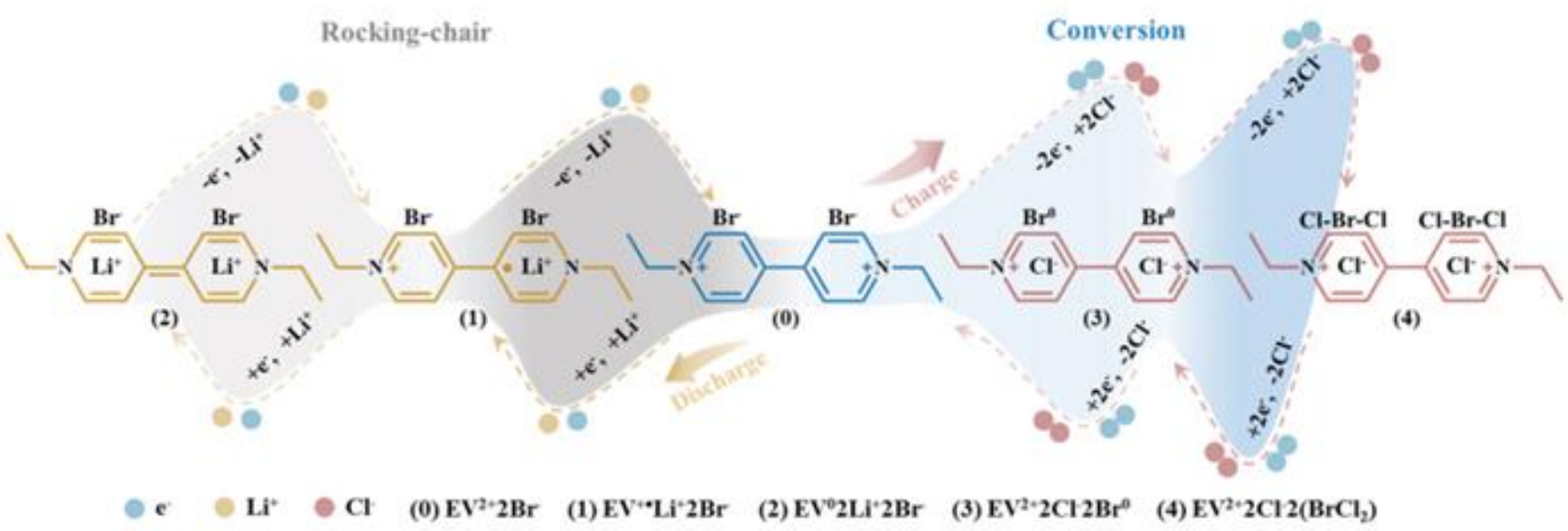

Fig. R25 | Diagram of six-electron reaction mechanism of Li||$EVBr_2$ battery.

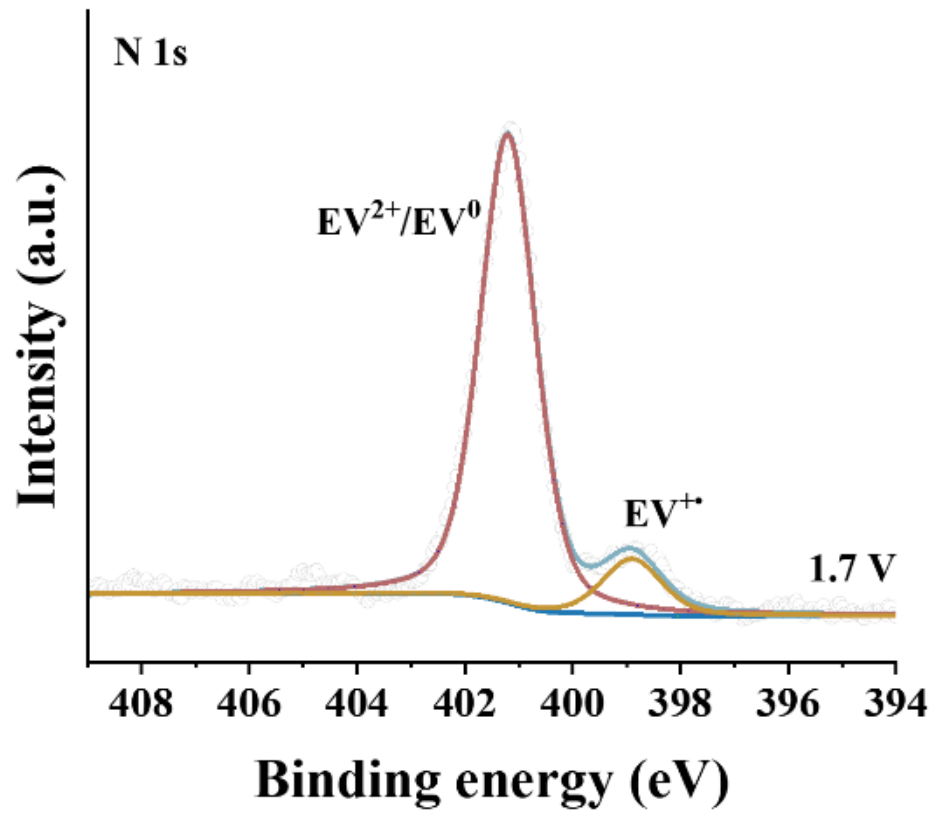

Fig. R42 | High-resolution N 1s XPS spectra at 1.7 V.

Please check the modified **Fig. 2d** on **Page 9** and **Fig. 6d** on **Page 20** in the main text, and the newly added **Fig. S36** on **Page 17** in the supplementary information.

Comment 11: *Self-discharge is widely recognised as a challenge for Br-based batteries, as identified by comments on the thermodynamic instability and volatility of bromine in the introduction. The results on improved shelf-life were clearly presented to address this challenge. However, shelf-life and self-discharge are not perfectly correlated. Could these rults not be reinforced by a comparison of the self-discharge rates for lithium-bromine batteries before and after ethyl-viologen modification?*

Response: Thank you for your constructive comments and valuable suggestions. To directly evaluate how ethyl viologen mitigates self-discharge, a control was performed with a conventional KBr cathode (KBr, Ketjen Black, and PVDF in a 6:3:1 mass ratio using NMP). Li||$EVBr_2$ and Li||KBr batteries were assembled, charged to 3.85 V at 1 A g^{-1}, allowed to stand for 2 hours, and discharged at the same current (**Fig. R43**).

Following a 2 hours rest, the Li||$EVBr_2$ battery maintained a higher voltage (2.7 V *vs.* 2.5 V for Li||KBr), demonstrating its superior resistance to self-discharge. This advantage was further confirmed upon discharge, with the Li||$EVBr_2$ battery delivering 49% capacity retention, vastly outperforming the control battery (14%). Together, these results conclusively show that the ethyl viologen framework suppresses bromine

shuttling and side reactions, thereby greatly improving charge retention.

We thank the reviewer for this insightful suggestion, which has strengthened the rigor and completeness of the manuscript.

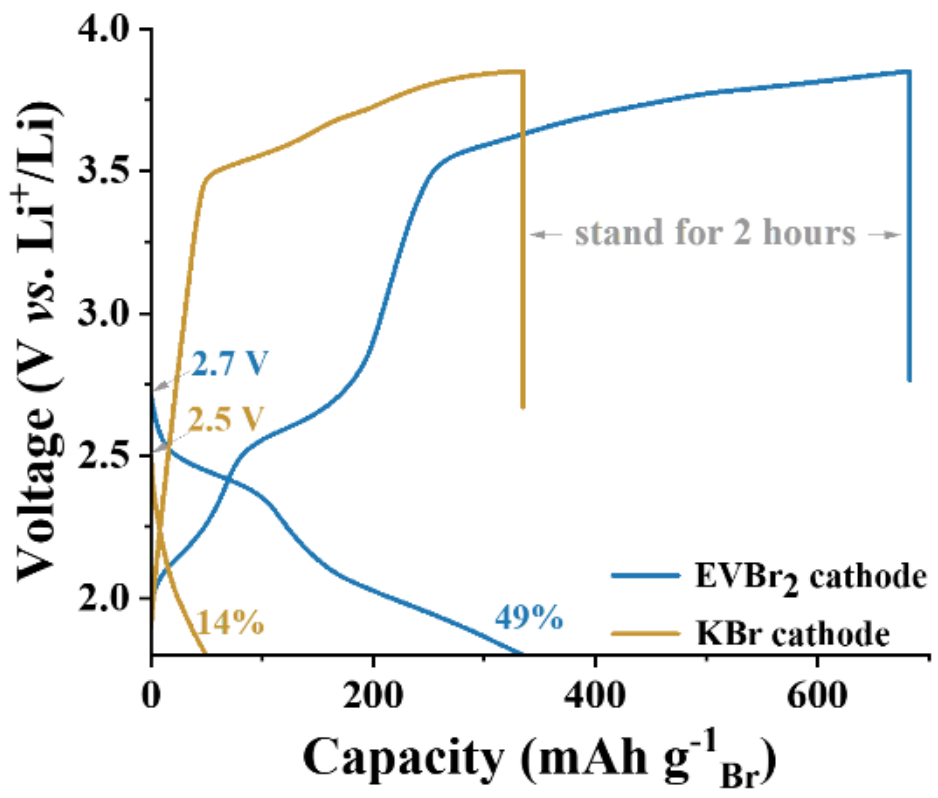

Fig. R43 | Comparison of the self-discharge behaviors for Li||$EVBr_2$ and Li||KBr batteries after charging at 1 A g^{-1}.

Please check the newly added **Fig. S32** on **Page 16** in the supplementary information and the improved discussion on **Page 13** in the main text.

Comment 12: *The introduction provides quality background info on the strengths and limitations of bromine for halogen batteries, but only briefly mentions lithium-bromine batteries and the role of ligands. Background on Cl^- ligands, work done to stabilise two electron transfer lithium-bromine, and a more rigorous explanation of how "ligands simultaneously functioning as redox-active charge reservoirs via rocking-chair-type mechanism (**Fig. 1c**)" would help provide theoretical explanations and clarify decisions in the article (e.g. the role of LiCl in DDC).*

Response: Thank you for your constructive comments and valuable suggestions. We fully agree that the background in the Introduction should be further strengthened to provide a more solid theoretical foundation for the mechanistic discussion and experimental design presented in this work.

In response to the reviewer's constructive comments, we have substantially revised and expanded the Introduction to provide a more rigorous theoretical foundation.

The key additions are summarized as follows:

(i) **Expanded background on viologen ligands.** Viologens are classic organic electroactive materials with rigid frameworks. Their structural characteristics enable highly reversible two-electron redox processes ($EV^{2+}/EV^{+\bullet}/EV^{0}$), fast kinetics, and good stability. These properties underpin their applications in electrochromic devices, redox-flow batteries, and organic electrodes. Critically, their dicationic character confers strong electrostatic affinity for polybromide intermediates, making them ideal scaffolds for suppressing shuttle effects in Br-based electrochemistry.

(ii) **Critical discussion of two-electron transfer in Li-Br batteries.** Current research on Br redox chemistry is largely confined to two-electron transfer reactions. In contrast, four-electron processes, which provide a pathway to simultaneously higher voltage and greater theoretical capacity, remain critically underexplored. The parasitic disproportionation of hypervalent Br^{+} species imposes a fundamental limitation on electrochemical reversibility.

(iii) **Theoretical explanation of Cl^{-} as a stabilizing ligand.** The instability of Br^{+} species stems from their strong Lewis acidity and electron-deficient nature, making them prone to reduction. Stabilization *via* coordination with electron donors is therefore essential, yet halide anions differ drastically in effectiveness. Fluoride (F^{-}) binds too strongly, causing overstabilization or unselective oxidation, while I^{-} acts as a reductant and induces decomposition. Cl^{-}, by contrast, provides a favorable electronic and steric balance. Its lone-pair electrons delocalize the positive charge and improve thermodynamic stability, while polyhalide assembly affords crucial steric protection.

We believe that these additions strengthen the logical coherence of the manuscript and help readers better appreciate the novelty and significance of this work. We thank the reviewer again for this constructive and insightful guidance.

Please check the improved discussion on **Pages 3-4** in the main text.

Comment 13: *The SEM image appears to be missing from Supplementary Fig. 1, with only EDS mapping provided.*

Response: Thank you for your constructive comments and valuable suggestions. The SEM image has now been added to **Fig. S1** in the revised manuscript (**Fig. R44**).

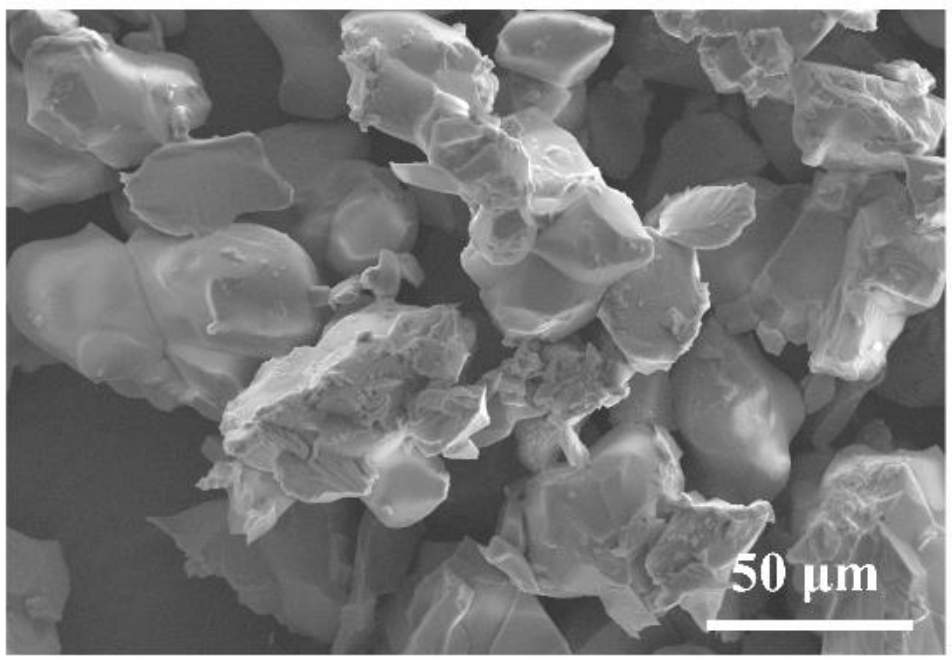

Fig. R44 | SEM image of $EVBr_2$.

Please check the modified **Fig. S1** on **Page 5** in the supplementary information and the improved discussion on **Page 5** in the main text.

Comment 14: *The citations associated with* ***Fig. 3g*** *are not clear at first glance.*

Response: Thank you for your constructive comments and valuable suggestions. We have improved this in the revised manuscript by adding a detailed performance comparison **Table R1** in the Supplementary Information and by directly citing the key references in **Fig. R35**, thereby ensuring that the sources of the compared data are clearly and readily identifiable.

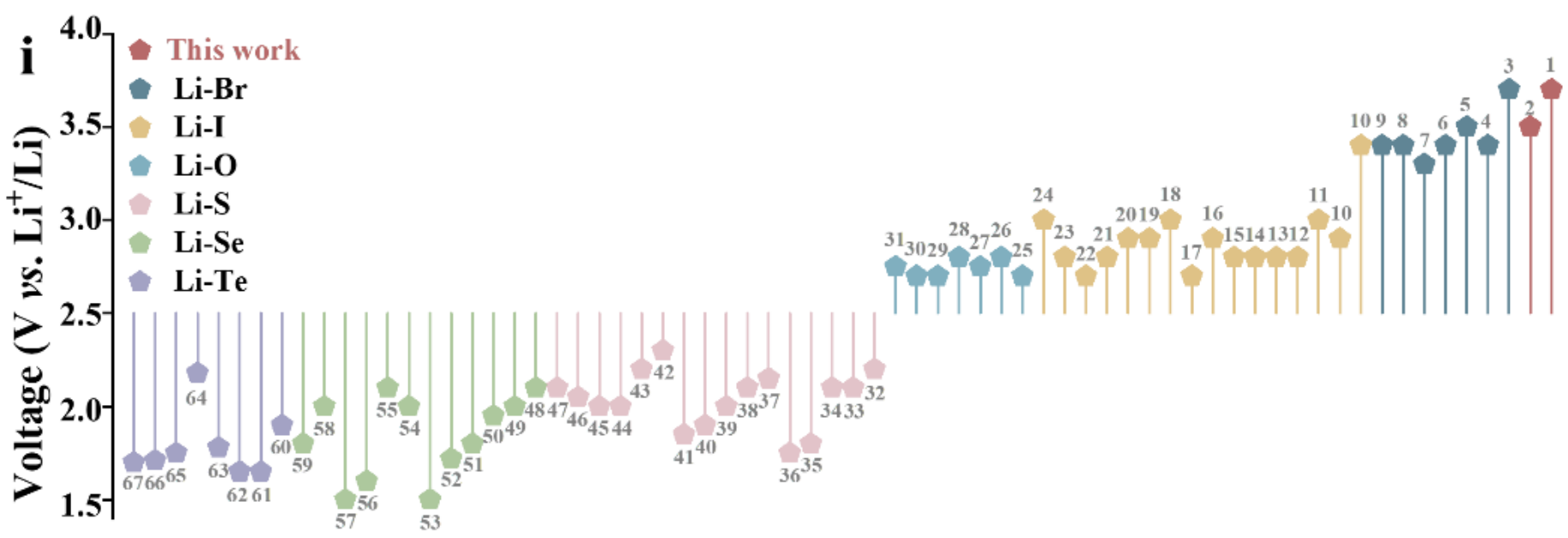

Fig. R35 | Performance comparison on output voltage between this work and reported conversion-type Li batteries.

Table R1. Comparison of the electrochemical performance between this work and

state-of-the-art lithium-ion batteries.

Battery type	Number	Anode	Plateau voltage (V)	Reference
Br	1	Li	3.7	**This work**
	2	Li	3.5	**This work**
	3	Li	3.7	*Sci. Adv.*[7]
	4	Li	3.4	*Adv. Mater.*[8]
	5	Li	3.5	*Joule*[9]
	6	Li	3.4	*J. Mater. Chem. A*[10]
	7	Li	3.3	*J. Energy Chem.*[11]
	8	Li	3.4	*Adv. Energy Mater.*[12]
	9	Li	3.4	*J. Power Sources*[13]
I	10	Li	3.4	*J. Am. Chem. Soc.*[14]
	10	Li	2.9	*J. Am. Chem. Soc.*[14]
	11	Li	3	*Adv. Mater.*[14]
	12	Li	2.8	*Energy Storage Mater.*[15]
	13	Li	2.8	*ACS Nano*[16]
	14	Li	2.8	*Angew. Chem. Int. Ed.*[17]
	15	Li	2.8	*Energy Environ. Sci.*[18]
	16	Li	2.9	*Adv. Funct. Mater.*[19]
	17	Li	2.7	*Carbon Energy*[20]
	18	Li	3	*Nat. Commun.*[21]
	19	Li	2.9	*J. Mater. Chem. A*[22]
	20	Li	2.9	*J. Power Sources*[23]
	21	Li	2.8	*ACS Nano*[16]
	22	Li	2.7	*J. Mater. Chem. A*[24]
	23	Li	2.8	*J. Mater. Chem. A*[25]
	24	Li	3	*Energy Storage Mater.*[26]
O	25	Li	2.7	*Nat. Mater.*[27]
	26	Li	2.8	*Chem. Rev.*[28]
	27	Li	2.75	*Nat. Commun.*[29]
	28	Li	2.8	*Nat. Commun.*[30]
	29	Li	2.7	*Adv. Mater.*[31]
	30	Li	2.7	*Adv. Energy Mater.*[32]
	31	Li	2.75	*Angew. Chem. Int. Ed.*[33]
S	32	Li	2.2	*Joule*[34]
	33	Li	2.1	*Adv. Sci.*[35]
	34	Li	2.1	*Adv. Energy Mater.*[36]

	35	Li	1.8	*Nano-Micro Lett.*[37]
	36	Li	1.75	*Adv. Mater.*[38]
	37	Li	2.15	*Energy Environ. Sci.*[39]
	38	Li	2.1	*Angew. Chem. Int. Ed.*[40]
	39	Li	2	*Angew. Chem. Int. Ed.*[41]
	40	Li	1.9	*Nat. Mater.*[42]
	41	Li	1.85	*Energy Storage Mater.*[43]
	42	Li	2.3	*ACS Nano*[44]
	43	Li	2.2	*Adv. Mater.*[45]
	44	Li	2	*Adv. Mater.*[46]
	45	Li	2	*Adv. Funct. Mater.*[47]
	46	Li	2.05	*Adv. Sci.*[48]
	47	Li	2.1	*Adv. Funct. Mater.*[49]
Se	48	Li	2.1	*J. Mater. Chem. A*[50]
	49	Li	2	*Adv. Mater.*[51]
	50	Li	1.95	*Joule*[52]
	51	Li	1.8	*Adv. Funct. Mater.*[53]
	52	Li	1.72	*J. Mater. Chem. A*[54]
	53	Li	1.5	*ACS Nano*[55]
	54	Li	2	*Energy Environ. Sci.*[56]
	55	Li	2.1	*Adv. Mater.*[57]
	56	Li	1.6	*Nano Energy*[58]
	57	Li	1.5	*Adv. Energy Mater.*[59]
	58	Li	2	*Energy Environ. Mater.*[60]
	59	Li	1.8	*Chem. Eng. J.*[61]
Te	60	Li	1.9	*Energy Storage Mater.*[62]
	61	Li	1.65	*ACS Nano*[63]
	62	Li	1.65	*J. Alloy. Compd.*[64]
	63	Li	1.78	*Nano Energy*[65]
	64	Li	2.18	*Energy Storage Mater.*[66]
	65	Li	1.75	*Carbon*[67]
	66	Li	1.71	*J. Power Sources*[68]
	67	Li	1.7	*Nano Energy*[69]

Please check the modified **Fig. 4i** on **Page 14** in the main text and the newly added **Table S1** on **Pages 21-22** in the supplementary information.

To Reviewer #6:

***There has been significant interest in new cathode materials for reversible Li-ion batteries.** This paper explores a (relatively) novel concept in a $6e^-$ reduction ethyl viologen dibromide cathode.*

Response: We thank the reviewer for acknowledging the broad interest in novel cathode materials and for recognizing the relatively new concept of the six-electron reduction process based on ethyl viologen dibromide introduced in this work. We agree that exploring such multi-electron redox systems represents a promising direction for advancing high-energy-density rechargeable batteries. We are also grateful for the insightful comments provided, which offer significant value for further enhancing the quality of the manuscript.

Comment 1: *Bromine and viologens have been considered for lithium ion battery cathode reactions for some time. A cursory search for "ethyl viologen lithium bromine battery" returns over 600 somewhat related publications since 2021. The introduction includes some information related to the state of the art but the novelty of this approach is not clear relative to other related publications included several (ACS Appl. Energy Mater. 2024, 7, 18, 7615-7623) that were referenced by the authors. In my view, the introduction is incomplete in terms of outlining the novelty and "state of the art."*

10.1016/j.cej.2023.142624

10.1038/s41467-025-63272-x

10.1039/D4CS00585F

10.1021/acsaem.3c02025

Response: Thank you for your constructive comments and valuable suggestions. We have implemented substantial revisions to the Introduction in response to the reviewer's suggestion. Specifically, we have systematically incorporated and discussed the literature highlighted by the reviewer, added a dedicated discussion on multi-electron redox storage mechanisms in viologen-based compounds, and updated the overview of recent progress and remaining challenges in lithium-bromine battery systems. By

strengthening the background and state-of-the-art discussion, the revised Introduction more clearly identifies existing research gaps and highlights the novelty of this work in enabling cooperative multi-electron transfer between organic ligands and bromine.

The specific changes are as follows: Viologens are classic organic electroactive materials with rigid frameworks. Their structural characteristics enable highly reversible two-electron redox processes ($EV^{2+}/EV^{+\bullet}/EV^{0}$), fast kinetics, and good stability. These properties underpin their applications in electrochromic devices, redox-flow batteries, and organic electrodes. Critically, their dicationic character confers strong electrostatic affinity for polybromide intermediates, making them ideal scaffolds for suppressing shuttle effects in Br-based electrochemistry. Current research on Br redox chemistry is largely confined to two-electron transfer reactions. In contrast, four-electron processes, which provide a pathway to simultaneously higher voltage and greater theoretical capacity, remain critically underexplored. The parasitic disproportionation of hypervalent Br^{+} species imposes a fundamental limitation on electrochemical reversibility. The instability of Br^{+} species stems from their strong Lewis acidity and electron-deficient nature, making them prone to reduction. Stabilization *via* coordination with electron donors is therefore essential, yet halide anions differ drastically in effectiveness. Fluoride (F^{-}) binds too strongly, causing overstabilization or unselective oxidation, while I^{-} acts as a reductant and induces decomposition. Cl^{-}, by contrast, provides a favorable electronic and steric balance. Its lone-pair electrons delocalize the positive charge and improve thermodynamic stability, while polyhalide assembly affords crucial steric protection.

Please check the improved discussion on **Pages 3-4** and the newly added **references 34-37** on **Page 25** in the main text.

Comment 2: *The stability and CE @ 3A/g in Figure 3 are very good. It would be helpful to know this rate relative to the total cathode capacity. Likewise it would be useful to know the current density. How does the CE and capacity retention perform at higher current densities / rates?*

Response: Thank you for your constructive comments and valuable suggestions. In response to this suggestion, we have added long-term cycling data at 5 A g^{-1} in the revised manuscript (**Fig. R13**). The battery maintains good capacity retention and high Coulombic efficiency over 1000 cycles at this elevated rate, further confirming the stability of the material under high current densities.

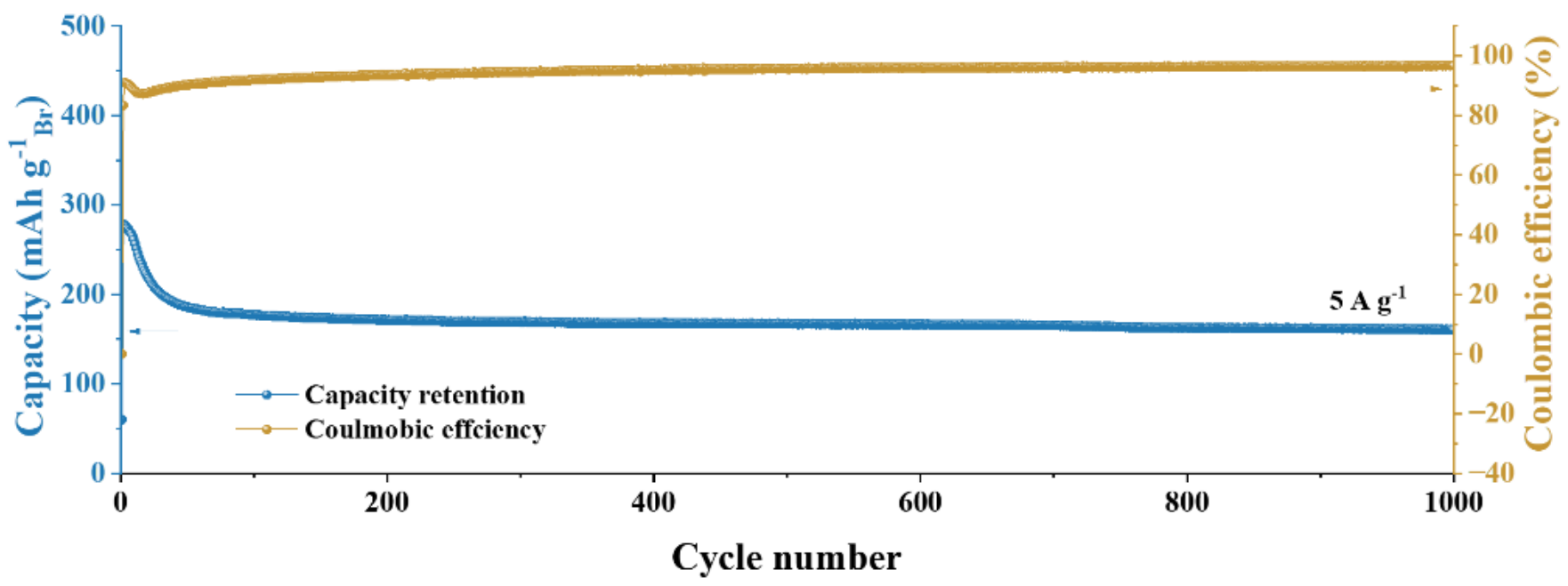

Fig. R13 | Long-term cycling performance of Li||$EVBr_2$ battery in DDC electrolyte at 5.0 A g^{-1}.

Please check the newly added **Fig. S29c** on **Page 15** in the supplementary information.

Comment 3: *At a practical level, the problem of multiple discharge potentials (Figure 3 b and c) limit the usefulness of such a cathode. Further, while these materials may be earth-abundant (lower cost than conventional Li-ion cathodes), the EV is organic and is subject to degradation over time (particularly at higher temperatures and higher current densities).*

Response: Thank you for your constructive comments and valuable suggestions. We fully agree that the multiple discharge plateaus increase voltage-profile complexity and challenge precise state-of-charge estimation in conventional battery management systems (BMS). Addressing this will be critical for practical application, and we plan to explore electrolyte engineering and interface modulation to flatten the profile. It is important to emphasize, however, that the multi-step redox process was deliberately designed to achieve a fundamental leap in energy density. By activating the novel

Br^0/Br^+ couple alongside the conventional Br^-/Br^0 step, we introduce a higher discharge plateau (~3.7 V), effectively representing a 34% increase over existing systems. Furthermore, the EV^{2+} ligand acts dually as a redox-active center and a bromine-confining host, which robustly suppresses polybromide shuttle. This synergistic mechanism represents the core innovation of our work (**Fig. R45**).

We fully acknowledge your concern regarding the long-term stability of the organic electrode material under harsh conditions (e.g., elevated temperature and high current density). As with many organic active materials, this remains a key challenge for practical implementation. To address this, our future efforts will focus on two complementary paths: first, to enhance cycling stability under demanding conditions through molecular design, electrolyte engineering, and interface control; and second, to harness the tunable degradation characteristics of such materials, transforming a stability challenge into a unique application opportunity in areas like biodegradable electronics and transient energy storage.

Once again, we wish to express our deep gratitude for your insightful comments. Your questions have not only helped to clarify the design rationale and specific aims of this work, but have also provided valuable guidance for our future research directions.

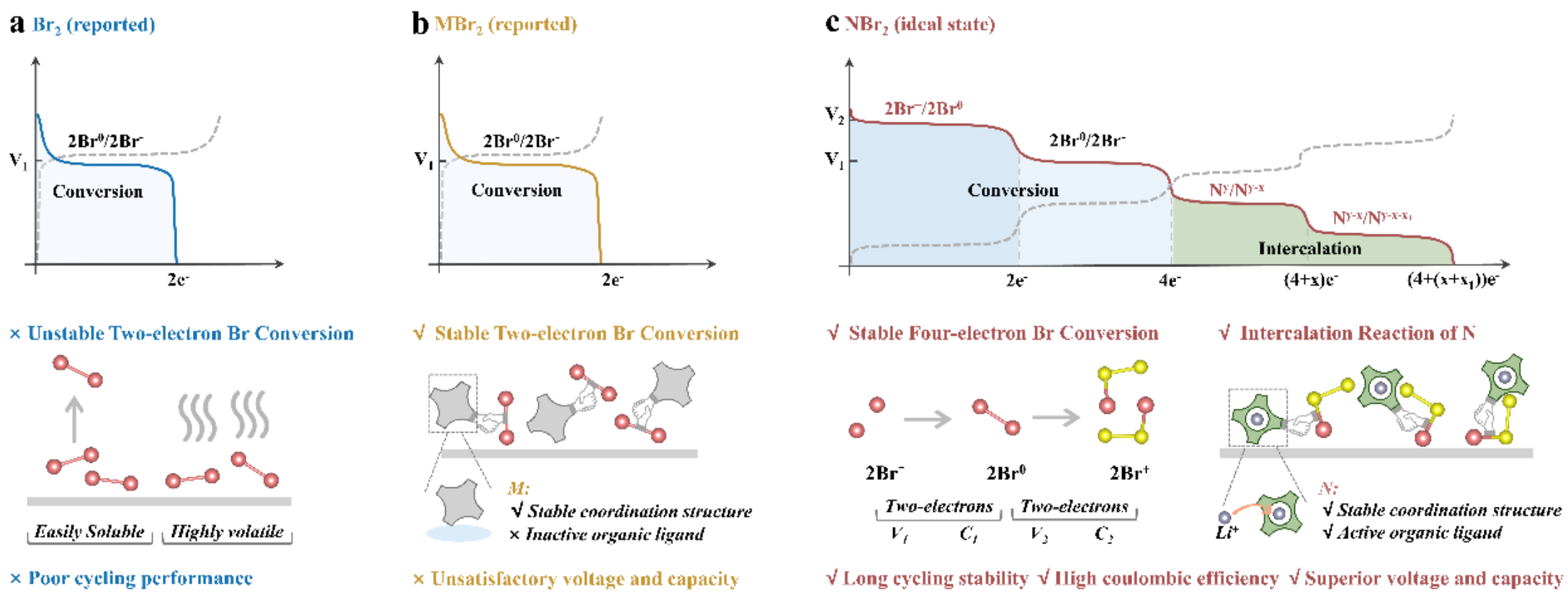

Fig. R45 | Schematic illustration of the challenges and ideal optimization strategies for advanced bromine batteries.

Response to Reviewers

Dear Reviewers:

Thanks a lot for your constructive comments and suggestions about our manuscript entitled "***Coordination electrochemistry taming reversible hypervalent bromine redox for energetic six-electron-transfer lithium-bromine battery***" (NCOMMS-25-81076A). Those comments are valuable and helpful for improving the quality of our paper and promoting our further research work. We have studied your comments very carefully and substantially revised our manuscript in the hope of addressing your concerns and meeting the high standards of **Nature Communications.** The comments are addressed point-by-point below, and changes have been highlighted in the revised main text and supporting information.

REVIEWER COMMENTS

To Reviewer #1:

Well update. It can be recommended for publication now.

Response: Thank you for recognizing our work and recommending publication.

To Reviewer #2:

My questions have been well responded. Thank the authors.

Response: Thank you for recognizing our work and recommending publication.

To Reviewer #3:

I co-reviewed this manuscript with one of the reviewers who provided the listed reports. This is part of the Nature Communications initiative to facilitate training in peer review and to provide appropriate recognition for Early Career Researchers who co-review manuscripts.

Response: Thank you for recognizing our work and recommending publication.

To Reviewer #4:

The paper can be accepted for publication; however, several modifications could further improve the manuscript.

Response: Thank you for recognizing our work and recommending publication.

1) The analysis of SEI thickness in Supplementary Figures 21 and 23 is a little tenuous, particularly for the CEI due to the low difference in contrast and lack of variance in crystallinity. Further images reproducing this thickness or alternative techniques such as XPS depth profiling would significantly improve the confidence of this finding.

Response: Thank you for your constructive comments and valuable suggestions. Following your suggestion, we have performed additional TEM imaging and XPS depth-profiling analyses. First, as shown in **Figs. R1** and **R2**, we have provided TEM images of the SEI and CEI obtained from different batches of samples. The measured thicknesses consistently fall within the originally reported ranges (approximately 40

nm for the SEI and 3-4 nm for the CEI), confirming the good reproducibility of the thickness measurements. Furthermore, XPS depth profiling was carried out to cross-validate the interfacial thickness from the perspective of compositional evolution. With increasing sputtering time, both the SEI and CEI exhibit clear depth-dependent compositional changes. The SEI is mainly composed of reduction products of the electrolyte, including LiF, Li_3N (originating from $LiNO_3$ decomposition), Li_2O/Li_2CO_3, and sulfur-containing species (e.g., Li_2S) derived from the reductive degradation of $TFSI^-$, with inorganic components gradually dominating after prolonged sputtering (**Fig. R3**). In contrast, the CEI consists primarily of oxidation products, including LiF, nitrogen-containing oxides (e.g., nitrates/nitrites) formed *via* oxidative decomposition of $LiNO_3$, and high-valence sulfur-oxygen species (e.g., sulfates) generated from the oxidative breakdown of $TFSI^-$ at high potentials (**Fig. R4**).

Collectively, multi-region TEM statistics combined with XPS depth profiling provide mutually corroborating evidence for the thickness of the SEI and CEI from both morphological and compositional perspectives, thereby enhancing the reliability of the interfacial thickness analysis.

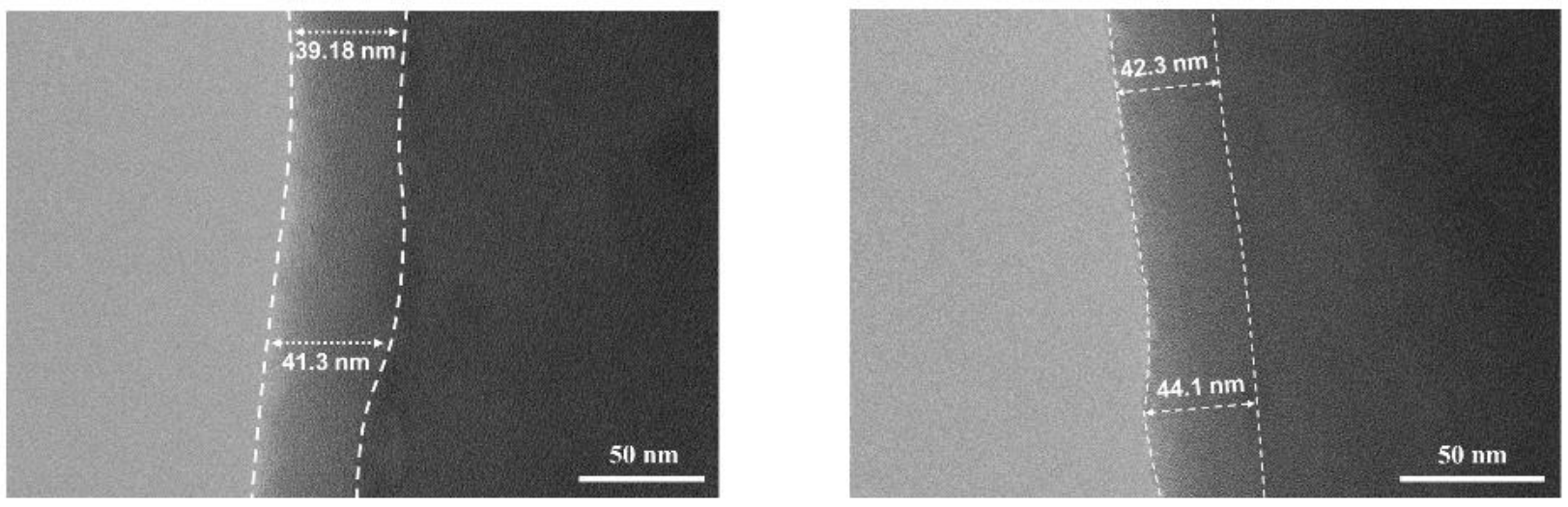

Fig. R1 | TEM image of the SEI layer.

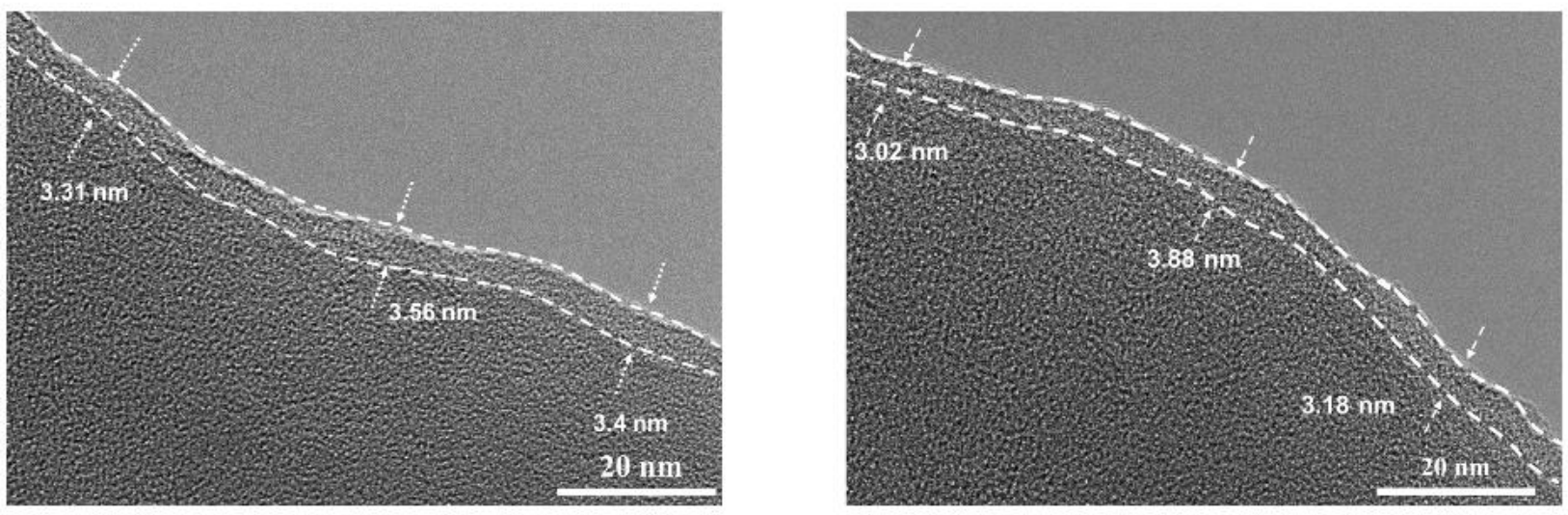

Fig. R2 | TEM image of the CEI layer.

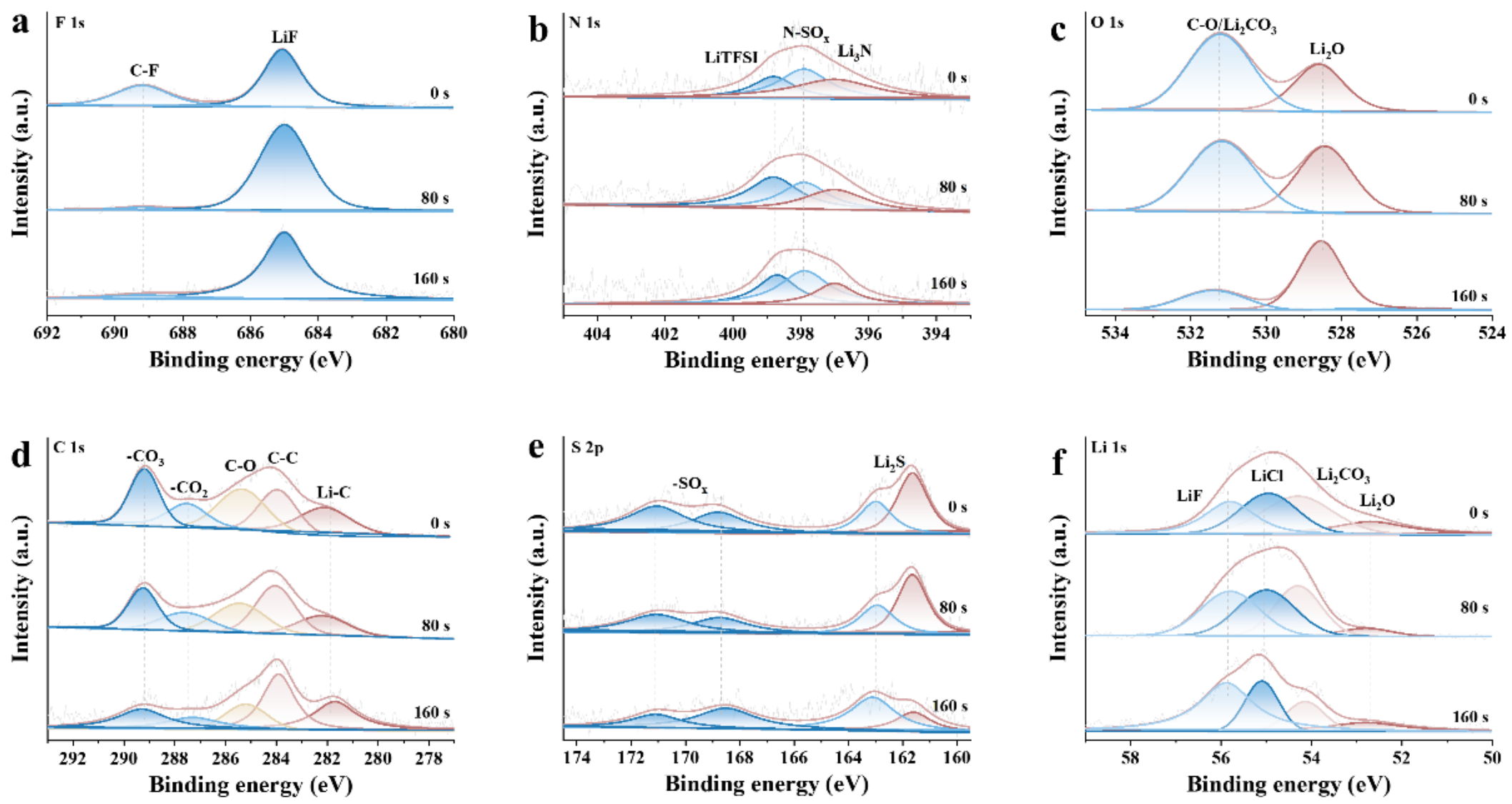

Fig. R3 | High-resolution XPS spectra of the SEI after cycling. (a) F 1s. (b) N 1s. (c) O 1s. (d) C 1s. (e) S 2p. (f) Li 1s.

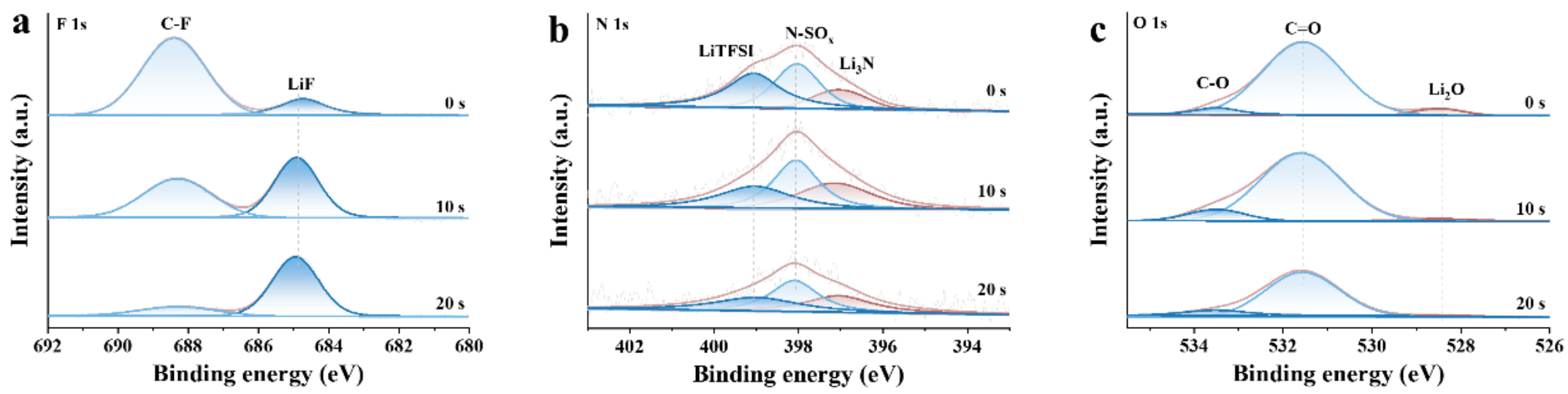

Fig. R4 | High-resolution XPS spectra of the CEI after cycling. (a) F 1s. (b) N 1s. (c) O 1s.

Please check the modified discussion on **Pages 10-11** in the main text, and the modified **Figs. S21-24** on **Pages 9-10** in the supplementary information.

2) The experimental details are very brief. Important details that effect electrochemical performance (e.g. Li foil thickness, coating method, electrolyte volume) as well as sample preparation procedures and instrument parameters for characterisation techniques would aid in reproducibility.

Response: Thank you for your constructive comments and valuable suggestions. Following your suggestions, we have revised the experimental section to include the missing details for improved reproducibility.

Please check the improved discussion on **Pages 21-23** in the main text.

3) The supplementary information requires editing. For example, KB is written as "Ketien blank" and NMP as "N-Methypyrrolidone" or energy-dispersive X-ray spectroscopy is abbreviated as EDX but subsequently referred to as EDS.

Response: We apologize for our oversight and thank the reviewer for pointing out these issues. The typos have been corrected, and the abbreviation of energy-dispersive X-ray spectroscopy has been unified as EDS throughout the manuscript to ensure consistency. The methods section has been moved to the main text.

Please check the corrected discussion on **Pages 21-22** in the main text.

To Reviewer #6:

The authors have addressed the majority of my concerns. This is good work and a good manuscript.

Response: Thank you for recognizing our work and recommending publication.
